# Modulating the Dimensions of Rectangular Hydrazone-Based Bispyridinium Macrocyclic Receptors

**DOI:** 10.1021/acs.joc.5c00695

**Published:** 2025-06-06

**Authors:** Natalia Fernández-Labandeira, Iván Montes de Oca, Elena Pazos, Arturo Blanco-Gómez, Carlos Peinador, Marcos D. García

**Affiliations:** CICA - Centro Interdisciplinar de Química e Bioloxía and Departamento de Química, Facultade de Ciencias, 16737 Universidade da Coruña, 15071 A Coruña, Spain

## Abstract

The development of new macrocyclic molecular receptors has driven major advances in supramolecular chemistry. However, realizing the full potential of host–guest systems requires addressing persistent challenges such as improved synthesis, aqueous performance, and implementation of stimuli-responsiveness. Herein, we present a new family of self-assembled polycationic molecular rectangles, derived from our previously reported *redbox* host. These novel cyclophanes share a common structural core with the parent compound—two pyridinium units linked by a hydrazone bond—but are designed with varying dimensions on the short sides, separating the bispyridinium walls. Synthesized via acid-catalyzed imine condensation reactions in water, these compounds were obtained in acceptable yields in gram scale through a modular and highly efficient approach. The aqueous molecular recognition properties of these new hosts were investigated using NMR spectroscopy with a 1,5-dihydroxynaphthalene derivative as a model aromatic electron donor. These studies demonstrate the formation of highly stable binary or ternary inclusion complexes in aqueous media, highlighting the tunability of the binding properties and applicative potential of this family of pH-responsive macrocyclic receptors.

## Graphical abstract

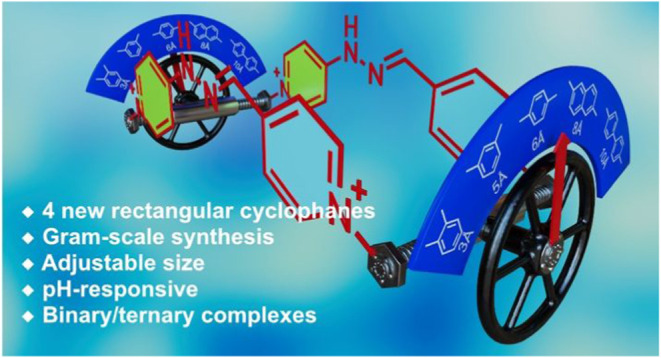

## 1 Introduction

Advances in the field of supramolecular chemistry have been parallel to the development of new abiotic macrocyclic hosts,^1^ with more than half a century of research leading to the design, synthesis and study of new artificial molecular receptors with improved capabilities in terms of the strength and selectivity of the recognition processes.^2^ Nevertheless, current macrocyclic-based supramolecular chemistry still faces certain unresolved issues such as the development of more efficient macrocyclization reactions,^3^ the discovery of new analogues able to exert their function in aqueous media efficiently,^4^ or to own intrinsic stimuli-responsiveness.^5^

In an effort to find solutions for some of the above-mentioned problems, we and others have recently exploited imine-forming reactions for the synthesis of bispyridinium-based macrocycles, cages and mechanically interlocked molecules in aqueous media.^6,7^ In essence, the cationic heterocyclic rings directly attached to the reacting carbonyl and/or amine counterparts act as electron sinks of the imine bonds formed, allowing for an adjustable hydrolytic lability of the obtained species by careful selection of the reacting partners. This strategy is reminiscent of Fujita’s “molecular-lock” used in Pt­(II)-directed self-assembly,^8^ as thermodynamic control can be reached in acidic aqueous media by heating, allowing the dynamic exchange of components under these conditions.^7d^ Importantly, the hydrolysis of the products formed is almost negligible when the system is cooled down, allowing the resulting imine-containing macrocyclic species to be easily isolated, purified, and characterized.

In particular, we have recently reported hydrazone-based analogues^7,9^ of the well-known Stoddart’s “little bluebox”,^10^ a cyclophane baptized as the *redbox* (**R**_**a**_H_2_^4+^, Scheme 1),^7b^ as well as related tricyclic,^7c^ *exo*-functionalized,^7d^ or simplified linear derivatives.^9^ In the particular case of **R**_**a**_H_2_^4+^, the species was found not only to act as a pH-based molecular switch in both aqueous and organic media, but also to translate this behavior into supramolecular responsiveness on the corresponding 1:1 inclusion complexes with aromatic electron donors. Crucially, the accessible p*K*_*a*_ = 8.7 of **R**_**a**_H_2_^4+^, responsible for the observed supramolecular behavior, could be correlated with the anomalously high stability of the imine bonds within the structure, in turn provoked by the high degree of electronic delocalization of the π-system on each of the long sides of the molecular rectangle.

**Scheme 1:**
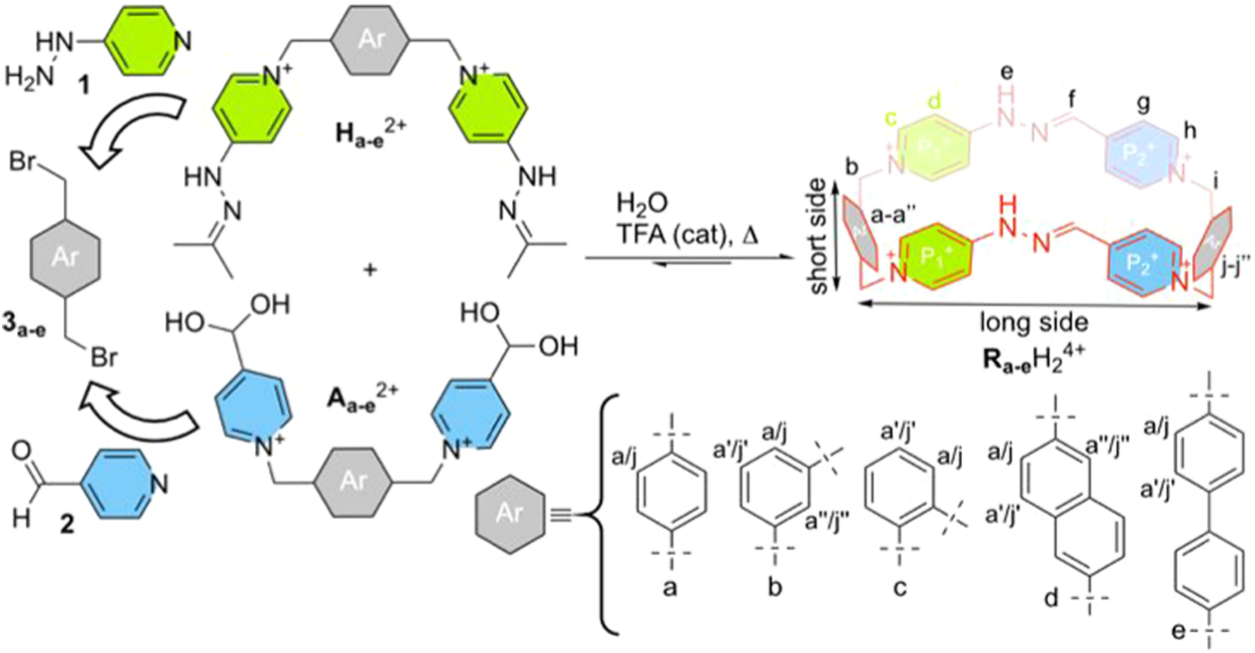
*Redbox* Analogues Discussed in This Work, Cyclophanes **R**_**a‑e**_H_2_^4+^

Following these previous results, we present herein a comprehensive study on the development of a new series of polycationic molecular rectangles, based on **R**_**a**_H_2_^4+^ and the following premises: to preserve the two dynamically linked bispyridinium subunits as pH-responsive molecular switches on the long sides and to modulate the distance between those on the short sides by using appropriate aromatic linkers. Consequently, as the main goal of our study, we envisioned the synthesis and structural characterization of **R**_**b‑e**_H_2_^4+^ analogues (Scheme 1), intended to be easily accessible from commercially available building blocks in a highly modular convergent fashion. Furthermore, in this work we will discuss the ability of the obtained macrocycles as molecular hosts, emphasizing the correlation between the dimensions of the long and short sides of the intended molecular rectangles and their ability to form host–guest aggregates of 1:1 (binary) or 1:2 (homoternary) stoichiometry.

## 2 Results and Discussion

### 2.1 Synthesis and Characterization of the *Redbox* Analogues

As shown in Scheme 1, **R**_**b‑e**_H_2_^4+^ could be accessed by a common strategy involving the hydrazone exchange reactions of matching bisaldehydes **A**_**b‑e**_^2+^ and bishydrazones **H**_**b‑e**_^2+^ in acidic aqueous media, which in turn could be easily prepared by reaction of commercially available 4-hydrazineylpyridine (**1**) or isonicotinealdehyde (**2**) as nucleophiles and bis­(bromomethyl) aromatics (**3**_**b‑e**_) as electrophiles. It should be noted that the reactions involving hydrazine **1** were carried out in refluxing acetone, not only to form the corresponding hydrazone, but also to avoid alkylation of the amine terminal group on the resulting salts. As expected, the ditopic building blocks were obtained in gram scale with good to excellent yields (56–99%), conveniently precipitated from the reaction mixtures in virtually pure form as the corresponding dibromides.^11^

With the above-mentioned building blocks in our hands, we proceeded to react each of the bishydrazones **A**_**b‑e**_^2+^ with its matching bisaldehydes **H**_**b‑e**_^2+^ (i.e., ensuring the same dimensions of both short sides on the resulting molecular rectangles) following our standard protocol, consisting on the condensation of 2.5 mM equimolar mixtures of the compounds in water at 60 °C and using 10% molar of trifluoroacetic acid as catalyst.^7,9^ The processes were easily monitored by ^1^H nuclear magnetic resonance (NMR), observing in all cases the consumption of the starting materials and the concomitant appearance of the diagnostic resonance for the newly formed imine bonds at ∼8.3 ppm (e.g., spectra for **R**_**b**_H_2_^4+^ in Figure 1a). After completion, the reactions were cooled to r.t. and an excess of KPF_6_ was added to the aqueous mixtures, resulting in the precipitation of the pyridinium-based cyclophanes due to the metathesis of the Br^–^ counterions. This process produced the macrocycles as PF_6_^–^ salts on a gram scale, with purities between 60 and 80%, as confirmed by analytical high-performance liquid chromatography (HPLC).^11^ After workup, all macrocycles as PF_6_^–^ salts were purified by reversed phase (semi)­preparative HPLC, yielding the trifluoroacetate salts in acceptable yields (22–45%).^11^

**Figure 1:**
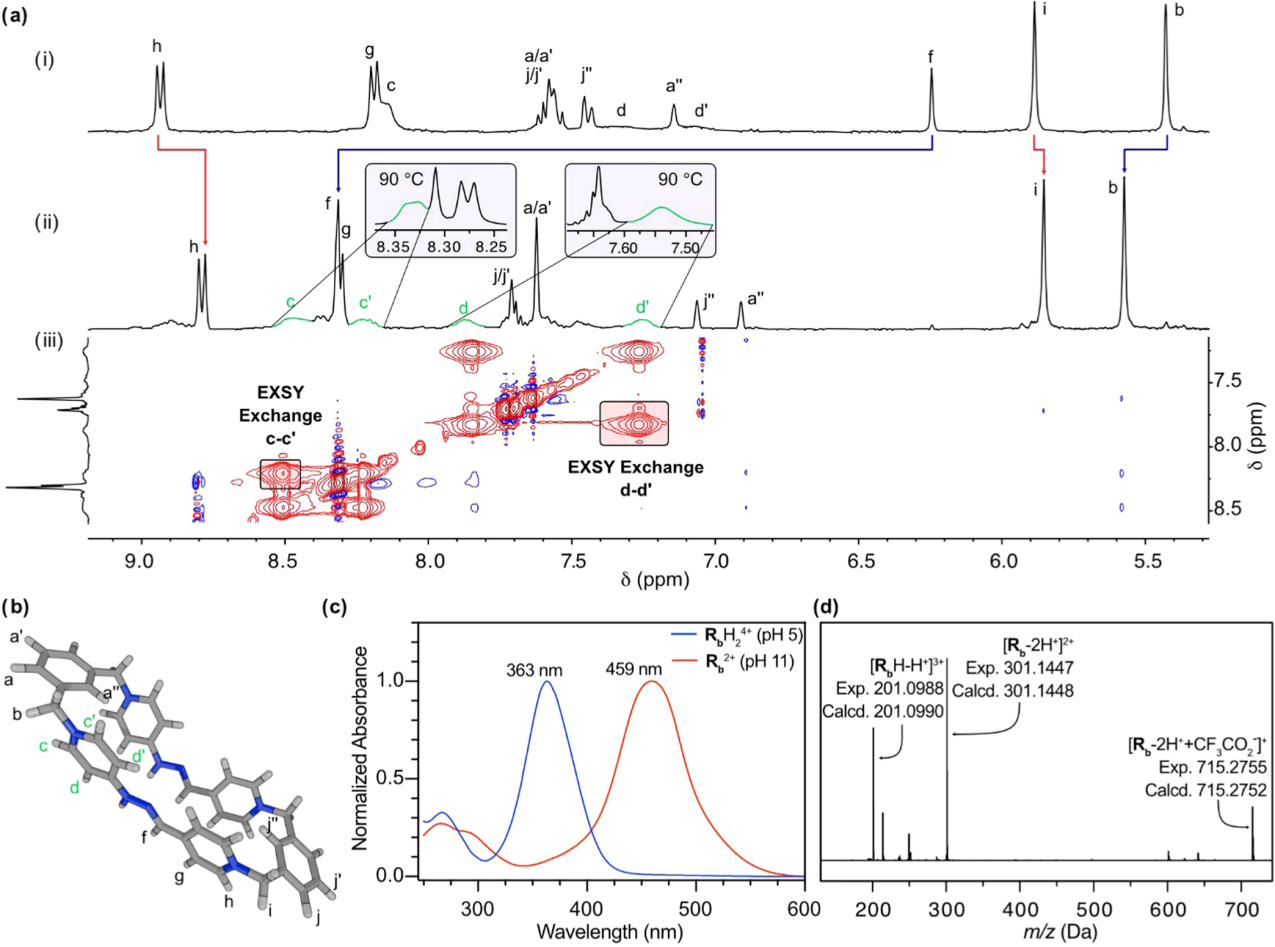
Structural characterization of **R**_**b**_H_2_^4+^: (a) Partially stacked ^1^H NMR spectra (D_2_O, 300 MHz) of (i) a 2.5 mM equimolar mixture of **H**_**b**_·2Br and **A**_**b**_·2Br at r.t. and *t* = 0; (ii) the same mixture after 24 h at 60 °C in the presence of TFA-*d*_3_ (10% molar); (iii) partial ^1^H–^1^H-NOESY spectrum of the previous mixture showing EXSY cross-peak between H_c‑c’_ and H_d‑d’_. (b) Sticks depiction of a representative minimum structure obtained for **R**_**b**_H_2_^4+^ at the r^2^SCAN-3c/CPCM­(water) level of theory, showing arbitrary labeling and the hampered rotation around the (P_1_^+^)­C–NHN bond. (c) Normalized UV–vis spectra of **R**_**b**_H_2_^4+^ (pH 5, blue solid line) and **R**_**b**_^2+^ (pH 11, red solid line). (d) HR-ESI-MS spectrum of **R**_**b**_H_2_·4TFA.

The cyclophanes **R**_**b,d,e**_H_2_·4TFA were extensively characterized in D_2_O and CD_3_CN by means of 1*D*/2D NMR techniques, as well as UV–vis and electrospray ionization mass spectrometry (ESI-MS) (as shown in Figure 1 for **R**_**b**_H_2_·4TFA),^11^ sharing a series of common features related to the anomalous hydrazone bonds formed, as previously reported for the parent macrocycle **R**_**a**_H_2_^4+^ and related analogues.^7,9^ First, the large delocalization of the amine lone pairs over the neighboring pyridinium rings results on the observation of restricted rotations around the (P_1_^+^)­C–NHN bonds in the ^1^H NMR of the compounds, manifested by the nonequivalence between the protons H_c‑c′/d‑d′_ positioned on the upper and lower sides of these heterocyclic rings (Figure 1b). This fact was verified by 2D-NOESY experiments,^11^ where EXSY cross-peaks can be observed in the spectra for the nuclei of the aminopyridinium moieties (Figure 1a), as a consequence of the slow exchange regime on the NMR time scale. In addition, VT ^1^H NMR experiments allowed the observation of a clear swap in the exchange rate for the hindered rotation from slow to rapid with increasing temperature, resulting in the merging of the signals for the interconverting nuclei (e.g., *insets* in Figure 1a). This fact allowed the estimation of an average Δ*G*_***r***_^**‡**^ value of 15.3 ± 0.2 kcal/mol for **R**_**b,d,e**_H_2_·4TFA, using the coalescence method,^11^ in good agreement with previously reported data.^7,9^ Finally, another quite distinctive structural feature of the macrocycles can also be deduced from the NMR data for **R**_**b,d,e**_H_2_·4TFA, namely the significantly deshielded resonances for the imine protons (8.11–8.31 ppm), resulting from the highly delocalized nature of the hydrazone bonds directly connected to two π-deficient pyridinium rings.

Regarding the HR-ESI-MS data recorded for **R**_**b,d,e**_H_2_·4TFA, they show the loss of TFA counterions and protons typical for this type of structures (e.g., Figure 1d for **R**_**b**_·H_2_·4TFA),^7,9^ which clearly correlates with the abnormal acidity of the NH groups on the molecular rectangles. This unusual acidity is also manifested in the UV–vis spectra of the species (e.g., Figure 1c for **R**_**b**_H_2_·4TFA). As previously reported for **R**_**a**_H_2_·4TFA and related hydrazones,^7c^ ^–e^ the spectra for the compounds in buffered aqueous solutions showed distinctive main absorption bands at λ_max_ ∼ 363–372 nm (pH = 5). These bands clearly disappear under basic conditions (pH = 11), resulting in the concomitant appearance of new main absorption bands centered at λ_max_ ∼ 459–468 nm, responsible for the characteristic red color of these *redbox* analogues.

Despite all the common features discussed for the characterization of **R**_**b,d-e**_H_2_·4TFA, those for the contracted analogue **R**_**c**_H_2_·4TFA deserve further comments. First, its HR-ESI-MS spectrum clearly corroborates the formation of the compound, showing the same features as the rest of the series (Figure S84). However, a more complex situation was observed in the NMR data for the macrocycle in D_2_O at r.t., with the ^1^H NMR spectrum recorded at 2.5 mM displaying all the signals for the cyclophane broadened, being particularly striking the appearance of multiple signals attributable to the CH_2_ groups on the structure (Figure 2a). Considering the possible presence of atropisomers for the species (*vide infra*), and the short distance between the two long sides of the cyclophane, rotation around the bonds involving the C­(*sp* ^3^) corners could certainly be hindered, in which case a situation of slow equilibrium between the potential different structural conformers on the NMR time scale could result, as has been observed with other *ortho*-substituted macrocyclic analogues.^12^ To verify this, and to discard the formation of other potential species (i.e., oligomers), we carried out VT ^1^H-RMN experiments in D_2_O in the temperature window 5–95 °C, which showed the successive simplification of the spectra upon heating (Figure S78). Hence, the signals in a near-coalescence regime at r.t. increasingly collapse into a single set of well-resolved resonances at 85 °C (Figure 2a), in good agreement with a situation of fast exchange equilibrium as that observed for the other analogues in the series.

**Figure 2:**
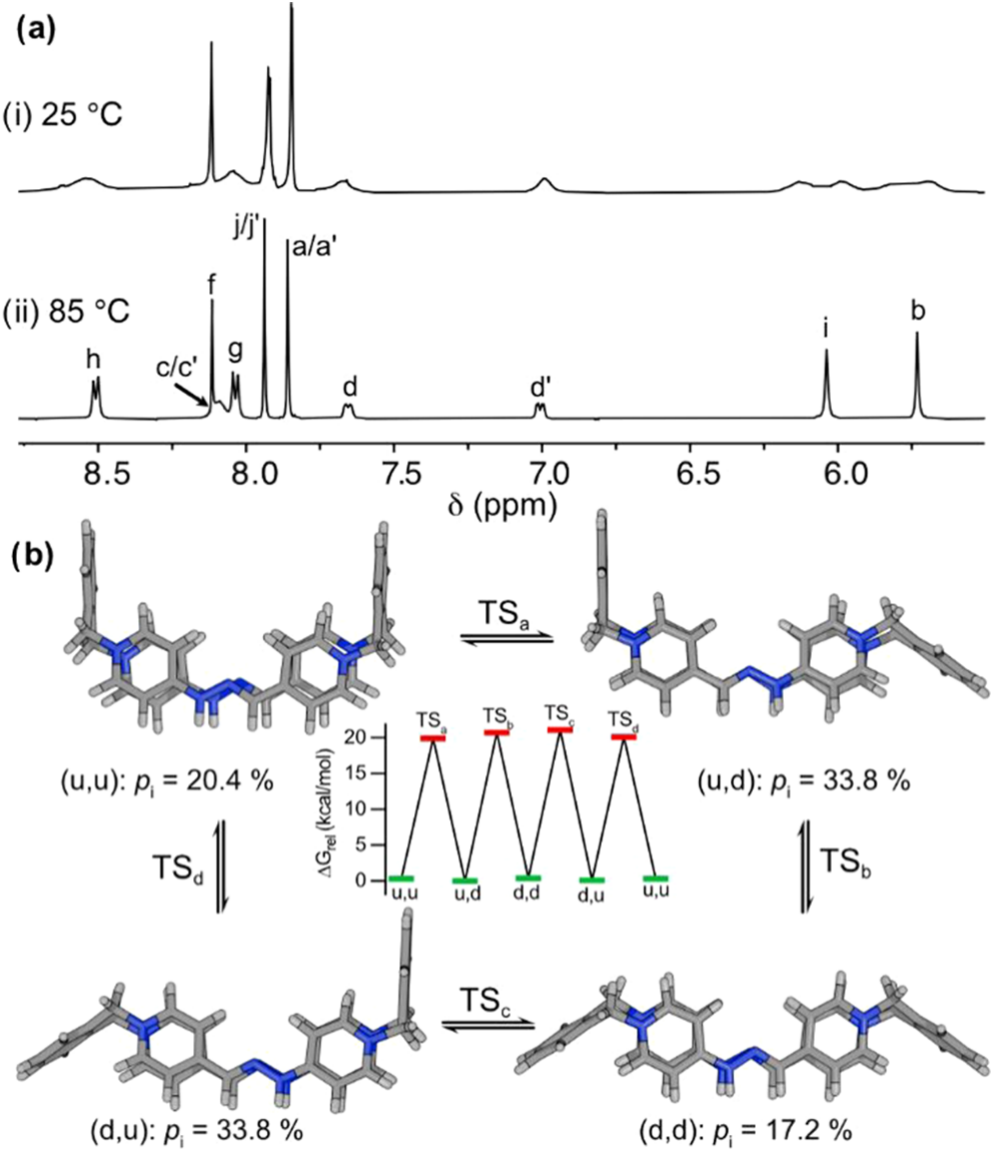
(a) Partially stacked ^1^H NMR spectra of **R**_**c**_H_2_·4TFA at 2.5 mM (D_2_O, 500 MHz): (i) at 25 °C and (ii) at 85 °C. (b) Schematic representation of the four conformational transitions between the four atropisomers of **R**_**c**_H_2_^4+^ obtained at the r^2^SCAN-3c/CPCM­(water) level of theory, including the arbitrary labeling and Boltzmann populations *p*_i_ at r.t. Inset: Free energy profile for the interconversion processes.

To obtain more information on the structural nature of the observed isomers, dispersion-corrected density functional theory (DFT) methods were used to study *in silico* the potential conformations of the macrocycle. By using the recently developed CREST^13^/CENSO^14^ protocol developed by Grimme et al., a representative conformational ensemble for the compound was obtained at the r^2^SCAN-3c^15^/CPCM^16^ (water) level of theory. This computation produced four distinctive representative minima on the conformational potential energy surface (PES) of the macrocycle, showing four atropisomeric species quite evenly populated at r.t. and differing on the relative *anti*/*syn* disposition of the phenylene rings of the macrocycles (arbitrarily labeled as up (u) or down (d), Figure 2b). As shown, using the well-established NEB-TS protocol,^17^ we were able to locate as well appropriate transition states for the four potential conversions between conformers at the same level of theory, leading to an average free energy barrier of 20.4 ± 0.5 kcal/mol for the conformational transition between atropisomers, in good agreement with the complex ^1^H NMR data obtained at r.t. for **R**_**c**_H_2_·4TFA.

### 2.2 Host–Guest Chemistry of the *Redbox* Analogues

Given the high affinity of **R**_**a**_H_2_^4+^ in water for electron-rich aromatic substrates (*K*_a_ ∼ 10^4^ M^–1^), which is mainly associated with the hydrophobic effect and, to a lesser extent, to π–π and C–H···π interactions,^7b^ in the present work we decided to study the complexation ability of the analogues **R**_**b‑e**_H_2_·4TFA in water, using the naphthalene derivative **4** as a model aromatic substrate due to its relatively high solubility in water and its π-donor nature. For this purpose, we performed 1*D*/2D NMR and DOSY experiments, starting from macrocycles at pD 5 (20 mM phosphate buffer) to ensure complete protonation of the *redbox* analogues.^18^

As expected, in the case of the smallest macrocycle **R**_**c**_H_2_·4TFA, the addition of 1 equiv of **4** to a 2.5 mM solution of the macrocycle resulted in no changes in the ^1^H NMR signals (Figure S139). As measured on the structure of the above-discussed representative minima calculated for the macrocycle at the DFT level (Figure 2b), the bispyridinium long sides of the macrocycle lay on almost parallel planes, but with effective distances between these moieties of only 1.9 Å, which would render a receptor with an insufficiently wide cavity for the complexation of the aromatic substrate.

Conversely, for **R**_**b**_H_2_·4TFA, the obtained data were more similar to that originally reported for the complexation of **4** by the *redbox*.^7b^ In this case, the DOSY experiment of a 2 mM equimolar mixture of **4** and the macrocycle showed a single diffusion of both species in solution, supporting the complexation of the guest (Figure 3c). Furthermore, the accompanying ^1^H NMR spectrum in these conditions showed typical complexation-induced chemical shifts (CISs), for a situation of fast exchange between the interacting species on the NMR time scale (Figure 3c). Regarding the guest (Table 1), the corresponding CISs showed the characteristic shielding effect for all the protons on the aromatic part of **4**, produced as a result of the insertion of the electron donor moiety within the hydrophobic cavity of **R**_**b**_H_2_·4TFA. This effect is more pronounced for H_2–3_, due to the establishment of C–H···π interactions and the presumable tilted insertion mode of the aromatic scaffold in the cavity of the host.^19^ This interaction mode is reproduced in Figure 3a for **4t**⊂**R**_**b**_H_2_^4+^, a minimum on the PES at the r^2^SCAN-3c^15^/CPCM^16^ (water) for a simplified analogue of our complex, conveniently truncated in the polyethylene-glycol chains of the guest **4**. Finally, a ^1^H-RMN titration of **R**_**b**_H_2_·4TFA (2 mM) with **4** confirmed the formation of the 1:1 complex, rendering an association constant in the order of 10^4^ M^–1^ (Figure 3b). This value is quite similar to that reported for the parent macrocycle **R**_**a**_H_2_^4+^ and reflects that the slight narrowing of the cavity of **R**_**b**_H_2_^4+^ compared to the original *redbox* does not significantly affect its ability to complex aromatic electron donors at pD 5 (see Table 1).

**Figure 3:**
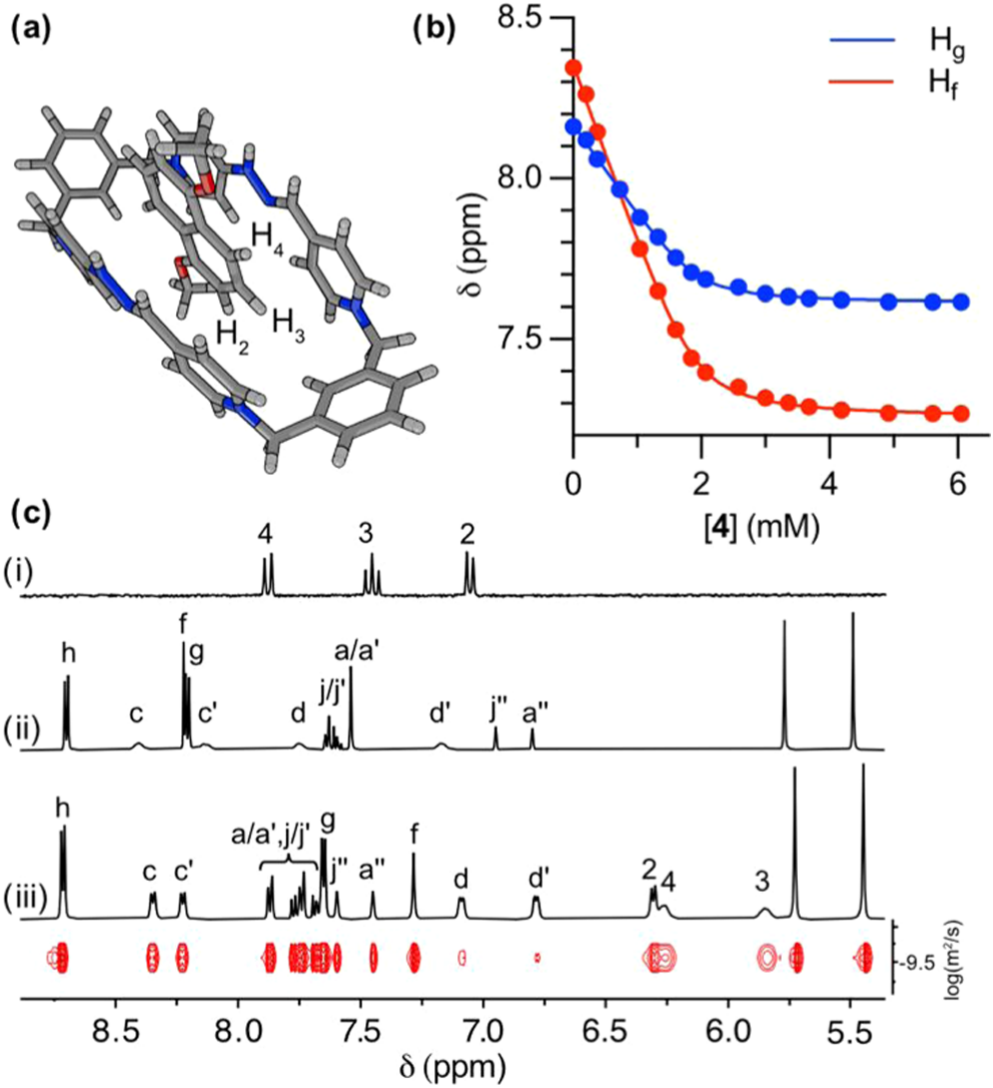
(a) Schematic depiction of a representative minimum on the PES of **4t**⊂**R**_**b**_H_2_^4+^ at the r^2^SCAN-3c/CPCM­(water) level of theory, showing a tilted binding mode of the 1,5-dihydroxynahpthalene moiety. (b) Fitting of the ^1^H NMR signals of H_f‑g_ of **R**_**b**_H_2_·4TFA during titration with increasing concentrations of **4**. (c) Partially stacked ^1^H NMR spectra (D_2_O, 500 MHz) of: (i) **4**; (ii) **R**_**b**_H_2_·4TFA; (iii) a 2 mM equimolar mixture of **R**_**b**_H_2_·4TFA and **4**; DOSY experiment (D_2_O, 500 MHz) of the previous mixture is shown at the bottom.

**Table 1:**
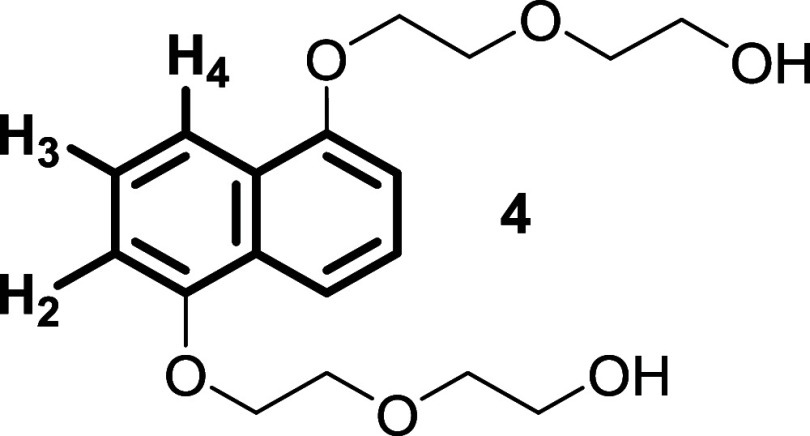
Complexation-Induced Shifts (Δδ *=* δ_free_ *–*δ_complexed_) for the Interactions between **R**_**a‑e**_H_2_·4TFA and **4** in Aqueous Media, Association Constant Values, and Stoichiometry Derived from the Corresponding NMR Titration Experiments^a^

	Δδ ^1^H macrocycle (ppm)					Δδ ^1^H **4** (ppm)				
macrocycle	H_g_	H_f_	H_a/j_	H_a′/j′_	H_a″/j″_	H_2_	H_3_	H_4_	*K*_a_	G⊂H stoichiometry
**R**_**a**_H_2_·4Cl^b^	–0.70	–0.91	0.16/0.17			**–1.26**	–**1.99**	–1.24	(1.61 ± 0.14) × 10^4^ M^–1^	1:1
**R**_**b**_H_2_·4TFA	–0.56	–0.94	0.20/0.25	0.14/0.15	0.65/0.64	–0.75	–**1.61**	–**1.61**	(1.54 ± 0.11) × 10^4^ M^–1^	1:1
**R**_**c**_H_2_·4TFA									na^c^	
**R**_**d**_H_2_·4TFA	–0.61	–0.78	0.23/0.27	0.27/0.32	0.18/0.23	–**1.14**	–**1.17**	–0.98	(1.63 ± 0.22) × 10^3^ M^–1^ ^d^	2:1
									(4.93 ± 0.87) × 10^3^ M^–1^ ^e^	
**R**_**e**_H_2_·4TFA	–0.69	–0.61	0.21/0.23	0.24/0.27		–**1.73**	–**1.71**	–1.30	(6.52 ± 0.80) × 10^3^ M^–1^ ^d^	2:1
									(6.50 ± 1.50) × 10^3^ M^–1^ ^e^	

a Bold values for **4** indicate the hydrogens that establish C–H···π interactions.

b Previously reported (ref 7b).

c No association observed.

d Value of the stepwise association constant *K*_1_.

e *K*_2_.

The two macrocycles **R**_**d,e**_H_2_·4TFA have an additional aromatic unit in the short sides of the corresponding molecular squares when compared to the parent *redbox*. Consequently, these have dimensions that potentially allow the inclusion of two aromatic molecules in a parallel π–π stacking arrangement, as has been reported for structurally related molecular receptors of similar size.^10^ Consequently, appropriate mixtures of 2 equiv of **4** and **R**_**d,e**_H_2_·4TFA (1.3 mM) were studied by ^1^H NMR in D_2_O, with the corresponding DOSY experiments showing a single diffusion for the interacting species in each case and the ^1^H NMR data being in good agreement with host–guest complexes being formed in solution under a fast equilibrium regime (e.g., 2:1 mixture of **4**:**R**_**d**_H_2_·4TFA, Figure 4c). ^1^H NMR titrations of 1.3 mM **R**_**d,e**_H_2_·4TFA with **4** were carried out, with the fitting of the observed changes in the chemical shifts of H_f–g_ (e.g., for **R**_**d**_H_2_·4TFA and **4**, Figure 4b) indicating the formation of 2:1 host–guest complexes. A detailed analysis of the binding isotherms revealed a cooperative binding process, as evidenced by the deconvolution of the overall binding into two distinct stepwise association constants *K*_1_ and *K*_2_ in the order of 10^3^ M^–1^ (Table 1). These results suggest that binding of the first guest molecule facilitates the association of the second, especially in the case of **4**_2_⊂**R**_**d**_H_2_^4+^. Additionally, attempts to fit the data using a noncooperative 2:1 model were unsuccessful,^11^ further supporting the presence of positive cooperativity.^20^ Regarding the observed CISs for **4**_2_⊂**R**_**d,e**_H_2_^4+^, these were estimated on the basis of extensive 1*D*/2D NMR experiments for a mixture of guest and host, and in this case reflect a longitudinal insertion mode of the aromatic scaffold of **4** within **4**_2_⊂**R**_**d,e**_H_2_^4+^, as illustrated in Figure 4a for the DFT-optimized structure of the truncated analogue **4t**_2_⊂**R**_**d**_H_2_^4+^.

**Figure 4:**
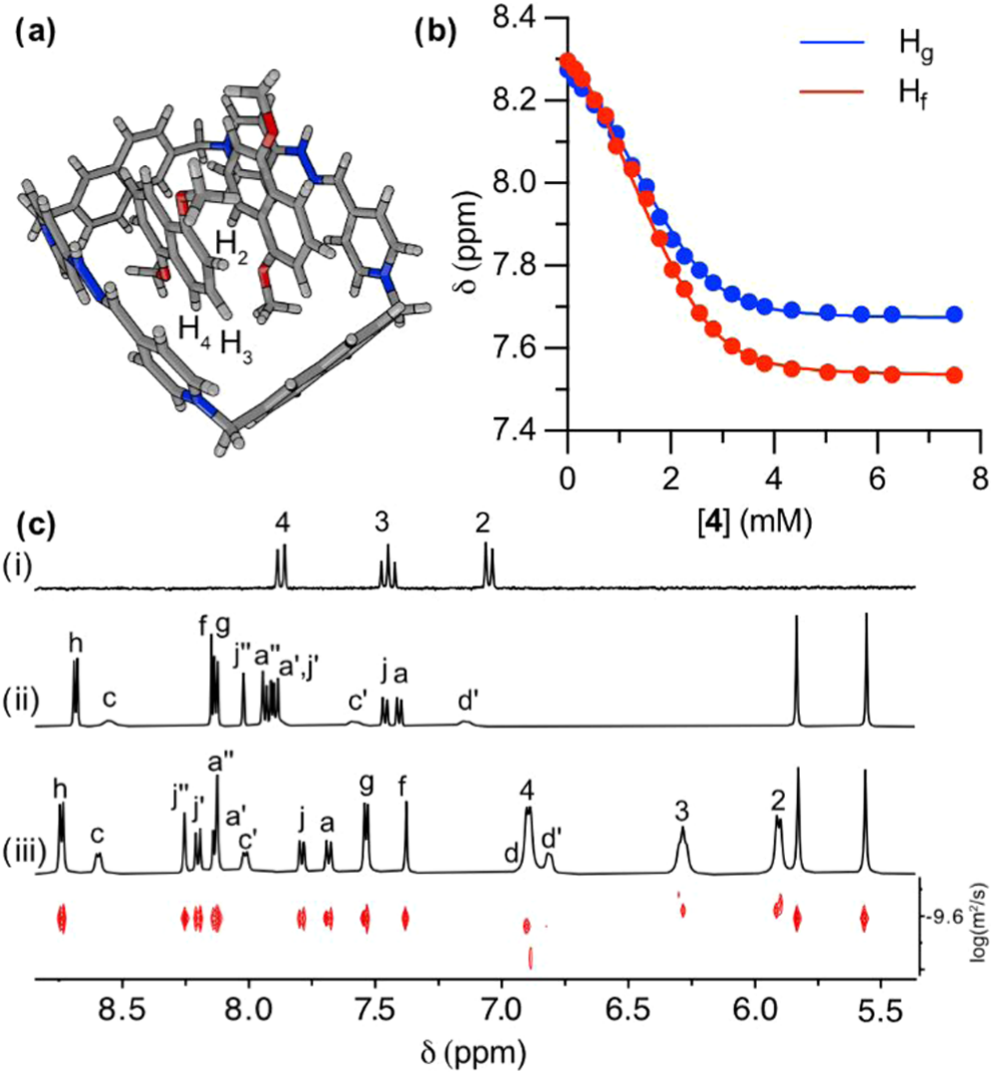
(a) Schematic depiction of a representative minimum on the PES of **4t**_2_⊂**R**_**d**_H_2_^4+^ at the r^2^SCAN-3c/CPCM­(water) level of theory, showing a longitudinal binding mode of the π-stacked 1,5-dihydroxynahpthalene moieties. (b) Fitting of the ^1^H NMR signals of H_f‑g_ of **R**_**d**_H_2_·4TFA during titration with increasing concentrations of **4**. (c) Partially stacked ^1^H NMR spectra (D_2_O, 500 MHz) of (i) **4**; (ii) **R**_**d**_H_2_·4TFA; (iii) a 1.3 mM mixture of **R**_**d**_H_2_·4TFA and **4**; DOSY experiment (D_2_O, 500 MHz) of the previous mixture is shown at the bottom.

## 3 Conclusions

In this work we have reported the extension of the family of *redbox* pH-responsive cyclophanes, by synthesizing four new rectangular analogues with different cavity dimensions when compared to the parent macrocycle. The synthesis of these new cyclophanes has been achieved on a gram scale in good to excellent yields, by hydrazone-forming reactions conducted in aqueous media under acid catalysis and heating. The kinetically inert self-assembled macrocycles show very similar structural features compared to their parent compound, with the exception of the smaller analogue **R_c_** H_2_·4TFA, which shows different slow-exchanging atropisomeric conformers at r.t., as supported by dispersion-corrected DFT calculations. All the macrocycles obtained, again with the exception of **R_c_** H_2_·4TFA, formed inclusion complexes with the electron-rich aromatic molecule **4** in aqueous media, with the dimensions of their inner cavities controlling the stoichiometry of the host–guest complex with the model guest. Hence, while the slight reduction in the dimensions of the *redbox* in **R**_**b**_H_2_^4+^ does not alter the ability of the latter to form binary complexes, the introduction of extra aromatic moieties in **R_d,e_** H_2_^4+^ leads to ternary complexes with association constants in the 10^7^ M^–2^ range. Overall, our results show the high efficiency of our modular imine-based approach for the construction of pH-responsive molecular receptors of adjustable size, hosts that are currently being investigated in our lab for the construction of stimuli-responsive mechanically interlocked molecules in aqueous media.

## Supplementary Material





## Data Availability

The data underlying this study are available in the published article and its Supporting Information.

## Notes

Cartesian coordinates for the geometries (ZIP)
General experimental details, detailed synthetic procedures, NMR spectra, spectroscopic characterization data, association constants determination, and computational details (PDF)
