# Supplementary material for: Modulating the Dimensions of Rectangular Hydrazone-Based Bispyridinium Macrocyclic Receptors
Source: J Org Chem. 2025 Jun 6;90(25):8621–7. doi: 10.1021/acs.joc.5c00695 (PMC12210216; doi:10.1021/acs.joc.5c00695)
Supplement: Supplementary file 2 [file jo5c00695_si_002.pdf]

Supporting Information

Modulating the dimensions of rectangular hydrazone-based bispyridinium macrocyclic receptors

Natalia Fernández-Labandeira, Iván Montes de Oca, Elena Pazos, Arturo Blanco-Gómez, Carlos Peinador,* and Marcos D. García*

CICA – Centro Interdisciplinar de Química e Bioloxía and Departamento de Química, Facultad de Ciencias. Universidade da Coruña, 15071, A Coruña, Spain.

Corresponding Author's Email: carlos.peinador@udc.es; marcos.garcia1@udc.es

1. EXPERIMENTAL SECTION S4

1.1. General procedures S4

1.2. Synthetic procedures S5

1.2.1. Synthesis and characterization data of A_b·2Br S5

1.2.2. Synthesis and characterization data of A_c·2Br S9

1.2.3. Synthesis and characterization data of A_d·2Br S13

1.2.4. Synthesis and characterization data of A_e·2Br S18

1.2.5. Synthesis and characterization data of H_b·2Br S23

1.2.6. Synthesis and characterization data of H_c·2Br S28

1.2.7. Synthesis and characterization data of H_d·2Br S32

1.2.8. Synthesis and characterization data of H_e·2Br S37

1.2.9. Synthesis and characterization data of R_bH_2·4TFA S42

1.2.10. Synthesis and characterization data of R_cH_2·4TFA S53

1.2.11. Synthesis and characterization data of R_dH_2·4TFA S59

1.2.12. Synthesis and characterization data of R_eH_2·4TFA S69

1.3. Determination of the energy of the rotational barrier ($\Delta G^{\neq}$) S79

1.3.1. $\Delta G^{\neq}$ for R_bH_2·4TFA S79

1.3.2. $\Delta G^{\neq}$ for R_cH_2·4TFA S80

1.3.3. $\Delta G^{\neq}$ for R_dH_2·4TFA S81

1.3.4. $\Delta G^{\neq}$ for R_eH_2·4TFA S82

1.4. UV-vis absorption spectra of $R_{b\text{-}e}H_2$·4TFA S83

1.4.1. UV-vis absorption spectrum of R_bH_2·4TFA S83

1.4.2. UV-vis absorption spectrum of R_cH_2·4TFA S83

1.4.3. UV-vis absorption spectrum of R_dH_2·4TFA S84

1.4.4. UV-vis absorption spectrum of R_eH_2·4TFA S84

2. HOST-GUEST STUDIES S85

2.1. Synthesis and characterization data of $4{\subset}R_bH_2$□4TFA S85

2.1.1. $4{\subset}R_bH_2$□4TFA at pD 5 .... S85

2.1.2. $4{\subset}R_bH_2$□4TFA at pD 11 .... S92

2.2. Verification of the non-formation of $4{\subset}R_cH_2$□4TFA S95

2.3. Synthesis and characterization data of $4_2{\subset}R_dH_2$□4TFA S96

2.4. Synthesis and characterization data of $4_2{\subset}R_eH_2$□4TFA S105

2.5. DOSY spectrum of 4□R_bH_2·4TFA /4_2□$R_{d\text{-}e}H_2$·4TFA S114

2.5.1. DOSY spectrum of 4□R_bH_2·4TFA S114

2.5.2. DOSY spectrum of 4_2□R_dH_2·4TFA S115

2.5.3. DOSY spectrum of 4_2□R_eH_2·4TFA S115

3. COMPUTATIONAL DETAILS S116

1. EXPERIMENTAL SECTION

1.1. General procedures

Starting materials were purchased from commercial suppliers and used without further purification. Compound 4-(2-(propan-2-ylidene)hydrazineyl)pyridine was prepared according to published procedures[1]. Milli-Q water was purified with a Millipore Gradient A10 apparatus. Merck 60 F254 foils were used for thin layer chromatography, and Merck 60 (230-400 mesh) silica gel was used for flash chromatography. When the products were lyophilized, a Telstar Cryodos -80 lyophilizer was used. All compounds were characterized using a Bruker Avance 500 and Bruker Avance III HD 400 spectrometers (400/500 MHz for ^{1}H and 101/126 MHz for ^{13}C) of the SAI (Research Support Services). NMR spectra were performed using D_2O and CD_3CN as solvent and taking the signal of the protonated solvent as reference. Mass spectrometry experiments were carried out in a LCQ-q-TOF Applied Biosystems QSTAR Elite spectrometer for low and high resolution ESI. UV/Vis spectra were recorded on a Jasco V-650 spectrometer. Titration experiments were carried out in 20 mM sodium phosphate buffer at 25 °C on a Bruker Advance 400 MHZ. pH was measured using a Fisherbrand accumet AE150 pHmeter microsamples with Ag/AgCl reference element. HPLC-MS analysis was performed using a Thermo Scientific UltiMate 3000 connected to a photo-diode array (PDA) detector and a single quadrupole mass spectrometer Thermo Scientific MSQ Plus, using an Aeris analytical column from Phenomenex (peptide XB-C18 stationary phase, 3.6 µm, 100 Å pore size, 150 × 2.1 mm). Melting points were measured using a SMP3 Stuart Scientific apparatus and are uncorrected. Structural assignments were made with additional information from gNOESY, gCOSY, gHSQC, gHMBC and gDOSY experiments.

[1] A. Blanco-Gómez, A., I. Neira, J. L. Barriada, M. Melle-Franco, C. Peinador, M. D. García, Thinking outside the "Blue Box": from molecular to supramolecular pH-responsiveness. *Chem. Sci.* **2019**, *10*(46), 10680–10686.

1.2. Synthetic procedures

1.2.1. Synthesis and characterization data of $\mathbf{A_b}$·2Br

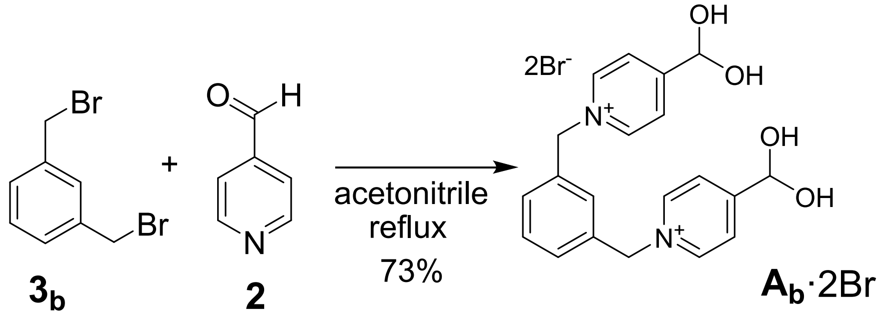

A solution of *α*,*α*'-dibromo-m-xylene ($\mathbf{3_b}$, 1.54 g, 5.85 mmol, 1 eq) and 4-pyridinecarboxaldehyde (**2**, 1.67 mL, 17.55 mmol, 3 eq) in 180 mL of acetonitrile was heated at reflux in a magnetic hot plate stirrer for 20 h. The resulting precipitated is filtered, washed with acetonitrile (3×30 mL), diethyl ether (3×30 mL) and dried under vacuum, yielding $\mathbf{A_b}$·2Br as a copper-coloured viscous liquid (2.20 g, 73%).

mp 239.7 – 240.8°C (decomposition). **^{1}H NMR** (400 MHz, D_2O) δ (ppm): 8.91 (d, J = 6.7, 4H), 8.15 (d, J = 6.8, 4H), 7.55 (m, 4H), 6.20 (s, 2H), 5.86 (s, 4H). **^{13}C{1H} NMR** (101 MHz, D_2O) δ (ppm): 160.4 (C), 144.7 (CH), 133.9 (C), 130.7 (CH), 130.4 (CH), 129.4 (CH), 125.6 (CH), 87.5 (CH), 63.6 (CH_2). **HRMS** (**ESI**) m/z: [$\mathbf{A_b}$+2CH_3]$^{2+}$ Calcd for $C_{22}H_{26}N_2O_4^{2+}$ 191.0941; Found 191.0940; and [$\mathbf{A_b}$+Br]$^+$ Calcd for $C_{22}H_{26}BrN_2O_4^+$ 461.1070; Found, $C_{22}H_{26}N_2O_4^{2+}$ 461.1070.

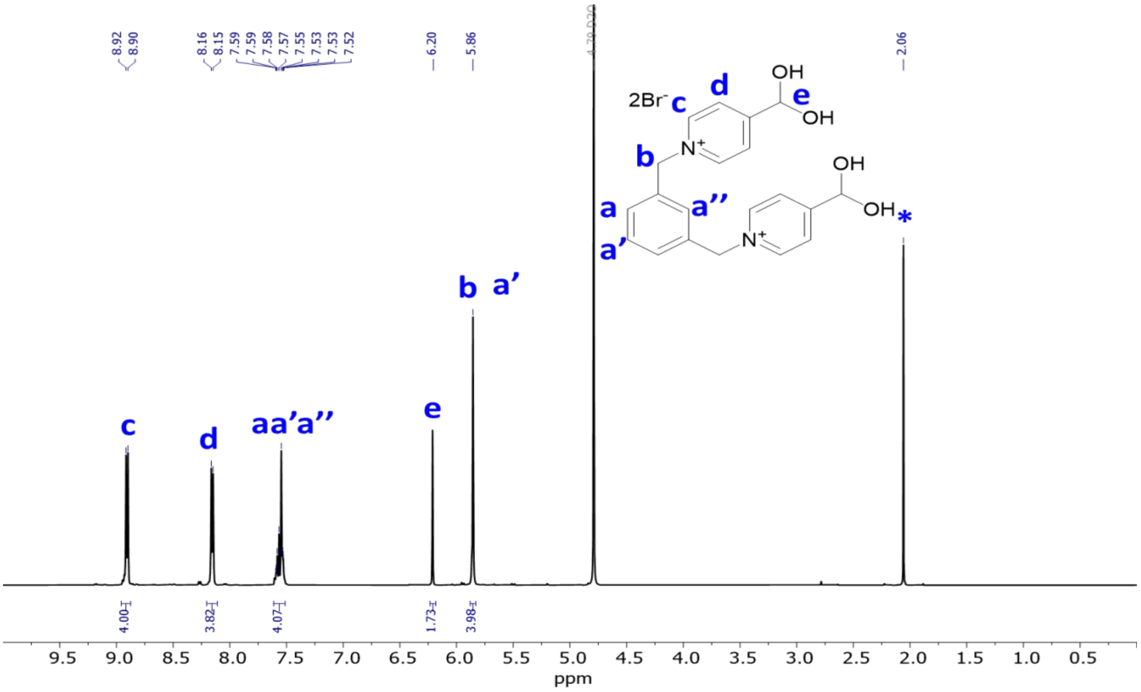

Figure S 1. ^{1}H NMR (400 MHz, D_2O) spectrum of $\mathbf{A_b}$·2Br. Impurities marked as *: acetone (2.06 ppm, s)

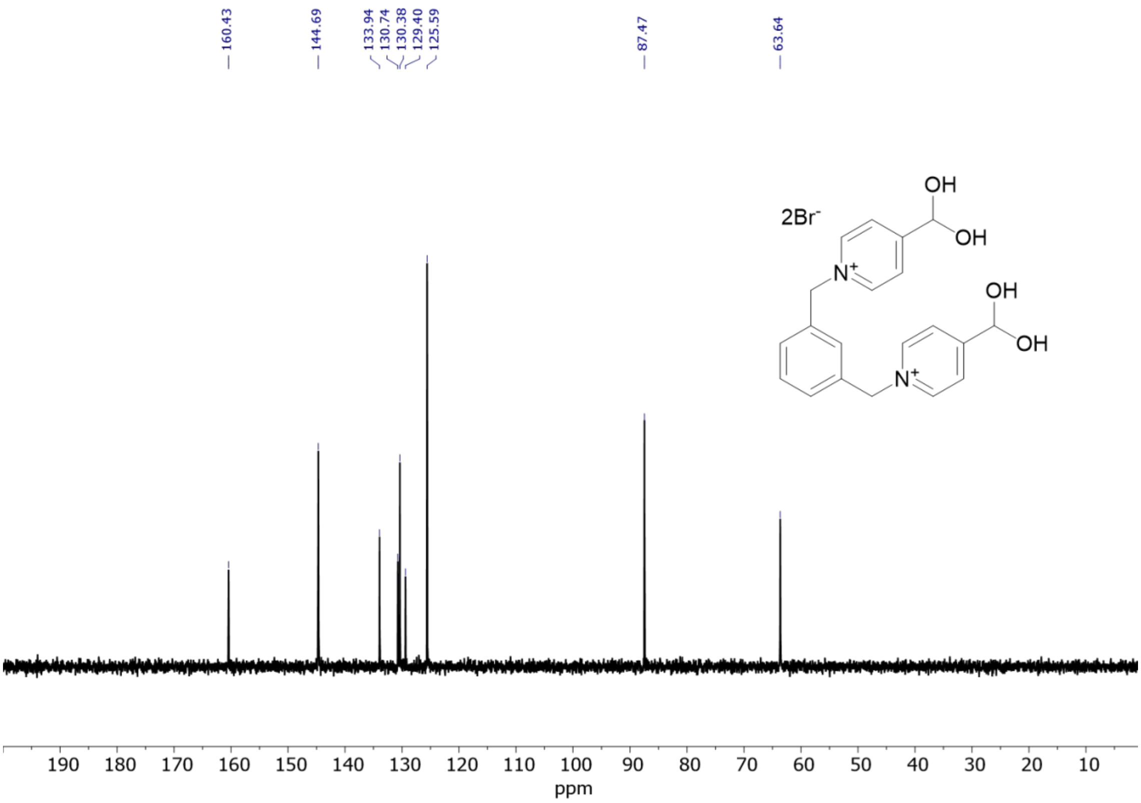

Figure S 2. ^{13}C{1H} NMR (101 MHz, D_2O) spectrum of of $\mathbf{A_b}$·2Br

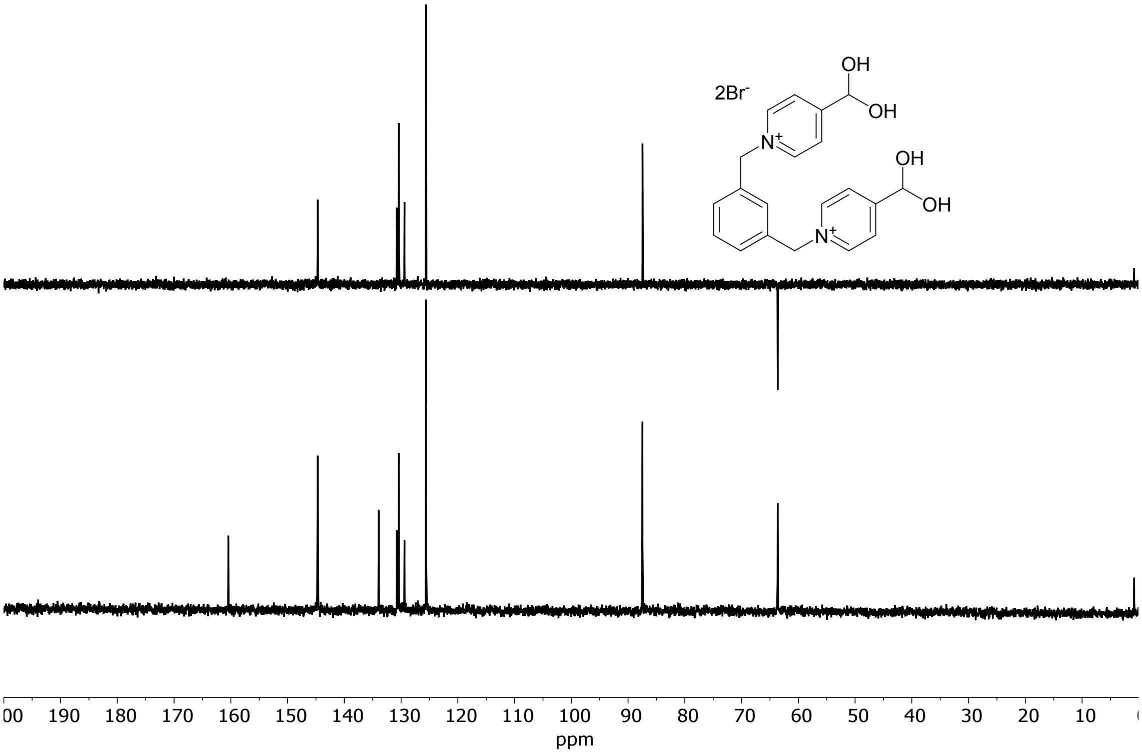

Figure S 3. ^{13}C{1H} NMR (101 MHz, D_2O) spectrum (top) and DEPT-135 (101 MHz, D_2O) spectrum (bottom) of $\mathbf{A_b}$·2Br

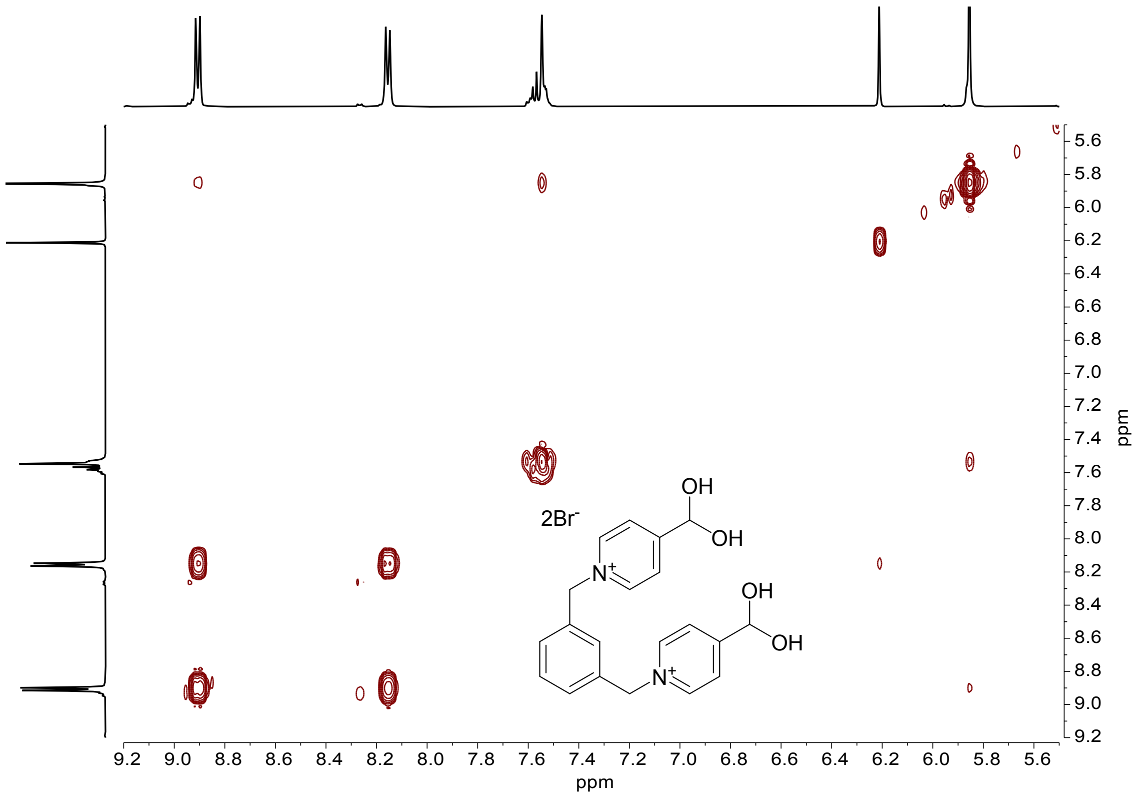

Figure S 4. ^{1}H-^{1}H COSY (400 MHz, D_2O) spectrum of $\mathbf{A_b}$·2Br

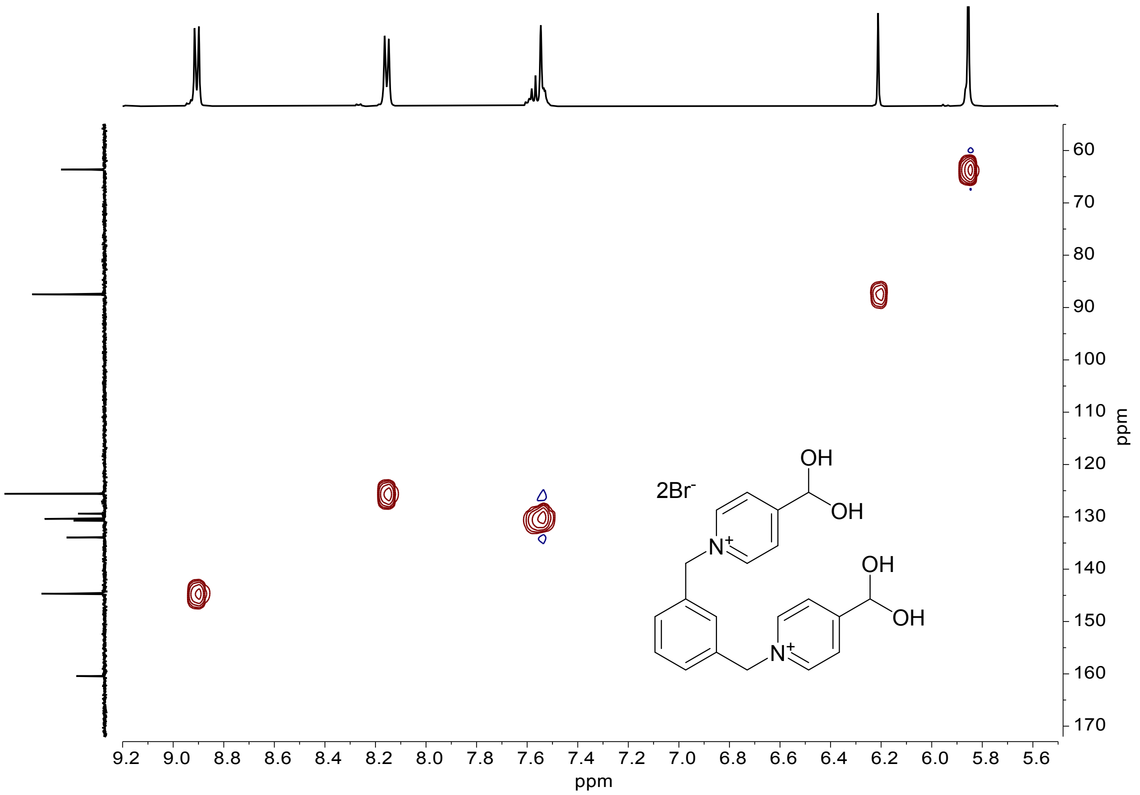

Figure S 5. ^{1}H-^{13}C HSQC (400 MHz/101 MHz, D_2O) spectrum of $\mathbf{A_b}$·2Br

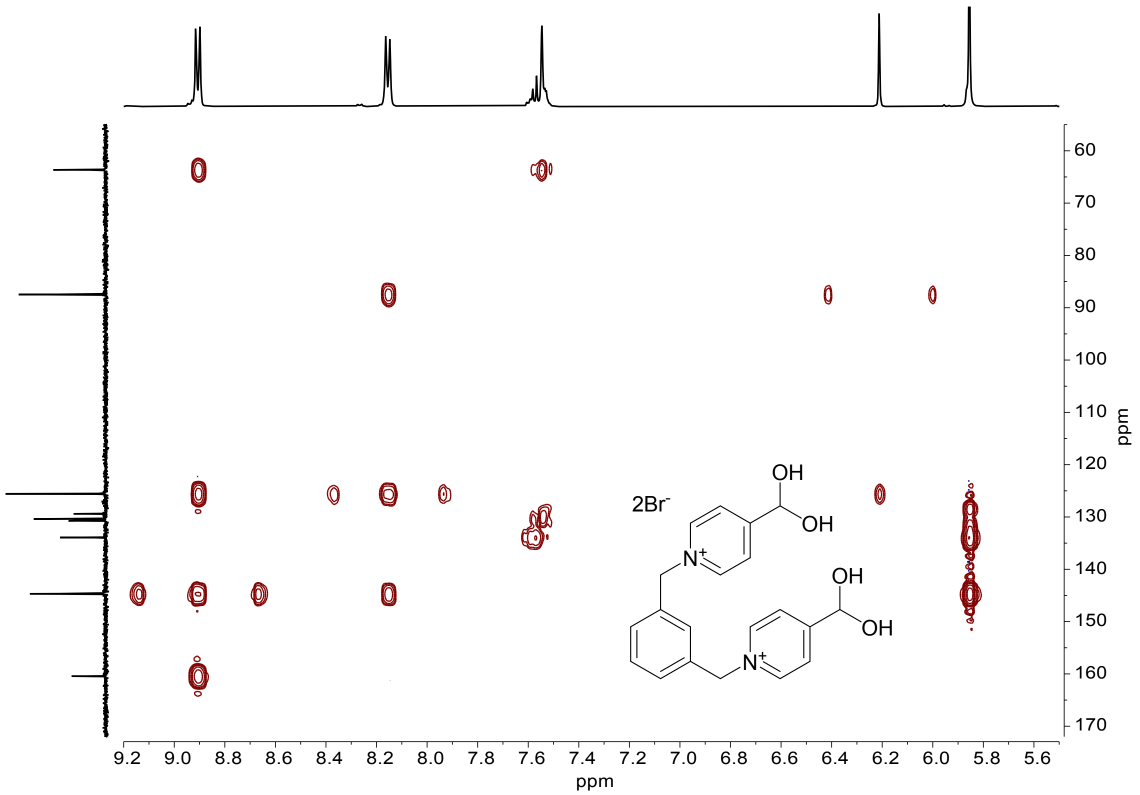

Figure S 6. ^{1}H-^{13}C HMBC (400 MHz/101 MHz, D_2O) spectrum of $\mathbf{A_b}$·2Br

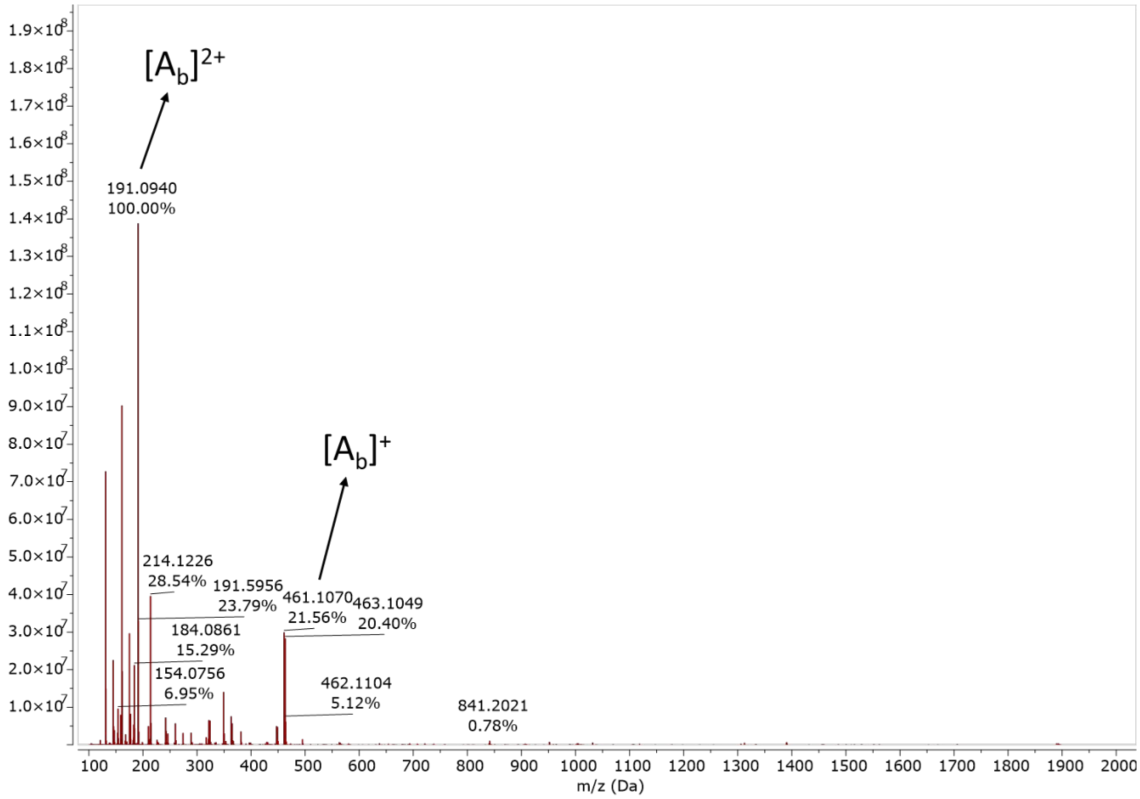

Figure S 7. HR ESI-MS spectrum of $\mathbf{A_b}$·2Br

1.2.2. **Synthesis and characterization data of A_c·2Br**

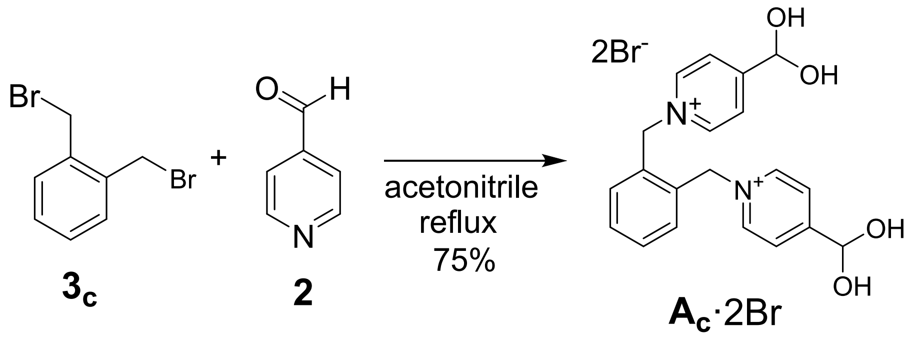

To a solution of 4-pyridinecarboxaldehyde (**2**, 1.71 mL, 18.0 mmol, 3 eq) in 180 mL of acetonitrile, 1,2-bis(bromomethyl)benzene (**3_c**, 1.58 g, 6 mmol, 1 eq) was added and the solution was heated at reflux in a magnetic hot plate stirrer for 18 h. The resulting precipitated is filtered, washed with acetonitrile (3×30 mL), diethyl ether (3×30 mL) and dried under vacuum, yielding **A_c**·2Br as a whitish solid (2.31 g, 75 %).

mp 240.7 – 251.9°C (decomposition).**^{1}H NMR** (500 MHz, D_2O), δ (ppm): 8.78 (d, J = 6.5 Hz, 4H), 8.11 (d, J = 6.4 Hz, 4H), 7.63 (dd, J = 7.5, 3.8 Hz, 2H), 7.39 (dd, J = 5.7, 3.4 Hz, 2H), 6.19 (s, 2H), 5.99 (s, 4H). **^{13}C{^{1}H} NMR** (126 MHz, D_2O), δ (ppm): 160.9 (C), 144.6 (CH), 131.5 (CH), 131.3 (CH), 130.9 (C), 125.7 (CH), 87.4 (CH), 60.8 (CH_2). **HRMS (ESI)** m/z: [**A_c**+2CH$_3$]$^{2+}$ Calcd for $C_{22}H_{26}N_2O_4^{2+}$ 191.0941; Found 191.0940.

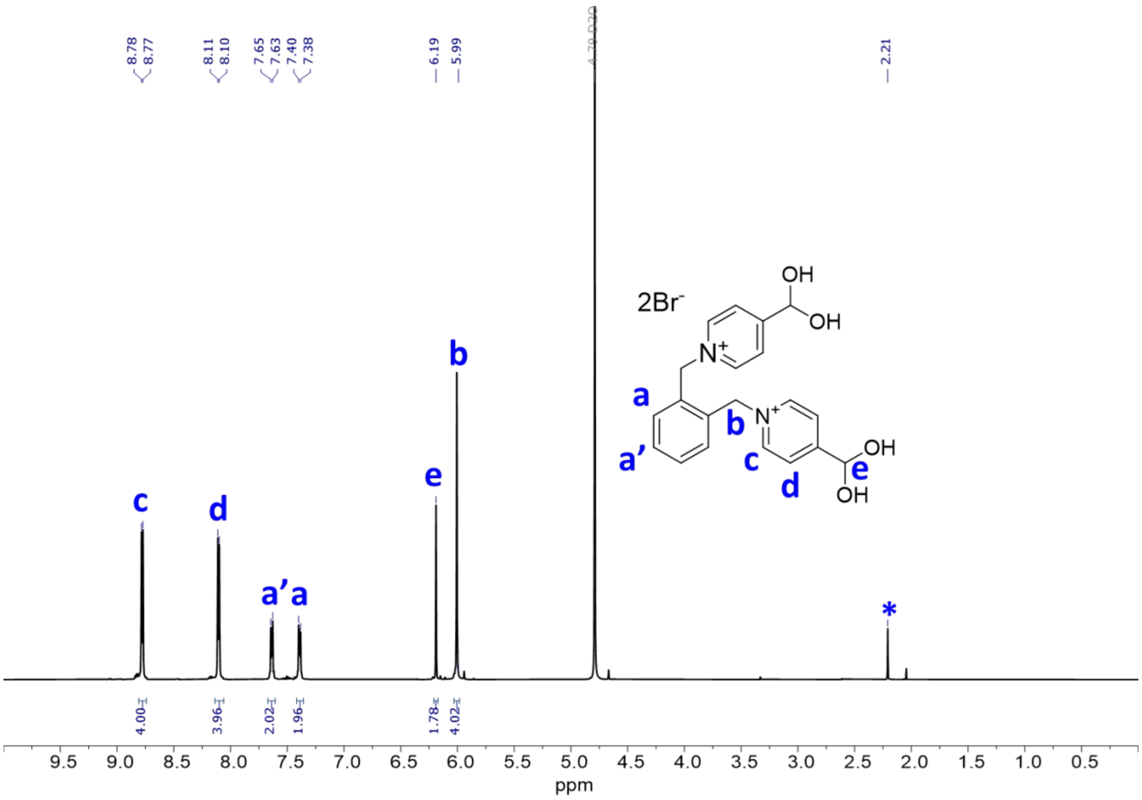

Figure S 8. ^{1}H NMR (500 MHz, D_2O) spectrum of **A_c**·2Br

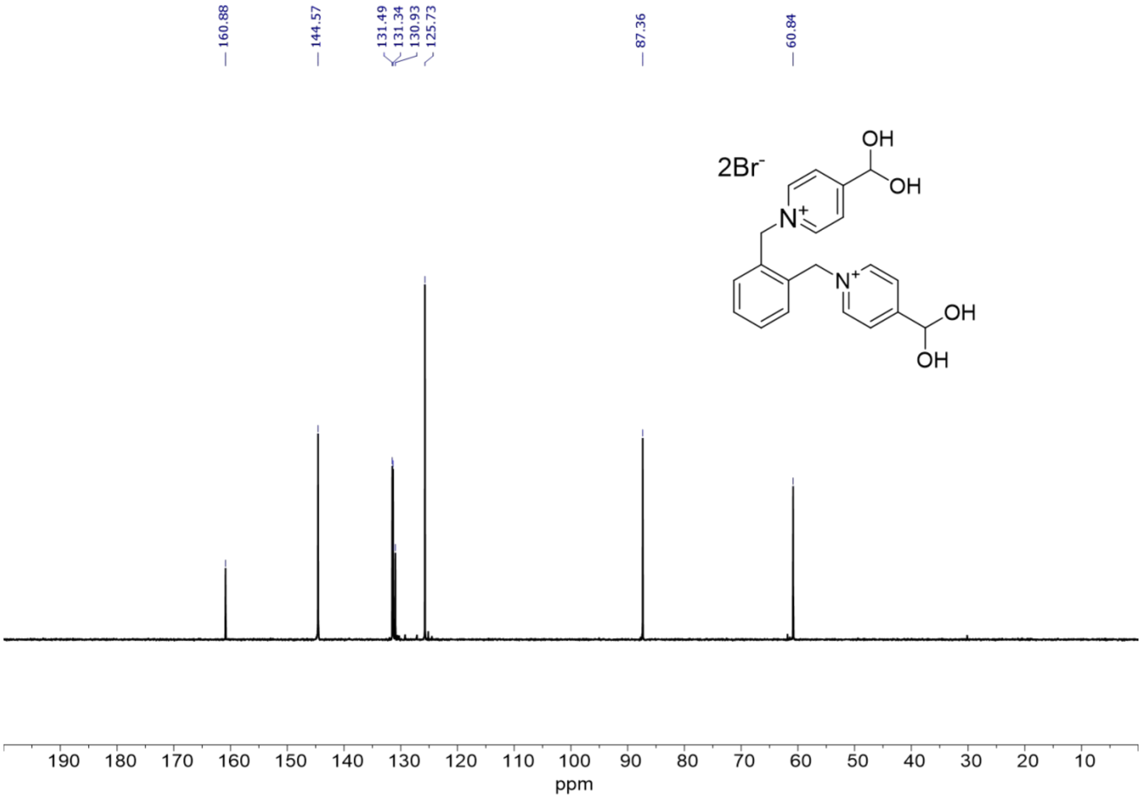

Figure S 9. $^{13}C\{1H\}$ NMR (126 MHz, D_2O) spectrum of of $\mathbf{A_c}$·2Br

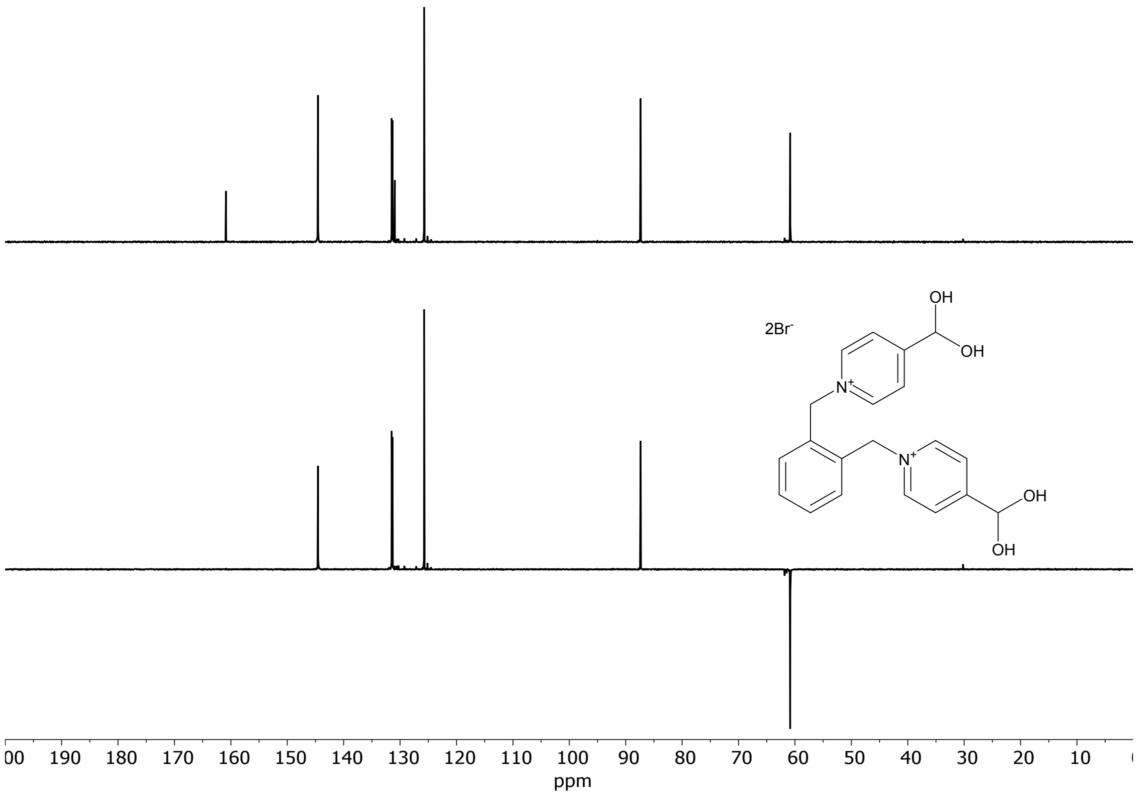

Figure S 10. $^{13}C\{1H\}$ NMR (126 MHz, D_2O) spectrum (top) and DEPT-135 (126 MHz, D_2O) spectrum of $\mathbf{A_c}$·2Br (bottom)

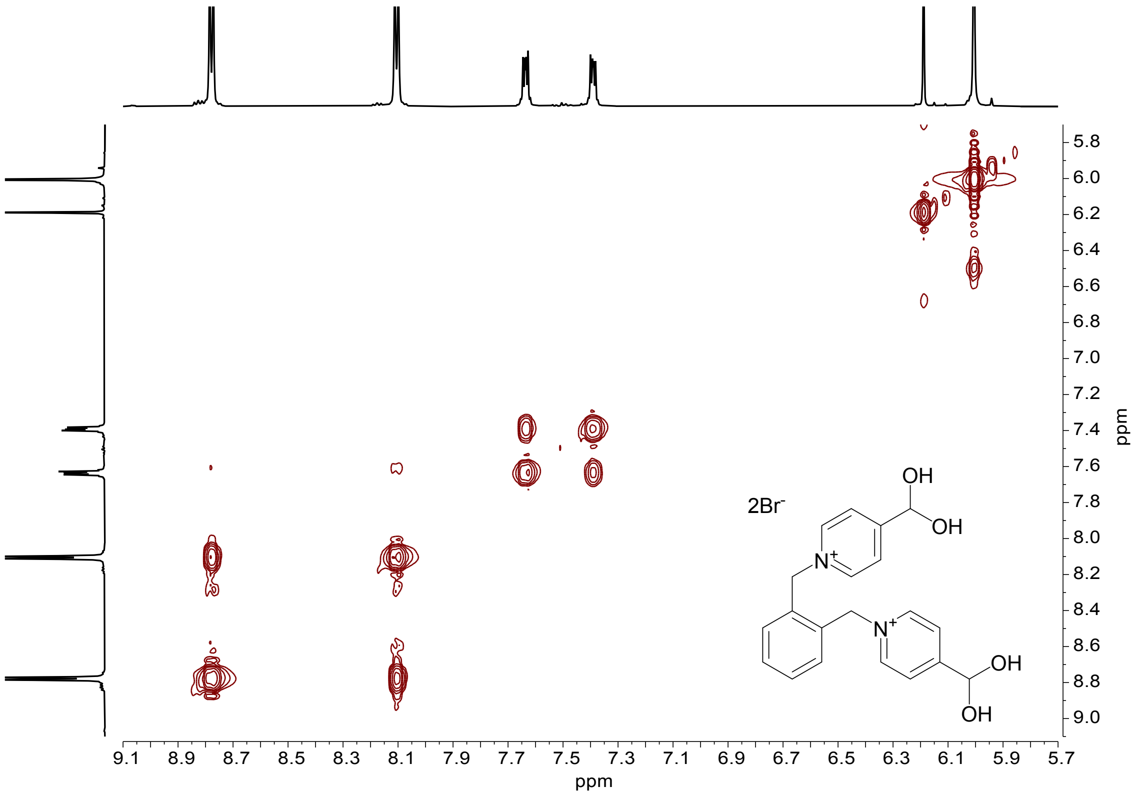

Figure S 11. ^{1}H-^{1}H COSY (500 MHz, D_2O) spectrum of $\mathbf{A_c}$·2Br

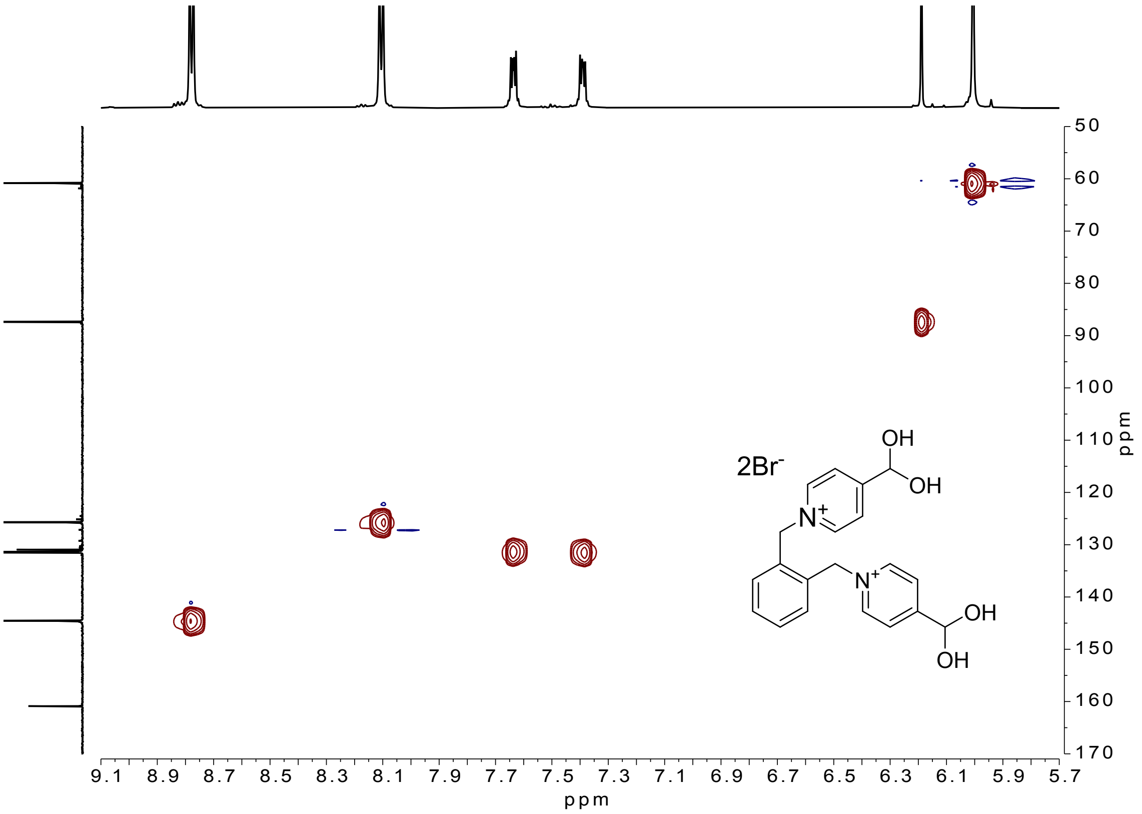

Figure S 12. ^{1}H-^{13}C HSQC (500 MHz, D_2O) spectrum of $\mathbf{A_c}$·2Br

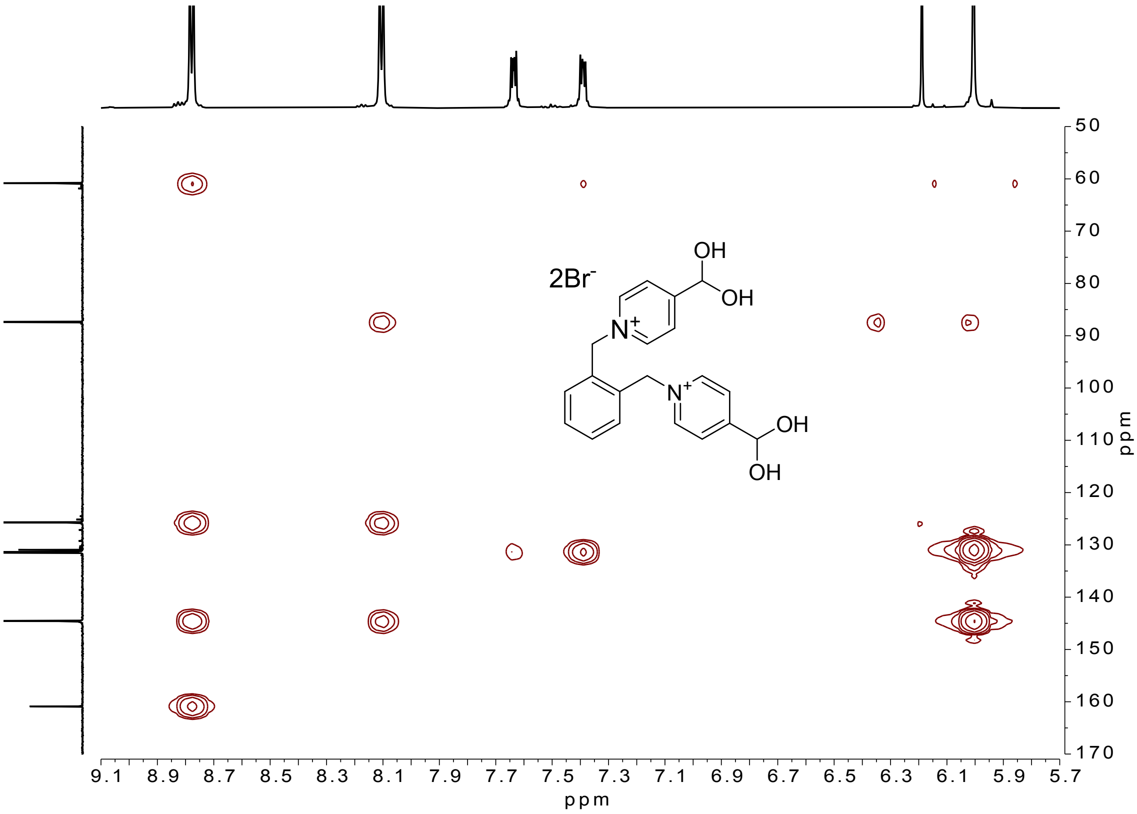

Figure S 13. ^{1}H-^{13}C HMBC (500 MHz, D_2O) spectrum of $\mathbf{A_c}$·2Br

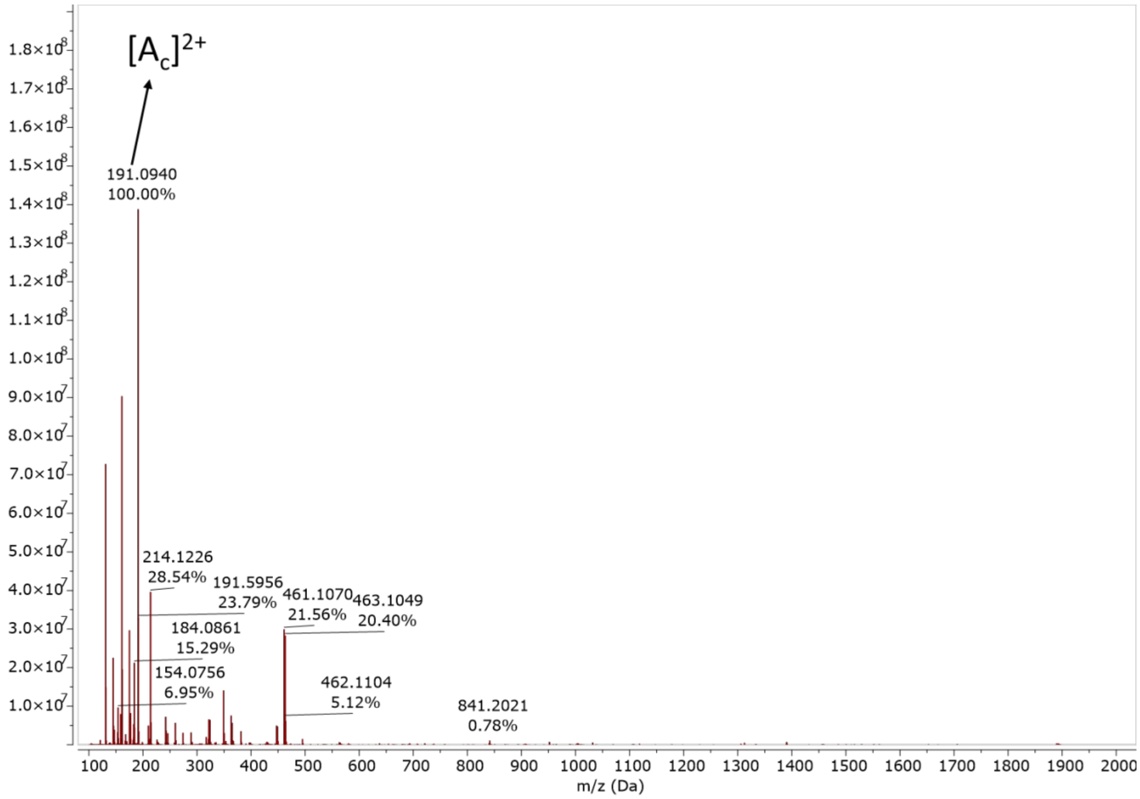

Figure S 14. HR ESI-MS spectrum of $\mathbf{A_c}$·2Br

1.2.3. **Synthesis and characterization data of A$_d$·2Br**

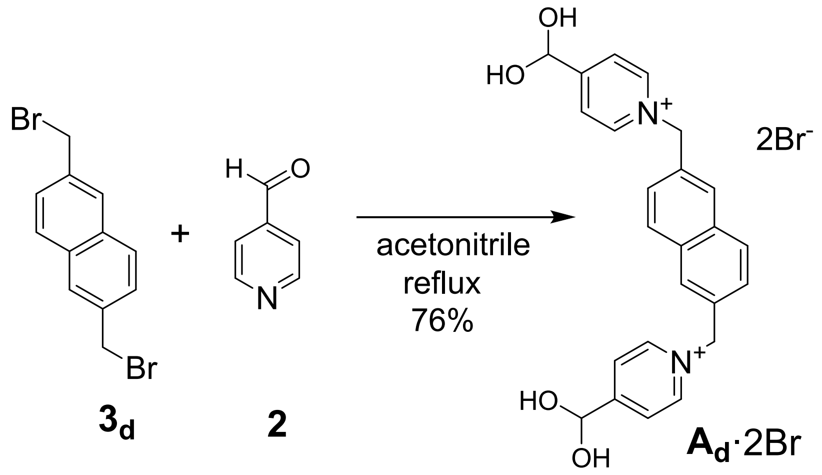

To a solution of 2,6-bis(bromomethyl)naphthalene (**3$_d$**, 2.67 g, 8.5 mmol, 1 eq) in 200 mL of acetonitrile, 4-Pyridinecarboxaldehyde (**2**, 2.40 mL, 25.5 mmol, 3 eq.) was added. The resulting mixture was heated at reflux in a magnetic hot plate stirrer for 24 h. The resulting precipitate is filtered, washed with acetonitrile (3×30 mL), diethyl ether (3×30 mL) and dried under vacuum, yielding **A$_d$**·2Br as a reddish solid (3.64 g, 76%).

mp 243.5 – 245.4°C (decomposition). **^{1}H NMR** (500 MHz, D_2O), δ (ppm): 8.98 (d, J = 6.5 Hz, 4H), 8.14 (d, J = 6.3 Hz, 4H), 8.03 (s, 2H), 8.03 (d, 4H), 7.58 (d, 2H), 6.19 (s, 2H), 5.99 (s, 4H). **^{13}C{^{1}H} NMR** (126 MHz, D_2O), δ (ppm): 160.3 (C), 144.7 (CH), 142.2 (C), 133.04 (C), 131.6 (C), 129.7 (CH), 128.8 (CH), 126.5 (CH), 125.53 (CH), 87.5 (CH), 64.2 (CH_2). **HRMS** (**ESI**) m/z: [**A$_d$**]$^+$ Calcd for $C_{24}H_{23}N_2O_4^+$ 403.1653; Found, 403.1656; [**A$_d$**-H$^+$+CH$_3$]$^+$ Calcd for $C_{25}H_{25}N_2O_4^+$ 417.1809; Found 417.1813; [**A$_d$**+Br]$^+$ Calcd for $C_{24}H_{24}BrN_2O_4^+$ 483.0914; Found, 483.0918; and [**A$_d$**+Br+CH$_3$]$^+$ Calcd for $C_{25}H_{26}BrN_2O_4^+$ 497.1071; Found 497.1075.

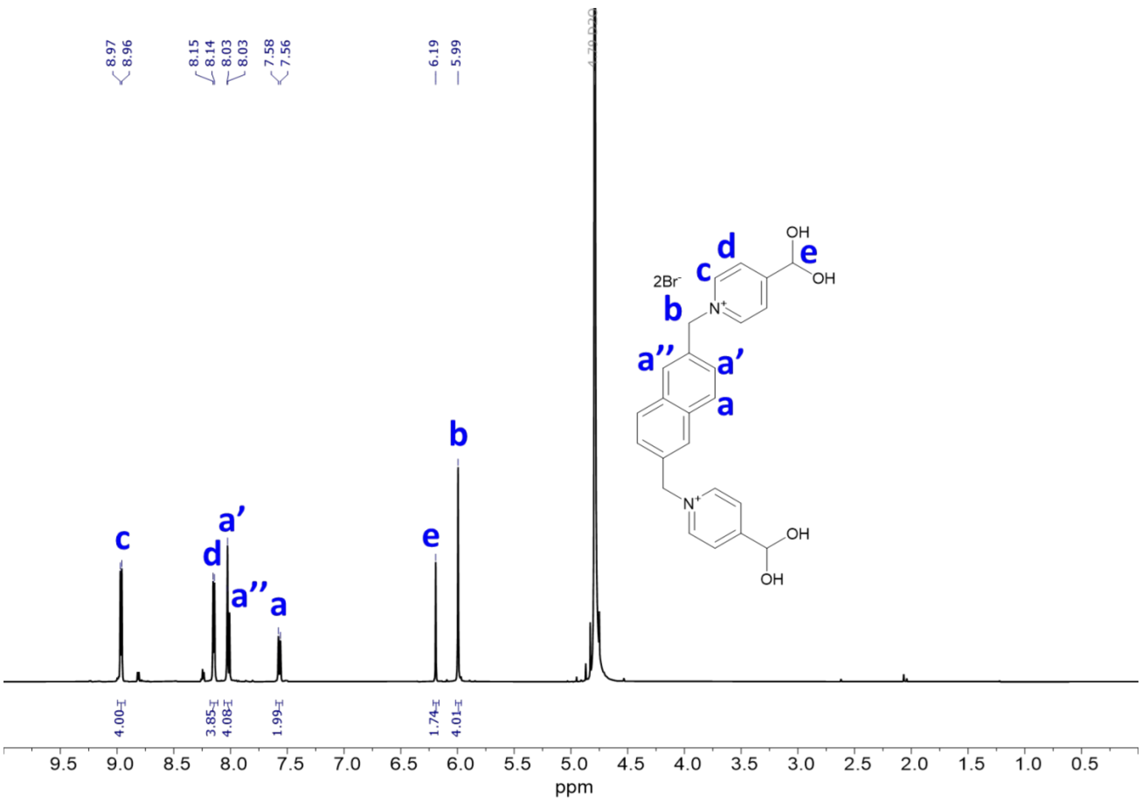

Figure S 15. ^{1}H NMR (500 MHz, D_2O) spectrum of $\mathbf{A_d}$·2Br

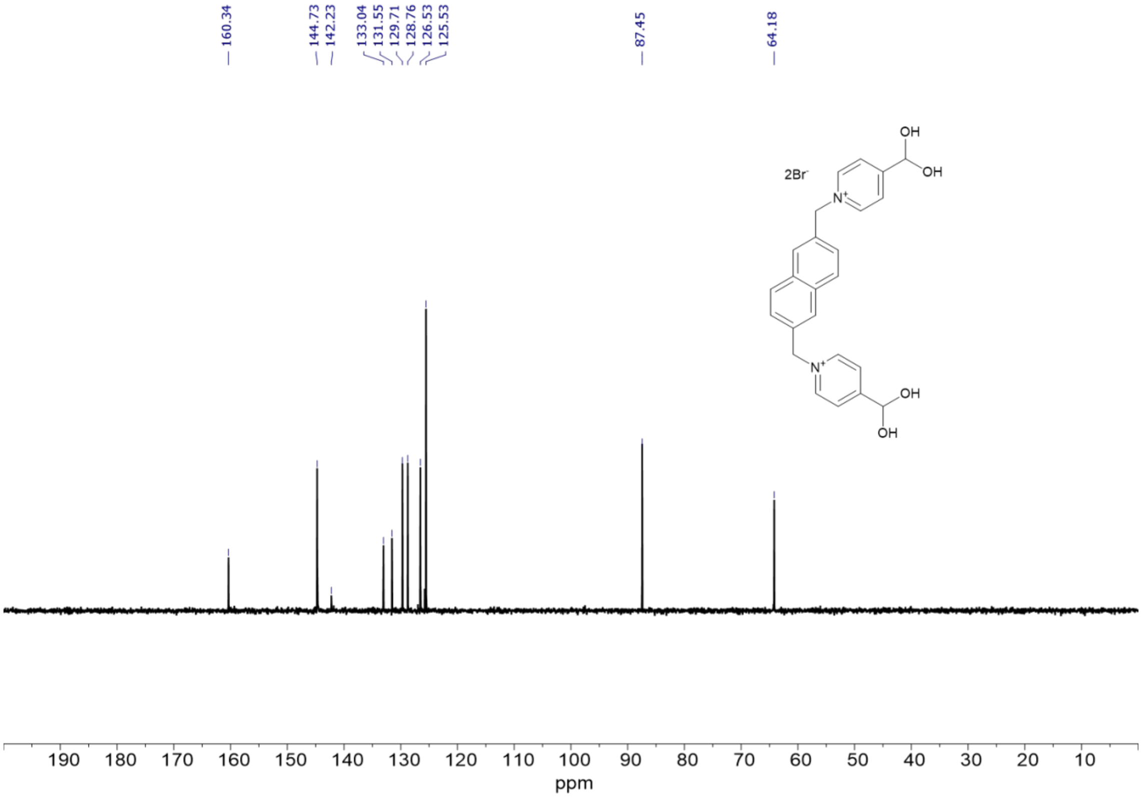

Figure S 16. $^{13}C\{^{1}H\}$ NMR (126 MHz, D_2O) spectrum of of $\mathbf{A_d}$·2Br

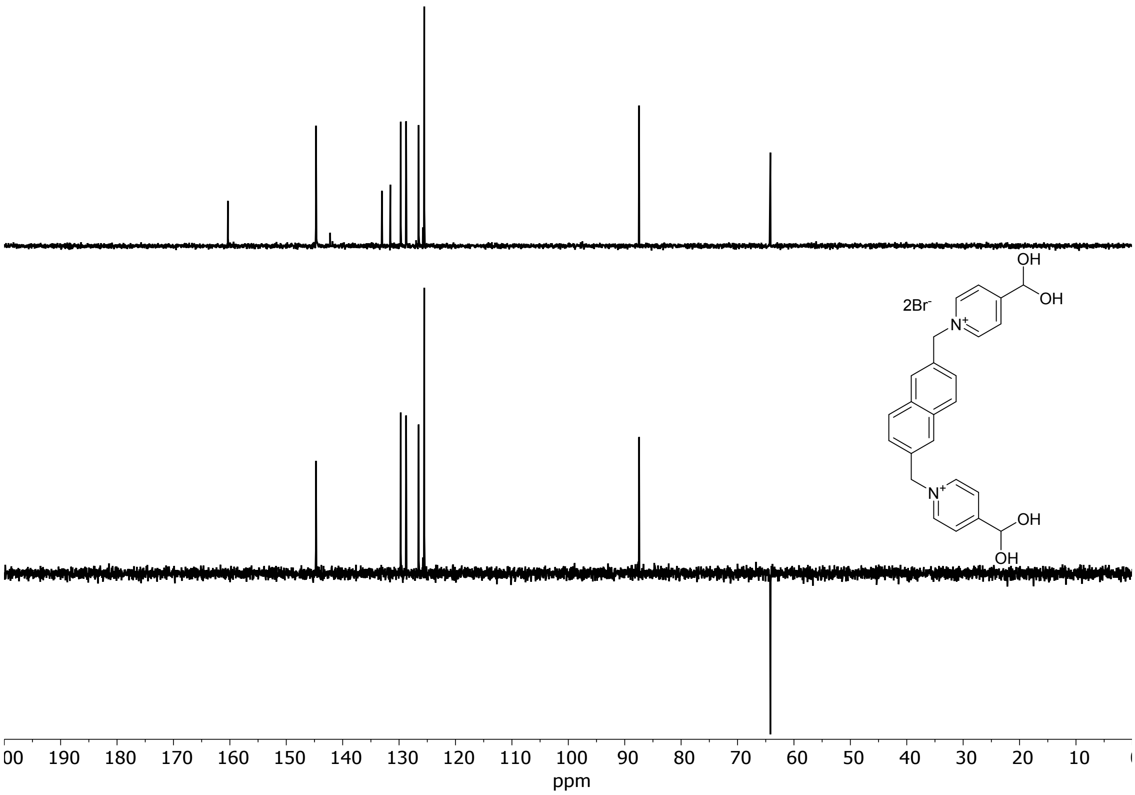

Figure S 17. $^{13}C\{^{1}H\}$ NMR (126 MHz, D_2O) spectrum (top) and DEPT-135 (126 MHz, D_2O) spectrum (bottom) of $\mathbf{A_d}$·2Br

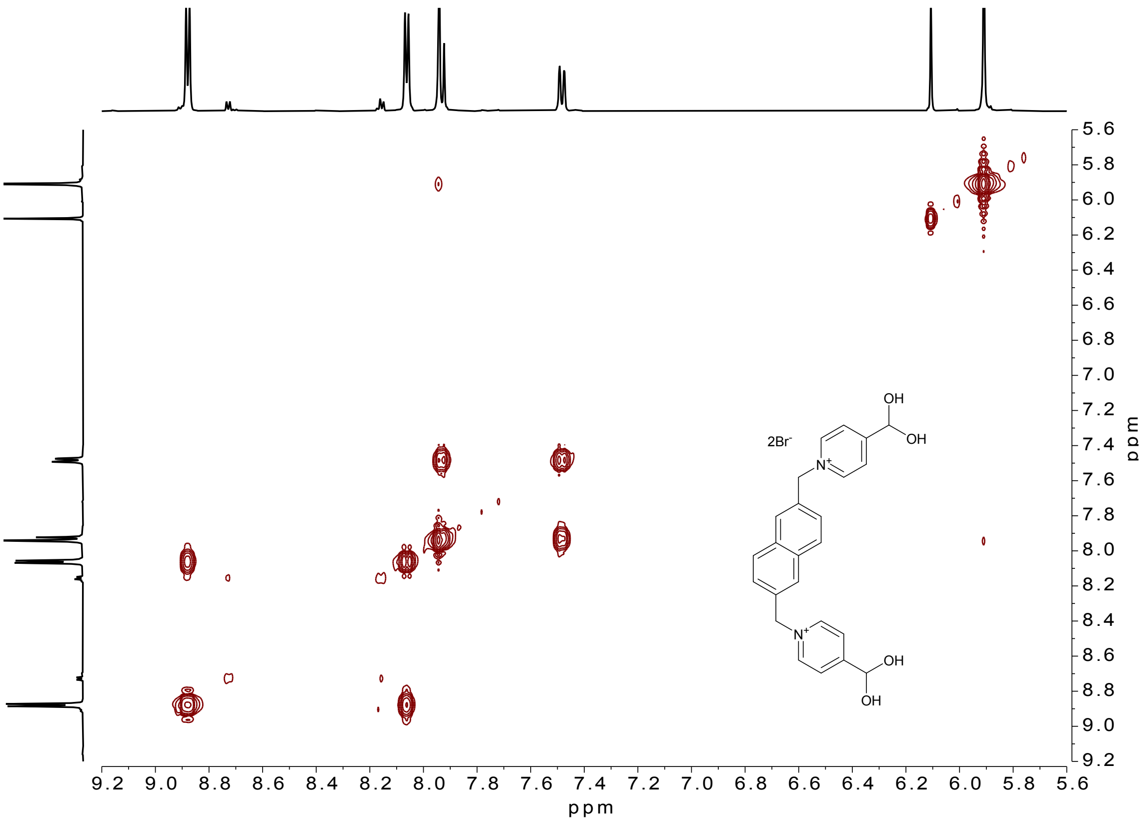

Figure S 18. ^{1}H-^{1}H COSY (500 MHz, D_2O) spectrum of $\mathbf{A_d}$·2Br

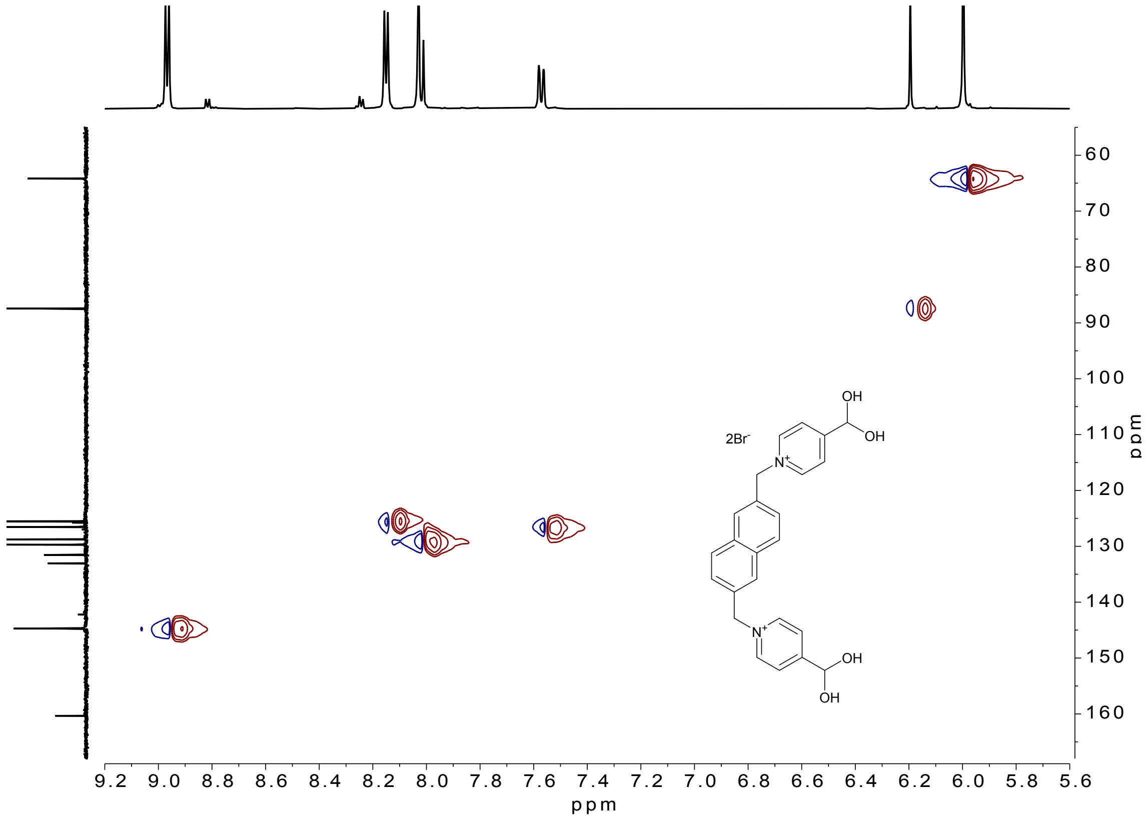

Figure S 19. ^{1}H-^{13}C HSQC (500 MHz/126 MHz, D_2O) spectrum of $\mathbf{A_d}$·2Br

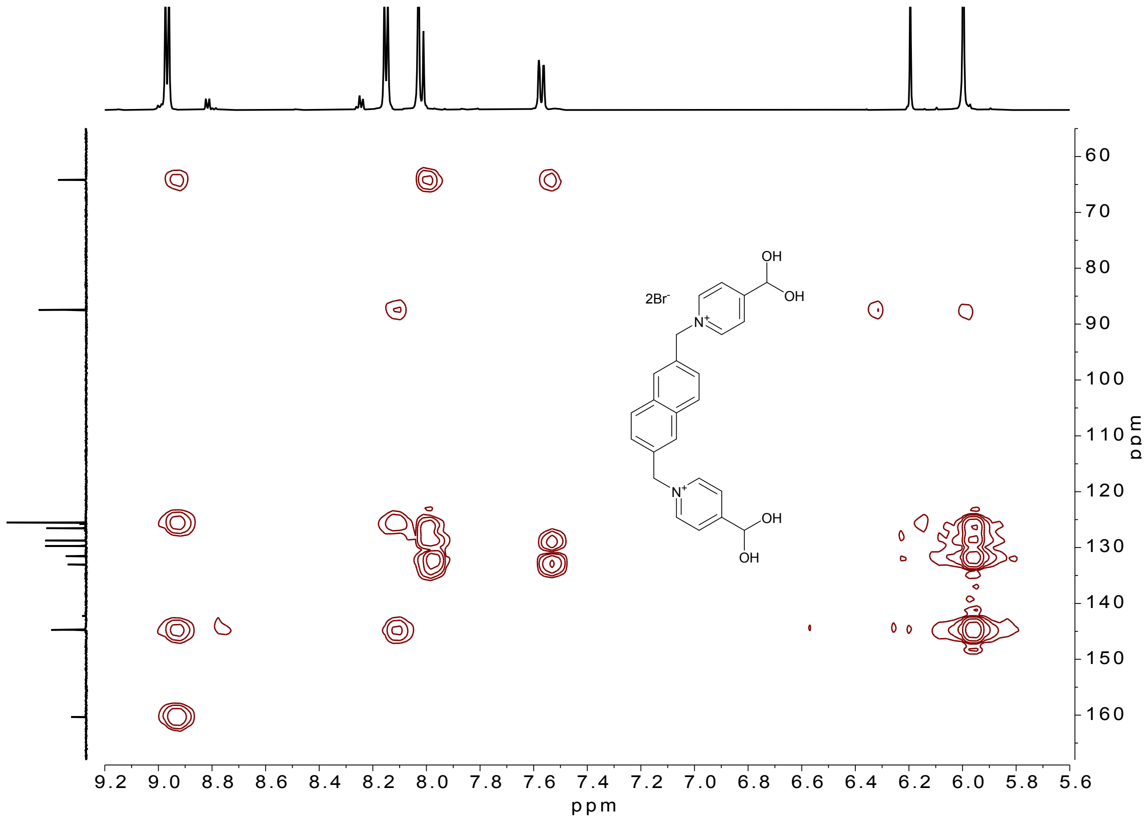

Figure S 20. ^{1}H-^{13}C HMBC (500 MHz/126 MHz, D_2O) spectrum of $\mathbf{A_d}$·2Br

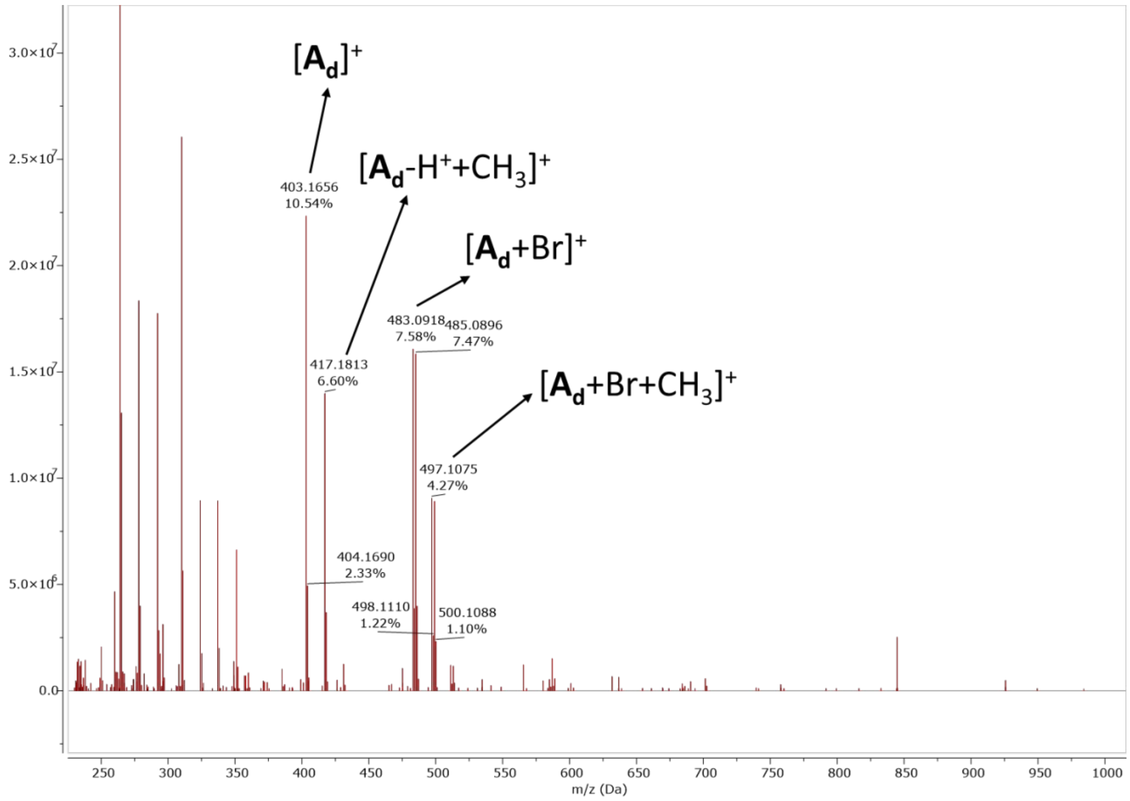

Figure S 21. HR ESI-MS spectrum of $\mathbf{A_d}$·2Br

1.2.4. **Synthesis and characterization data of A_e·2Br**

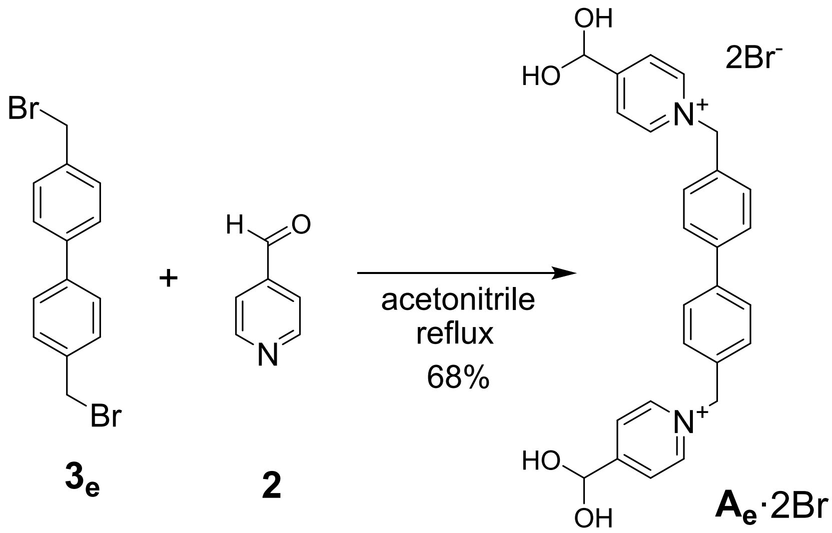

4,4'-bis(bromomethyl)biphenyl (**3**$_e$, 2.89 g, 8.5 mmol, 1 eq) and 4-pyridinecarboxaldehyde (**2**, 2.4 mL, 525.5 mmol, 3 eq) were dissolved in 200 mL of acetonitrile and the resulting solution was heated at reflux in a magnetic hot plate stirrer for 24 h. The resulting precipitate was vacuum filtered and washed with acetonitrile (3×30 mL) and ethyl acetate (3x30 mL), yielding a dark solid **A**$_e$·2Br (3.41 g, 68%).

mp 245.0 – 246.9°C (decomposition).**^{1}H NMR** (500 MHz, D_2O), δ (ppm): 8.84 (d, J = 6.4 Hz, 4H), 8.06 (d, J = 6.4 Hz, 4H), 7.69 (d, J = 2.0 Hz, 4H), 7.47 (d, J = 8.2 Hz, 4H), 6.11 (s, 2H), 5.78 (s, 4H). **^{13}C{^{1}H} NMR** (126 MHz, D_2O) δ (ppm): 160.3 (C), 144.7 (CH), 141.0 (C), 132.3 (C), 129.7 (CH), 128.0 (CH), 125.50 (CH), 87.5 (CH), 63.8 (CH_2). **HRMS** (**ESI**) m/z: [**A**$_e$]$^+$ Calcd for $C_{26}H_{25}N_2O_4^+$ 429.1809; Found, 429.1804; [**A**$_e$-H$^+$+CH$_3$]$^+$ Calcd for $C_{27}H_{27}N_2O_4^+$ 443.1966; Found 443.1960; [**A**$_e$+Br]$^+$ Calcd for $C_{26}H_{26}BrN_2O_4^+$ 511.1050; Found, 511.1042; and [**A**$_e$+Br+CH$_3$]$^+$ Calcd for $C_{27}H_{28}BrN_2O_4^+$523.1227; Found 523.12181075.

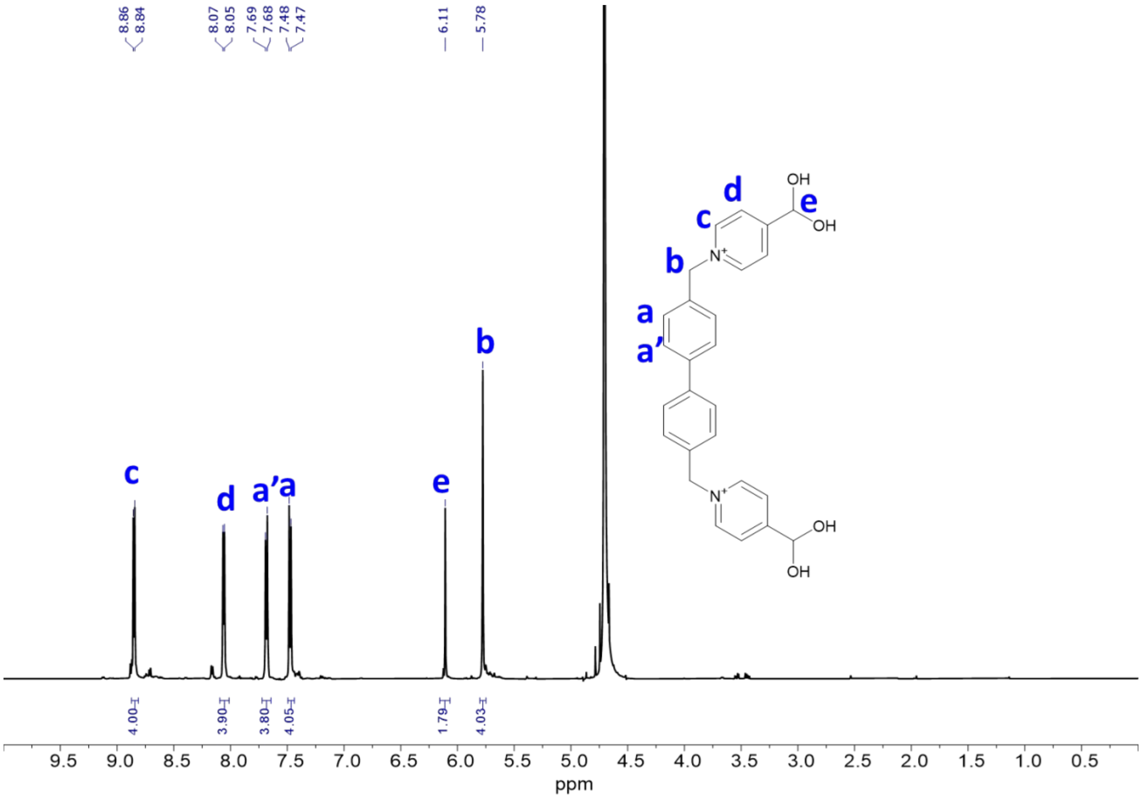

Figure S 22. ^{1}H NMR (500 MHz, D_2O) spectrum of $\mathbf{A_e}$·2Br

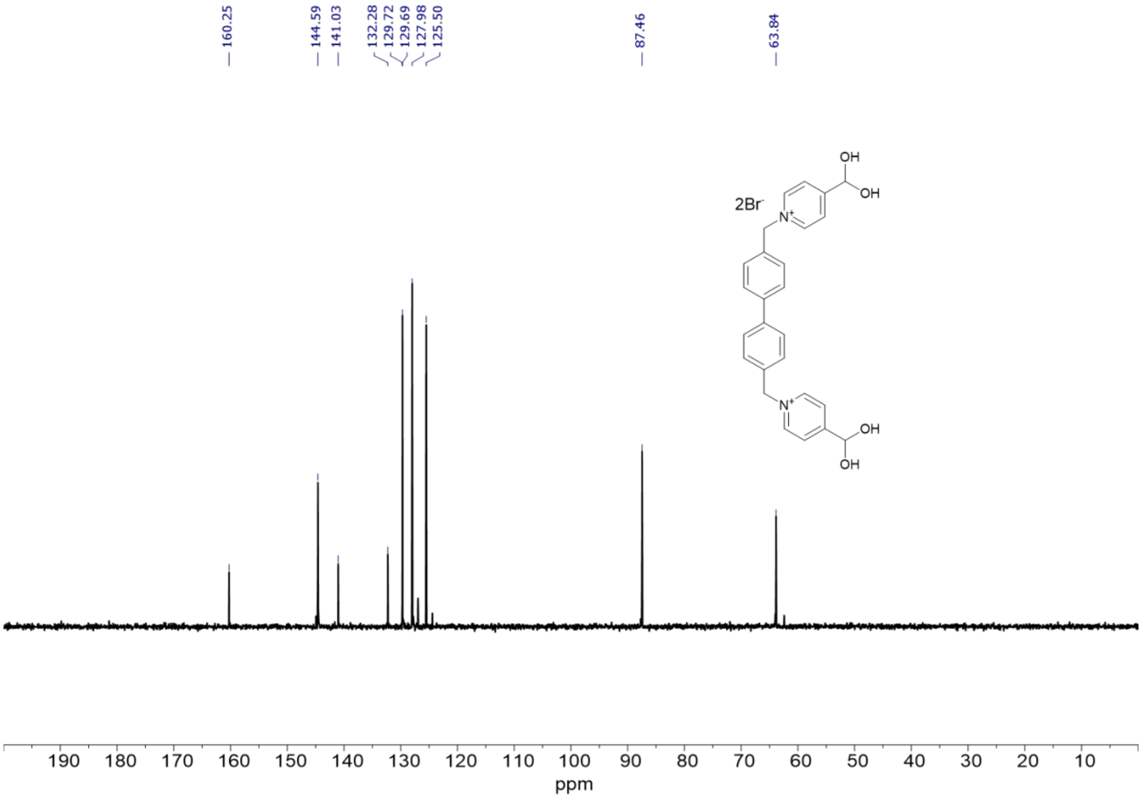

Figure S 23. $^{13}C\{^{1}H\}$ NMR (126 MHz, D_2O) spectrum of of $\mathbf{A_e}$·2Br

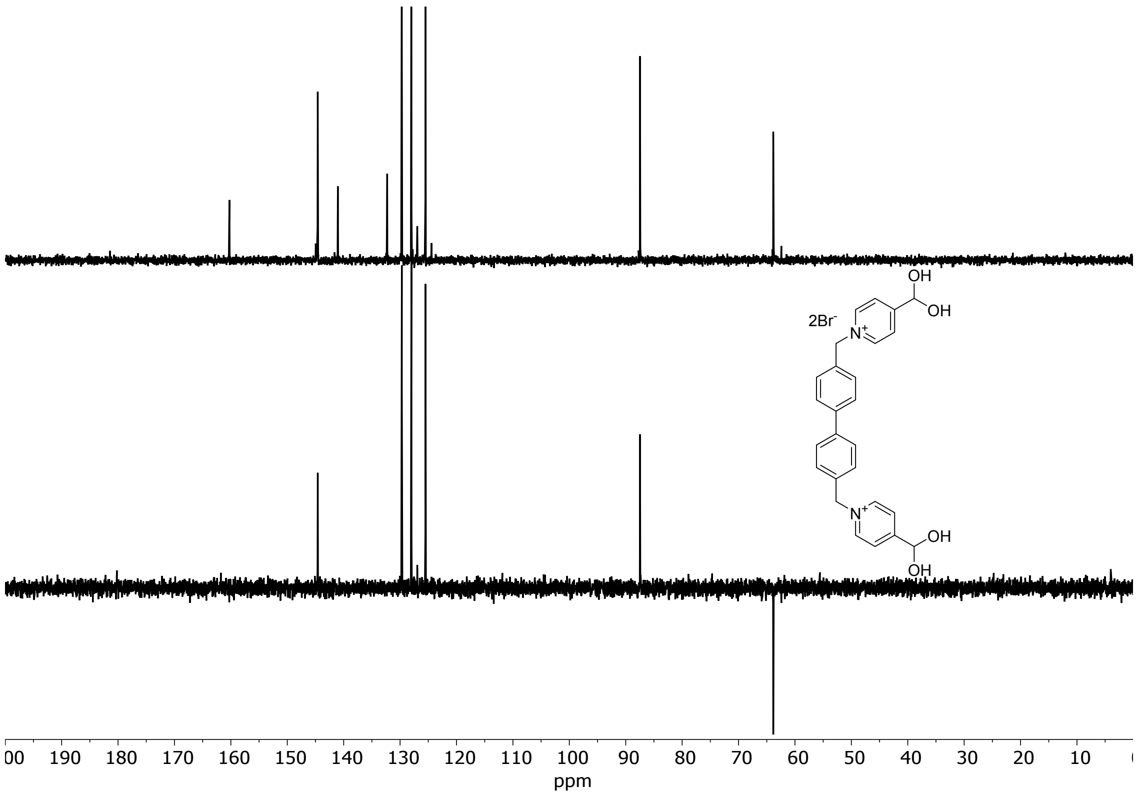

Figure S 24. $^{13}C\{^{1}H\}$ NMR (126 MHz, D_2O) spectrum (top) and DEPT-135 (126 MHz, D_2O) spectrum (bottom) of $\mathbf{A_e}$·2Br

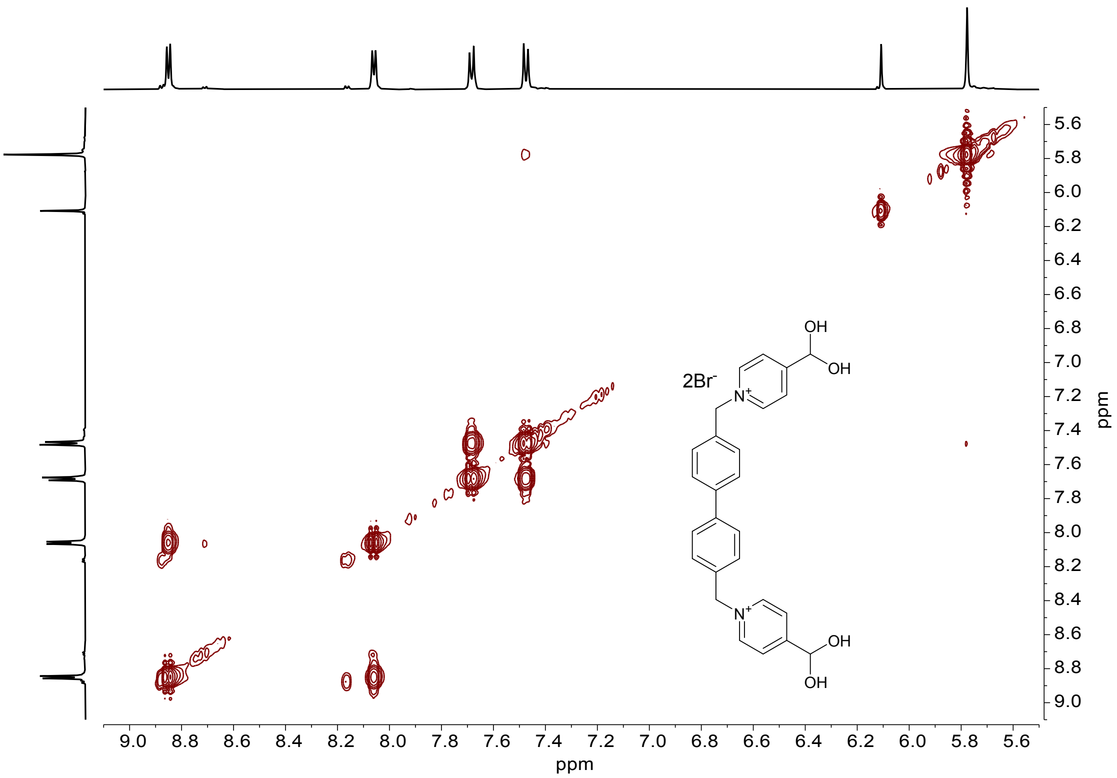

Figure S 25. ^{1}H-^{1}H COSY (500 MHz, D_2O) spectrum of $\mathbf{A_e}$·2Br

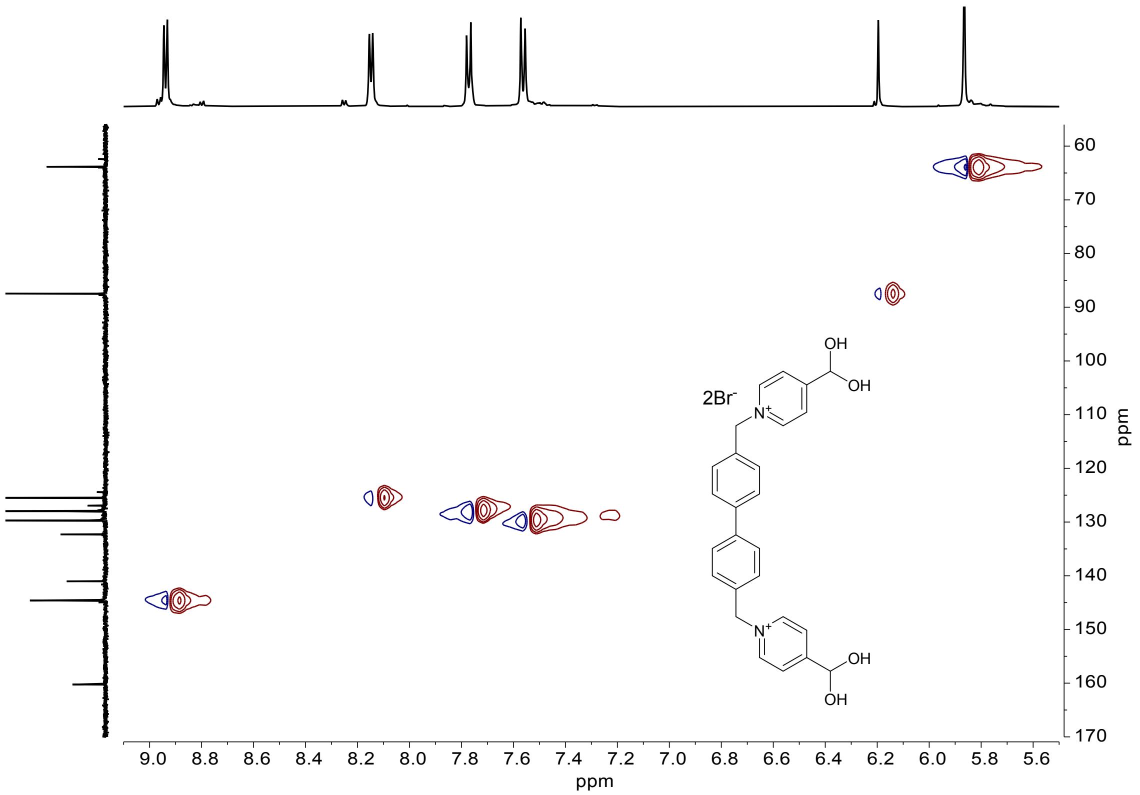

Figure S 26. ^{1}H-^{13}C HSQC (500 MHz, D_2O) spectrum of $\mathbf{A_e}$·2Br

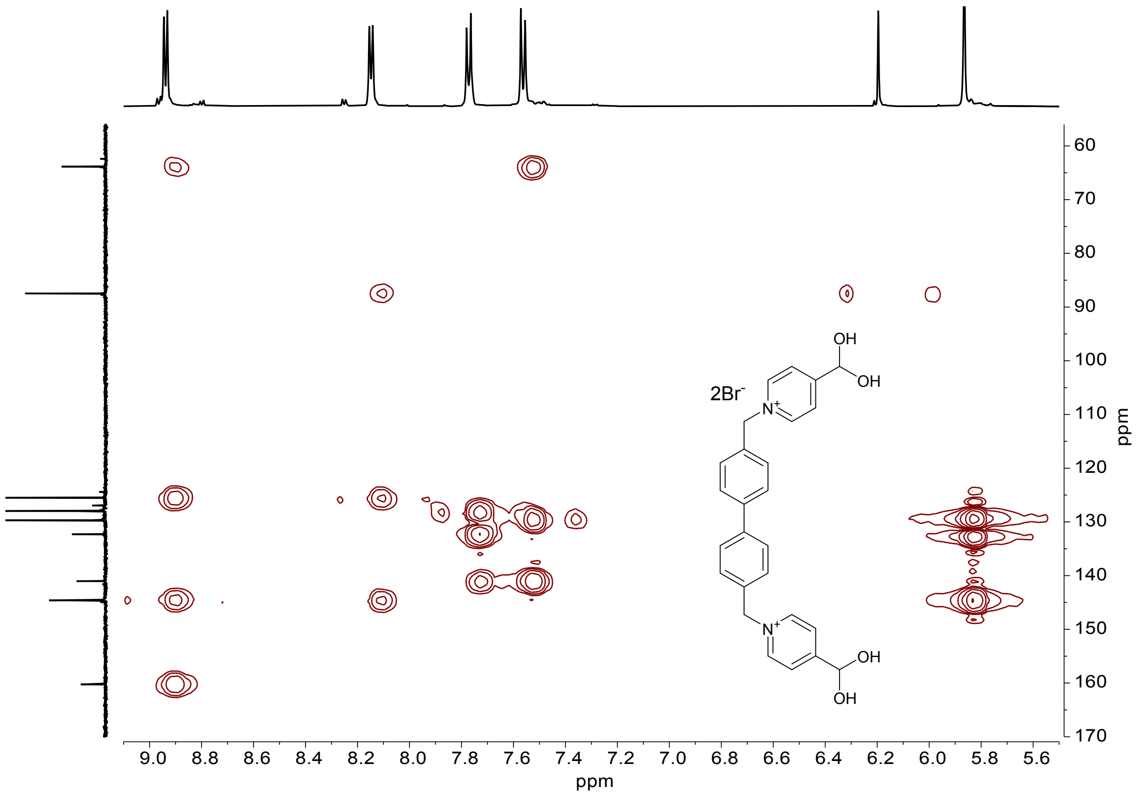

Figure S 27. ^{1}H-^{13}C HMBC (500 MHz, D_2O) spectrum of $\mathbf{A_e}$·2Br

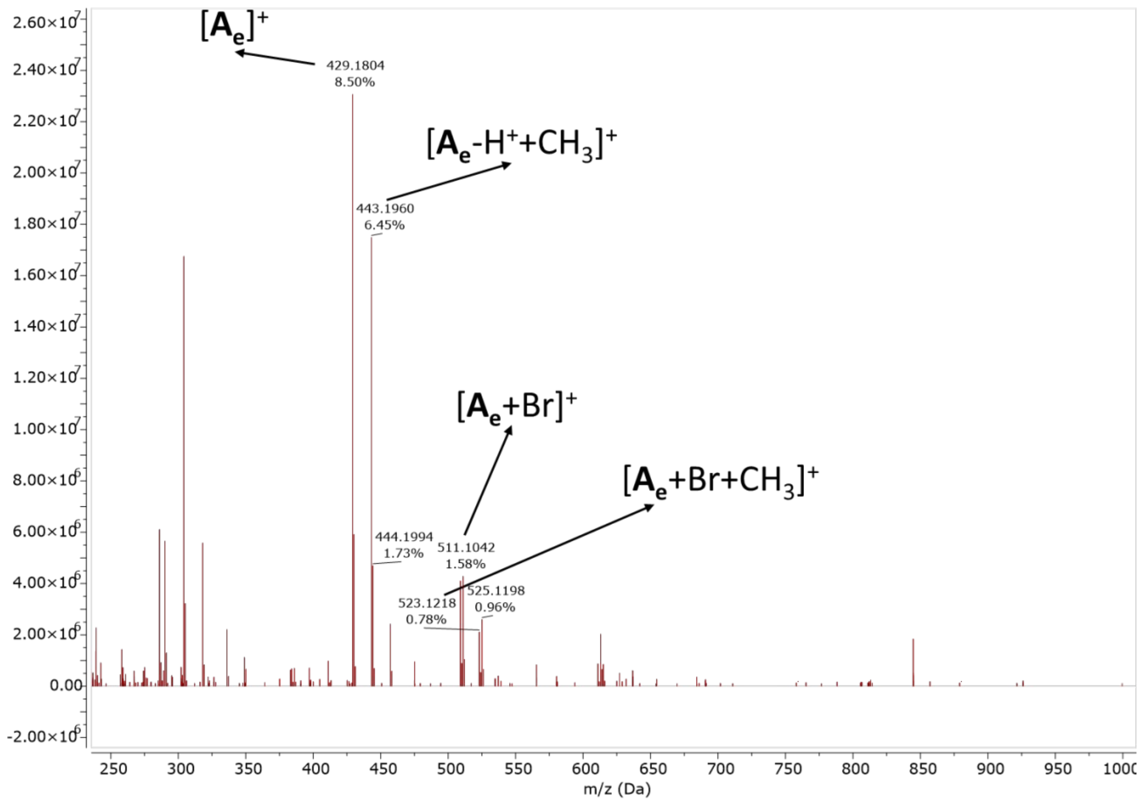

Figure S 28. HR ESI-MS spectrum of $\mathbf{A_e}$·2Br

1.2.5. **Synthesis and characterization data of H_b·2Br**

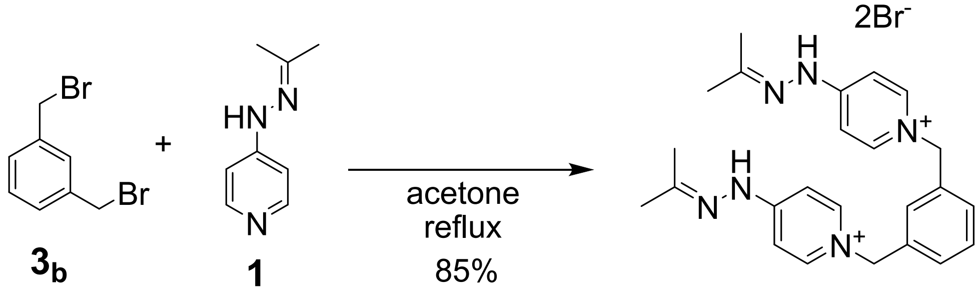

3_b **1** **H_b**·2Br

4-(2-(propan-2-ylidene)hydrazineyl)pyridine (**1**, 2.69 g, 18.0 mmol, 3 eq) is dissolved in 150 mL of acetone, and *α*,*α*'-dibromo-m-xylene (**3_b**, 1.58 g, 6.0 mmol, 1 eq) is added. The solution is heated at reflux in a magnetic hot plate stirrer for 20 h. Upon cooling, the resulting precipitated is filtered, washed with acetone (3×30 mL), diethyl ether (3×30 mL) and dried under vacuum, to yield **H_b**·2Br as a whitish powdery solid (2.87 g, 85%).

mp 249.7 – 252.0°C (decomposition). **^{1}H NMR** (400 MHz, D_2O) δ (ppm): 8.15 (s ancho, 4H), 7.55 (dt, J = 7.7, 5.8 Hz, 1H), 7.45 (dd, J = 7.8, 1.8 Hz, 2H), 7.36 (s ancho, 2H), 7.15 (s, 1H), 7.06 (s ancho, 2H), 5.43 (s, 4H), 2.15 (s, 6H), 2.08 (s, 6H). **^{13}C{^{1}H} NMR** (101 MHz, D_2O) δ (ppm): 162.9(C), 154.3 (C), 135.7 (C), 130.2 (CH), 128.7 (CH), 126.9 (CH), 60.7 (CH), 24.4 (CH_3), 17.4 (CH_3). **HRMS** (**ESI**) m/z: [**H_b**]$^{2+}$ Calcd for $C_{24}H_{30}N_6^{2+}$ 201.1261; Found 201.1260; and [**H_b**]$^+$ Calcd for $C_{24}H_{29}N_6^+$ 401.2499; Found 401.2449.

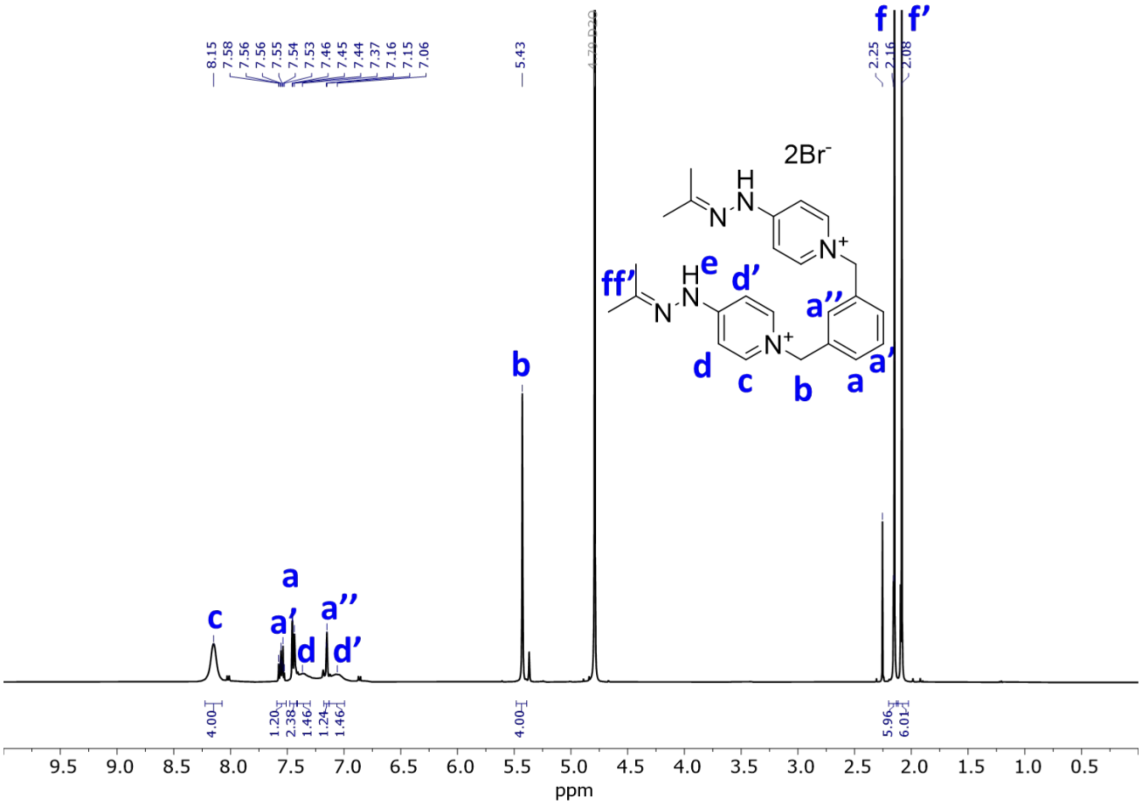

Figure S 29. ^{1}H NMR (500 MHz, D_2O) spectrum of $\mathbf{H_b}$·2Br

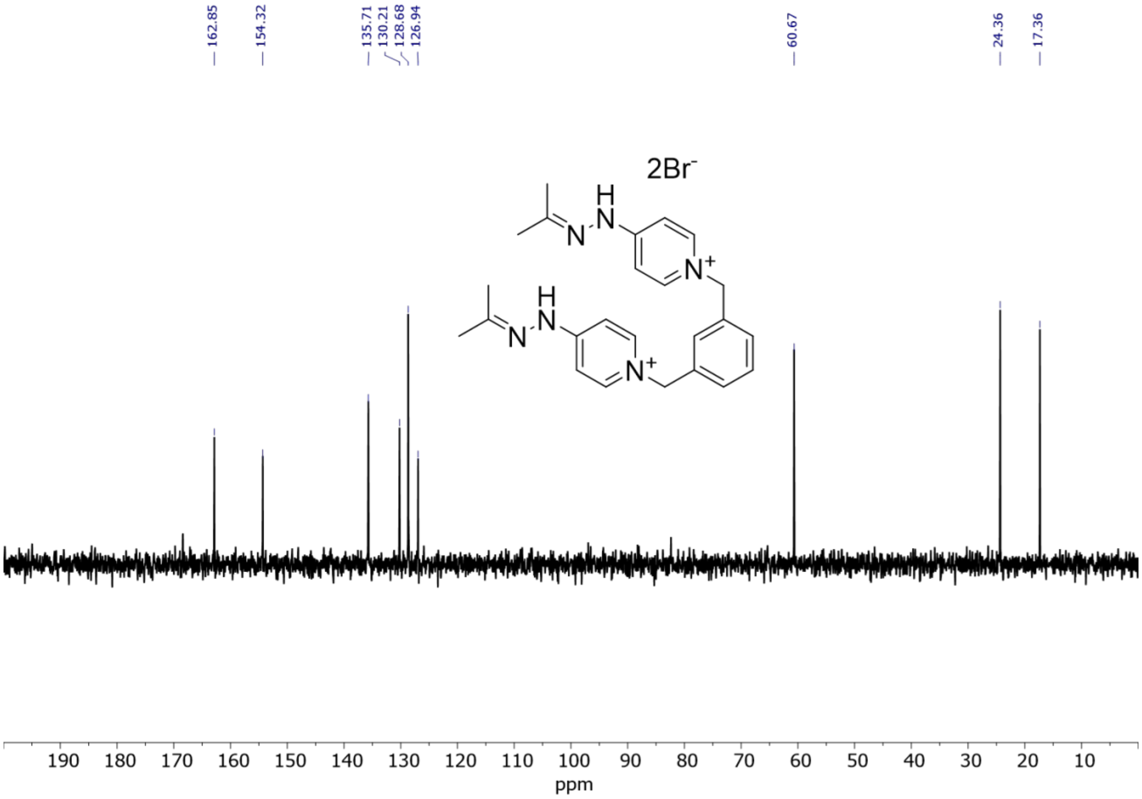

Figure S 30. $^{13}C\{^{1}H\}$ NMR (101 MHz, D_2O) spectrum of of $\mathbf{H_b}$·2Br

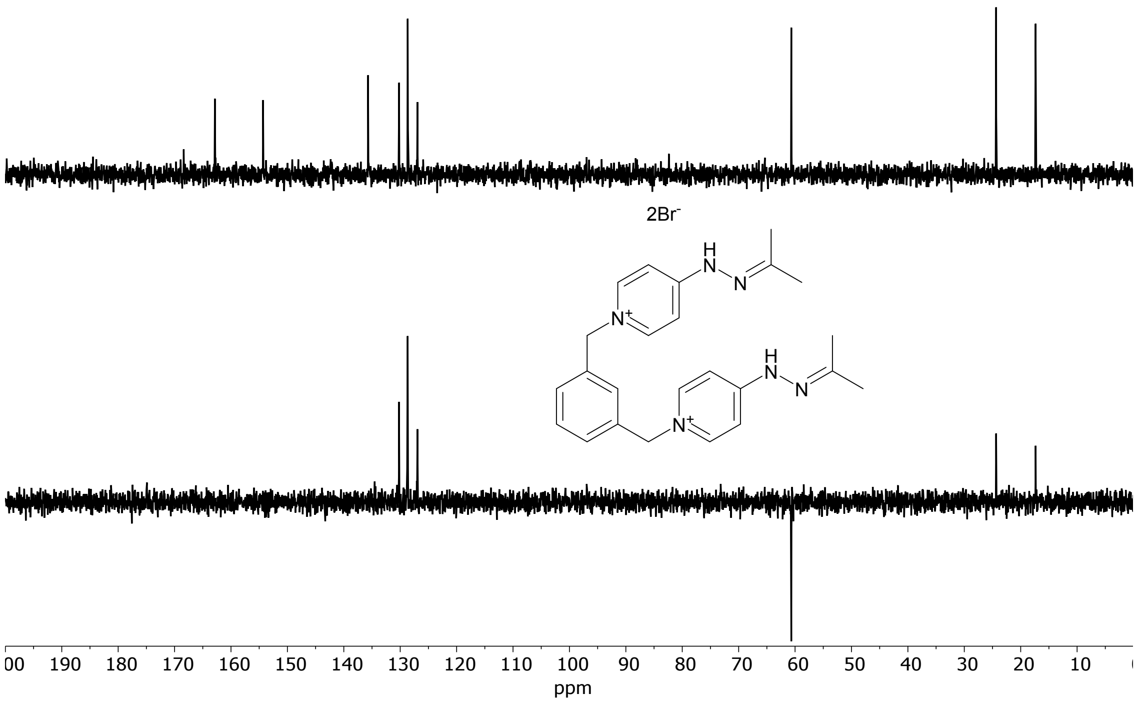

Figure S 31. $^{13}C\{^{1}H\}$ NMR (101 MHz, D_2O) spectrum (top) and DEPT-135 (101 MHz, D_2O) spectrum (bottom) of $\mathbf{H_b}$·2Br

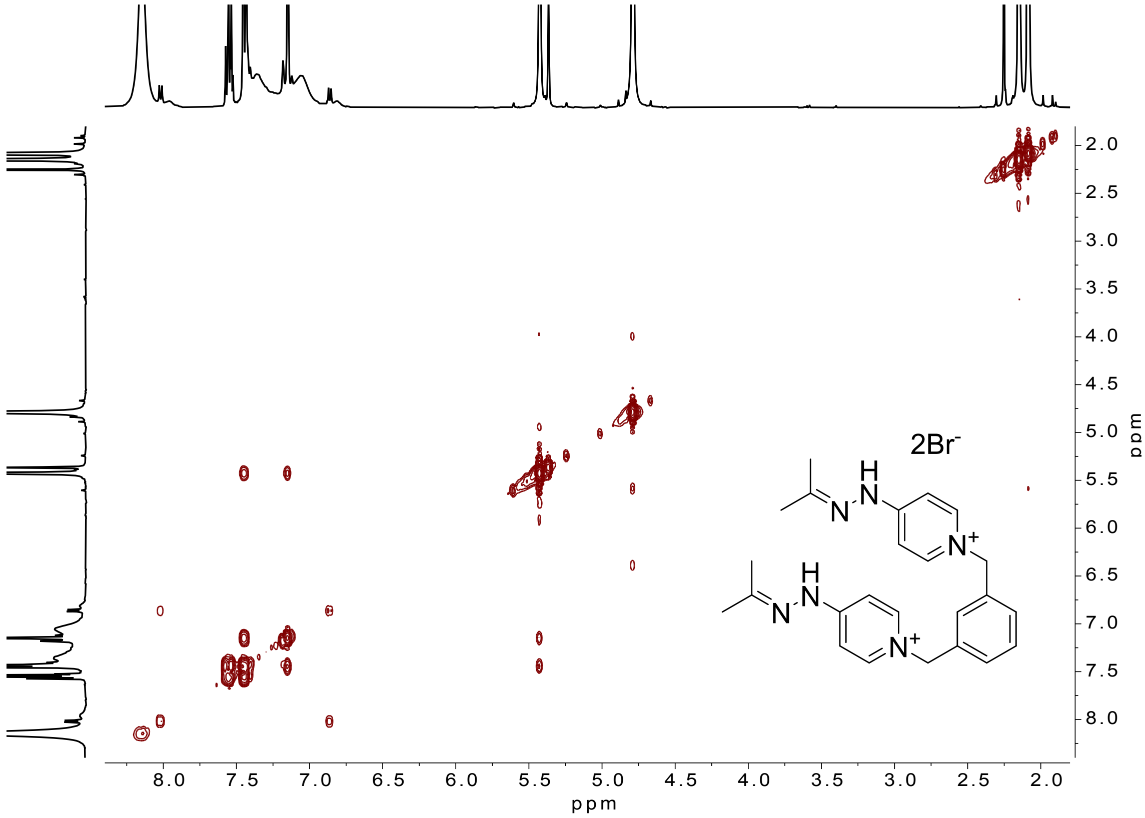

Figure S 32. ^{1}H-^{1}H COSY NMR (500 MHz, D_2O) spectrum of $\mathbf{H_b}$·2Br

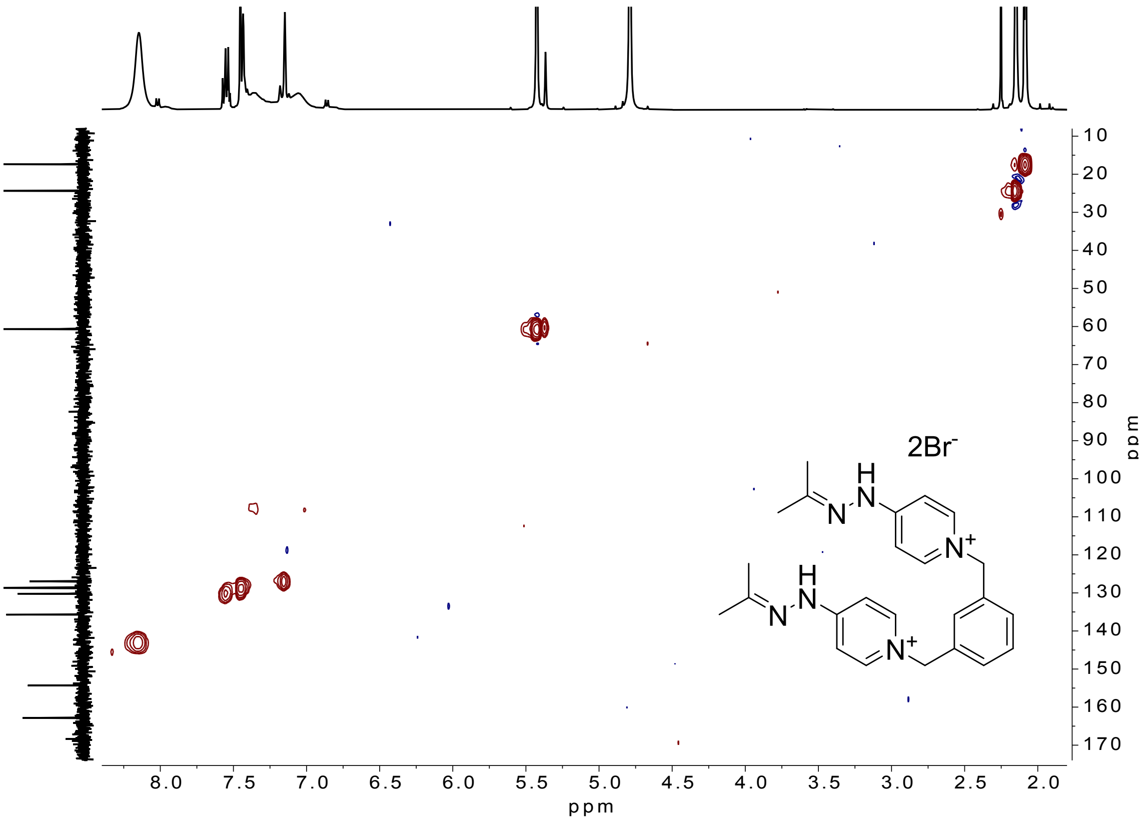

Figure S 33. ^1H-^{13}C HSQC (500 MHz/101 MHz, D_2O) spectrum of $\mathbf{H_b}$·2Br

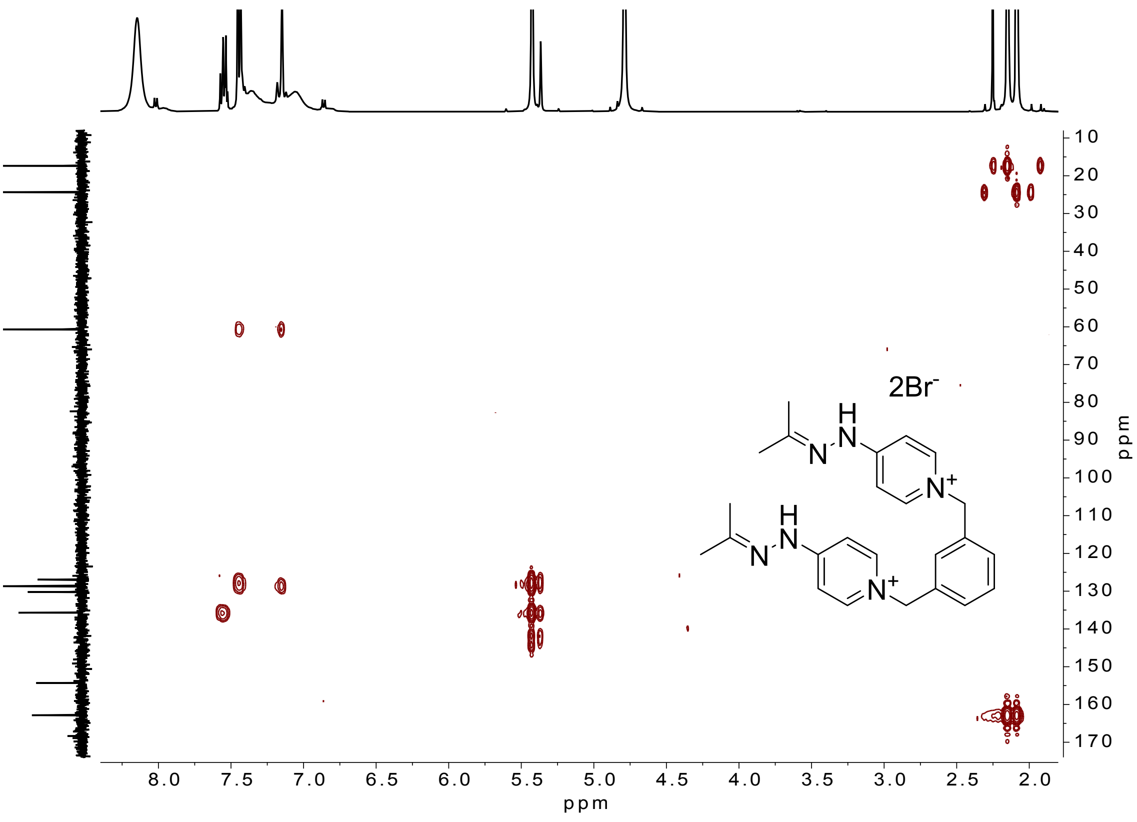

Figure S 34. ^1H-^{13}C HMBC (500 MHz/101 MHz, D_2O) spectrum of $\mathbf{H_b}$·2Br

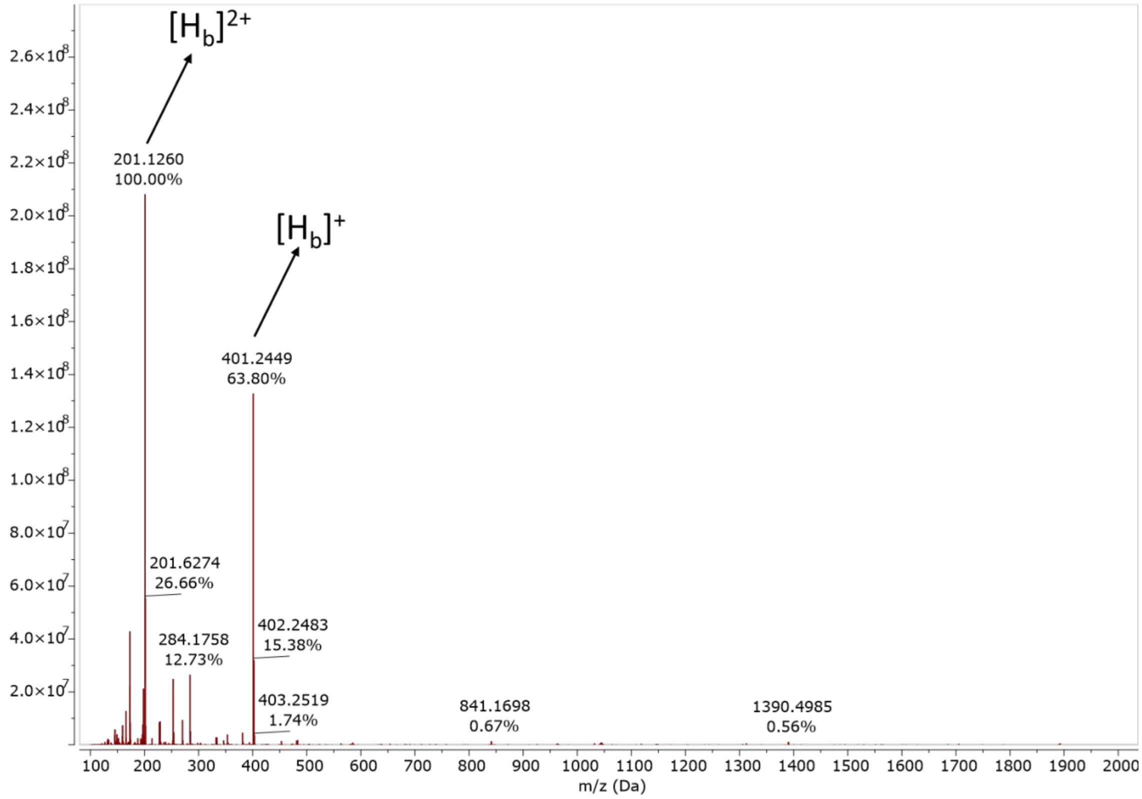

Figure S 35. HR ESI-MS spectrum of $\mathbf{H_b}$·2Br

1.2.6. **Synthesis and characterization data of H$_c$·2Br**

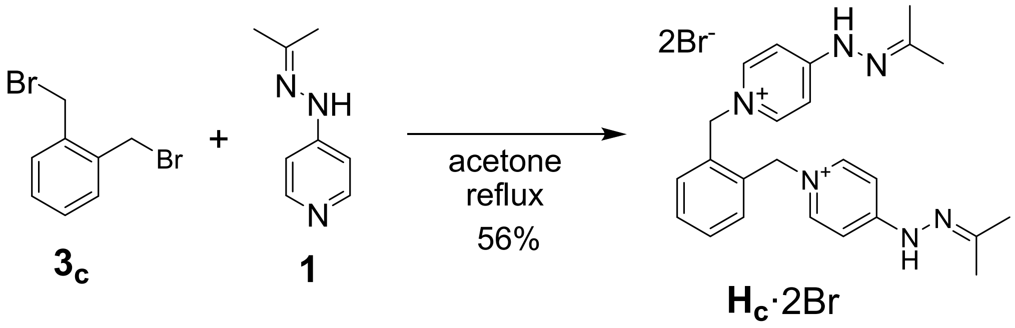

A solution 4-(2-(propan-2-ylidene)hydrazineyl)pyridine (**1**, 2,91 g, 19.5 mmol, 3 eq) in 150 mL acetone was heated at reflux for 1 hour. Then, 1,2-bis(bromomethyl)benzene (**3$_c$**, 1.72 g, 6.5 mmol, 1 eq) was added and it was heated at reflux in a magnetic hot plate stirrer for 18 h. The resulting solid was then filtered under vacuum and washed with acetone (3×30 mL) and diethyl ether (3×30 mL), yielding a whitish solid **H$_c$**·2Br (2.05 g, 56 %).

mp 250.6 – 252.4°C (decomposition). **^{1}H NMR** (500 MHz, D_2O), δ (ppm): 7.73 (d, 5H), 7.62 (m, 2H), 7.54 (m, 2H), 7.01 (d, J = 5.9 Hz, 3H), 6.70 (d, J = 6.3 Hz, 3H), 5.41 (s, 4H), 1.98 (s, 6H), 1.89 (s, 6H). **^{13}C{^{1}H} NMR** (126 MHz, D_2O), δ (ppm): 161.5 (C), 153.9 (C), 142.5 (CH), 141.2 (CH), 133.8 (CH), 131.7 (C), 131.1 (CH), 108.9 (CH), 106.9 (CH), 58.5 (CH_2), 24.4 (CH_3), 17.3 (CH_3). **HRMS** (**ESI**) m/z: [**H$_c$**]$^{2+}$ Calcd for $C_{24}H_{30}N_6^{2+}$ 201.1261; Found 201.1260; and [**H$_c$**]$^+$ Calcd for $C_{24}H_{29}N_6^+$ 401.2499; Found 401.2449.

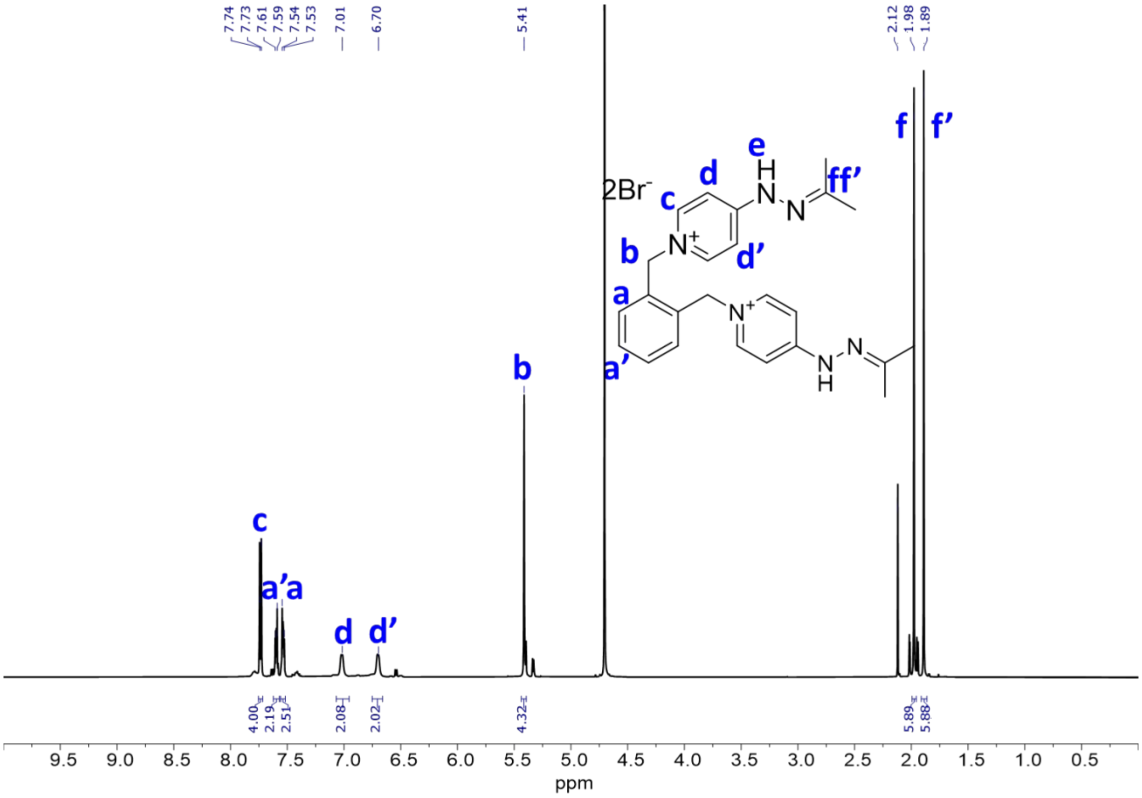

Figure S 36. ^{1}H NMR (500 MHz, D_2O) spectrum of **H$_c$**·2Br

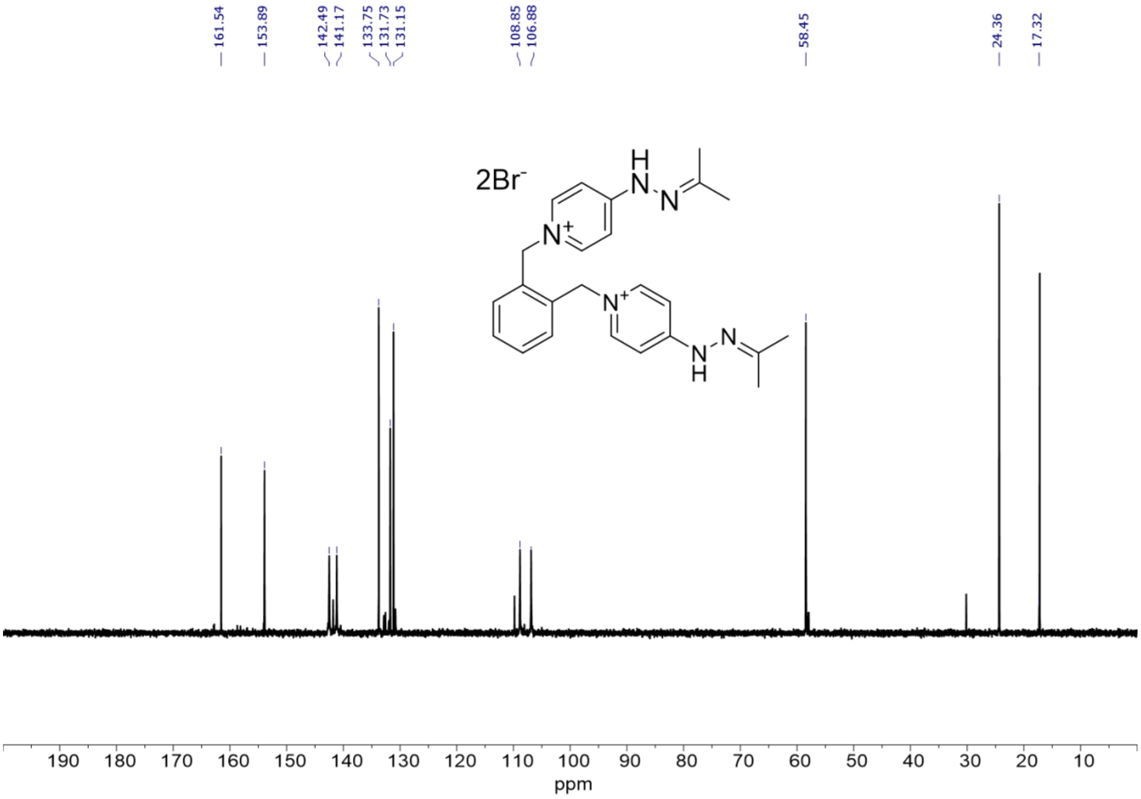

Figure S 37. $^{13}C\{^{1}H\}$ NMR (126 MHz, D_2O) spectrum of of **H_c**·2Br

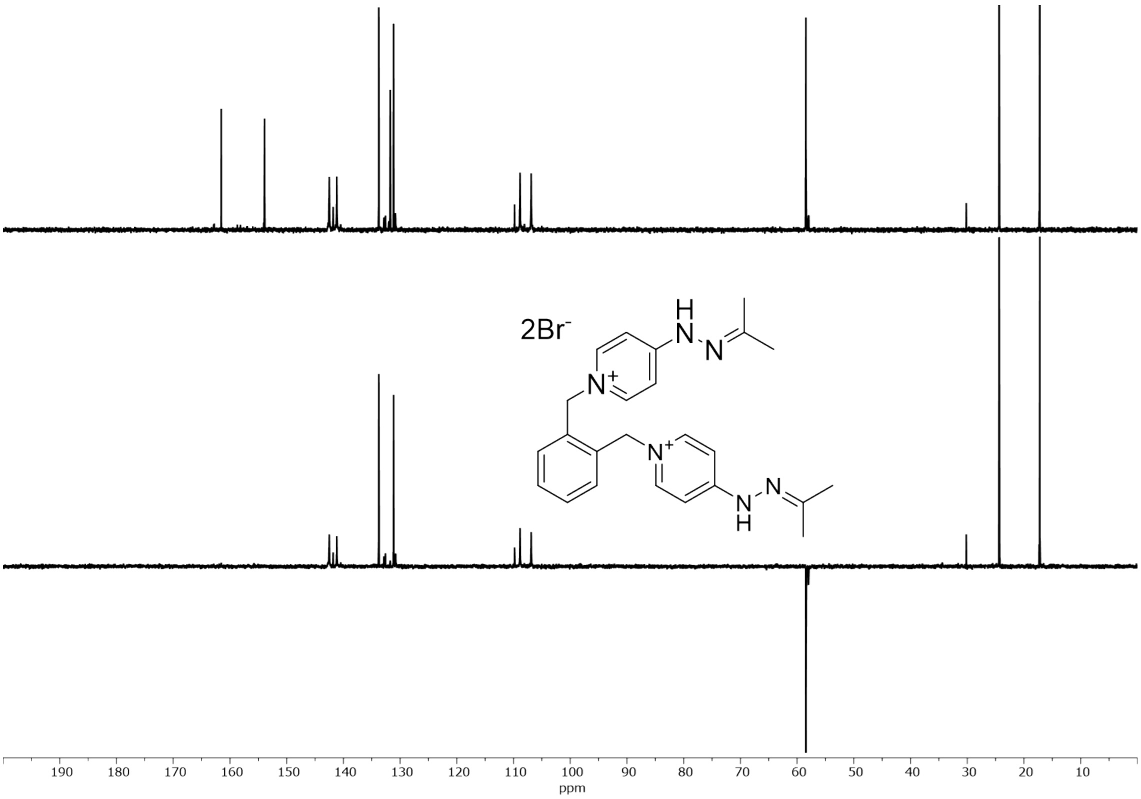

Figure S 38. $^{13}C\{^{1}H\}$ NMR (126 MHz, D_2O) spectrum (top) and DEPT-135 (126 MHz, D_2O) spectrum of **H_c**·2Br (bottom)

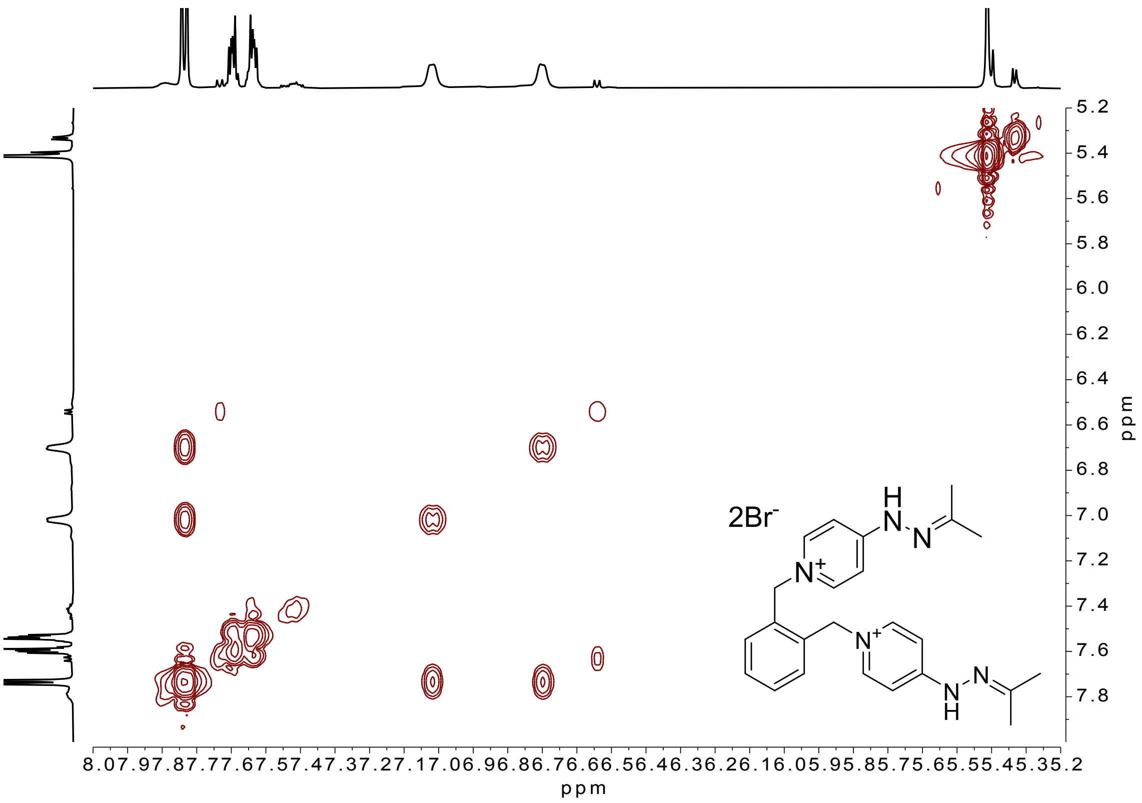

Figure S 39. ^1H-^1H COSY (500 MHz, D_2O) spectrum of $\mathbf{H_c}$·2Br

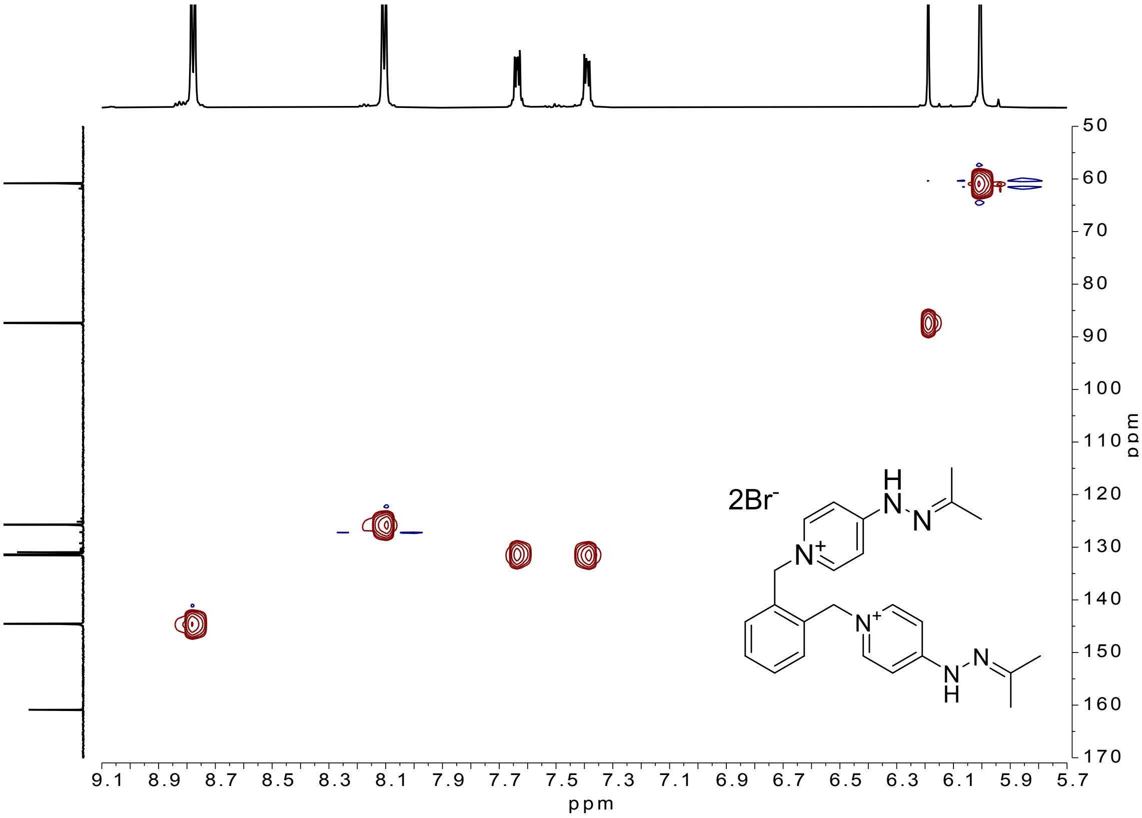

Figure S 40. ^1H-^{13}C HSQC (500 MHz, D_2O) spectrum of $\mathbf{H_c}$·2Br

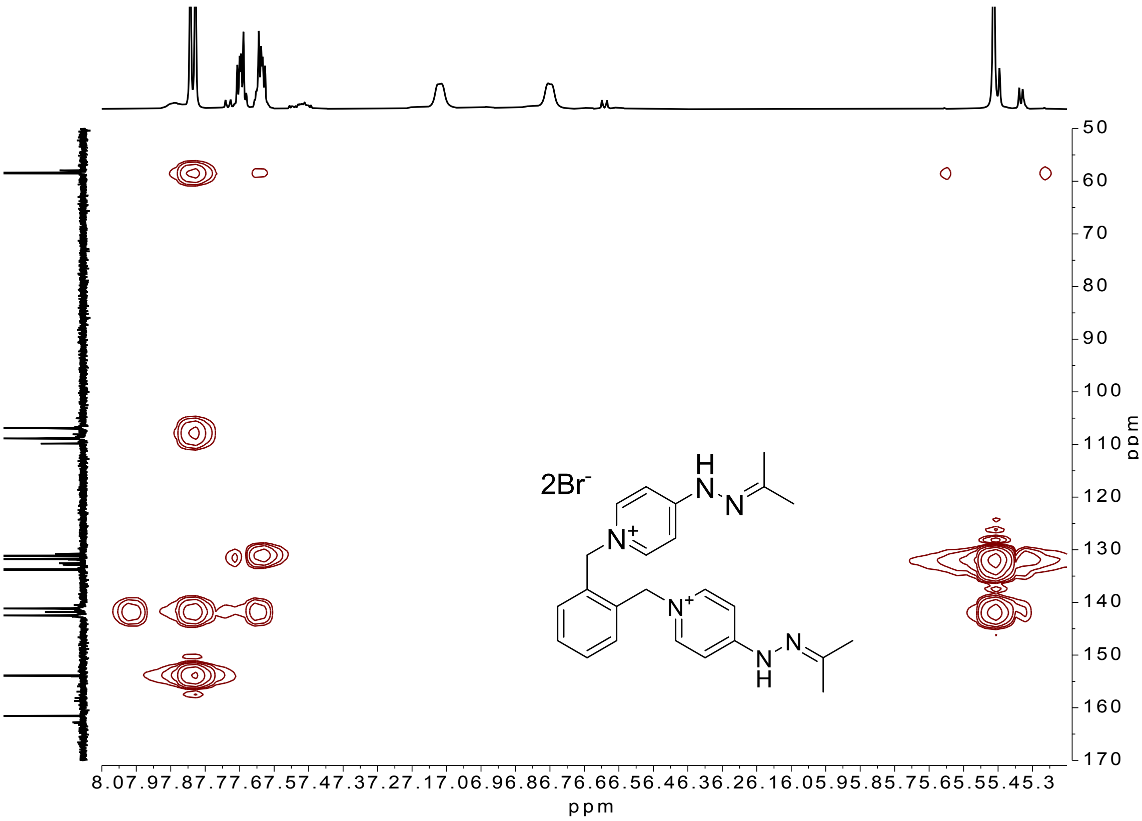

Figure S 41. ^{1}H-^{13}C HMBC (500 MHz, D_2O) spectrum of $\mathbf{H_c}$·2Br

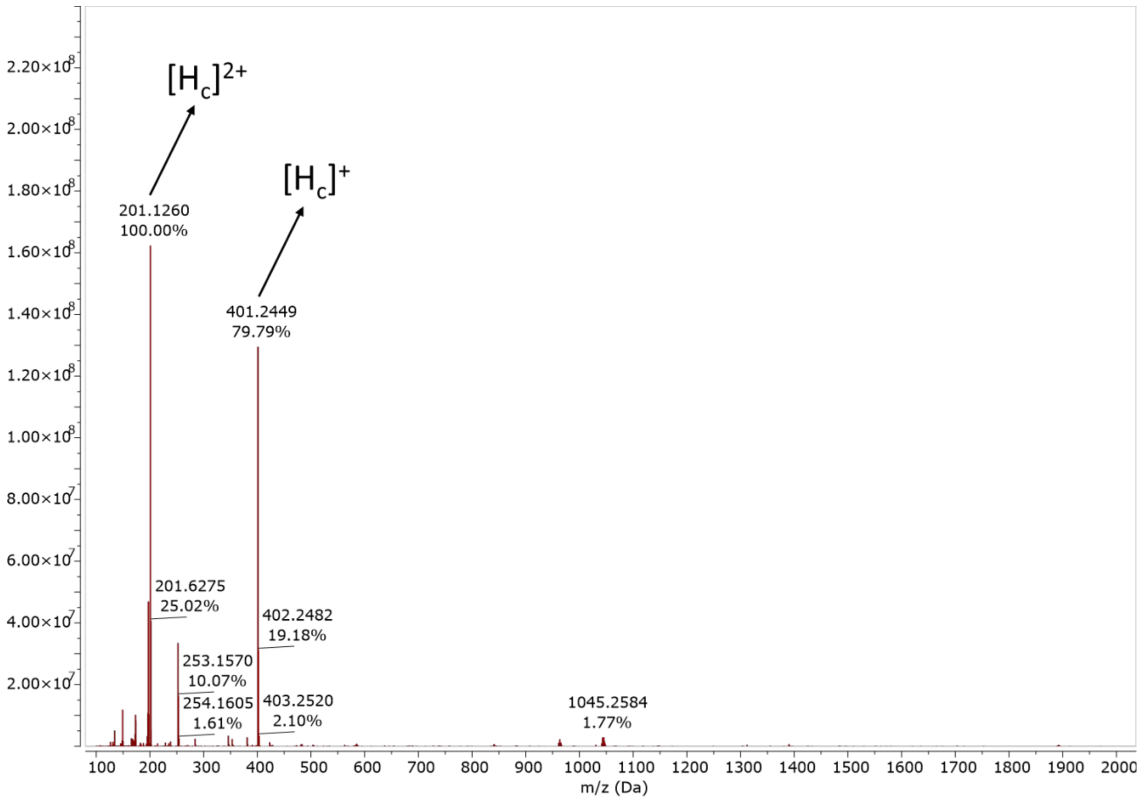

Figure S 42. HR ESI-MS spectrum of $\mathbf{H_c}$·2Br

1.2.7. **Synthesis and characterization data of H$_d$·2Br**

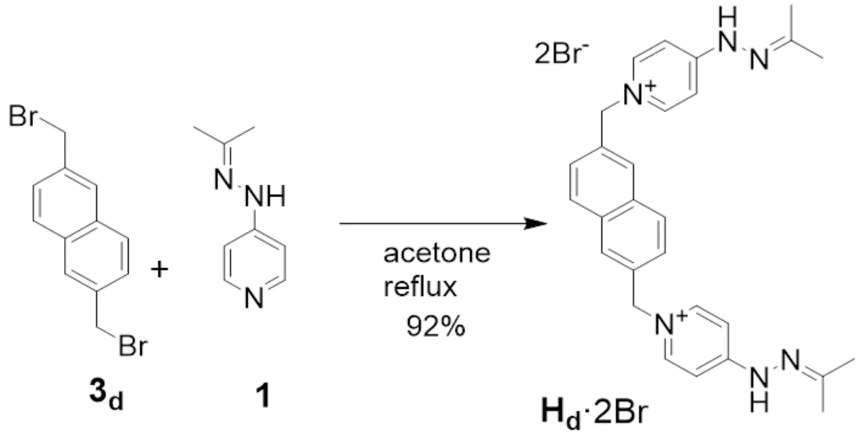

2,6-bis(bromomethyl)naphthalene (**3$_d$**, 1.88 g, 6.0 mmol, 1 eq) was dissolved in 150 mL of a solution of 4-(2-(propan-2-ylidene)hydrazineyl)pyridine (**1**, 2.69 g, 2.166 mmol, 3 eq) in commercial acetone, and it was heated at reflux in a magnetic hot plate stirrer for 24 hours. After, the obtaining solid was vacuum filtered and washed with acetone (3×30 mL) and diethyl ether (3×30 mL), yielding a grayish solid, **H$_d$**·2Br (3.38 g, 92%).

mp 255.2 – 257.0°C (decomposition). **^{1}H NMR** (500 MHz, D_2O), δ (ppm): 8.17 (s, 4H), 7.96 (dd, J = 8.5, 2.3 Hz, 2H), 7.87 (s, 2H), 7.49 (dd, J = 8.6, 1.7 Hz, 2H), 7.32 (s, 1H), 7.00 (s, 1H), 5.50 (s, 4H), 2.10 (s, 6H), 2.01 (s, 6H). **^{13}C{^{1}H} NMR** (126 MHz, D_2O), δ (ppm): 162.8 (C), 154.3 (C), 133.0 (C), 132.8 (C), 129.3 (CH), 127.3 (CH), 127.3 (CH), 126.1 (CH), 60.8 (CH_2), 24.3 (CH_3), 17.3 (CH_3). **HRMS** (**ESI**) m/z: [**H$_d$**]$^+$ Calcd for $C_{28}H_{31}N_6^+$ 451.2604; Found 451.2605.

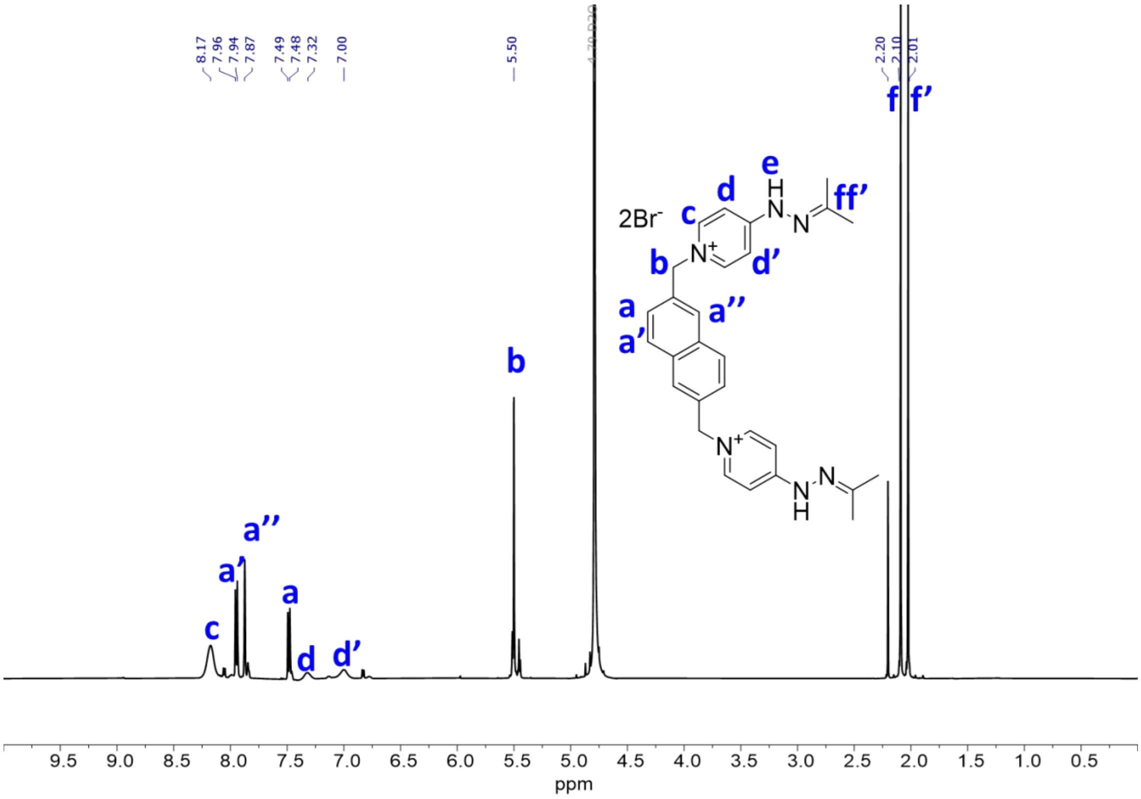

Figure S 43. ^{1}H NMR (500 MHz, D_2O) spectrum of $\mathbf{H_d}$·2Br

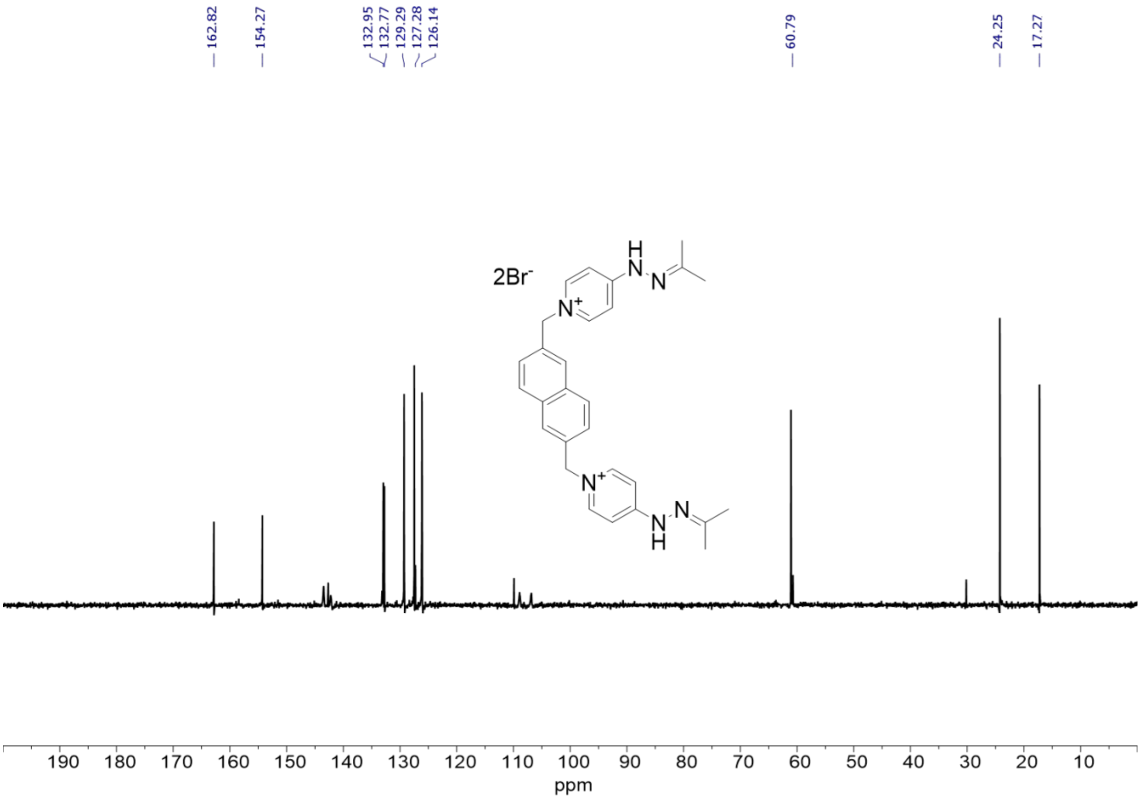

Figure S 44. 13C{1H} NMR (126 MHz, D2O) spectrum of of $\mathbf{H_d}$·2Br

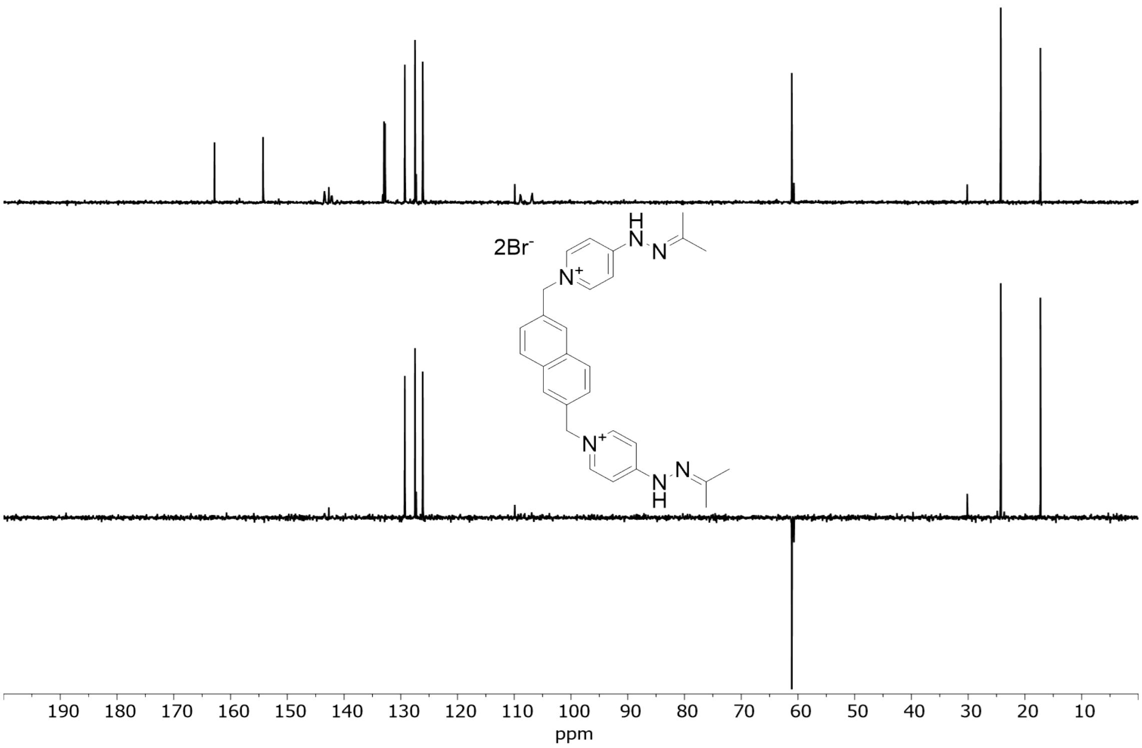

Figure S 45. $^{13}C\{1H\}$ NMR (126 MHz, D_2O) spectrum (top) and DEPT-135(126 MHz, D_2O) spectrum (bottom) of $\mathbf{H_d}$·2Br

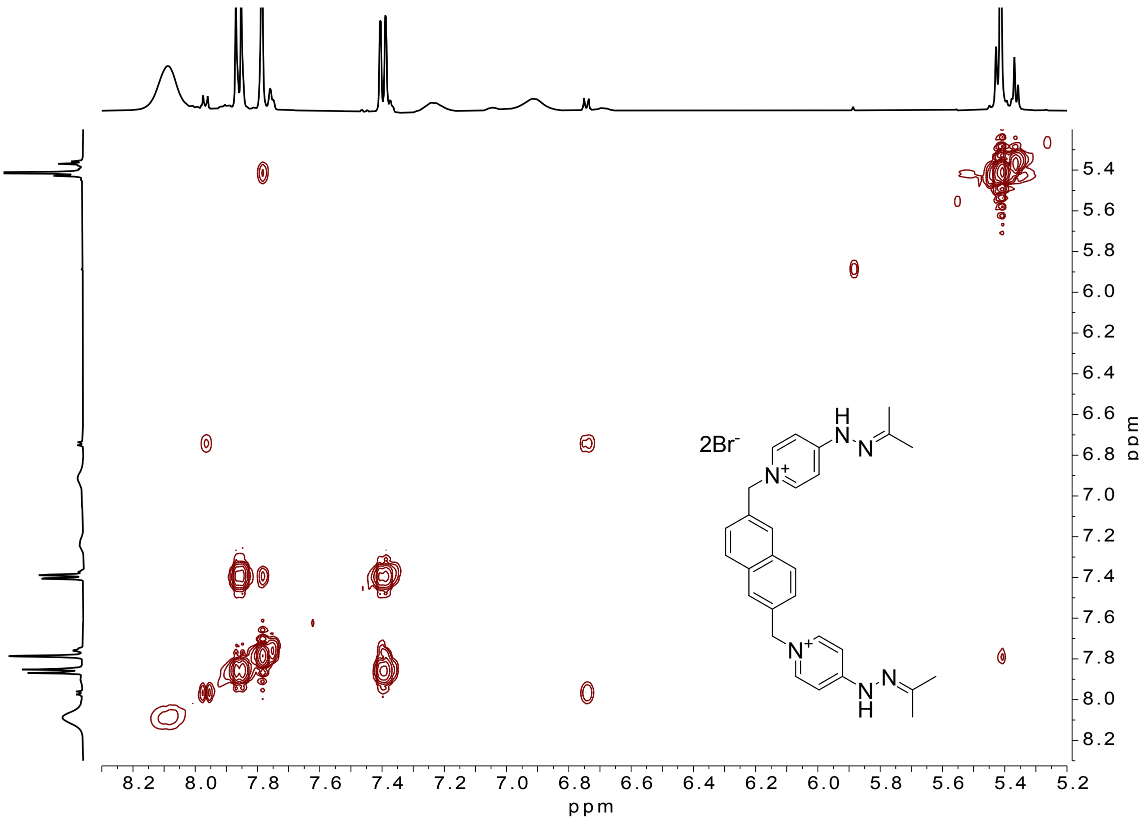

Figure S 46. ^{1}H-^{1}H COSY NMR (500 MHz, D_2O) spectrum of $\mathbf{H_d}$·2Br

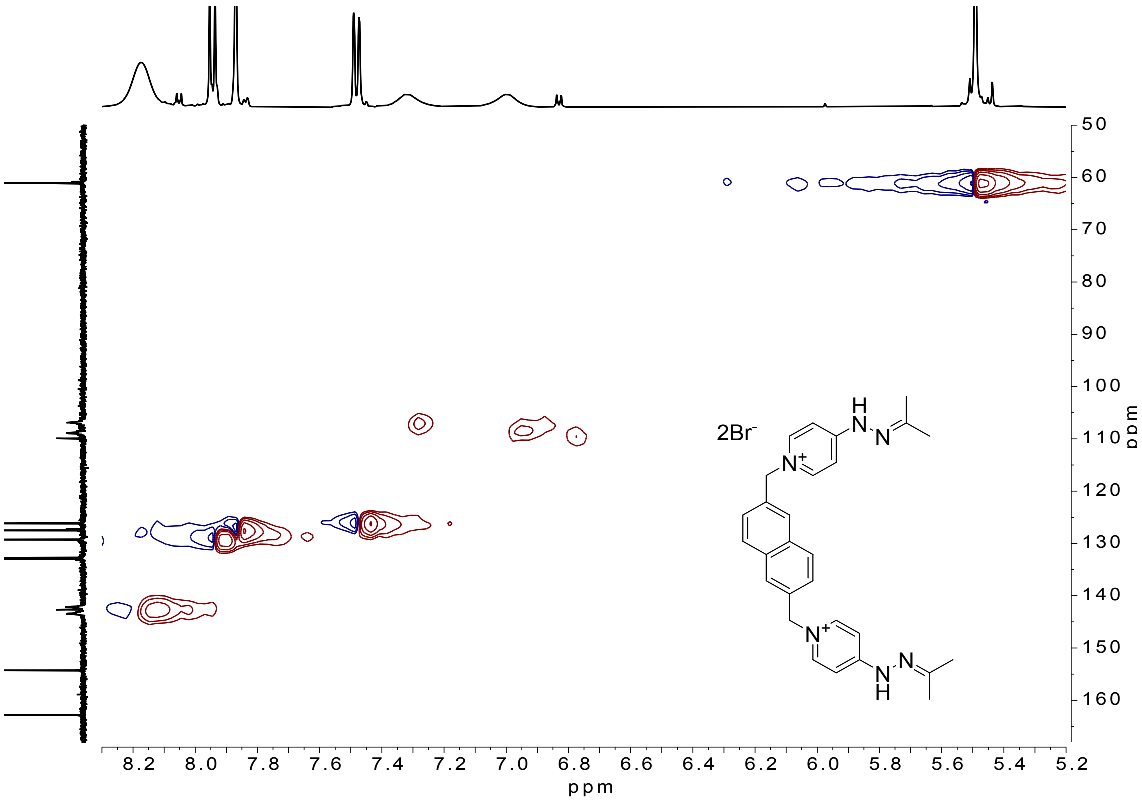

Figure S 47. ^{1}H-^{13}C HSQC (500 MHz/126 MHz, D_2O) spectrum of $\mathbf{H_d}$·2Br

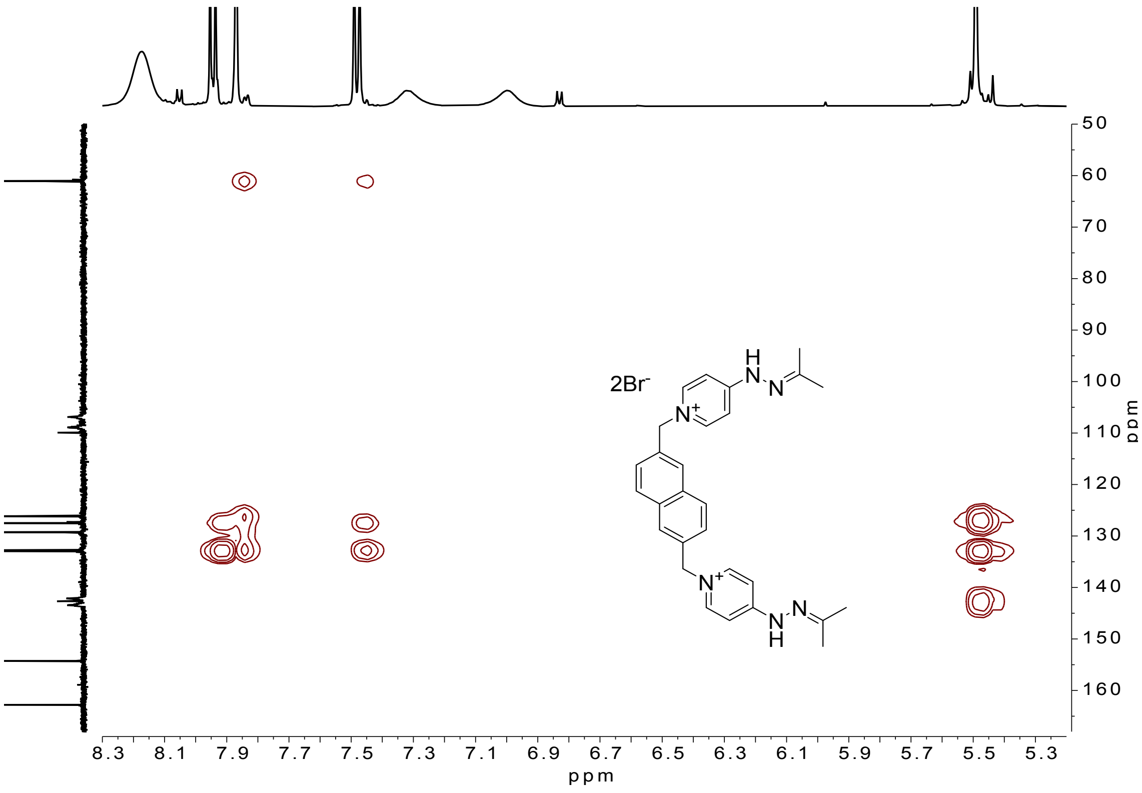

Figure S 48. ^{1}H-^{13}C HMBC (500 MHz/126 MHz, D_2O) spectrum of H_d·2Br

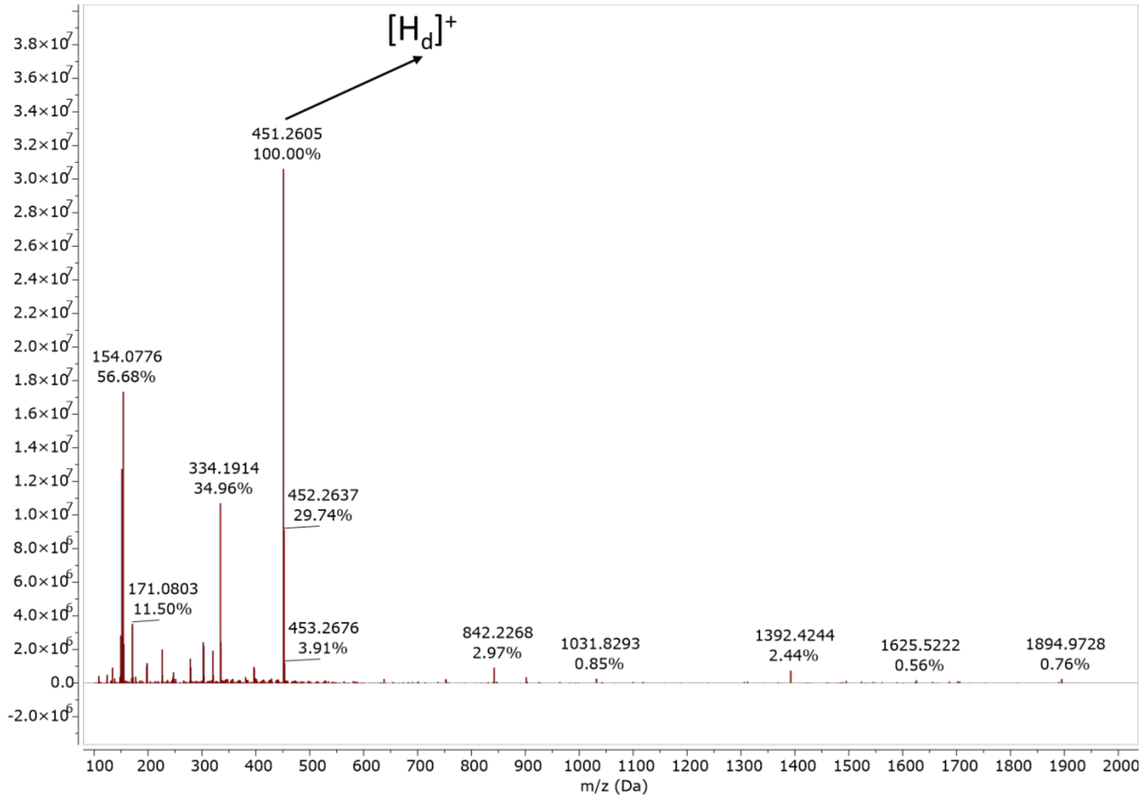

Figure S 49. HR ESI-MS spectrum of $\mathbf{H_d}$·2Br

1.2.8. Synthesis and characterization data of H_e·2Br

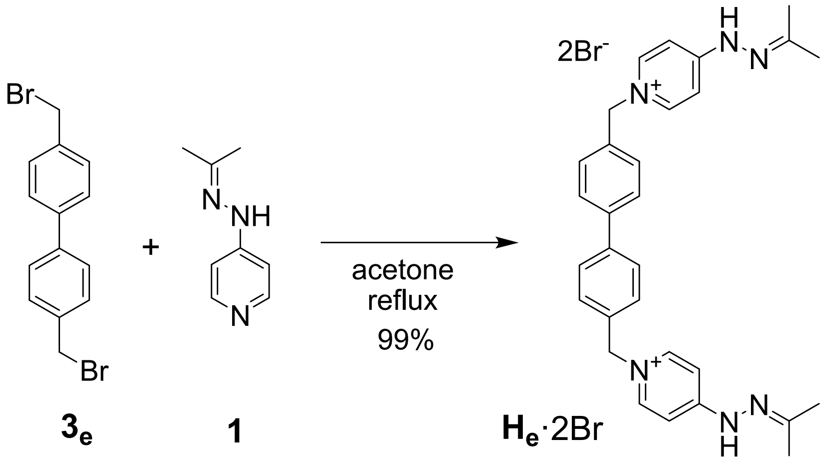

To a solution of 4-(2-(propan-2-ylidene)hydrazineyl)pyridine (**1**, 2.69 g, 18.0 mmol, 3 eq) in 150 mL of acetone, 4,4'-bis(bromomethyl)biphenyl (**3**$_e$, 2.04 g, 6.0 mmol, 1 eq) was added and the solution was heated at reflux in a magnetic hot plate stirrer for 24 h. The resulting solid was then filtered under vacuum and washed with acetone (3×30 mL) and diethyl ether (3×30 mL), yielding a grayish solid **H**$_e$·2Br (3.79 g, 99 %).

mp 258.8 – 260.4°C (decomposition). **^{1}H NMR** (500 MHz, D_2O), δ (ppm): 8.08 (s, 4H), 7.57 (d, J = 7.9 Hz, 4H), 7.38 (d, J = 8.0 Hz, 4H), 7.23 (s, 2H), 6.93 (s, 2H), 5.29 (s, 4H), 2.08 (s, 6H), 2.00 (s, 6H). **^{13}C{^{1}H} NMR** (126 MHz, D_2O), δ (ppm): 162.3 (C), 154.1 (C), 140.2 (C), 134.0 (C), 128.8 (CH), 127.6 (CH), 60.7(CH_2), 24.3 (CH_3), 17.3 (CH_3). **HRMS** (**ESI**) m/z: [**H**$_e$]$^+$ Calcd for $C_{30}H_{33}N_6^+$ 477.2761; Found 477.2763.

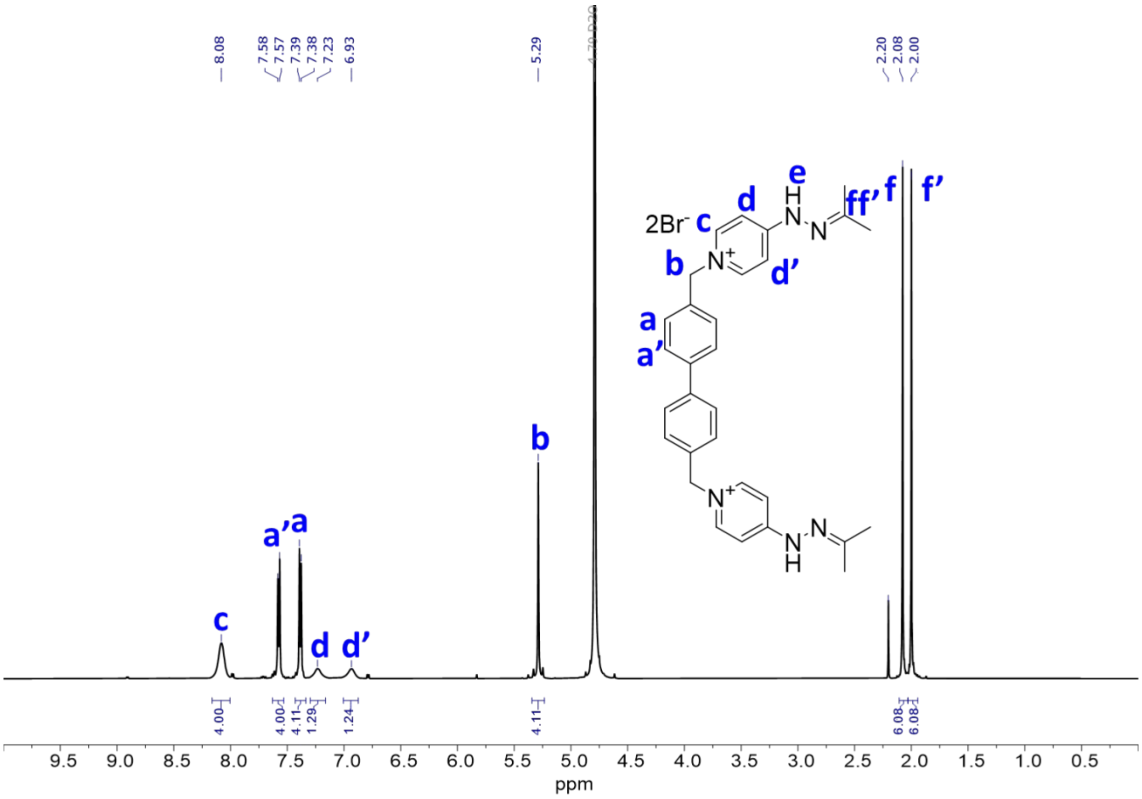

Figure S 50. ^{1}H NMR (500 MHz, D_2O) spectrum of $\mathbf{H_e}$·2Br

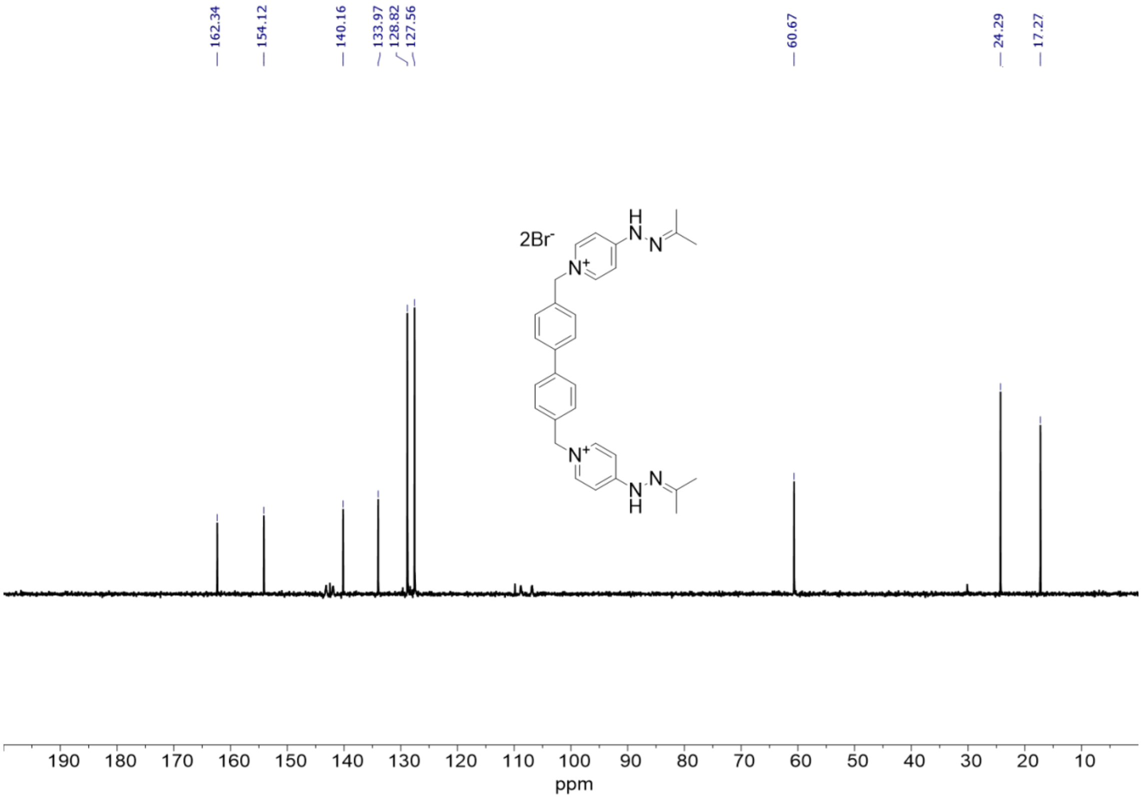

Figure S 51. $^{13}C\{^{1}H\}$ NMR (126 MHz, D_2O) spectrum of of $\mathbf{H_e}$·2Br

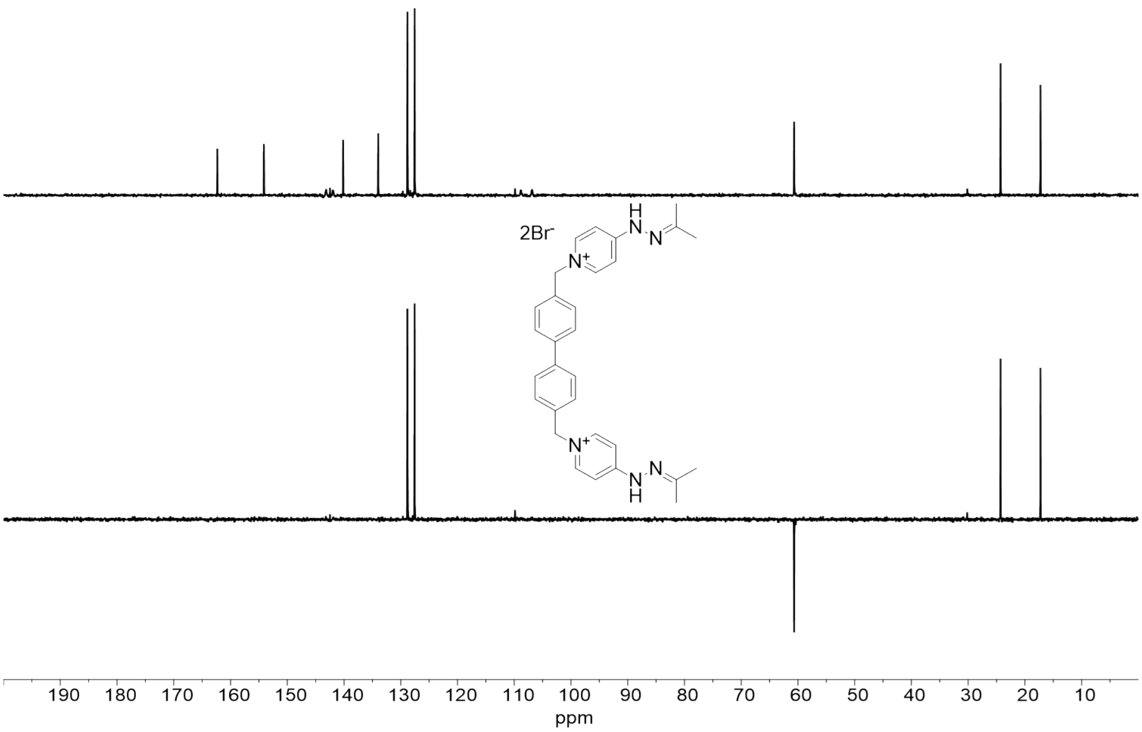

Figure S 52. $^{13}C\{^1H\}$ NMR (126 MHz, D_2O) spectrum (top) and DEPT-135 (126 MHz, D_2O) spectrum of $\mathbf{H_e}$·2Br (bottom)

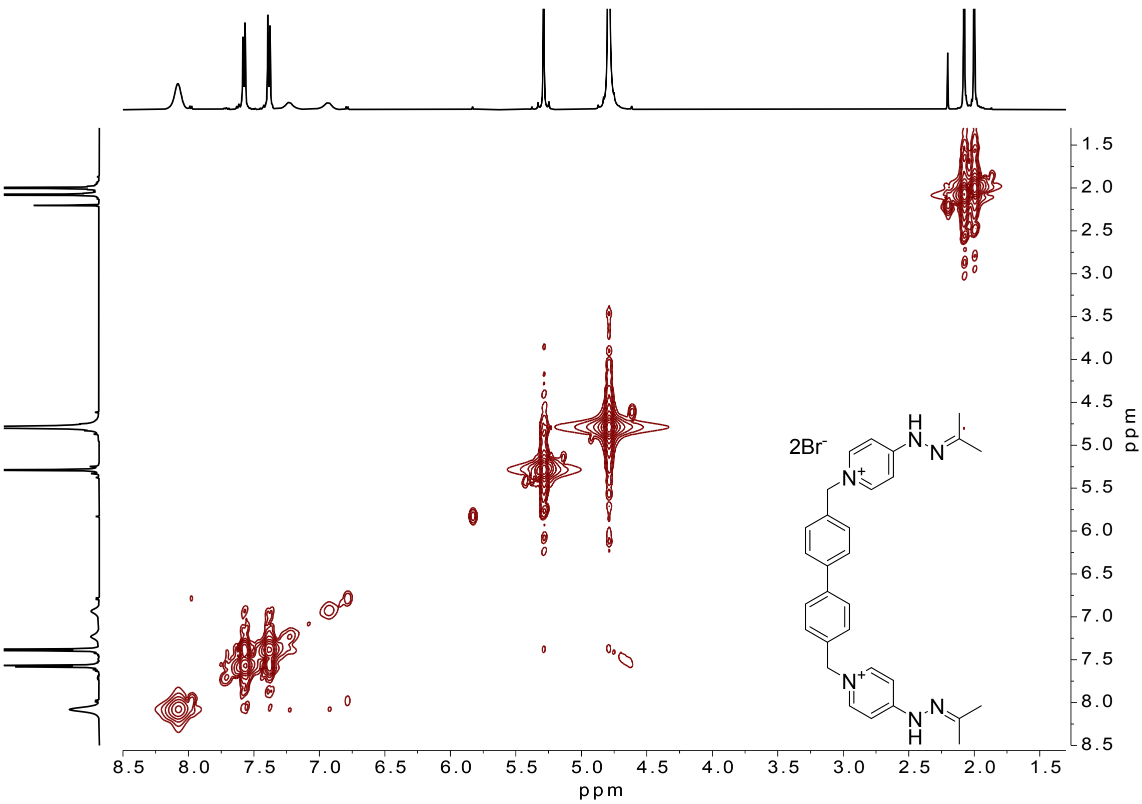

Figure S 53. ^1H-^1H COSY (500 MHz, D_2O) spectrum of $\mathbf{H_e}$·2Br

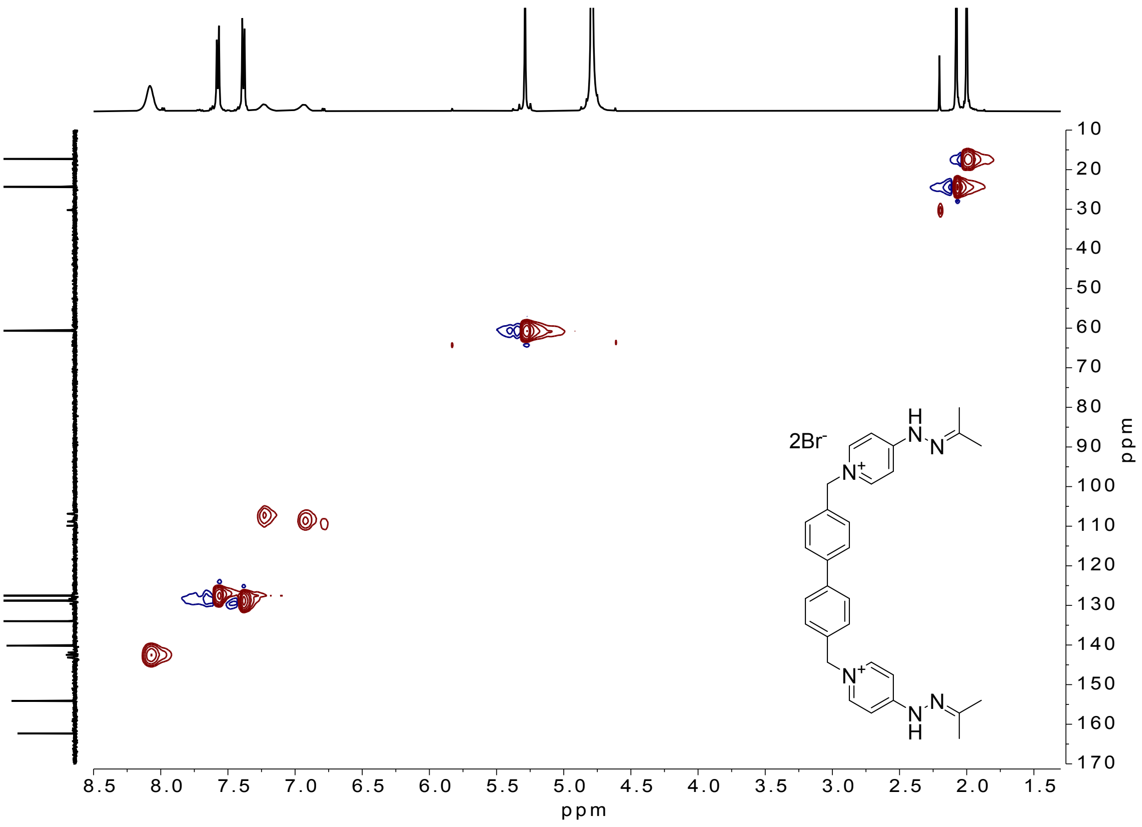

Figure S 54. ^{1}H-^{13}C HSQC (500 MHz, D_2O) spectrum of $\mathbf{H_e}$·2Br

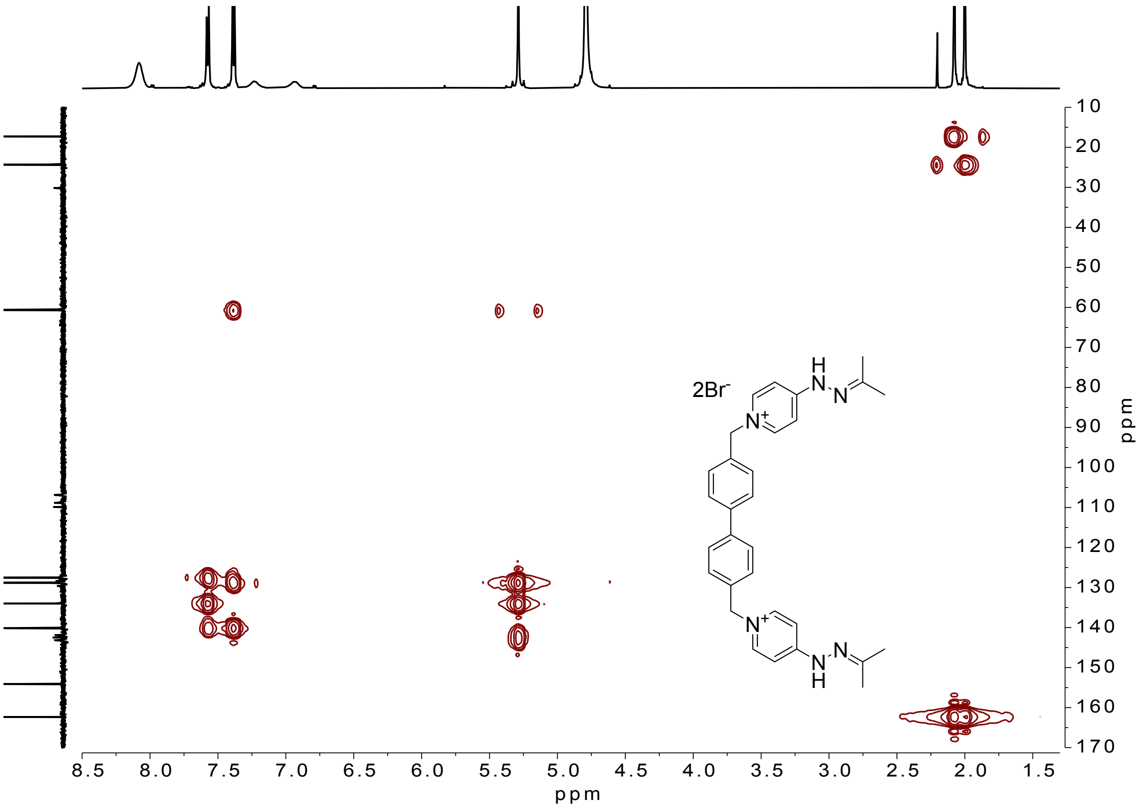

Figure S 55. ^{1}H-^{13}C HMBC (500 MHz, D_2O) spectrum of $\mathbf{H_e}$·2Br

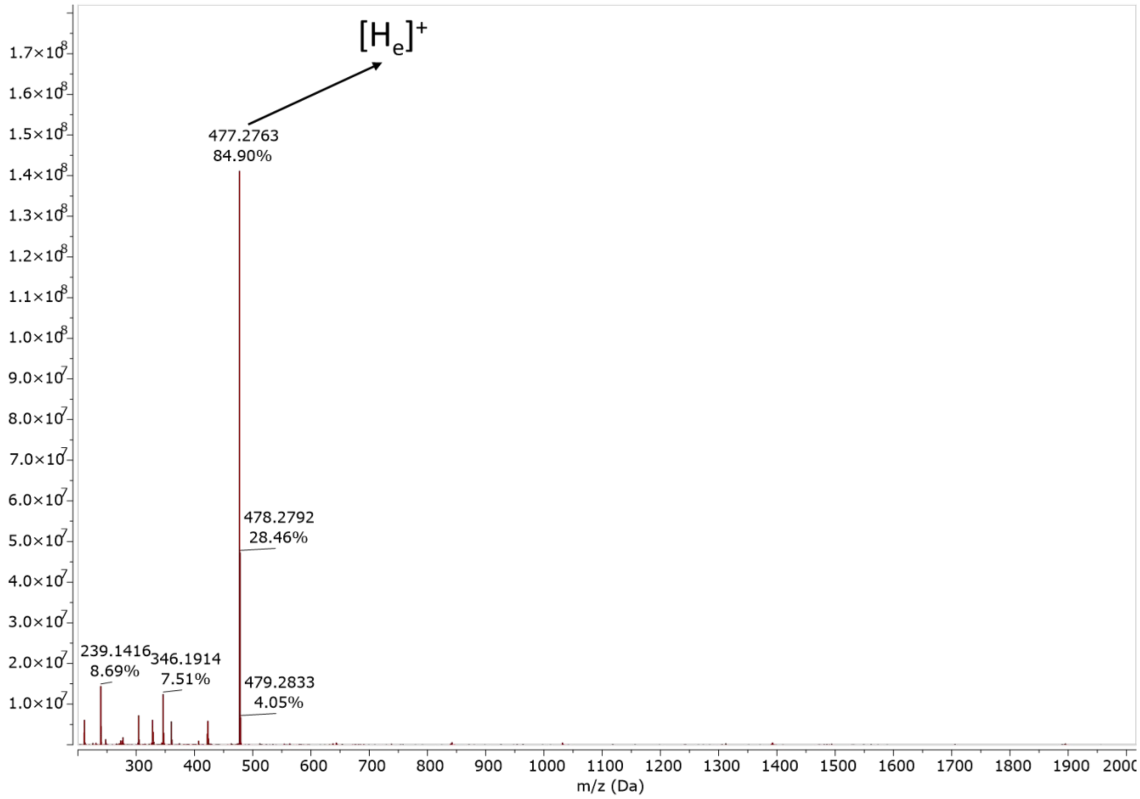

Figure S 56. HR ESI-MS spectrum of $\mathbf{H_e}$·2Br

1.2.9. Synthesis and characterization data of $\mathbf{R_bH_2 \cdot 4TFA}$

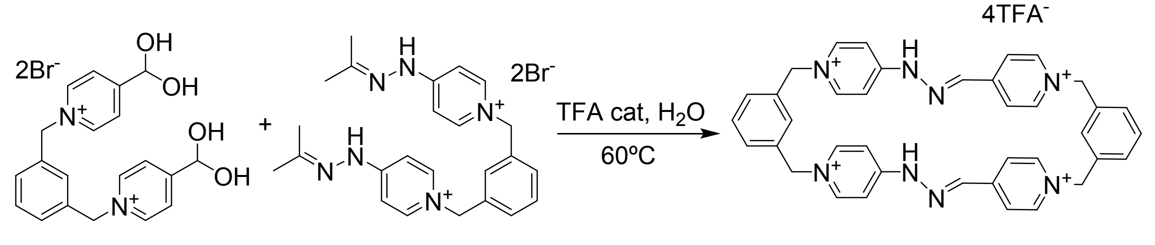

A_b·2Br **H_b**·2Br **R_b**H_2·4TFA

A_b·2Br (1.83 g, 3.56 mmol, 1 eq), and **H_b**·2Br (2.00 g, 3.56 mmol, 1 eq) were dissolved in a solution of 1.4 L of water and 27.4 µL (10 mol%) of trifluoroacetic acid (divided into three 500 mL round bottom flasks). The mixture is heated and stirred at 60°C for 20 h using a magnetic hot plate stirrer. After checking the completion of the reaction by NMR, the reaction is cooled and an excess of KPF_6 was added until no further precipitation was observed. The mixture was left stirring at room temperature for 30 min. The obtaining reddish solid is then filtered under vacuum and washed with water (3×50 mL) and diethyl ether (3×50 mL). 3.04 g of **R_b**H_2·4PF_6 were obtained with a purity of 80%, achieving a yield of 72%. The product was purified by reverse-phase semipreparative HPLC (RP, H_2O + 0.1% TFA/CH_3CN + 0.1% TFA = 5/95, flow rate = 0.3 mL/min, I = 220 nm, t_R = 10.9 min), yielding **R_b**H_2·4TFA as a yellowish solid (1.47 g, 58 %), with an overall reaction yield of 42%.

mp 309.4 – 310.7°C (decomposition). **^{1}H NMR** (500 MHz, D_2O) δ (ppm): 8.70 (d, J = 7.0 Hz, 4H), 8.41 (bs, 1H), 8.22 (s, 2H), 8.20 (d, J = 6.9 Hz, 2H), 8.13 (bs, 1H), 7.75 (bs, 1H), 7.61 (m, 3H), 7.54 (d, J = 1.4 Hz, 3H), 7.17 (bs, 1H), 6.95 (s, 1H), 6.80 (s, 1H), 5.77 (s, 4H), 5.49 (s, 4H). **^{13}C{^{1}H} NMR** (101 MHz, D_2O) δ (ppm): 154.5 (C), 149.9 (C), 144.6 (CH), 140.9 (CH), 135.5 (C), 134.6 (C), 130.8 (CH), 130.7 (CH), 130.4 (CH), 129.6 (CH), 127.5 (CH), 126.4 (CH), 124.7 (CH), 63.6 (CH_2), 61.5 (CH_2). **HRMS** (**ESI**) m/z: [**R_b**H-H$^+$]$^{3+}$ Calcd for $C_{38}H_{35}N_8^{3+}$ 201.0989; Found 201.0988; [**R_b**-2H$^+$]$^{2+}$ Calcd for $C_{38}H_{34}N_8^{2+}$ 301.1448; Found 301.1447; and [**R_b**-2H$^+$+TFA$^-$]$^+$ Calcd for $C_{40}H_{34}F_3N_8O_2^+$ 715.2751; Found, 715.2755.

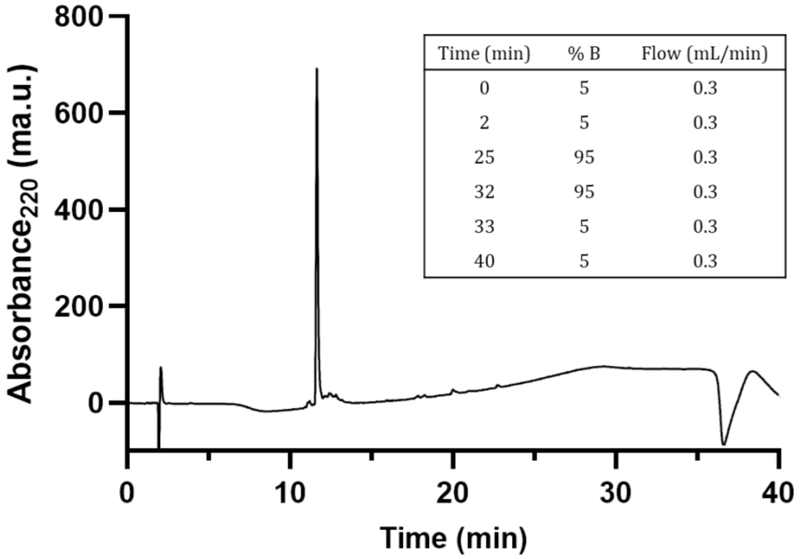

Figure S 57. HPLC chromatogram (220 nm) of $\mathbf{R_b}H_2 \cdot 4PF_6$ at t_R = 11.6 min. *Inset.* Elution conditions

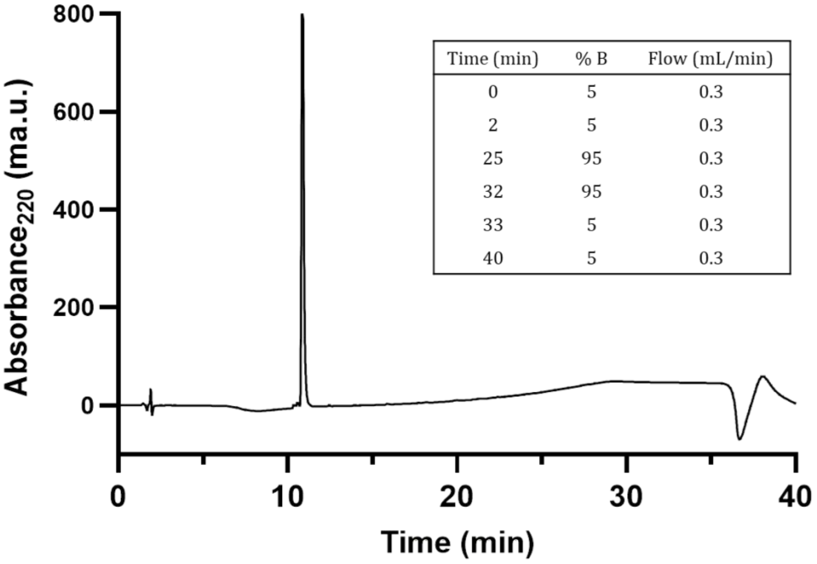

Figure S 58. HPLC chromatogram (220 nm) of purified $\mathbf{R_b}H_2 \cdot 4TFA$ at t_R = 10.9 min. *Inset*. Elution conditions

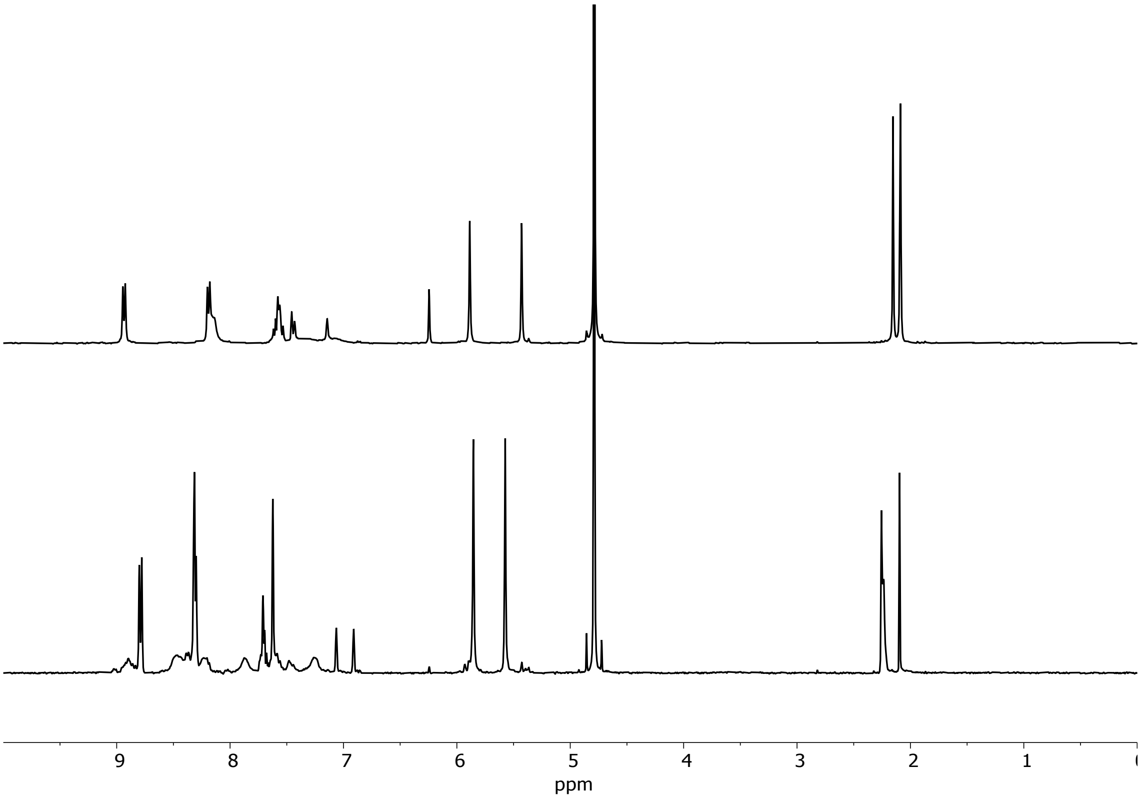

Figure S 59. ^{1}H NMR (300 MHz, D_2O) stacked spectra of: (top) equimolar 2.5 mM mixture of $\mathbf{A_b}$·2Br and $\mathbf{H_b}$·2Br at t = 0; (bottom) same mixture after 24 hours at 60 °C with TFA-d_3 (10% molar)

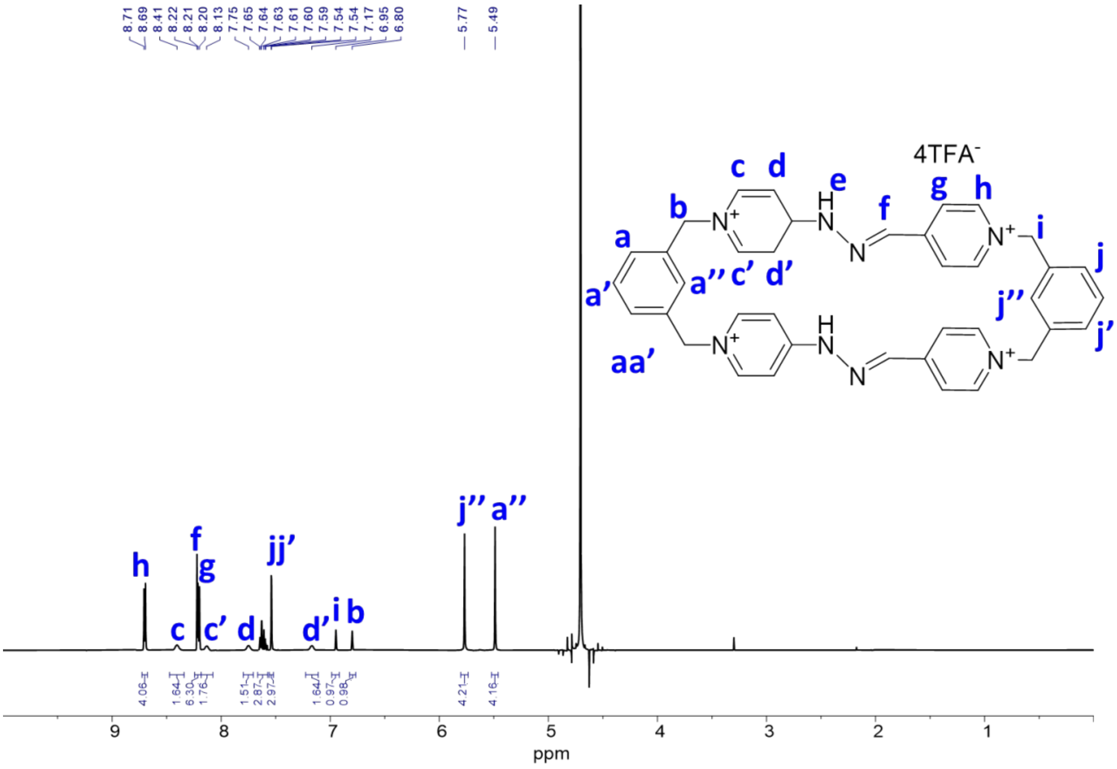

Figure S 60. ^{1}H NMR (500 MHz, D_2O) spectrum of $\mathbf{R_b}H_2$·4TFA

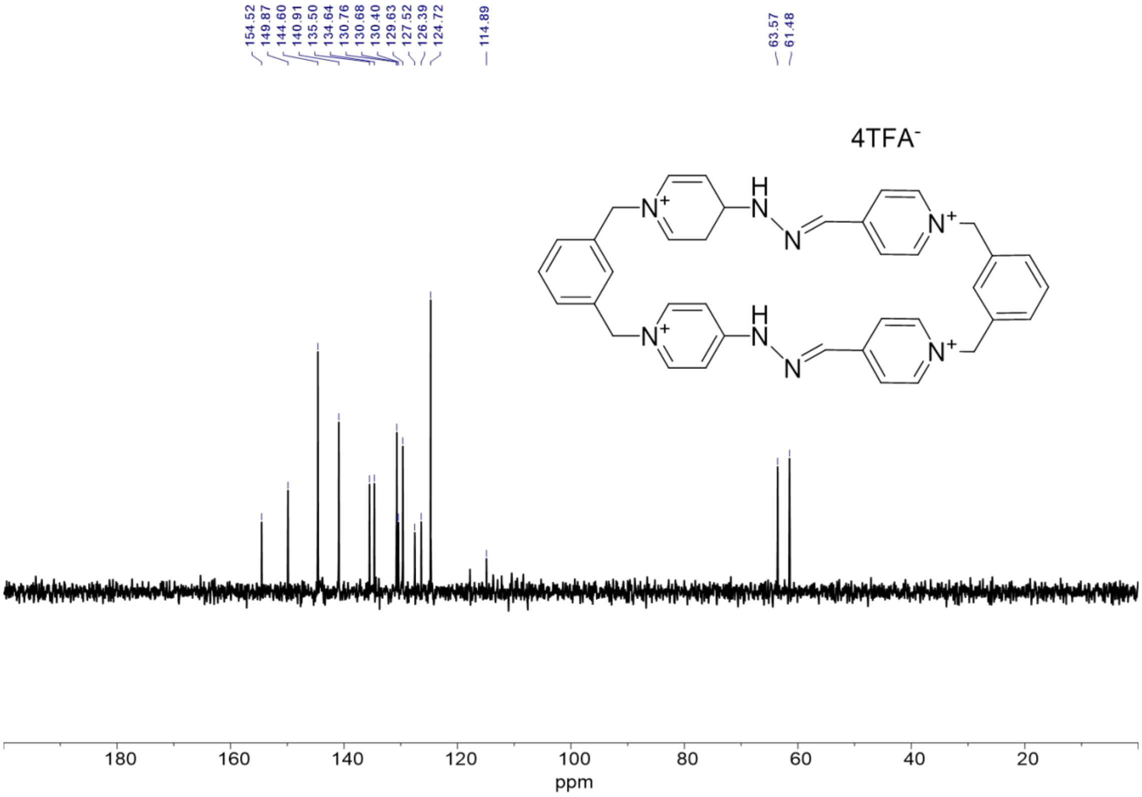

Figure S 61. $^{13}C\{^1H\}$ NMR (101 MHz, D_2O) spectrum of $\mathbf{R_b}H_2$·4TFA

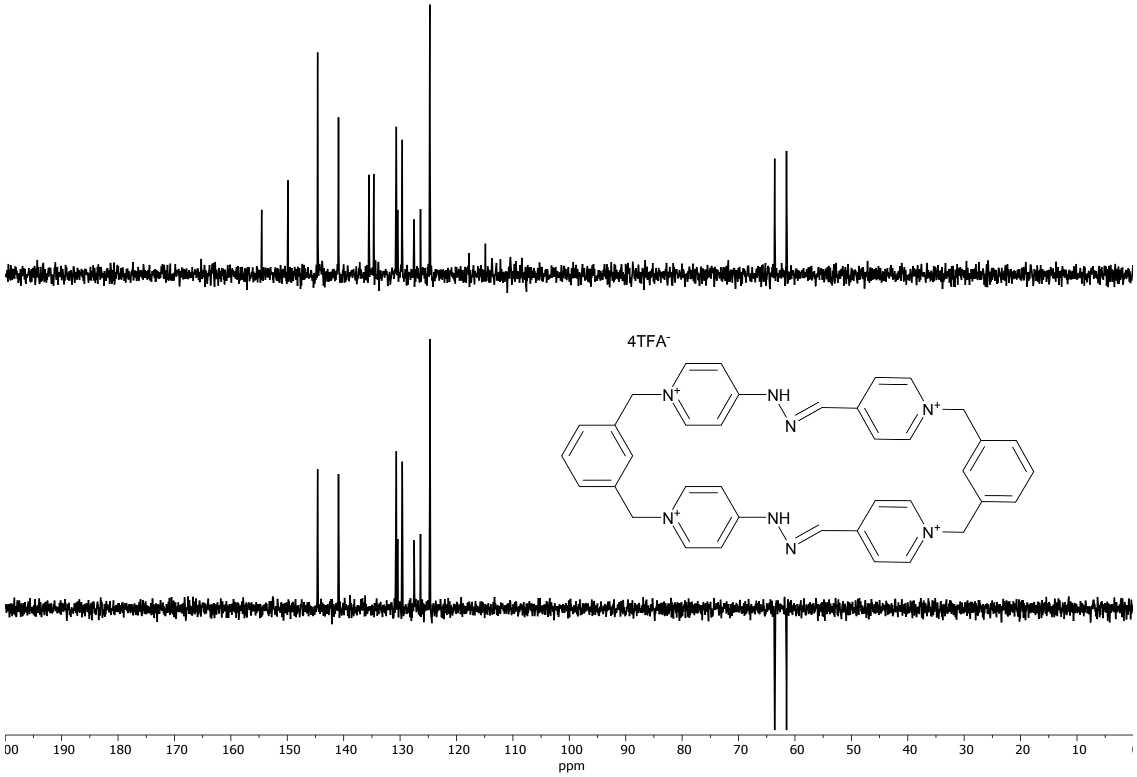

Figure S 62. $^{13}C\{^1H\}$ NMR (101 MHz, D_2O) spectrum (top) and DEPT-135 (101 MHz, D_2O) spectrum (bottom) of $\mathbf{R_b}H_2$·4TFA

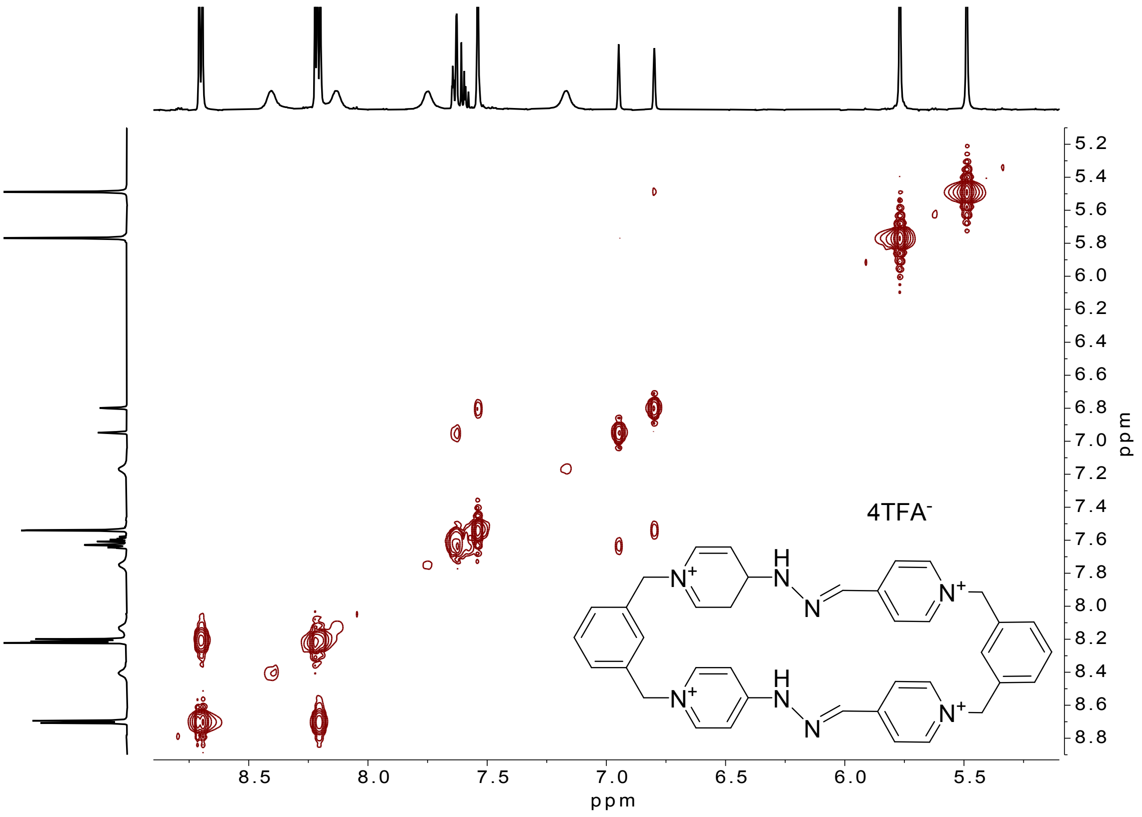

Figure S 63. ^{1}H-^{1}H COSY (500 MHz, D_2O) spectrum of $\mathbf{R_b}H_2$·4TFA

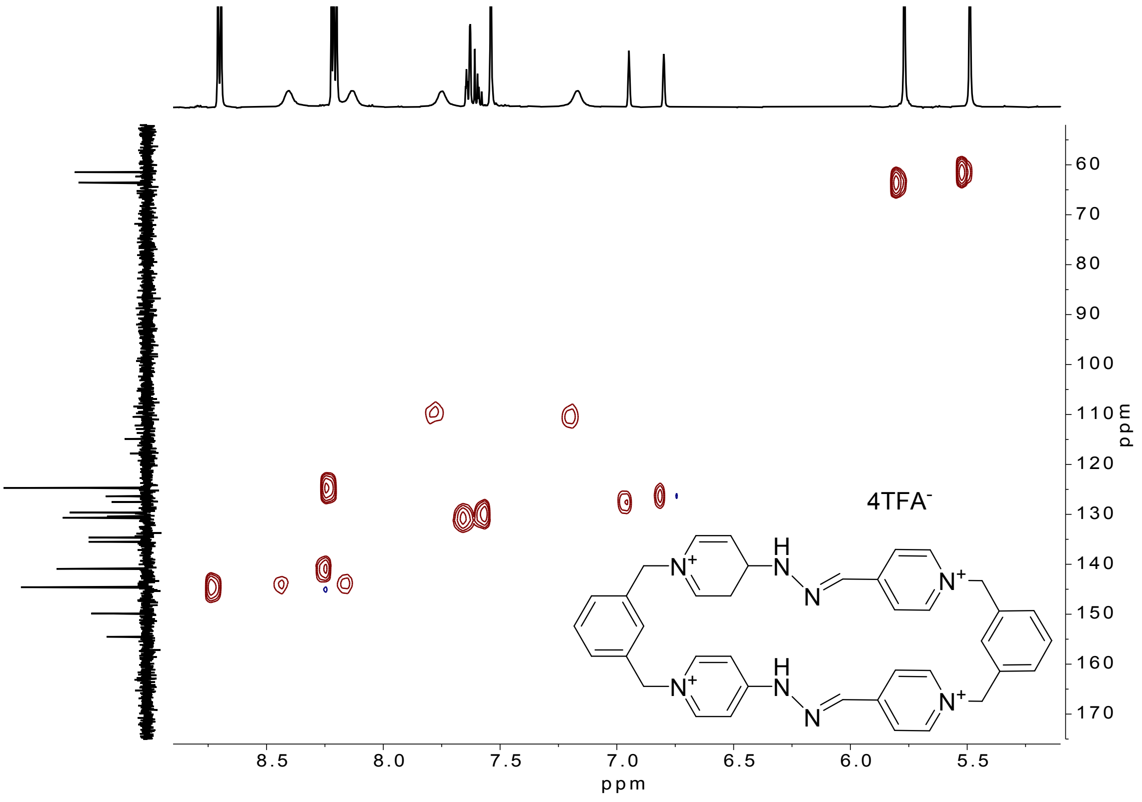

Figure S 64. ^{1}H-^{13}C HSQC (500 MHz/101 MHz, D_2O) spectrum of $\mathbf{R_b}H_2$·4TFA

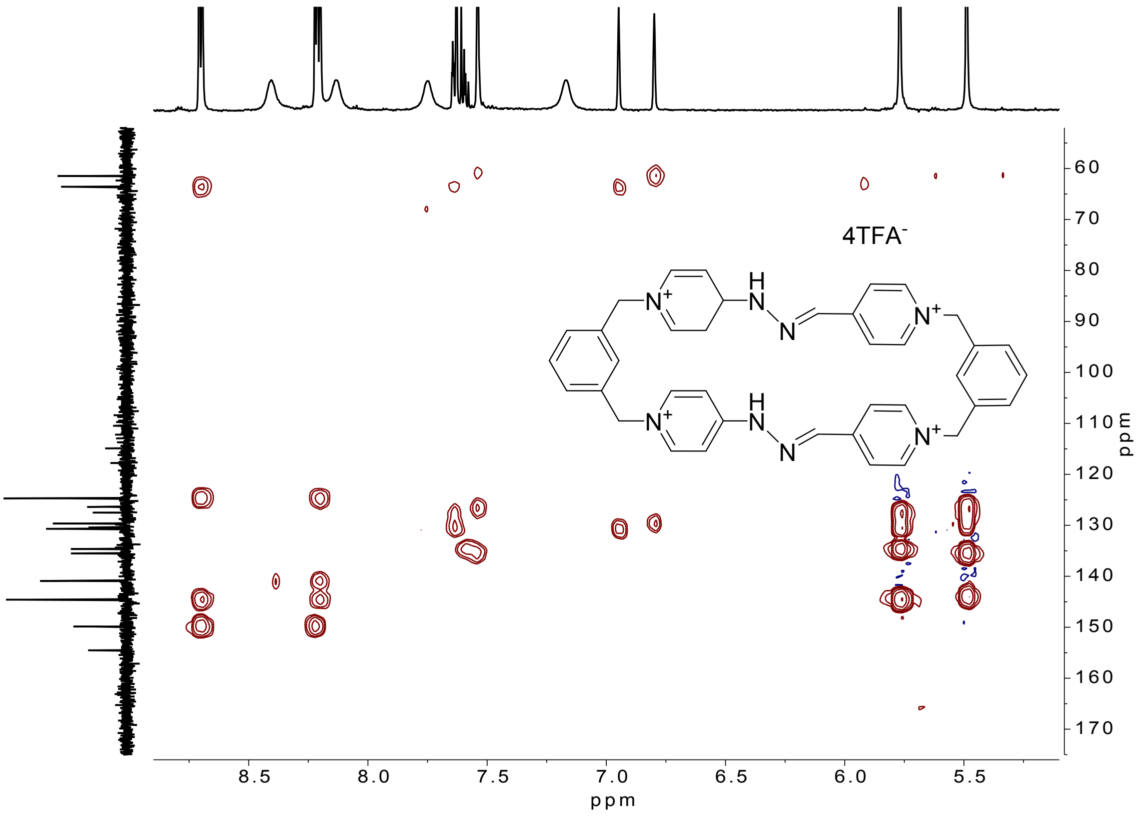

Figure S 65. ^{1}H-^{13}C HMBC (500 MHz/101 MHz, D_2O) spectrum of $\mathbf{R_b}H_2$·4TFA

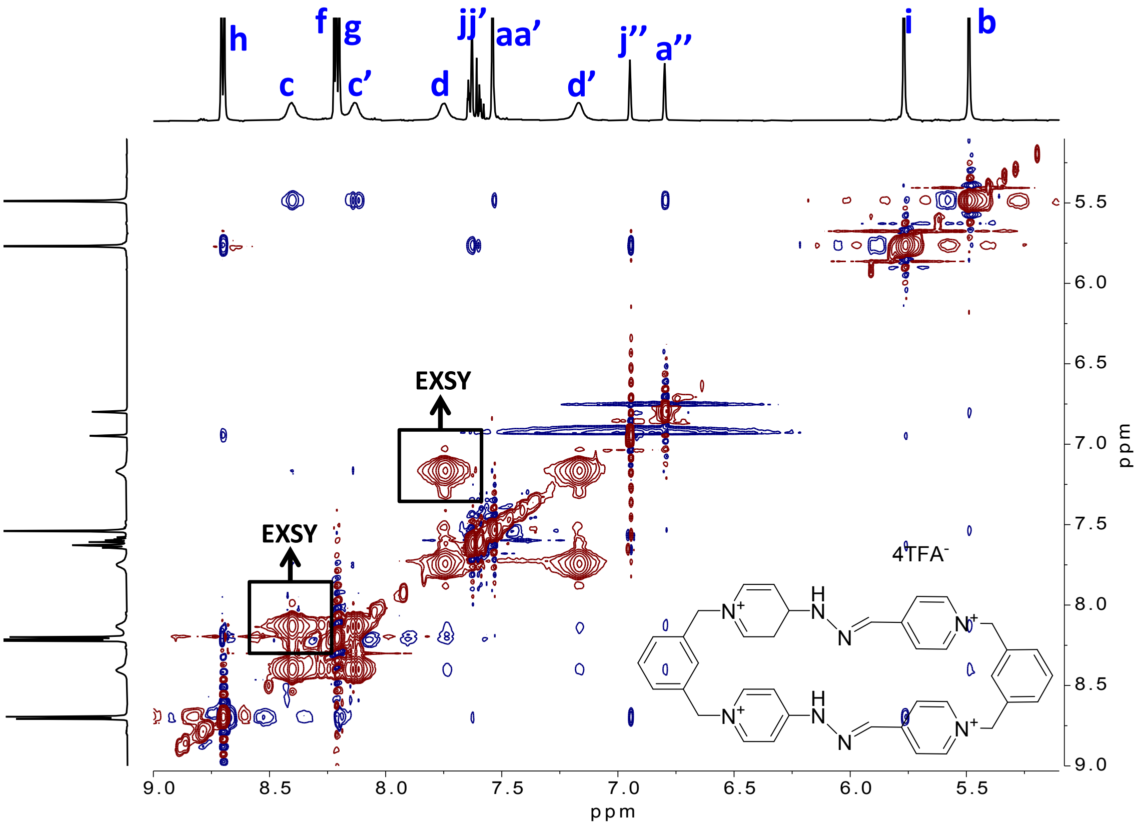

Figure S 66. ^{1}H-^{1}H NOESY (500 MHz, D_2O) spectrum of $\mathbf{R_b}H_2$·4TFA

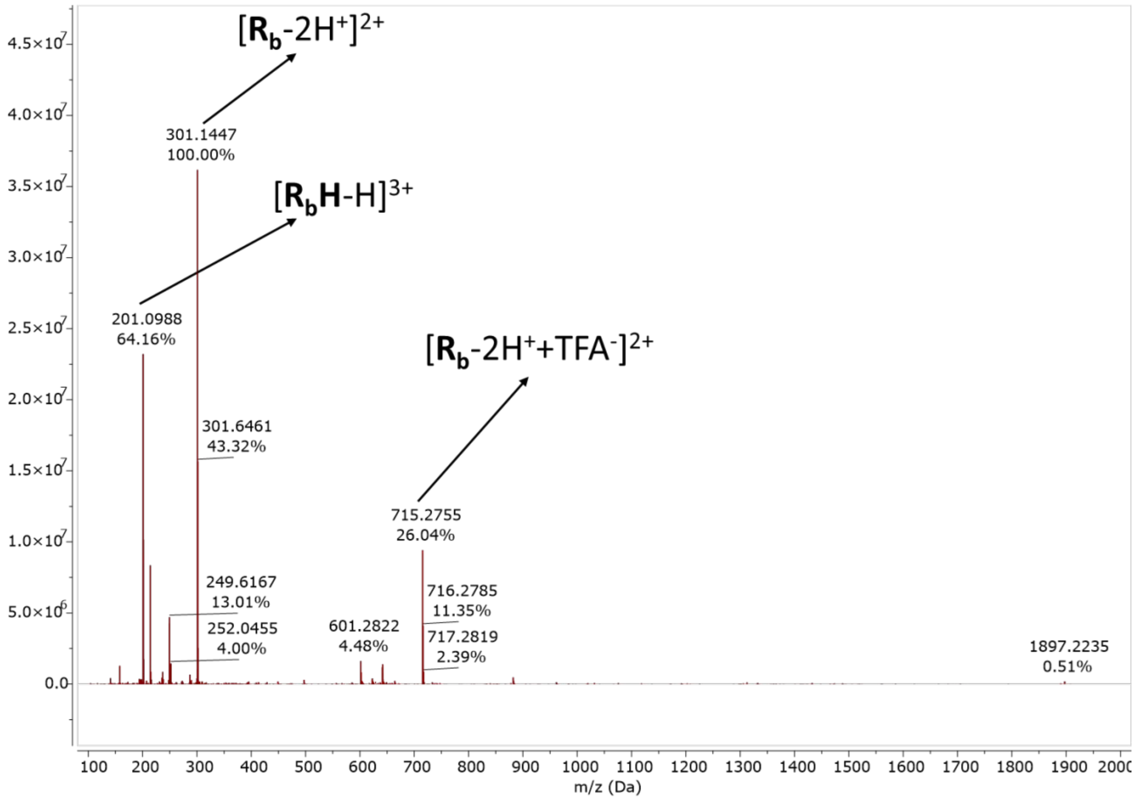

Figure S 67. HR ESI-MS spectrum of $\mathbf{R_b}H_2$·4TFA

^{1}H NMR (500 MHz, CD_3CN) δ (ppm): 8.65 (d, J = 6.9 Hz, 4H), 8.57 (s, 2H), 8.21 (d, J = 6.9 Hz, 2H), 8.12 (d, J = 6.9 Hz, 4H), 8.12 (d, J = 7.0 Hz, 2H), 7.73 (d, J = 7.4, 2H), 7.63 (m, 3H), 7.55 (s, 3H), 7.52 (d, J = 7.4, 2H), 7.08 (s, 1H), 6.95 (s, 1H), 5.69 (s, 4H), 5.41 (s, 4H). **^{13}C{^{1}H} NMR** (101 MHz, CD_3CN) δ (ppm): 155.9 (C), 150.8 (C), 145.4 (CH), 144.6 (CH), 142.1 (CH), 136,4 (C), 135.5 (C), 131.2 (CH), 130.2 (CH), 125.4 (CH), 119.7 (CH), 111.8 (C), 109.8 (CH), 64.0 (CH_2), 61.3 (CH_2).

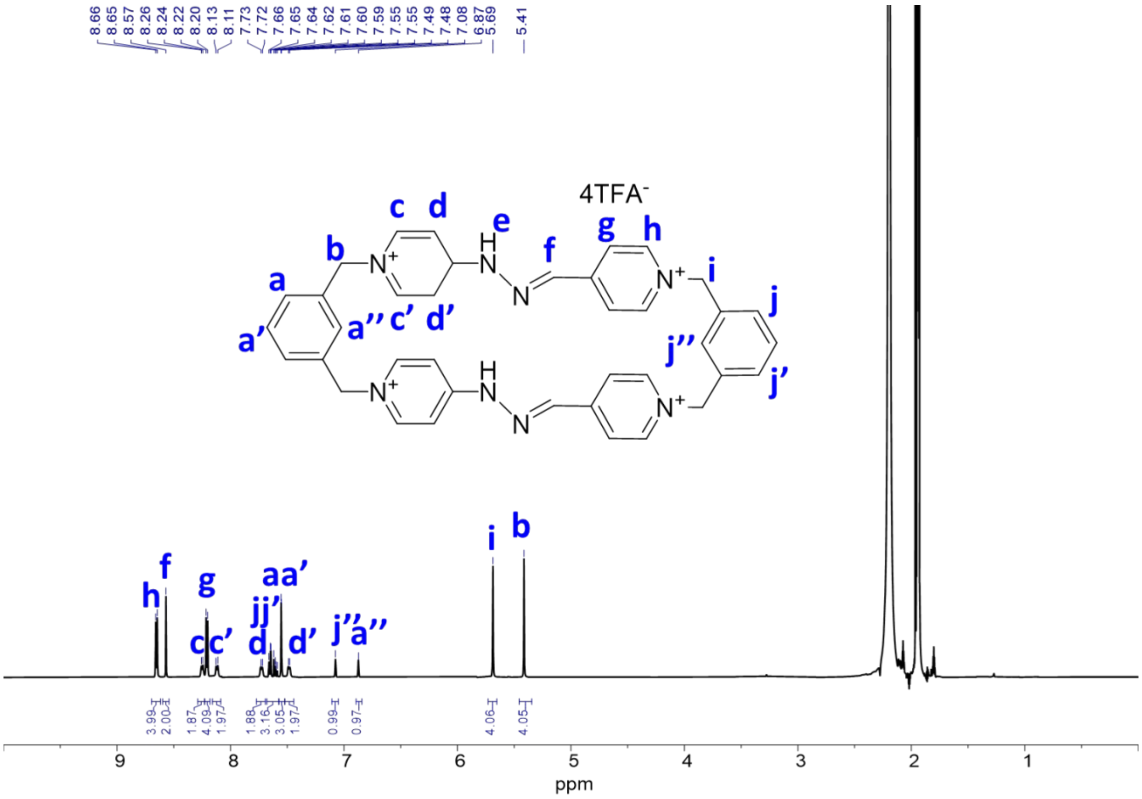

Figure S68. ^{1}H NMR (500 MHz, CD_3CN) spectrum of $\mathbf{R_b}H_2$·4TFA

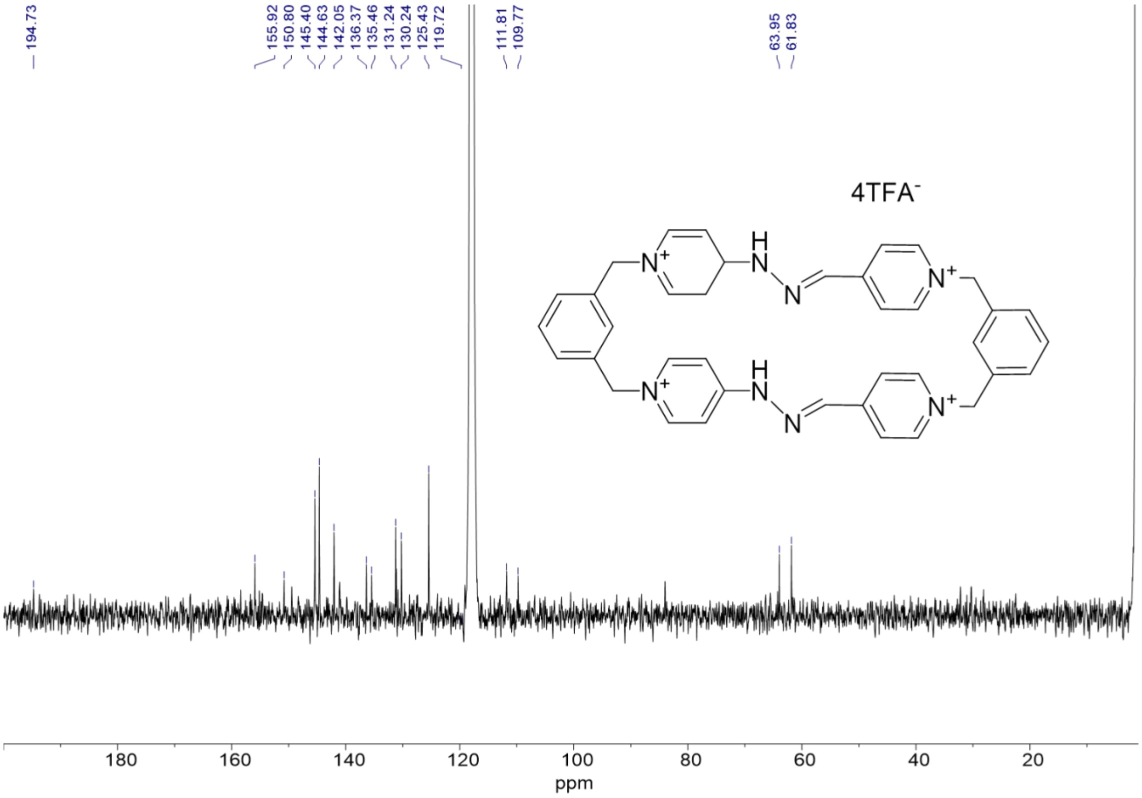

Figure S 69. $^{13}C\{^{1}H\}$ NMR (101 MHz, CD_3CN) spectrum of $\mathbf{R_b}H_2$·4TFA

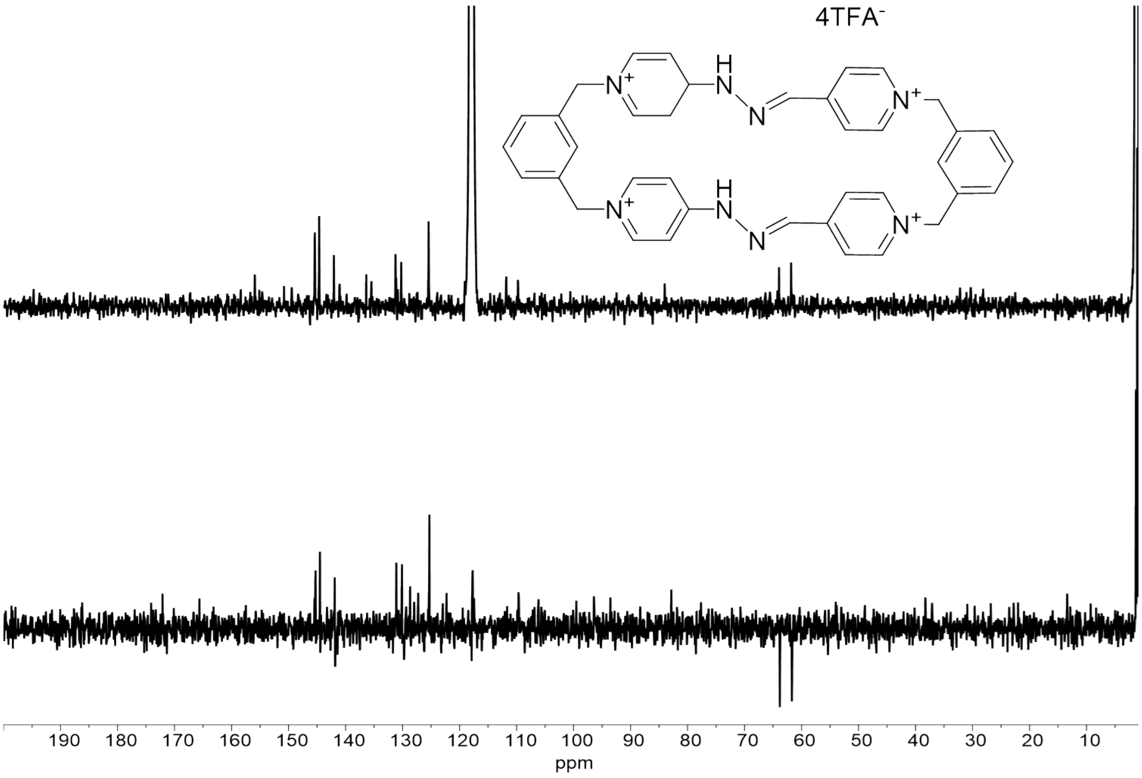

Figure S 70. $^{13}C\{^1H\}$ NMR (101 MHz, CD_3CN) spectrum (top) and DEPT-135 (101 MHz, CD_3CN) spectrum (bottom) of $\mathbf{R_b}H_2$·4TFA

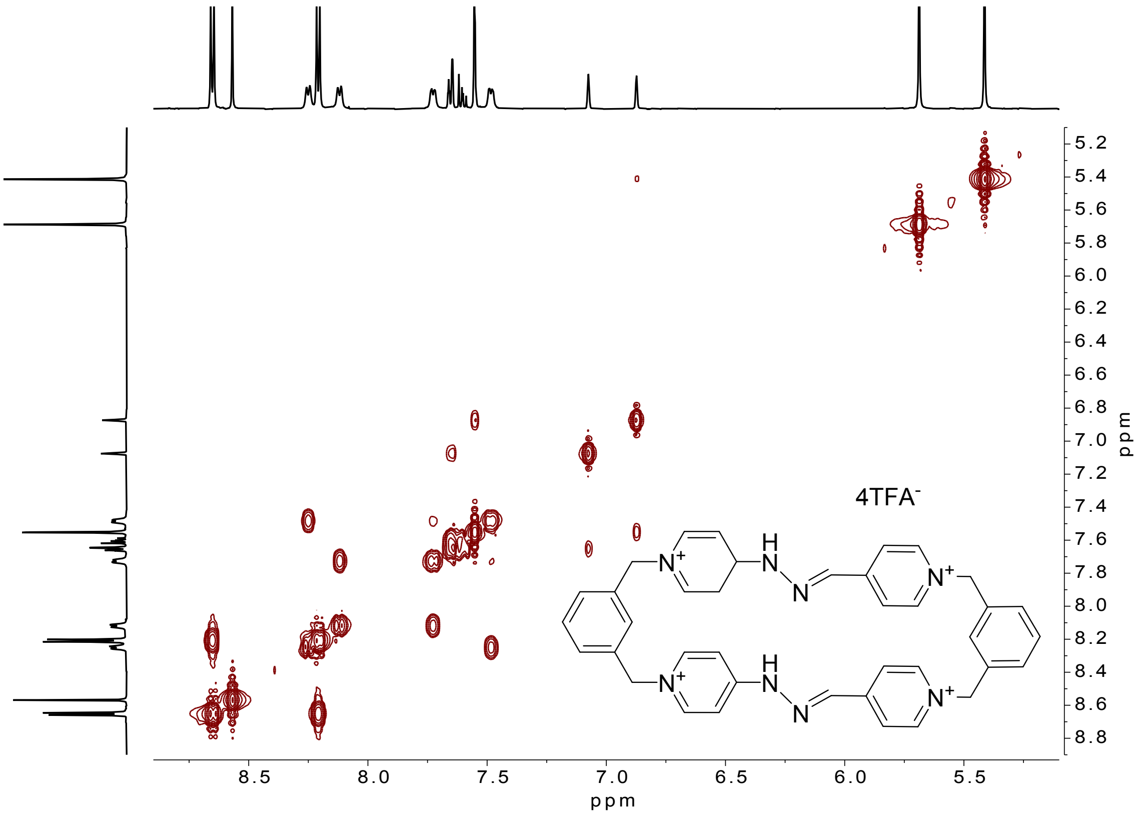

Figure S 71. ^1H-^1H COSY (500 MHz, CD_3CN) spectrum of $\mathbf{R_b}H_2$·4TFA

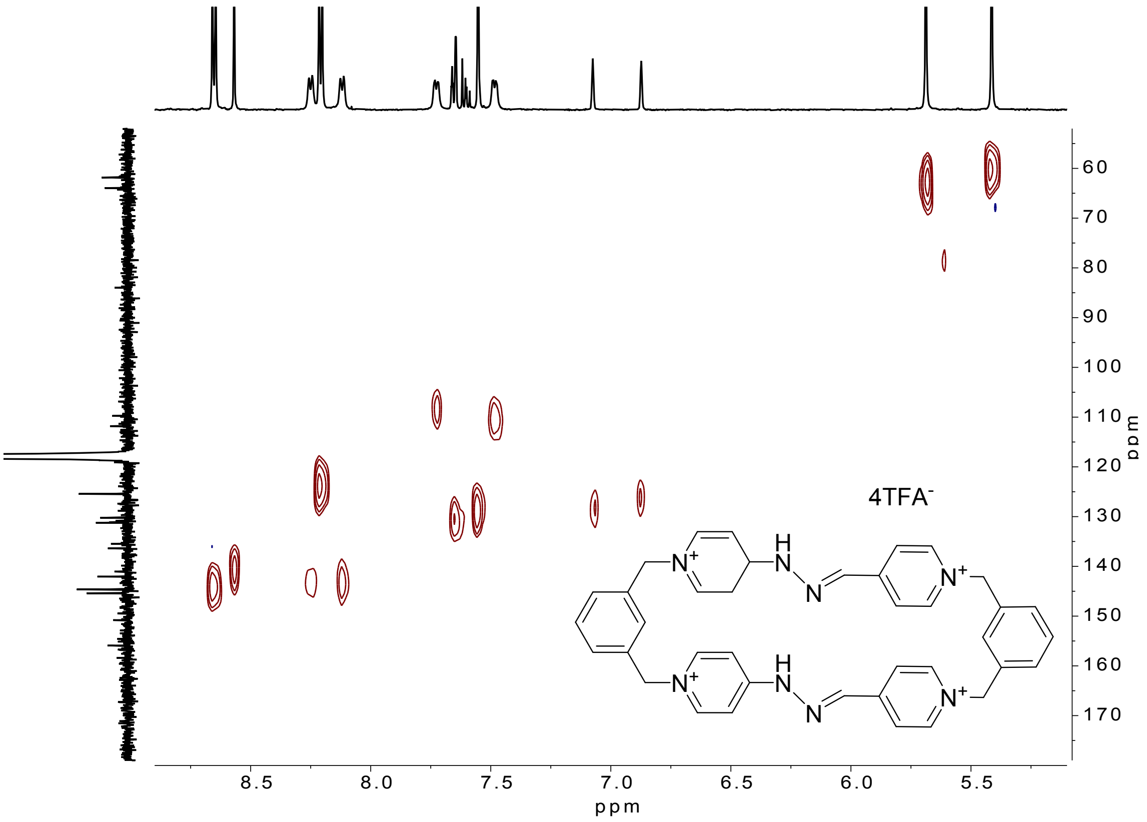

Figure S 72. ^{1}H-^{13}C HSQC (500 MHz/101 MHz, CD_3CN) spectrum of $\mathbf{R_b}H_2$

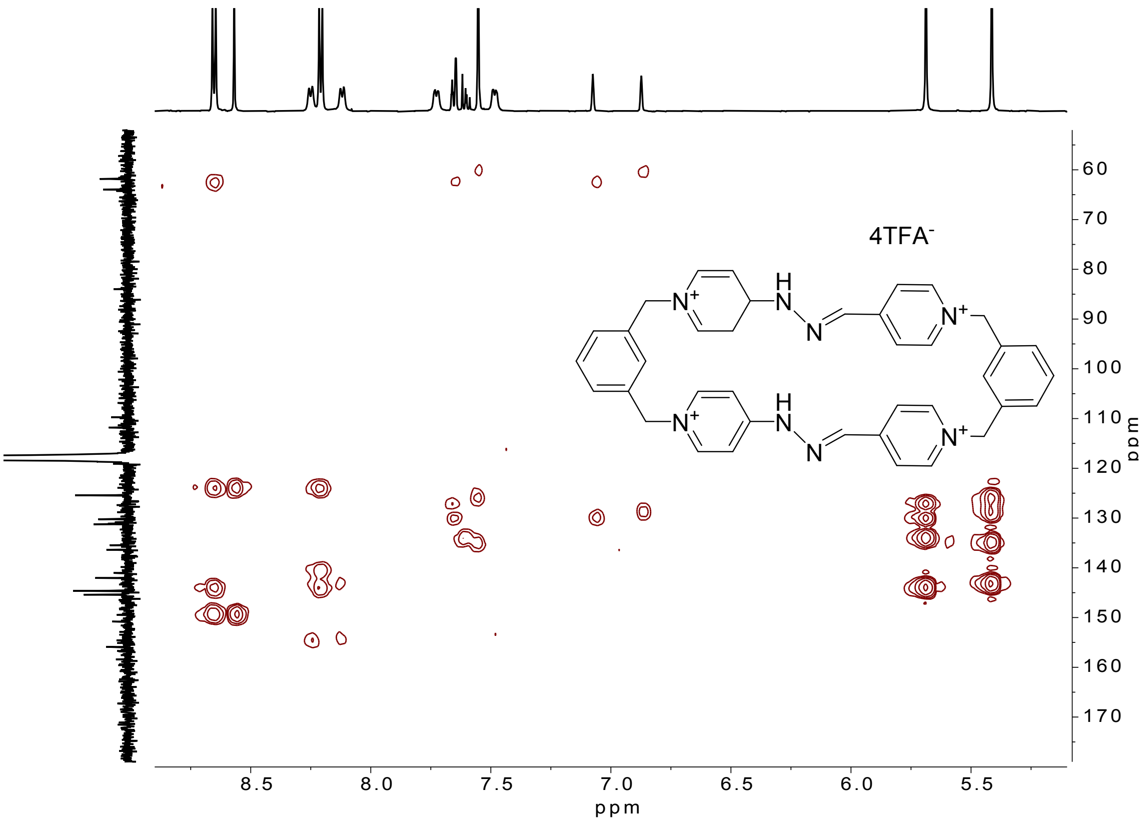

Figure S 73. ^{1}H-^{13}C HMBC (500 MHz/101 MHz, CD_3CN) spectrum of $\mathbf{R_b}H_2 \cdot 4TFA$

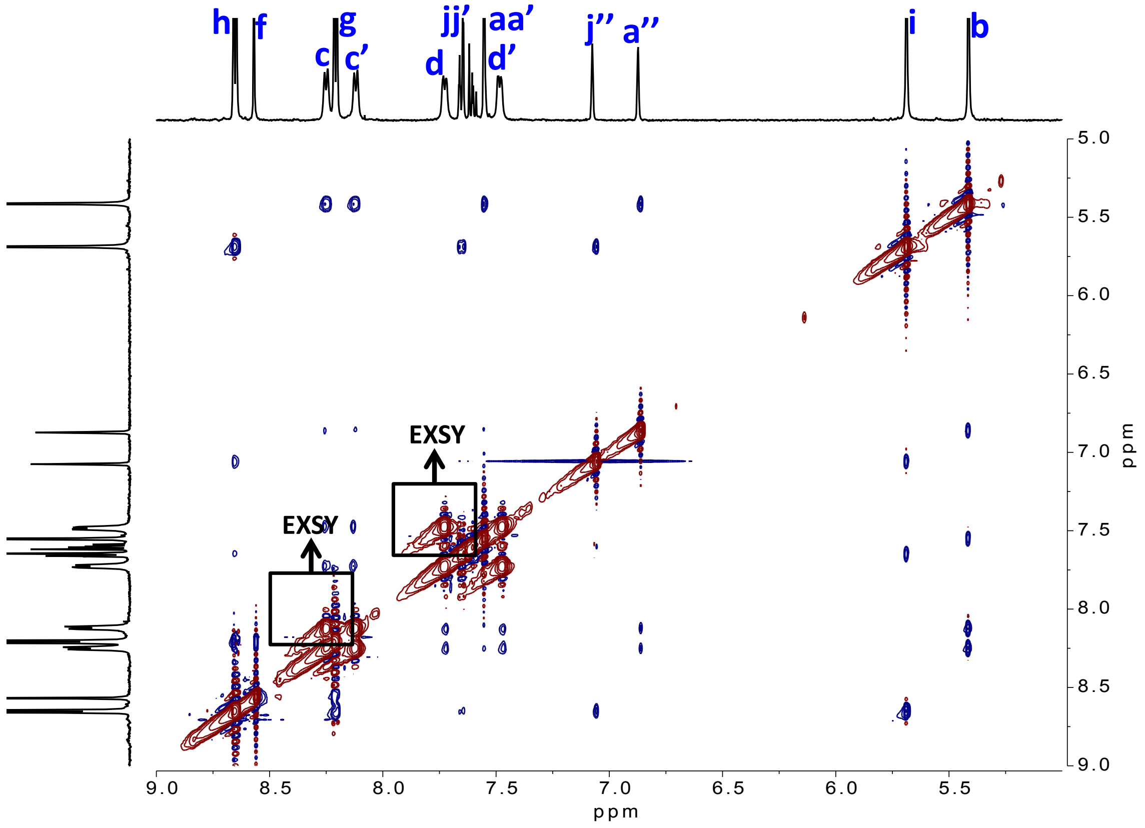

Figure S 74. ^1H-^1H NOESY (500 MHz, CD_3CN) spectrum of $\mathbf{R_b}H_2$·4TFA

1.2.10. Synthesis and characterization data of R_cH_2·4TFA

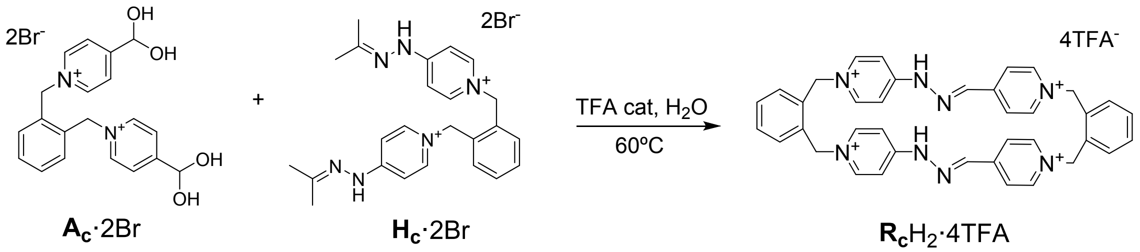

A$_c$·2Br (1.83 g, 3.56 mmol, 1 eq), and **H**$_c$·2Br (2.00 g, 3.56 mmol, 1 eq) were dissolved in a solution of 1.4 mL of water and 27.4 µL (10 mol%) of trifluoroacetic acid (divided into three 500 mL round bottom flasks). The mixture is heated and stirred at 60°C for 20 h using a magnetic hot plate stirrer. After checking the completion of the reaction by NMR, the reaction is cooled and an excess of KPF_6 was added until no further precipitation was observed. The mixture was left stirring at room temperature for 30 min. The obtaining reddish solid is then filtered under vacuum and washed with water (3×50 mL) and diethyl ether (3×50 mL). 2.87 g of **R**$_c$H$_2$·4PF$_6$ were obtained with a purity of 72%, achieving a yield of 68%. The product was purified by reverse-phase semipreparative HPLC (RP, H_2O + 0.1% TFA/CH_3CN + 0.1% TFA = 5/95, flow rate = 0.3 mL/min, I = 220 nm, t_R = 8.4 min), yielding a yellowish solid **R**$_c$H$_2$·4TFA (1.59 g, 66 %), with an overall reaction yield of 45%.

mp 312.3 – 313.7°C (decomposition). **^{1}H NMR** (500 MHz, D_2O, 90°C) δ (ppm): 9.16 (d, J = 6.5 Hz, 2H), 8.76 (s, 2H), 8.73 (d, J = 7.1 Hz, 3H), 8.69 (d, J = 6.6 Hz, 4H), 8.61 (s, 4H), 8.53 (s, 4H), 8.31 (s, 2H), 7.68 (s, 2H), 6.70 (s, 4H), 6.39 (s, 4H). **^{13}C{^{1}H} NMR** (126 MHz, D_2O, 90°C) δ (ppm): 154.6 (C), 150.3 (C), 144.6 (CH), 144.2 (C), 143.6 (C), 141.7 (CH), 136.5 (CH), 136.0 (CH), 133.4 (CH), 132.8 (CH), 132.0 (C), 131.3 (C), 125.7 (CH), 111.8 (C), 110.4 (C), 62.6 (CH_2), 60.5 (CH_2). **HRMS** (**ESI**)m/z: [**R**$_c$-2H$^+$]$^{2+}$ Calcd for $C_{38}H_{34}N_8^{2+}$ 301.1448; Found 301.1448; and [**R**$_c$-2H$^+$+TFA$^-$]$^+$ Calcd for $C_{40}H_{34}F_3N_8O_2^+$ 715.2751; Found, 715.2757.

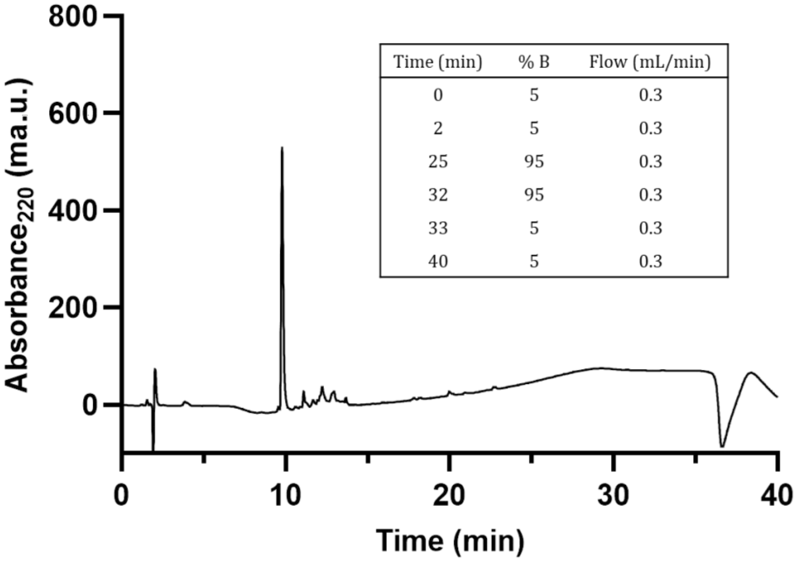

Figure S 75. HPLC chromatogram (220 nm) of $\mathbf{R_c}H_2{\cdot}4PF_6$ at t_R = 9.8 min. *Inset*. Elution conditions

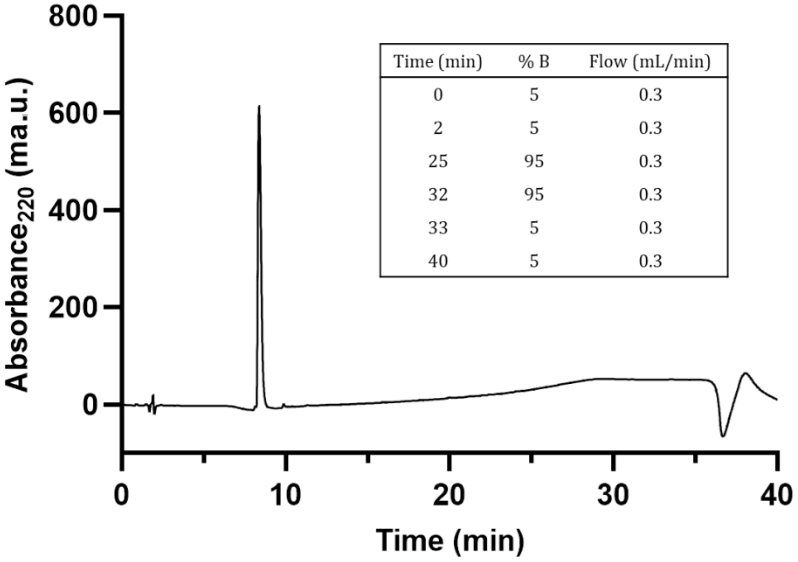

Figure S 76. HPLC chromatogram (220 nm) of purified $\mathbf{R_c}H_2{\cdot}4TFA$ at t_R = 8.4 min. *Inset*. Elution conditions

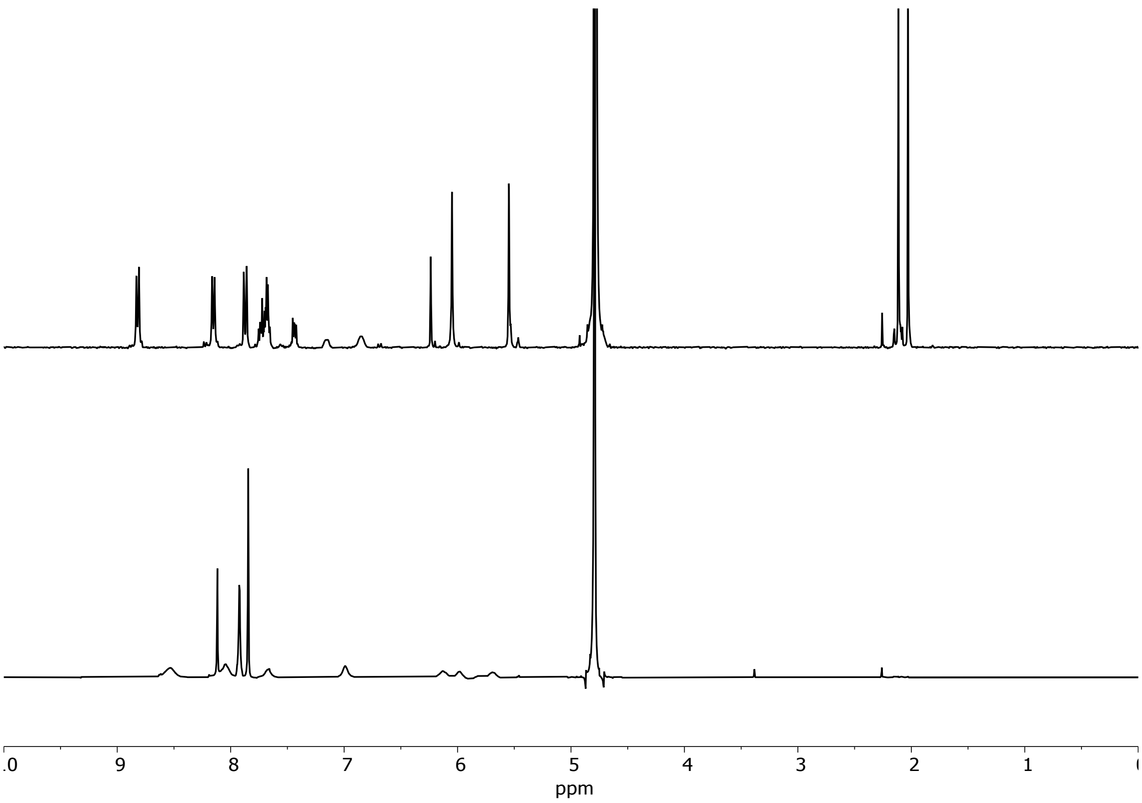

Figure S 77. ^{1}H NMR (300 MHz, D_2O) stacked spectra of: (top) equimolar 2.5 mM mixture of $\mathbf{A_c}$·2Br and $\mathbf{H_c}$·2Br at t = 0; (bottom) same mixture after 24 hours at 60°C with TFA-d_3 (10% molar).

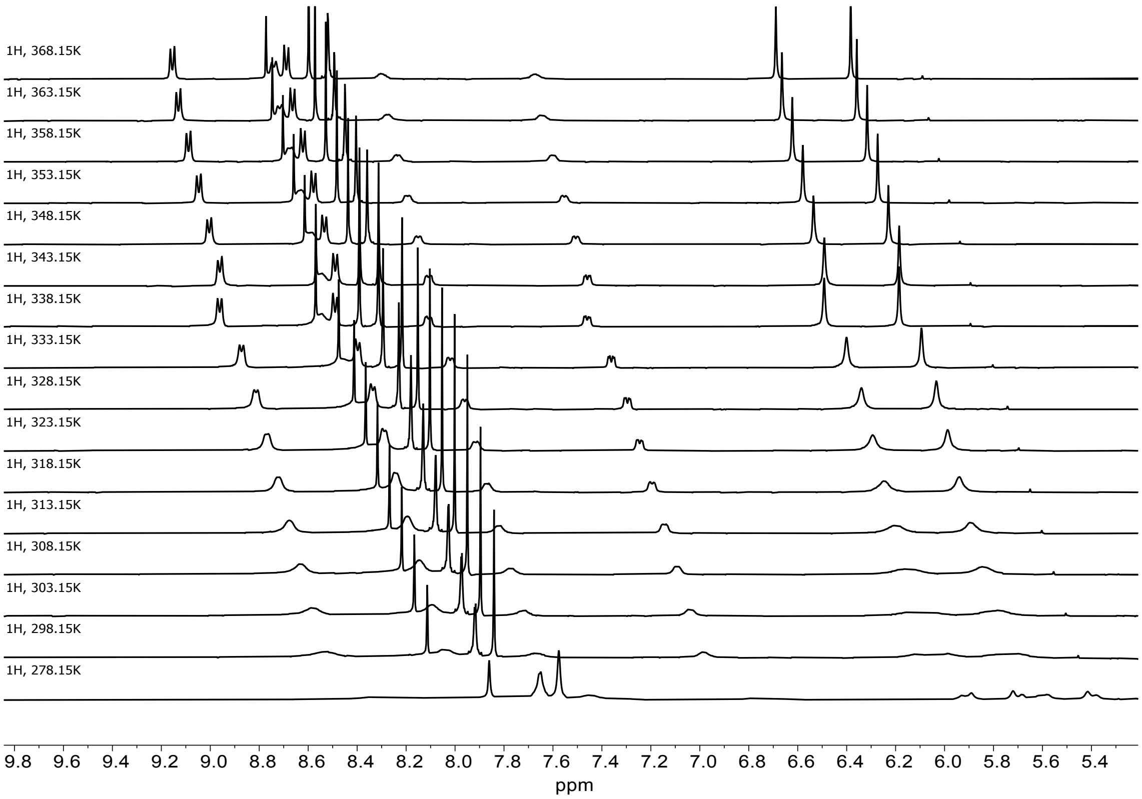

Figure S 78. VT ^{1}H NMR (400 MHz, D_2O) stacked spectra of $\mathbf{R_c}H_2$·4TFA

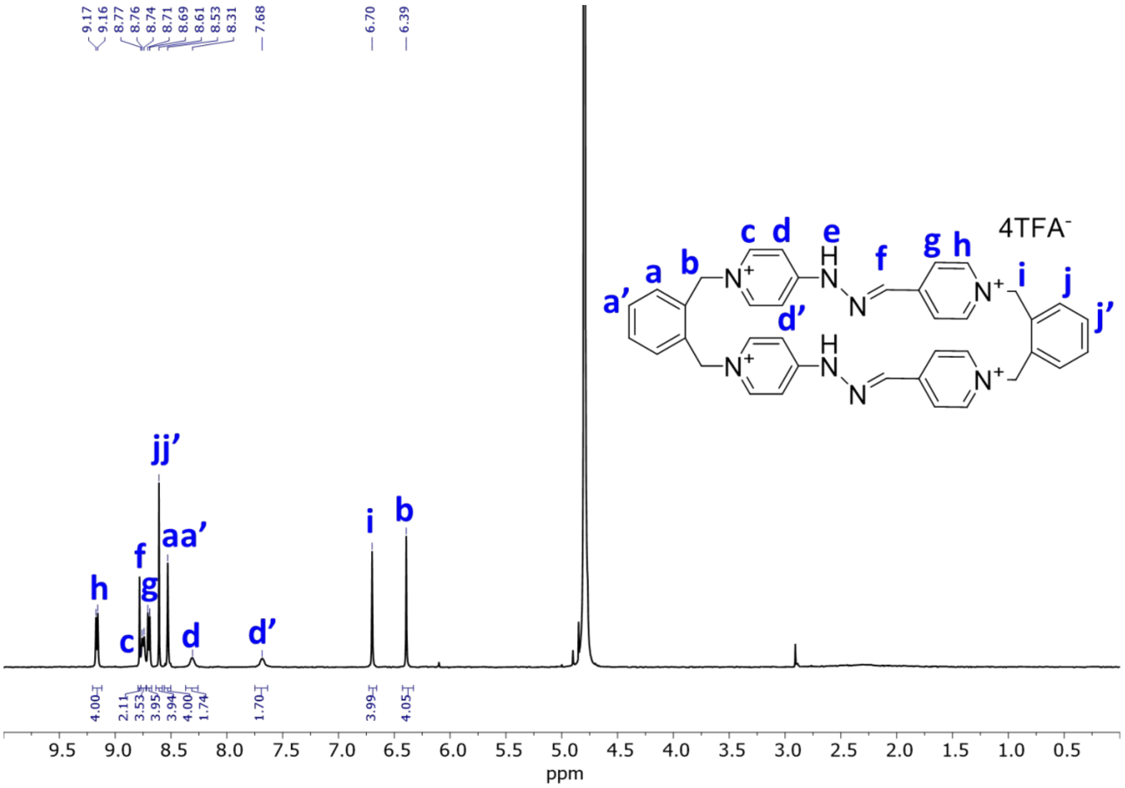

Figure S 79. ^{1}H NMR (500 MHz, D_2O, 90°C) spectrum of $\mathbf{R_c}H_2$·4TFA

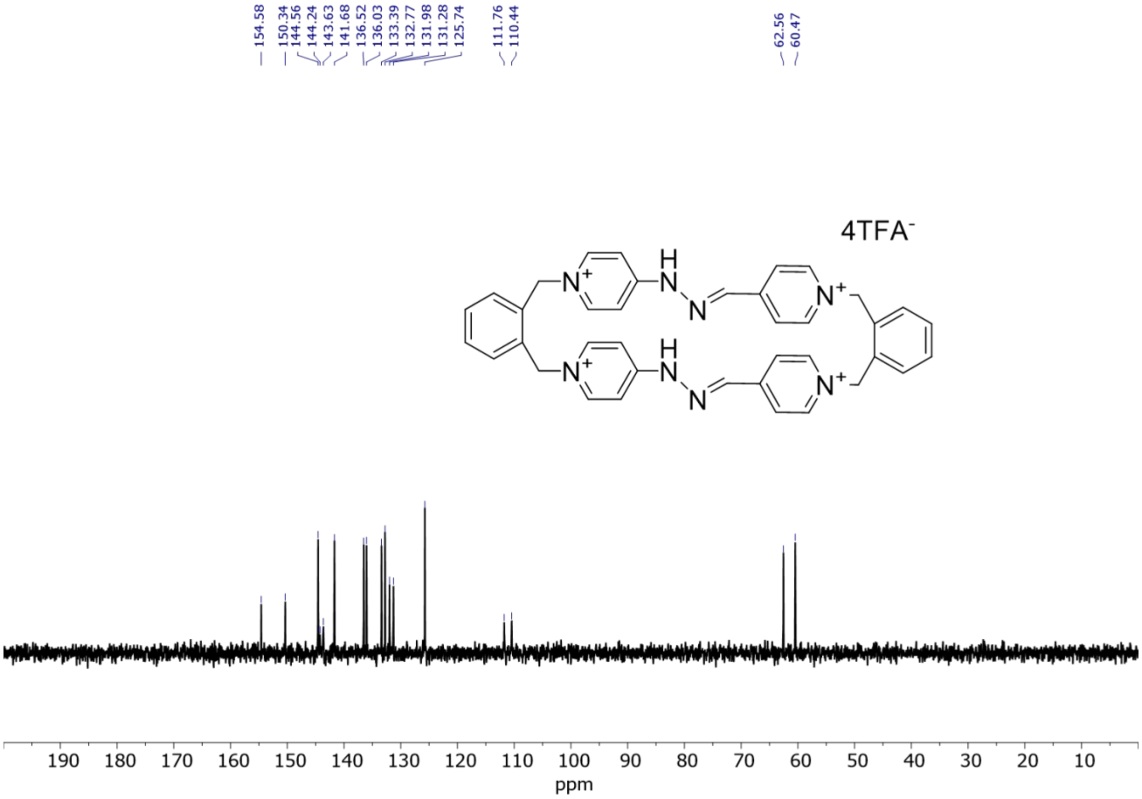

Figure S 80. $^{13}C\{^{1}H\}$ NMR (126 MHz, D_2O, 90°C) spectrum (top) of $\mathbf{R_c}H_2$·4TFA

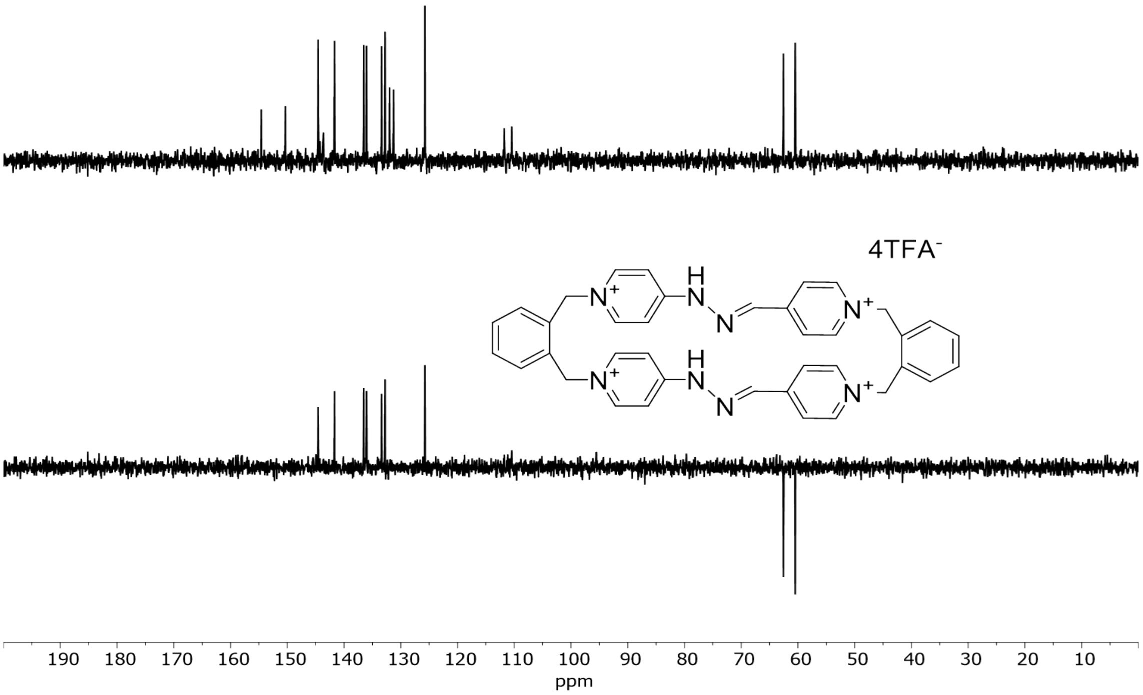

Figure S 81. $^{13}C\{^{1}H\}$ NMR (126 MHz, D_2O, 90°C) spectrum (top) and DEPT-135 (126 MHz, D_2O, 90°C) spectrum (bottom) of $\mathbf{R_c}H_2{\cdot}4TFA$

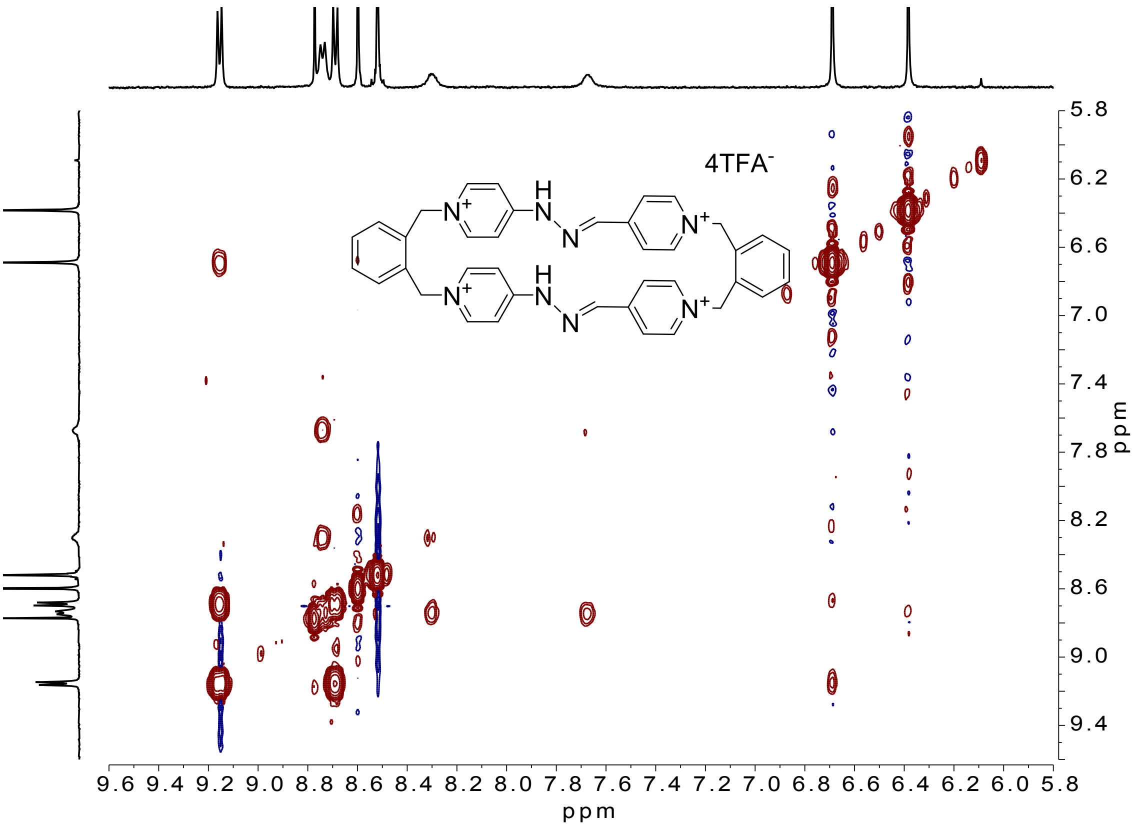

Figure S 82. ^{1}H-^{1}H COSY (500 MHz, D_2O, 90°C) spectrum of $\mathbf{R_c}H_2{\cdot}4TFA$

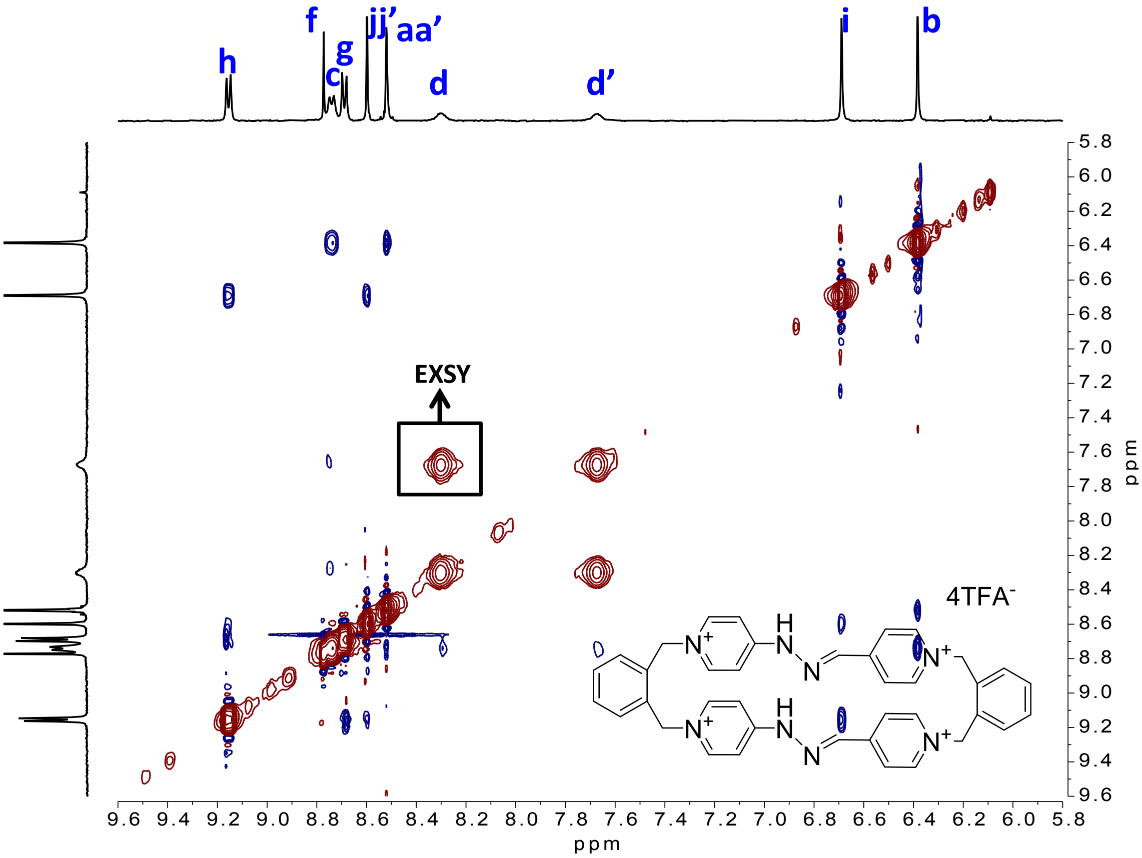

Figure S 83. ^{1}H-^{1}H NOESY (500 MHz, D_2O, 90°C) spectrum of $\mathbf{R_c}H_2$·4TFA

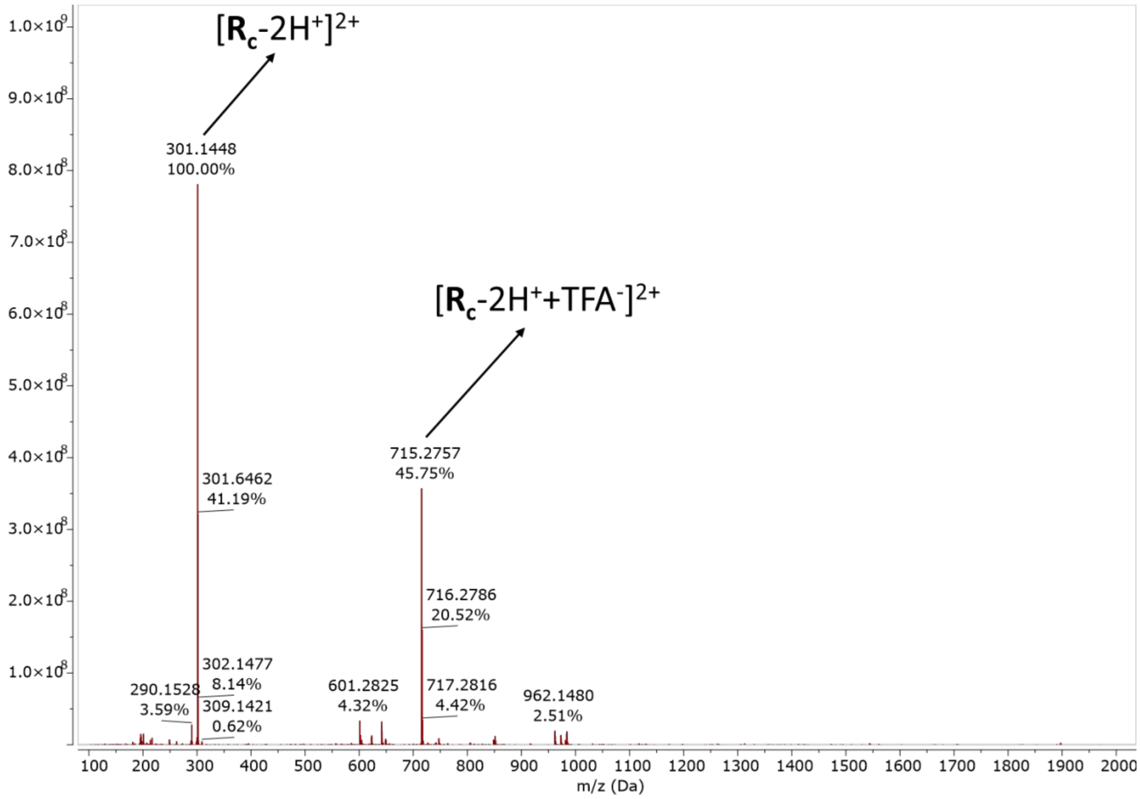

Figure S 84. HR ESI-MS spectrum of $\mathbf{R_c}H_2$·4TFA

1.2.11. Synthesis and characterization data of $\mathbf{R_dH_2{\cdot}4TFA}$

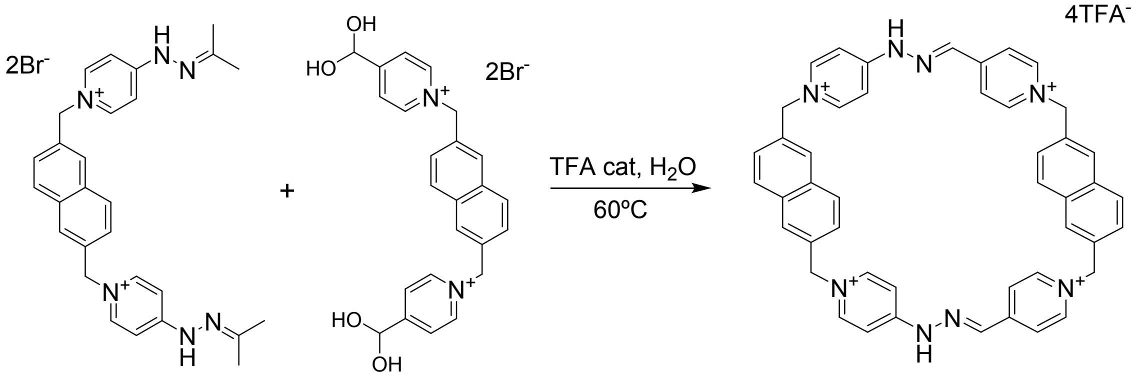

Equimolar amounts of $\mathbf{H_d}$·2Br (1.92 g, 3.13 mmol, 1 eq) and $\mathbf{A_d}$·2Br (0.1.77 g, 3.13 mmol, 1 eq) were dissolved in 1.2 L of water with 24.0 µL (10 mol%) of trifluoroacetic acid (divided into three 500 mL round bottom flasks), and it was heated at 60°C h using a magnetic hot plate stirrer for 24 h. After checking the completion of the reaction by NMR, the reaction is cooled and an excess of KPF_6 was added until no further precipitation was observed. The mixture was left stirring at room temperature for 30 min. The obtaining reddish solid is then filtered under vacuum and washed with water (3×50 mL) and diethyl ether (3×50 mL). 1.99 g of $\mathbf{R_d}H_2{\cdot}4PF_6$ were obtained with a purity of 70%, achieving a yield of 55%. The product was purified by reverse-phase semipreparative HPLC (RP, H_2O + 0.1% TFA/CH_3CN + 0.1% TFA = 5/95, flow rate = 0.3 mL/min, I = 220 nm, t_R = 12.1 min), yielding $\mathbf{R_d}H_2{\cdot}4TFA$ as a yellowish solid (0.80 g, 41 %), with an overall reaction yield of 22%.

mp 316.9 – 318.3°C (decomposition). **^{1}H RMN** (500 MHz, D_2O), δ (ppm): 8.77 (d, J = 6.7 Hz, 4H), 8.64 (bs, 2H), 8.24 (s, 2H), 8.22 (d, J = 6.6 Hz, 4H), 8.11 (d, J = 1.7 Hz, 2H), 8.03 (s, 2H), 8.01 (d, J = 8.5 Hz, 2H), 7.98 (d, J = 8.5 Hz, 2H), 7.96 (bs, 2H), 7.67 (bs, 2H), 7.54 (d, J = 1.7 Hz, 2 H), 7.49 (d, J = 1.7 Hz, 2H), 7.23 (bs, 2H), 5.92 (s, 4H), 5.64 (s, 4H). **^{13}C{^{1}H} NMR** (126 MHz, D_2O), δ (ppm): 154.5 (C), 149.9 (C), 144.2 (CH), 142.8 (C), 140.6 (CH), 133.1 (C), 132.9 (C), 132.2 (C), 131.5 (C), 129.9 (CH), 129.8 (CH), 129.7 (CH), 129.3 (CH), 127.0 (CH), 126.8 (CH), 124.6 (CH), 117.4 (C), 115.1 (C), 64.1 (CH_2), 62.0 (CH_2). **HRMS** (**ESI**) m/z: $[\mathbf{R_d}]^{2+}$ Calcd for $C_{46}H_{38}N_8^{2+}$ 351.1604; Found 351.1604; and $[\mathbf{R_d}\text{-}2H^{+}+TFA^{-}]^{+}$ Calcd for $C_{46}H_{34}F_3N_8O_2^{+}$ 815.3065; Found 815.3069.

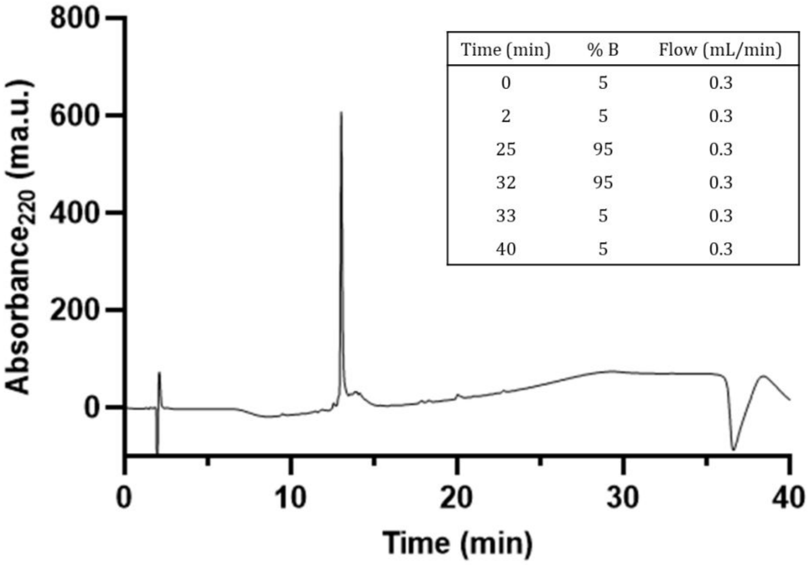

Time (min)	% B	Flow (mL/min)
0	5	0.3
2	5	0.3
25	95	0.3
32	95	0.3
33	5	0.3
40	5	0.3

Figure S 85. HPLC chromatogram (220 nm) of $\mathbf{R_d}H_2$·4PF_6 at t_R = 13.1 min. *Inset*. Elution condition

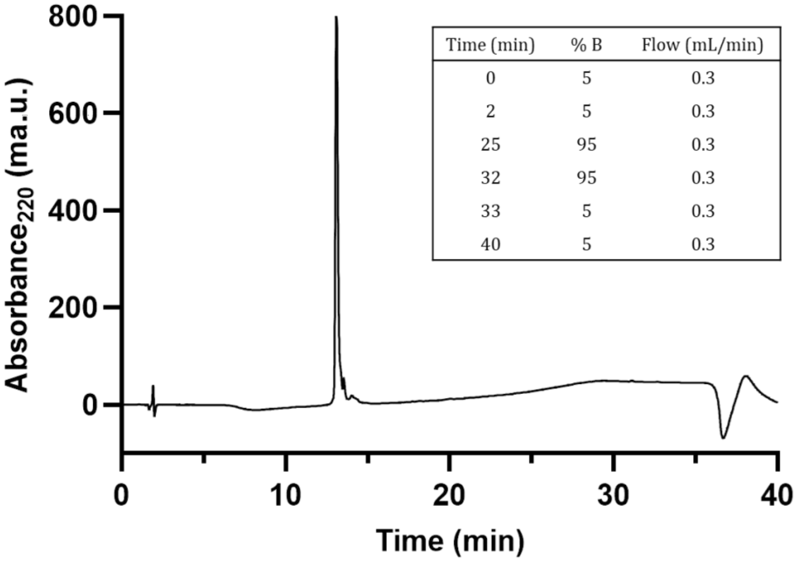

Time (min)	% B	Flow (mL/min)
0	5	0.3
2	5	0.3
25	95	0.3
32	95	0.3
33	5	0.3
40	5	0.3

Figure S 86. HPLC chromatogram (220 nm) of purified $\mathbf{R_d}H_2$·4TFA at t_R = 12.1 min. *Inset*. Elution conditions

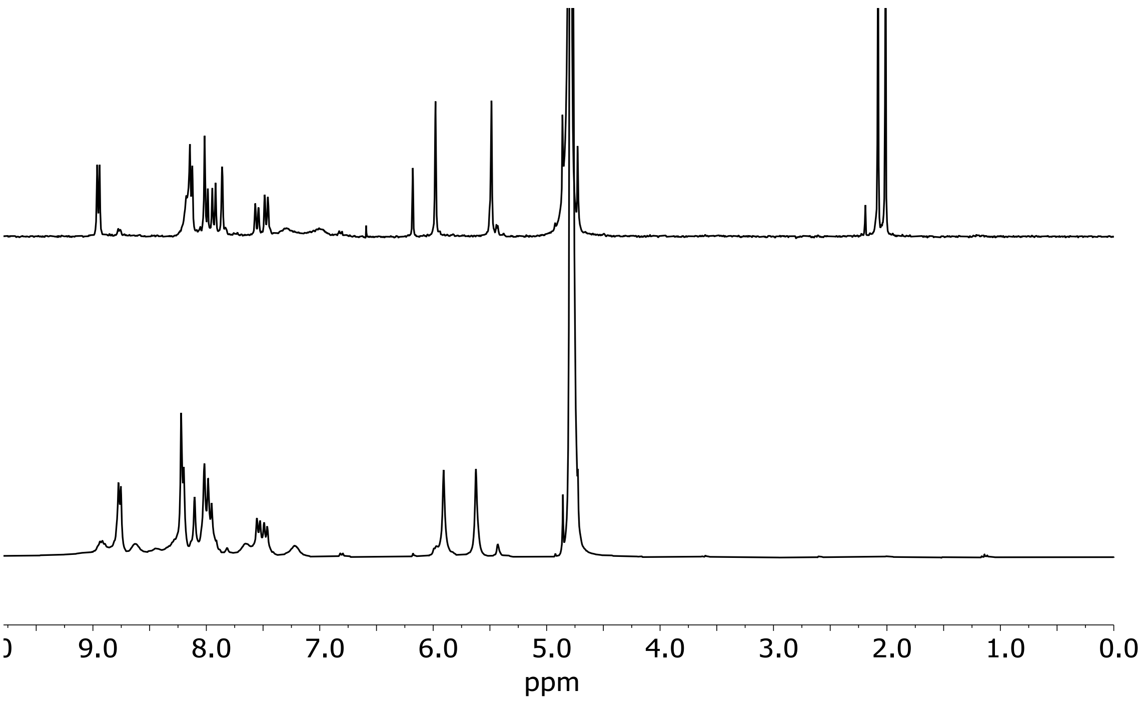

Figure S 87. ^{1}H NMR (300 MHz, D_2O) staked spectra of: (top) equimolar 2.5 mM mixture of $\mathbf{A_d}$·2Br and $\mathbf{H_d}$·2Br at t = 0; (bottom) same mixture after 24 hours at 60 °C with TFA-d_3 (10% molar)

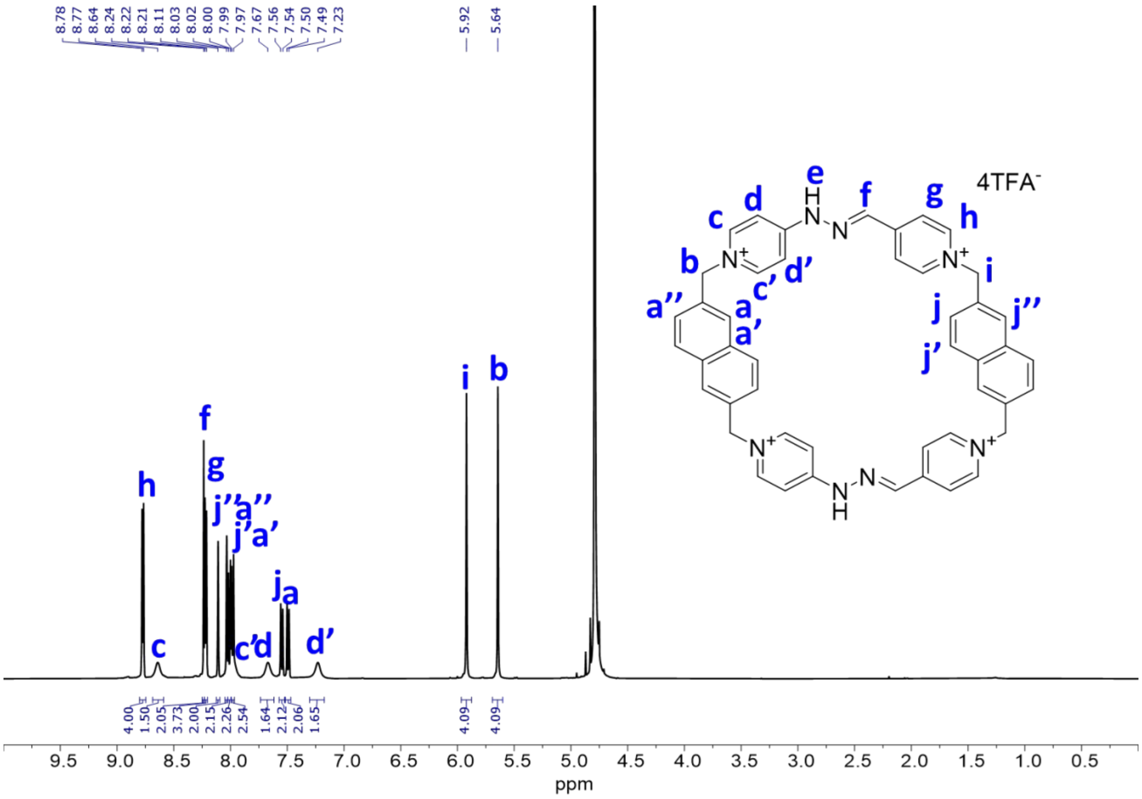

Figure S 88. ^{1}H NMR (500 MHz, D_2O) spectrum of $\mathbf{R_d}H_2$·4TFA

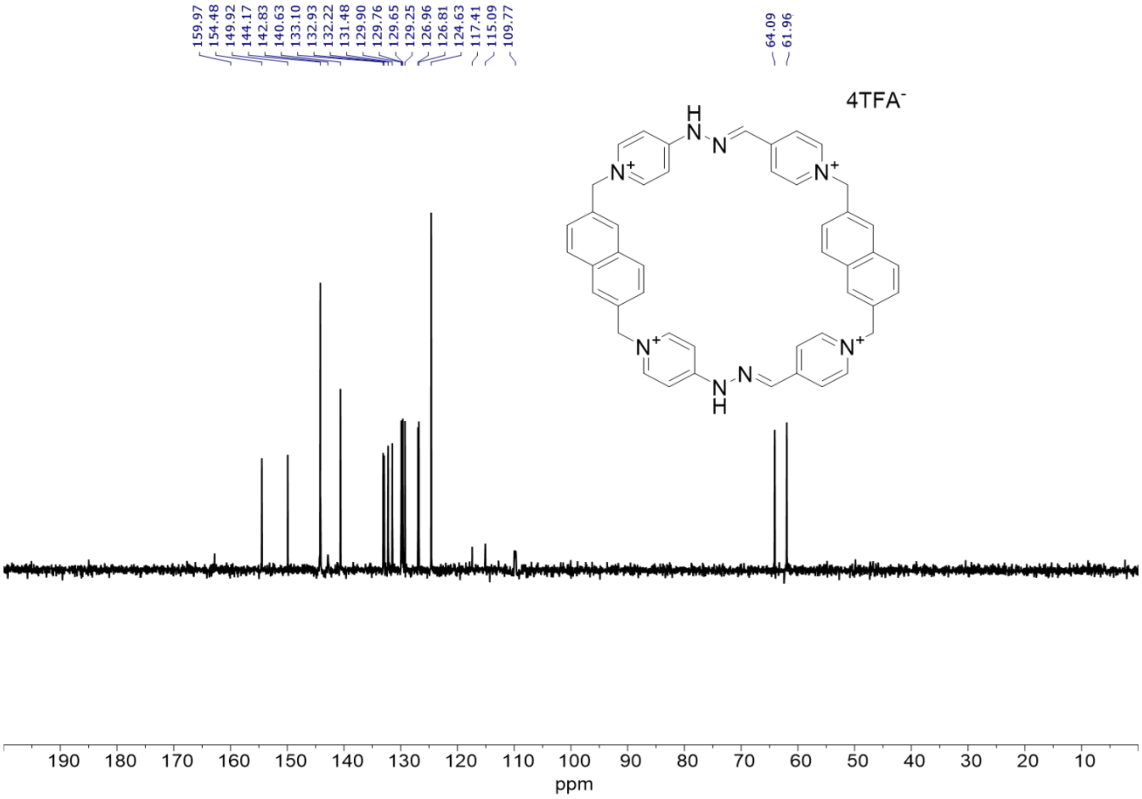

Figure S 89. $^{13}C\{1H\}$ NMR (126 MHz, D_2O) spectrum (top) of $\mathbf{R_d}H_2$·4TFA

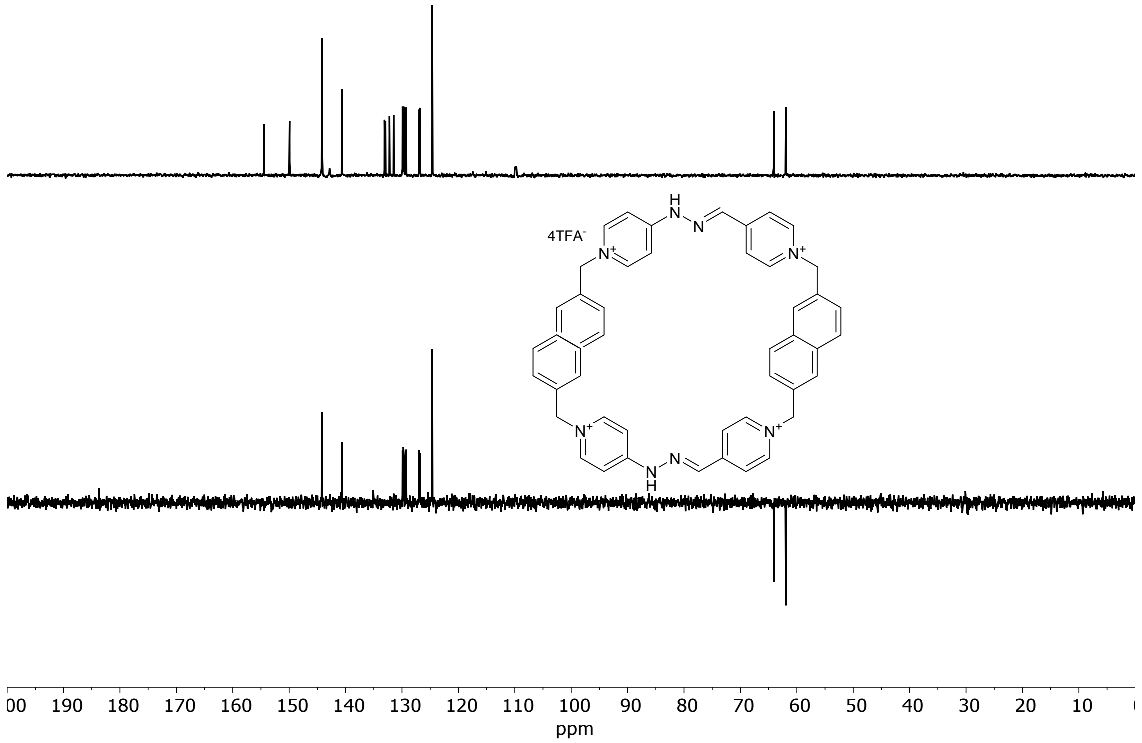

Figure S 90. $^{13}C\{^1H\}$ NMR (126 MHz, D_2O) spectrum (top) and DEPT-135 (126 MHz, D_2O) spectrum (bottom) of $\mathbf{R_d}H_2$·4TFA

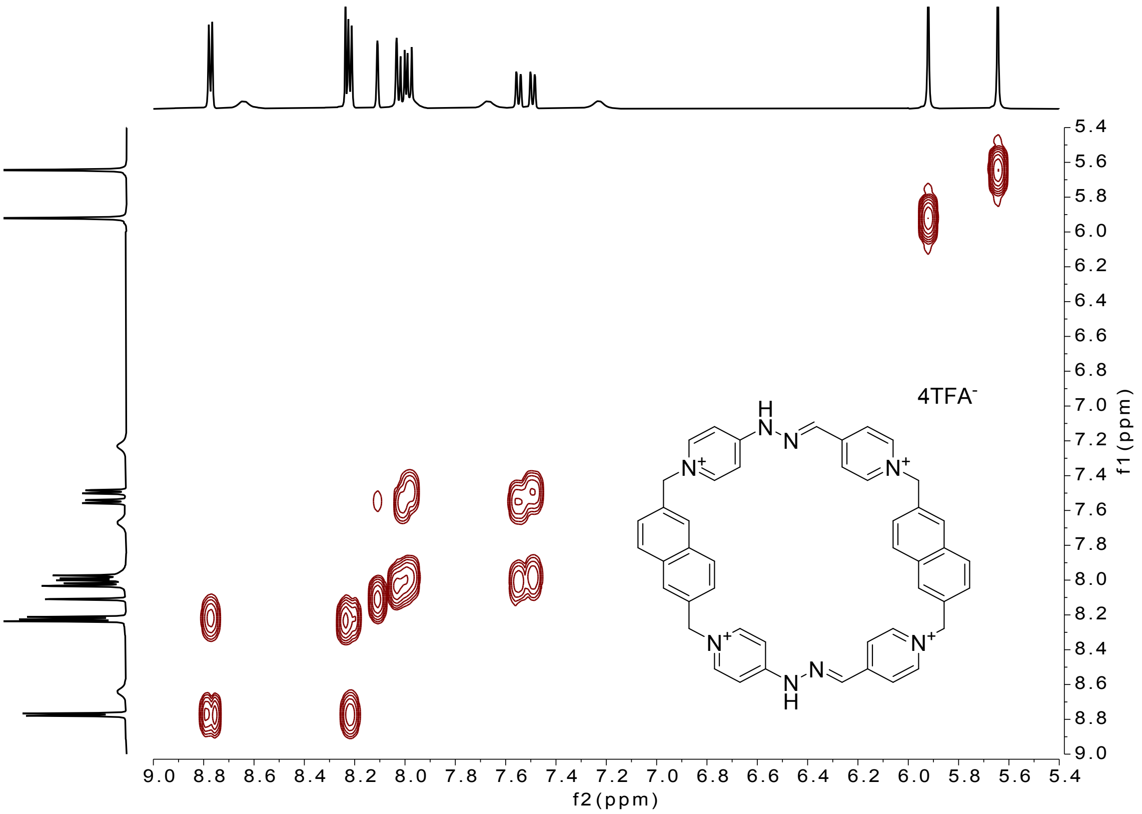

Figure S 91. ^1H-^1H COSY (500 MHz, D_2O) spectrum of $\mathbf{R_d}H_2$·4TFA

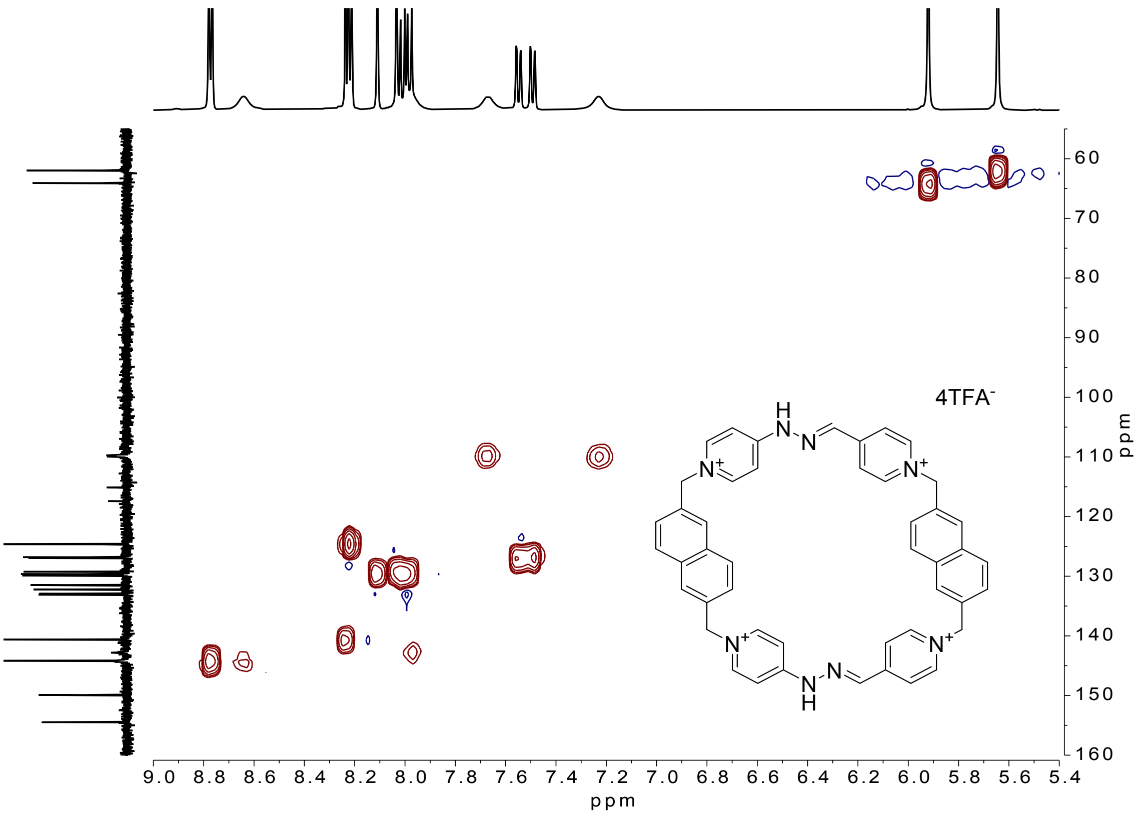

Figure S 92. ^1H-^{13}C HSQC (500 MHz/126 MHz, D_2O) spectrum of $\mathbf{R_d}H_2$·4TFA

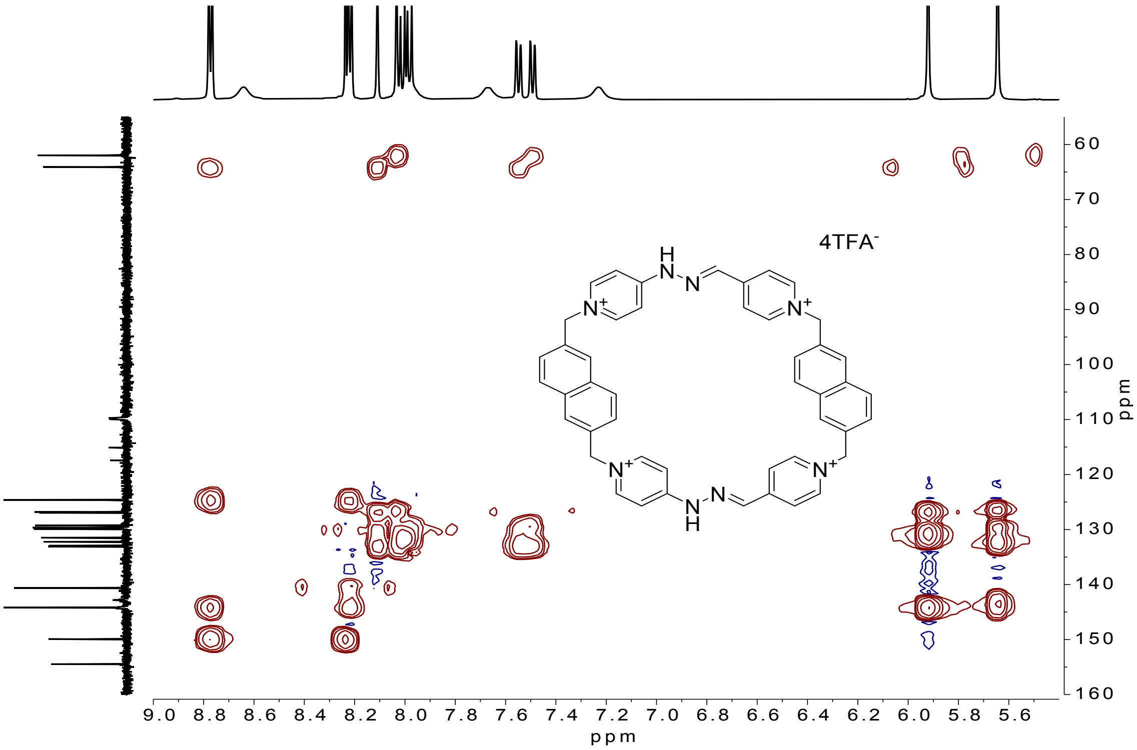

Figure S 93. ^{1}H-^{13}C HMBC (500 MHz/126 MHz, D_2O) spectrum of $\mathbf{R_d}H_2$·4TFA

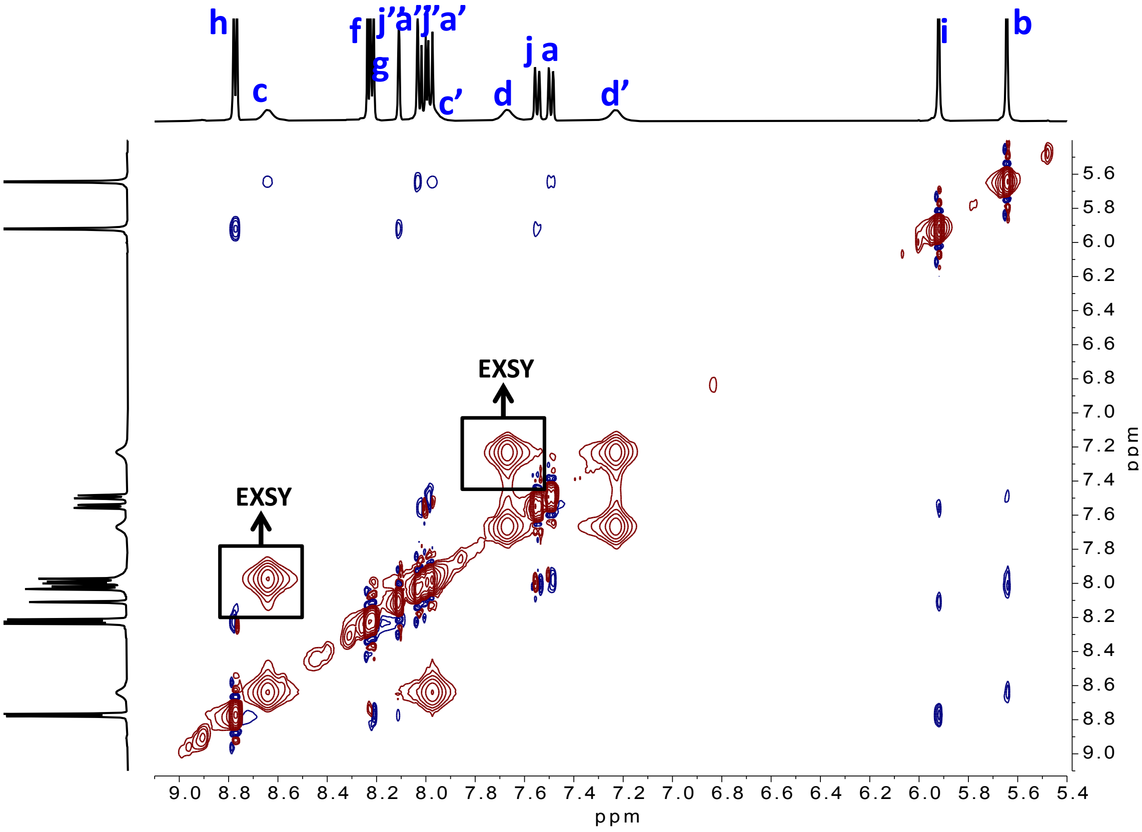

Figure S 94. ^{1}H-^{1}H NOESY (500 MHz, D_2O) spectrum of $\mathbf{R_d}H_2$·4TFA

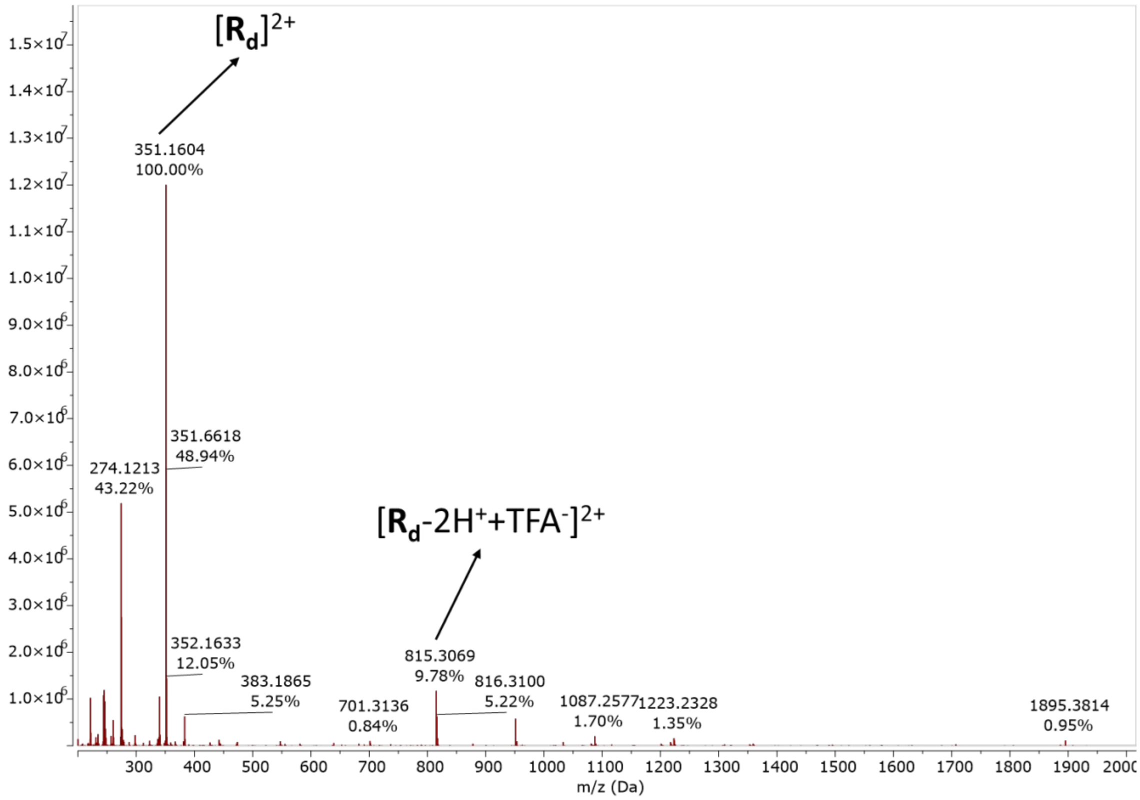

Figure S 95. HR ESI-MS spectrum of $\mathbf{R_d}H_2{\cdot}4TFA$

^{1}H RMN (500 MHz, CD_3CN), δ (ppm): 8.64 (d, J = 6.7 Hz, 4H), 8.62 (s, 2H), 8.50 (d, J = 7.0 Hz, 2H), 8.17 (d, J = 6.7 Hz, 4H), 8.07 (s, 2H), 7.98 (s, 2H), 7.95 (m, 4H), 7.87 (d, J = 7.2 Hz, 2 H), 7.60 (m, 4H), 7.53 (dd, J = 8.5, 1.7 Hz, 2H), 7.46 (dd, J = 8.6, 1.7 Hz, 2H), 5.81 (s, 4H), 5.53 (s, 4H). **^{13}C{^{1}H} NMR** (126 MHz, CD_3CN), δ (ppm): 160.6 (C), 160.3 (C), 156.2 (C), 151.2 (C), 145.1 (CH), 143.5 (CH), 141.8 (CH), 133.8, 133.7 (C), 133.6 (C), 132.8 (C), 130.5 (CH), 130.4 (CH), 130.3 (CH), 129.8 (CH), 127.7 (CH), 127.5 (CH), 125.4 (CH), 118.0 (CH), 116.3 (C), 111.4 (CH), 110.2 (CH), 64.6 (CH_2), 62.4 (CH_2).

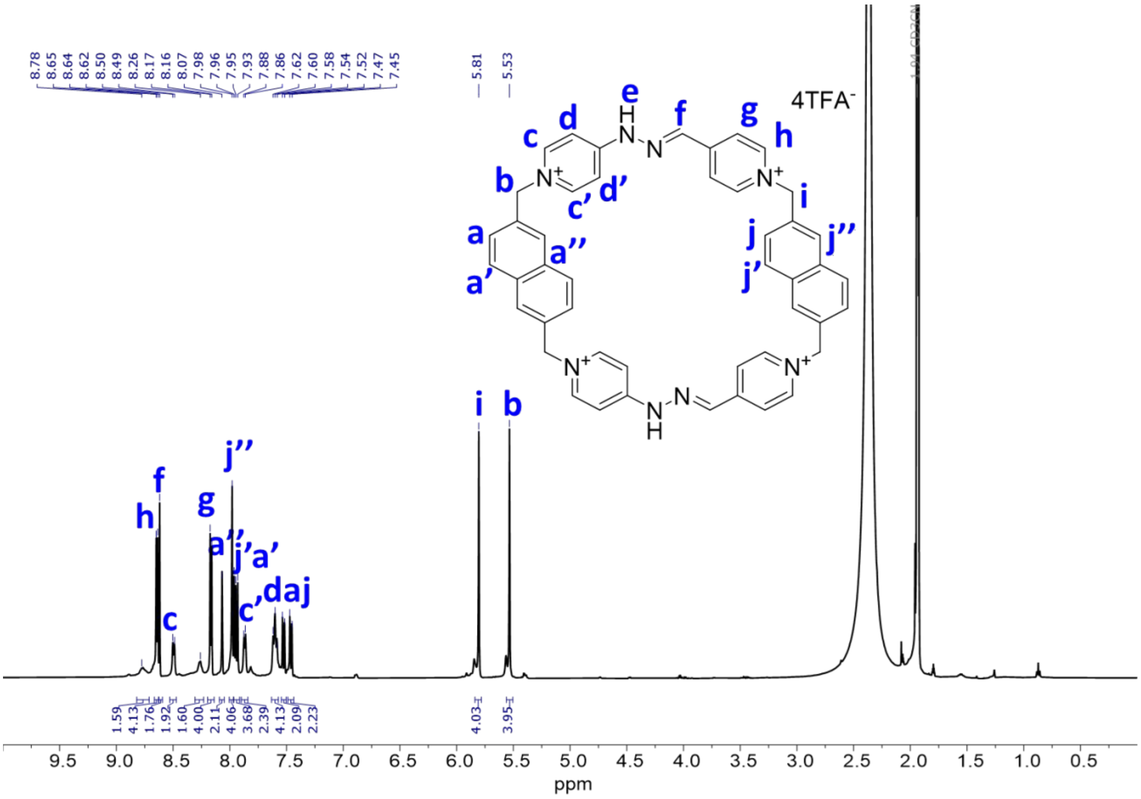

Figure S 96. ^{1}H NMR (500 MHz, CD_3CN) spectrum of $\mathbf{R_d}H_2$·4TFA

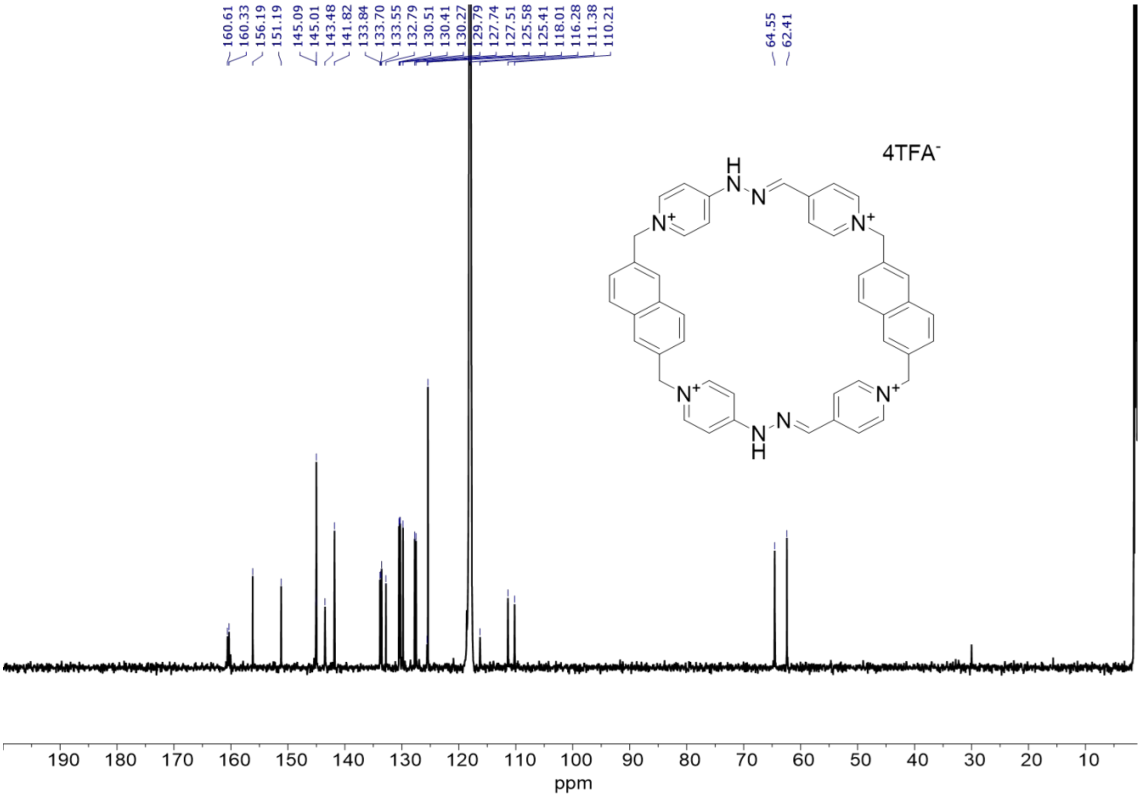

Figure S 97. $^{13}C\{^{1}H\}$ NMR (126 MHz, CD_3CN) spectrum of $\mathbf{R_d}H_2$·4TFA

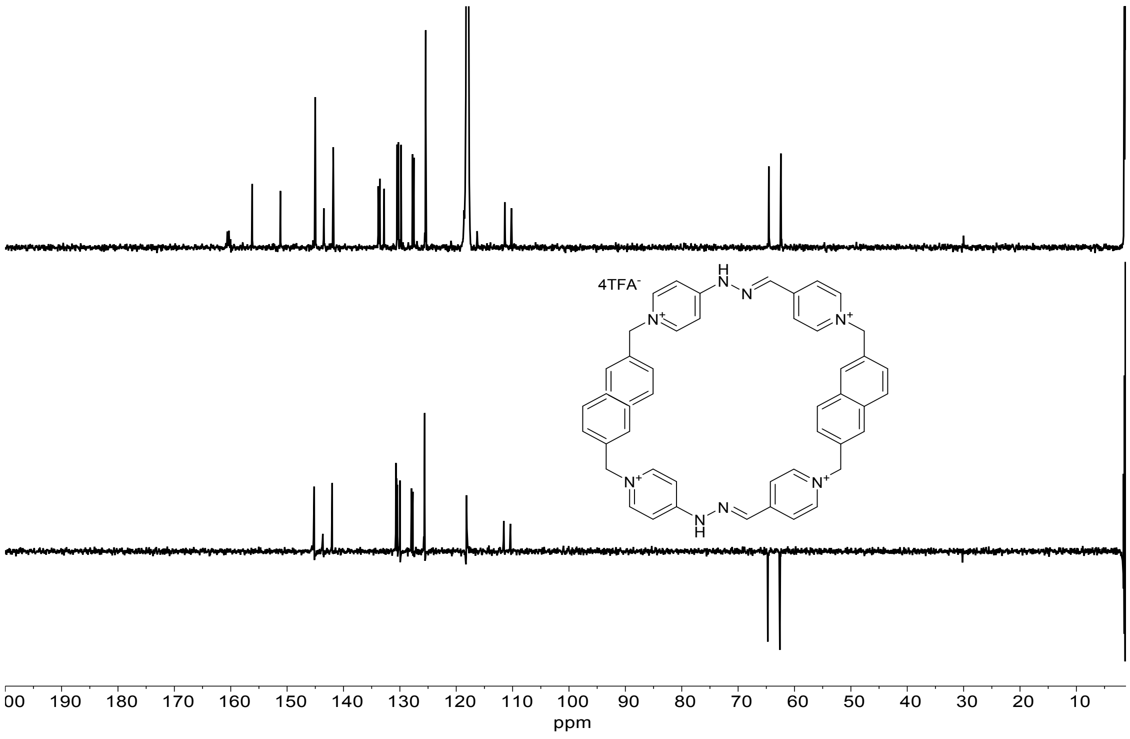

Figure S 98. $^{13}C\{^{1}H\}$ NMR (126 MHz, CD_3CN) spectrum (top) and DEPT-135 (126 MHz, CD_3CN) spectrum (bottom) of $\mathbf{R_d}H_2{\cdot}4TFA$

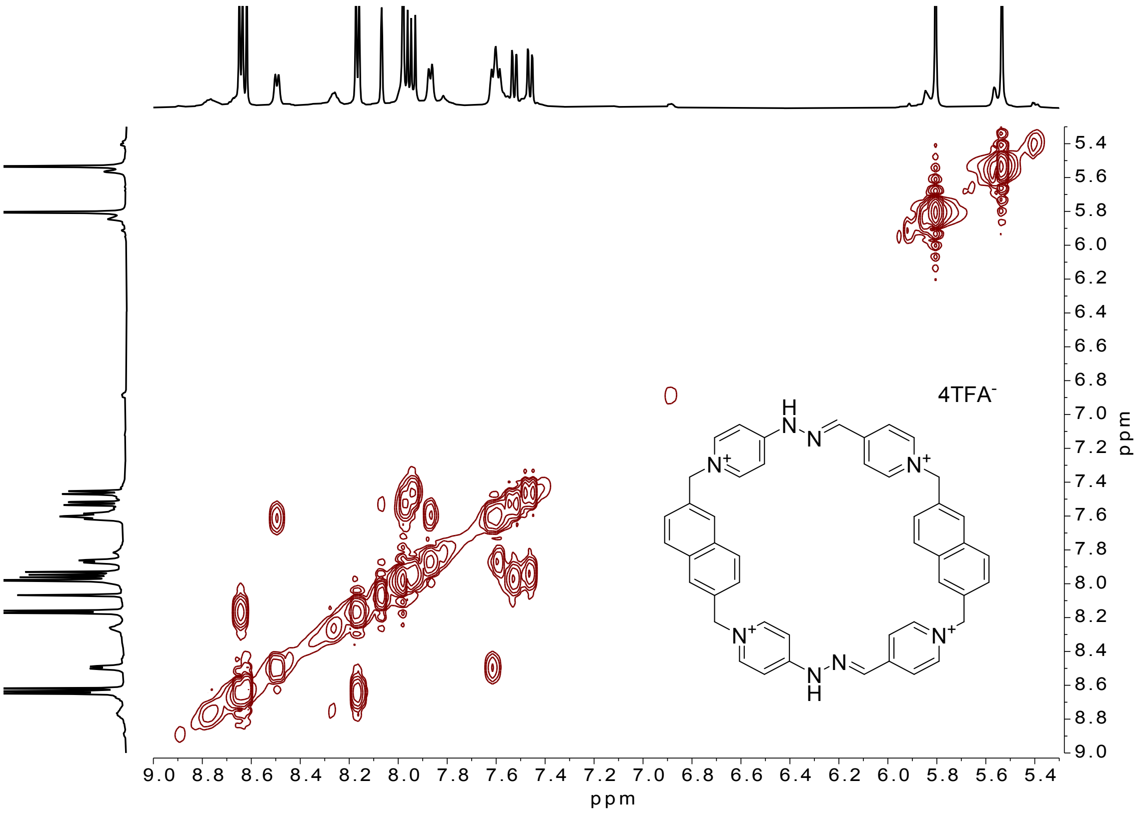

Figure S 99. ^{1}H-^{1}H COSY (500 MHz, CD_3CN) spectrum of $\mathbf{R_d}H_2{\cdot}4TFA$

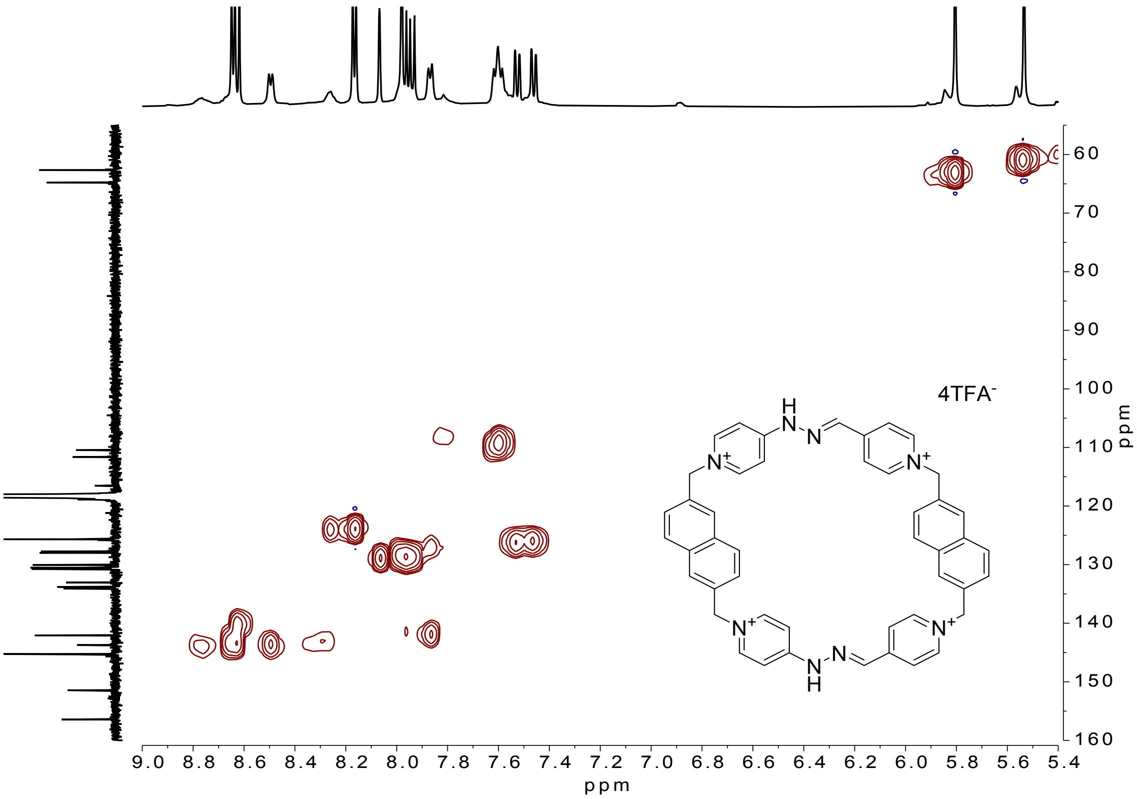

Figure S 100. ^{1}H-^{13}C HSQC (500 MHz/126 MHz, CD_3CN) spectrum of $\mathbf{R_d}H_2$·4TFA

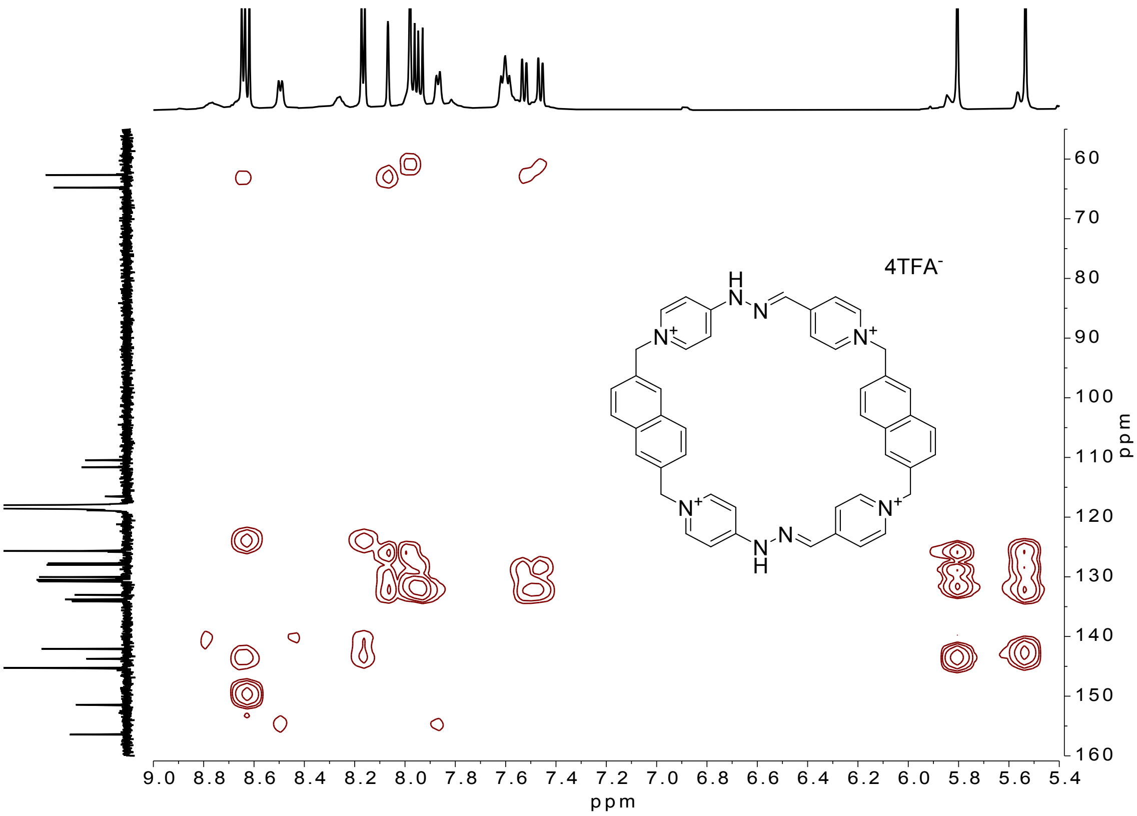

Figure S 101. ^{1}H-^{13}C HMBC (500 MHz/126 MHz, CD_3CN) spectrum of $\mathbf{R_d}H_2$·4TFA

1.2.12. Synthesis and characterization data of $\mathbf{R_eH_2}$·4TFA

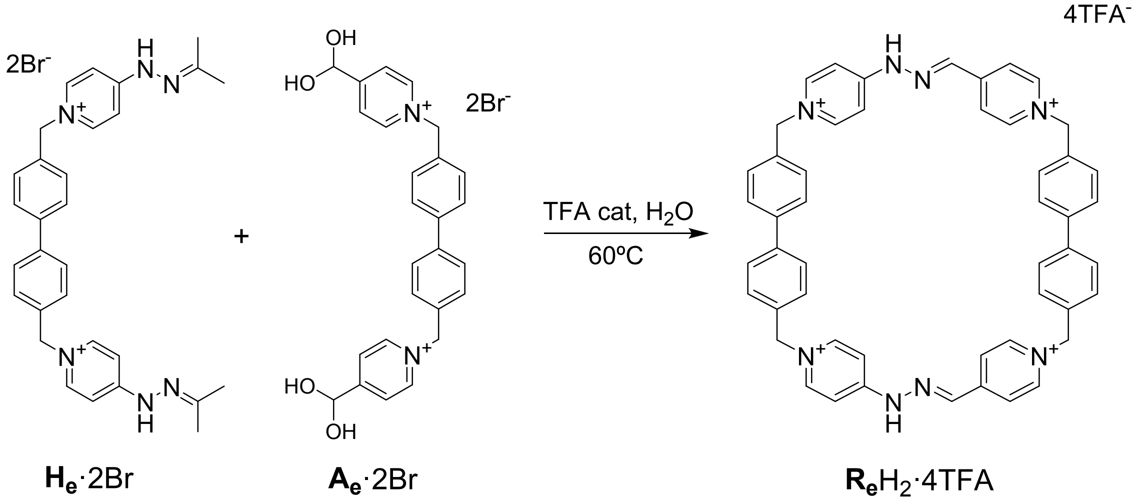

Equimolar quantities of $\mathbf{H_e}$·2Br (2.00 g, 3.13 mmol, 1 eq), and $\mathbf{A_e}$·2Br (1.85 g, 3.13 mmol, 1 eq) were dissolved in 1.2 L of water with 24.0 µL (10 mol%) of trifluoroacetic acid (divided into three 500 mL round bottom flasks). This mixture was agitated and heated at 60°C for 24 hours using a magnetic hot plate stirrer. After checking the completion of the reaction by NMR, the reaction is cooled and an excess of KPF_6 was added until no further precipitation was observed. The mixture was left stirring at room temperature for 30 min. The obtaining reddish solid is then filtered under vacuum and washed with water (3×50 mL) and diethyl ether (3×50 mL). $\mathbf{R_e}H_2$·4PF_6 was obtained with a purity of 60% (2.22 g), achieving a yield of 72%. The product was purified by reverse-phase semipreparative HPLC (RP, H_2O + 0.1% TFA/CH_3CN + 0.1% TFA = 5/95, flow rate = 0.3 mL/min, I = 220 nm, t_R = 12.0 min), yielding $\mathbf{R_e}H_2$·4TFA as a yellowish solid (0.98 g, 49 %), with an overall reaction yield of 26%.

mp 318.4 – 319.6°C (decomposition). **^{1}H-RMN** (500 MHz, D_2O), δ (ppm): 8.80 (d, J = 6.6 Hz, 4H), 8.57 (bs, 2H), 8.24 (d, J = 5.8 Hz, 4H), 8.24 (s, 2H), 8.11 (bs, 2H), 7.75 (dd, J = 8.4, 6.9 Hz, 8H), 7.53 (d, J = 8.1 Hz, 4H), 7.47 (d, J = 8.1 Hz, 4H), 7.21 (bs, 2H), 5.80 (s, 4H), 5.52 (s, 4H). **^{13}C{^{1}H} NMR** (126 MHz, D_2O), δ (ppm): 154.5 (C), 149.9 (C), 144.2 (CH), 140.7 (CH). 140.6 (CH), 140.3 (C), 133.5 (C), 132.7 (C), 129.8 (CH), 129.6 (CH), 127.8 (CH), 127.6 (CH), 124.7 (CH), 117.4 (C), 115.1 (C), 63.7 (CH_2), 61.5 (CH_2). **HRMS** (**ESI**) m/z: [$\mathbf{R_e}$]$^{2+}$ Calcd for $C_{50}H_{42}N_8^{2+}$ 377.1759; Found 377.1759.

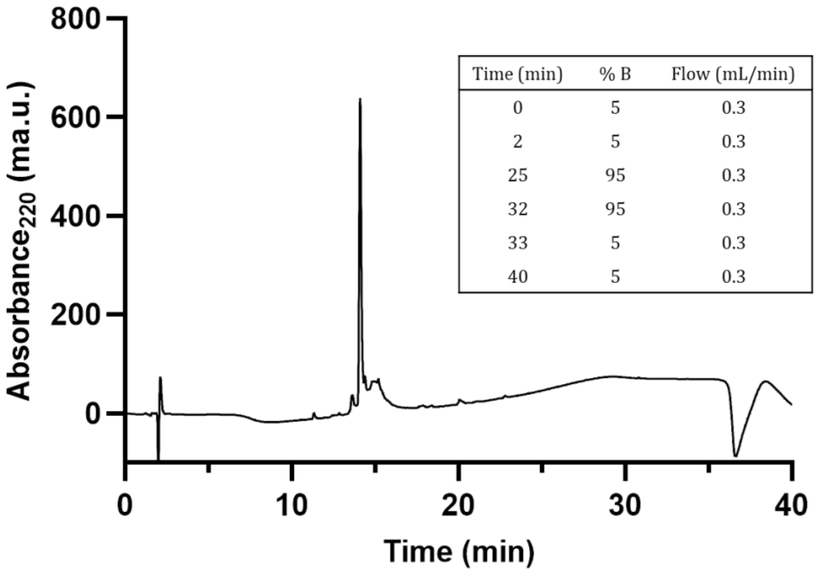

Figure S 102. HPLC chromatogram (220 nm) of $\mathbf{R_e}H_2$·4TFA at t_R = 14.1 min. *Inset*. Elution conditions

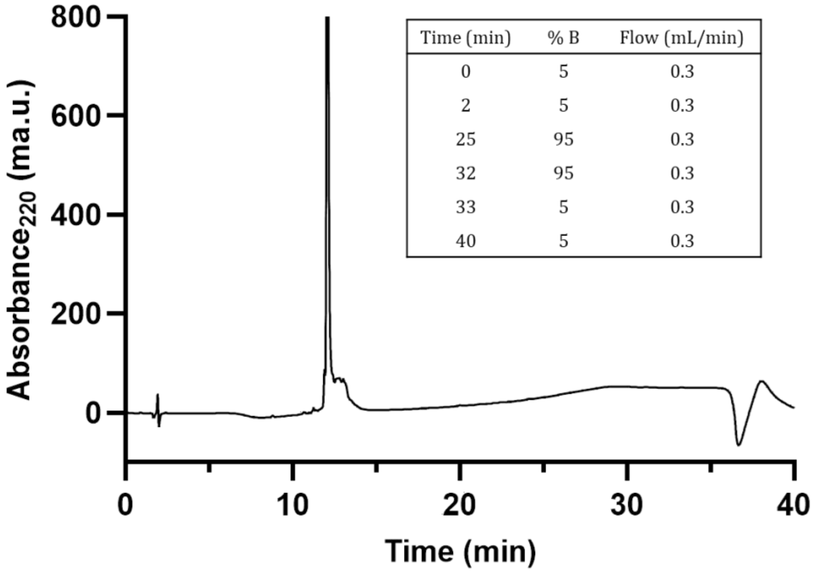

Figure S 103. HPLC chromatogram (220 nm) of purified $\mathbf{R_e}H_2$·4TFA at t_R = 12.0 min. *Inset*. Elution conditions

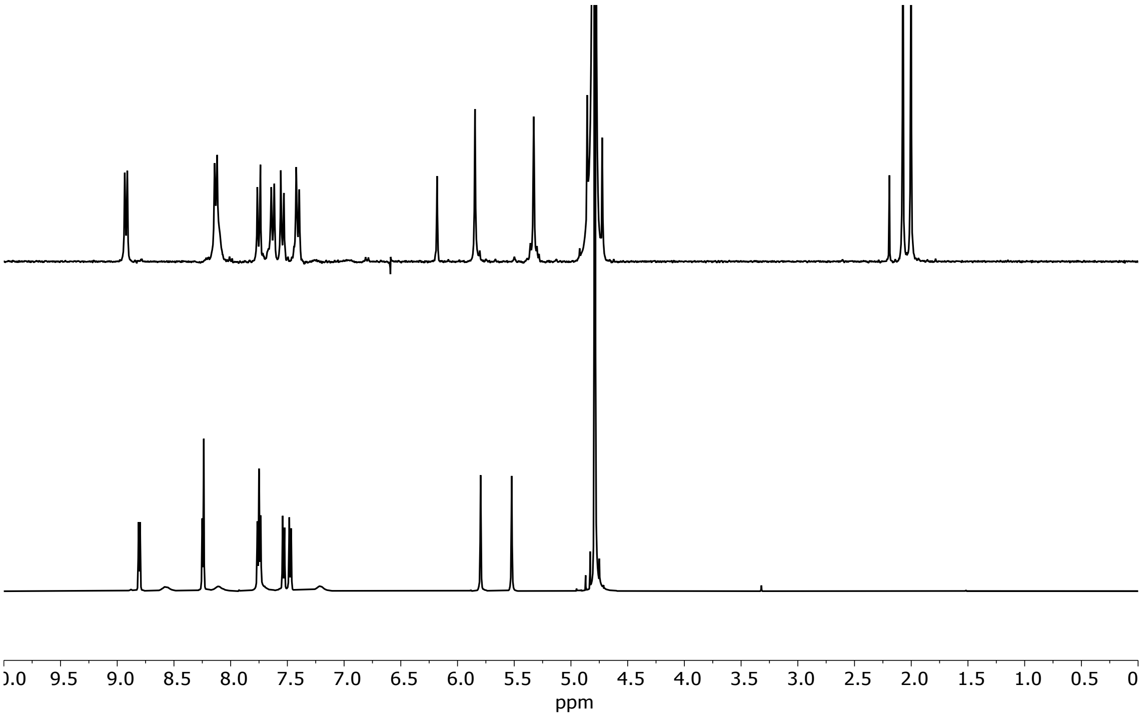

Figure S 104. ^{1}H NMR (300 MHz, D_2O) stacked spectra of: (top) equimolar 2.5 mM mixture of $\mathbf{A_e}$·2Br and $\mathbf{H_e}$·2Br at t = 0; (bottom) same mixture after 24 hours at 60 °C with TFA-d_3 (10% molar)

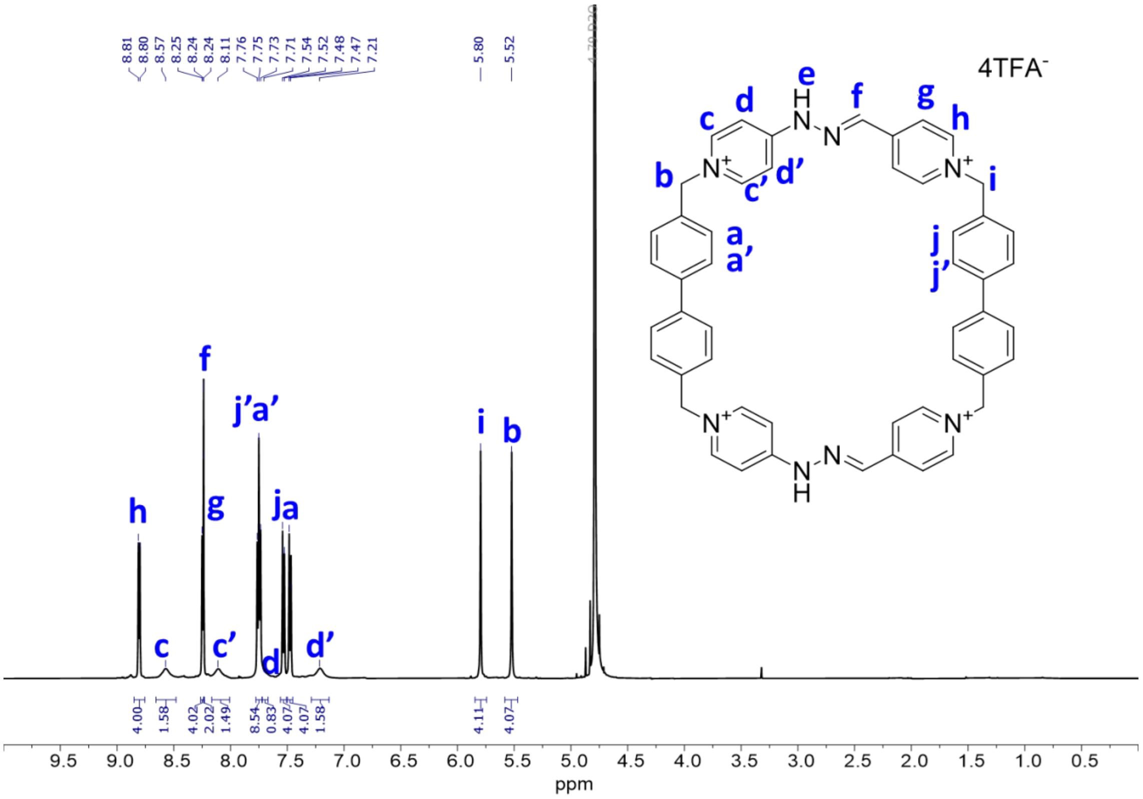

Figure S 105. ^{1}H NMR (500 MHz, D_2O) spectrum of $\mathbf{R_e}H_2$·4TFA

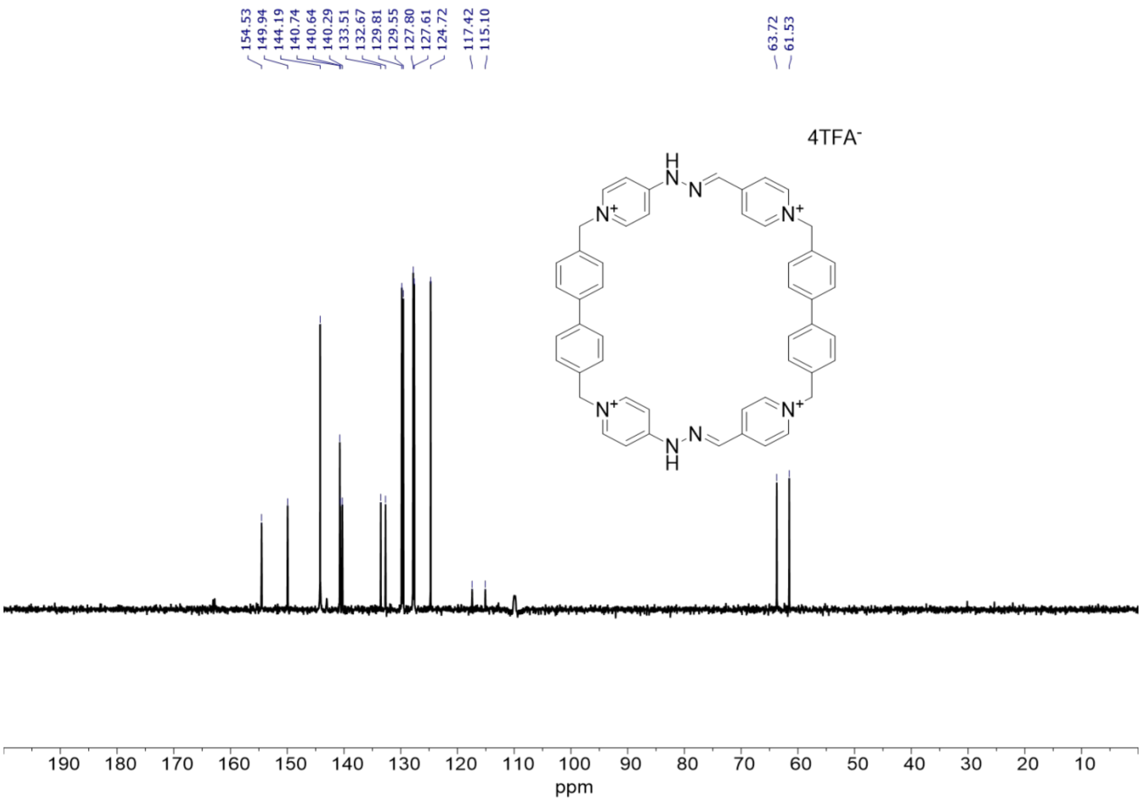

Figure S 106. $^{13}C\{^{1}H\}$ NMR (126 MHz, D_2O) spectrum of $\mathbf{R_e}H_2$·4TFA

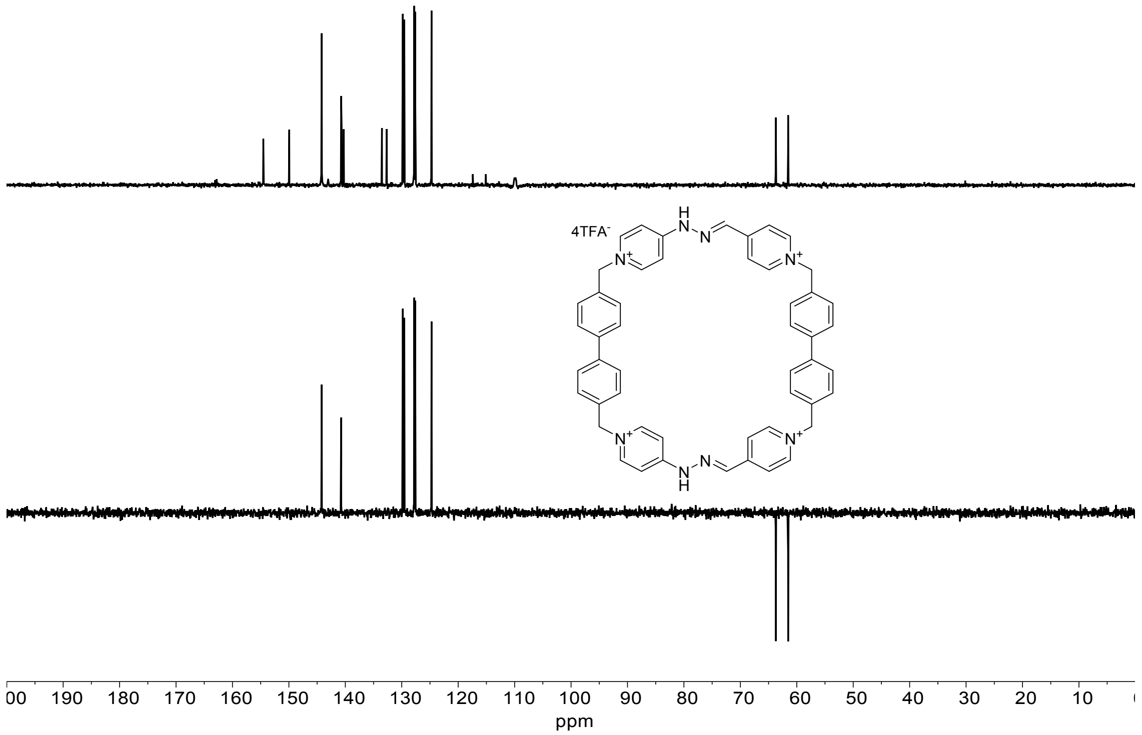

Figure S 107. $^{13}C\{^{1}H\}$ NMR (126 MHz, D_2O) spectrum (top) and DEPT-135 (126 MHz, D_2O) spectrum (bottom) of $\mathbf{R_e}H_2$·4TFA

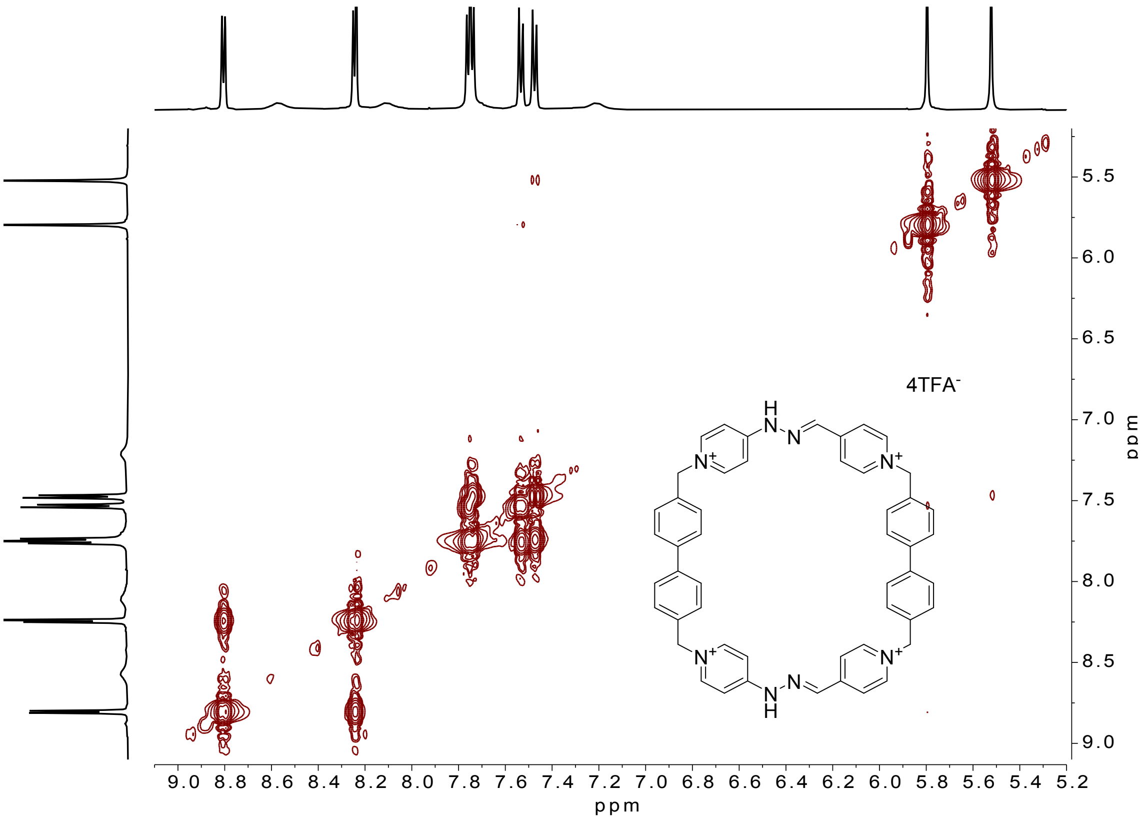

Figure S 108. ^{1}H-^{1}H COSY (500 MHz, D_2O) spectrum of $\mathbf{R_e}H_2$·4TFA

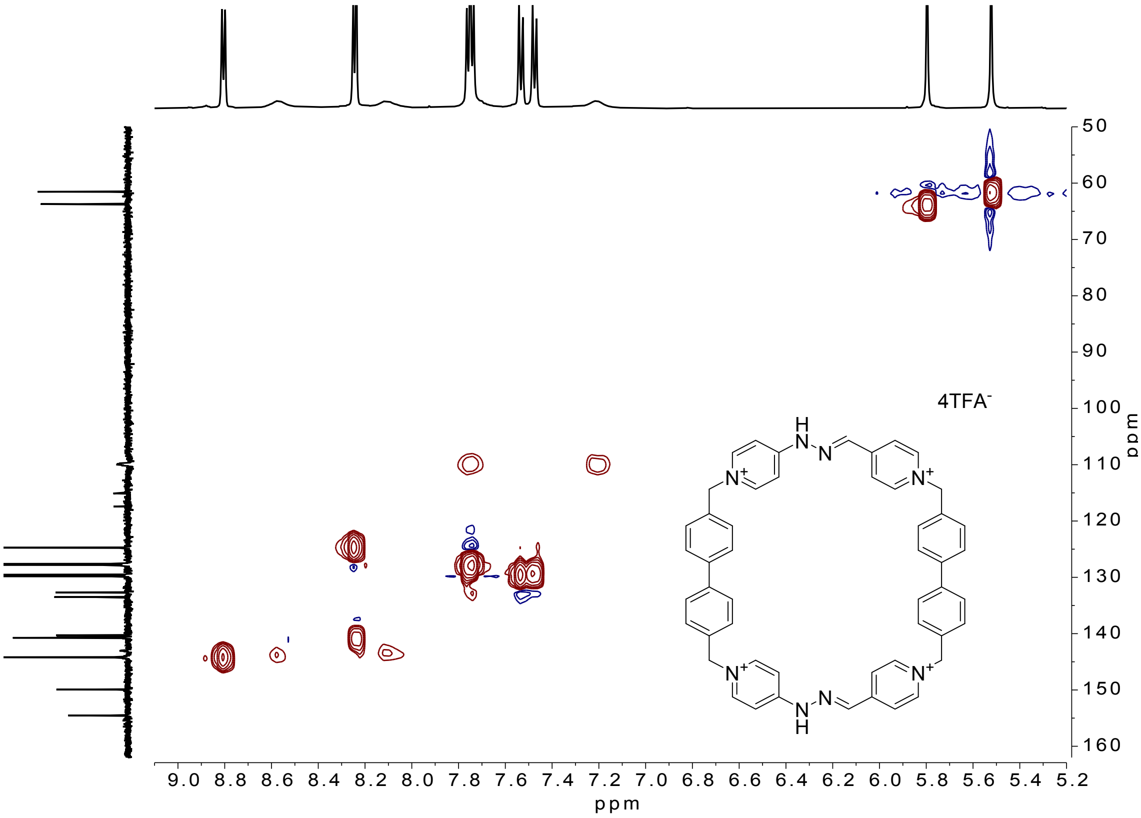

Figure S 109. ^{1}H-^{13}C HSQC (500 MHz, D_2O) spectrum of $\mathbf{R_e}H_2$·4TFA

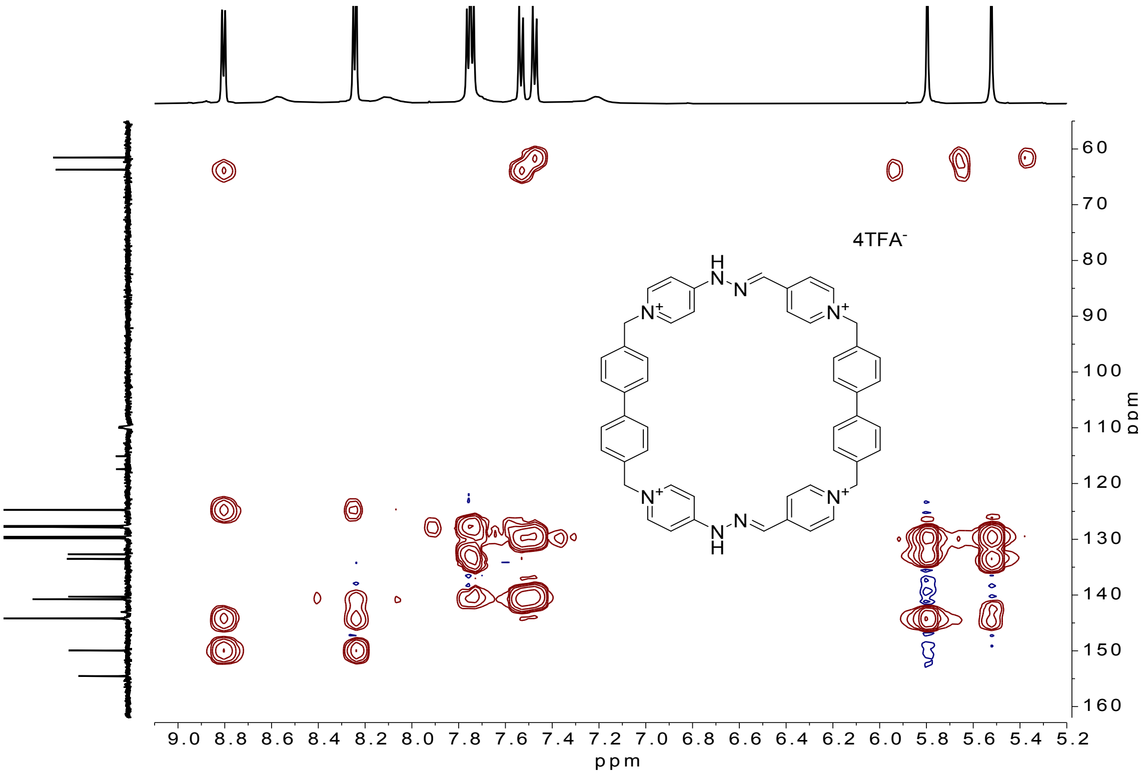

Figure S 110. ^{1}H-^{13}C HMBC (500 MHz, D_2O) spectrum of **R**$_e$$H_2$·4TFA

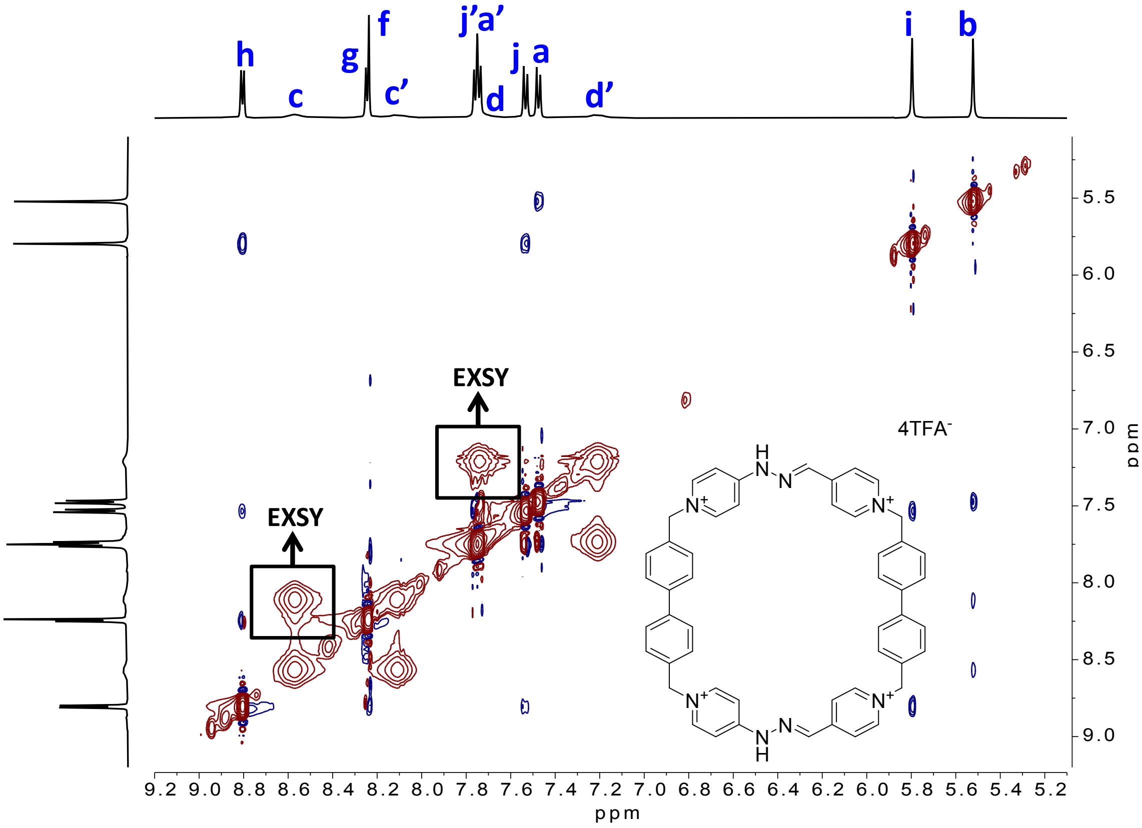

Figure S 111. ^{1}H-^{1}H NOESY (500 MHz, D_2O) spectrum of **R**$_e$$H_2$·4TFA

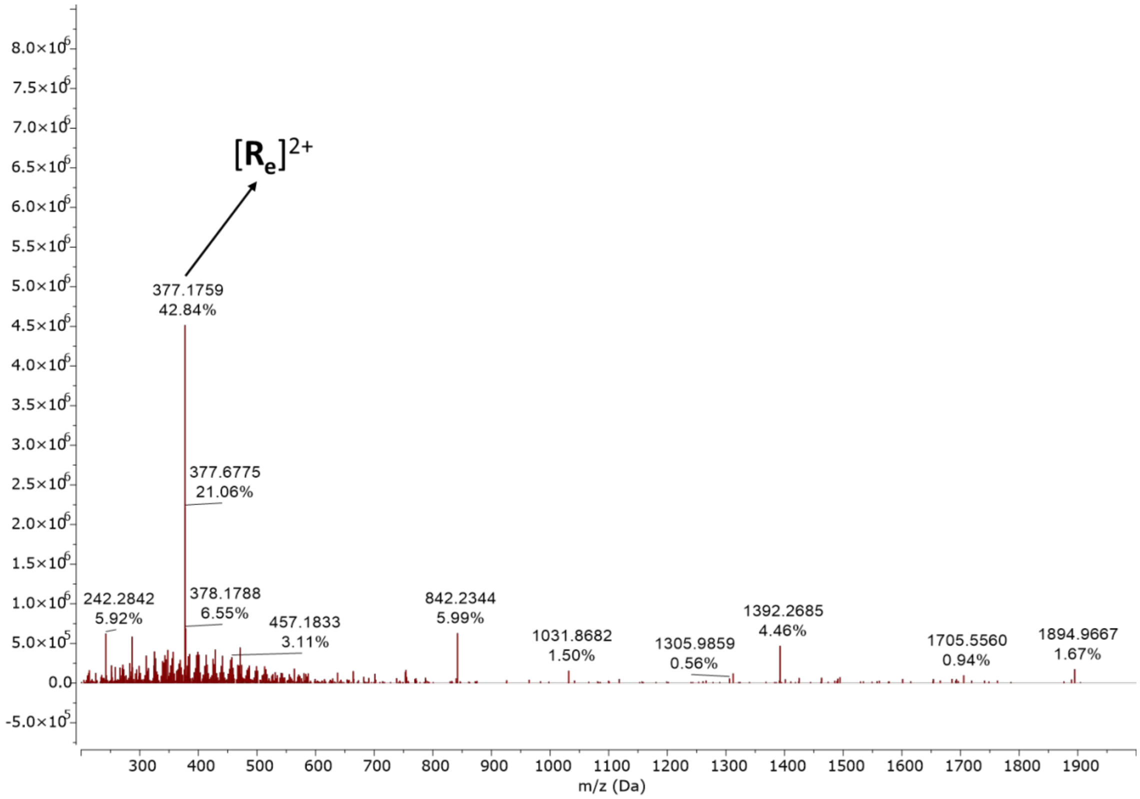

Figure S 112. HR ESI-MS spectrum of $\mathbf{R_e}H_2$·4TFA

^{1}H-RMN (500 MHz, CD_3CN), δ (ppm): 8.48 (d, J = 6.5 Hz, 4H), 8.43 (s, 2H), 8.24 (d, J = 7.1 Hz, 2H), 8.09 (d, J = 6.7 Hz, 2H), 8.03 (d, J = 6.5 Hz, 4H), 7.80 (d, J = 7.8 Hz, 2H), 7.52 (m, 12H), 7.41 (d, J = 7.0 Hz, 2H), 7.31 (d, J = 8.2 Hz, 4H), 7.24 (d, J = 8.1 Hz, 4H), 5.50 (s, 4H), 5.24 (s, 4H). **^{13}C{^{1}H} NMR** (126 MHz, CD_3CN), δ (ppm): 159.3 (C), 159.0 (C), 155.1 (C), 150.2 (C), 144.0 (CH), 140.9 (CH), 140.4 (C), 140.0 (C), 133.7 (C), 132.8 (C), 129.5 (CH), 129.1 (CH), 127.6 (CH),), 127.5 (CH), 127.3 (CH), 124.6, (CH), 114.9 (C), 110.5 (C), 109.2 (C), 63.2 (CH_2), 61.0 (CH_2).

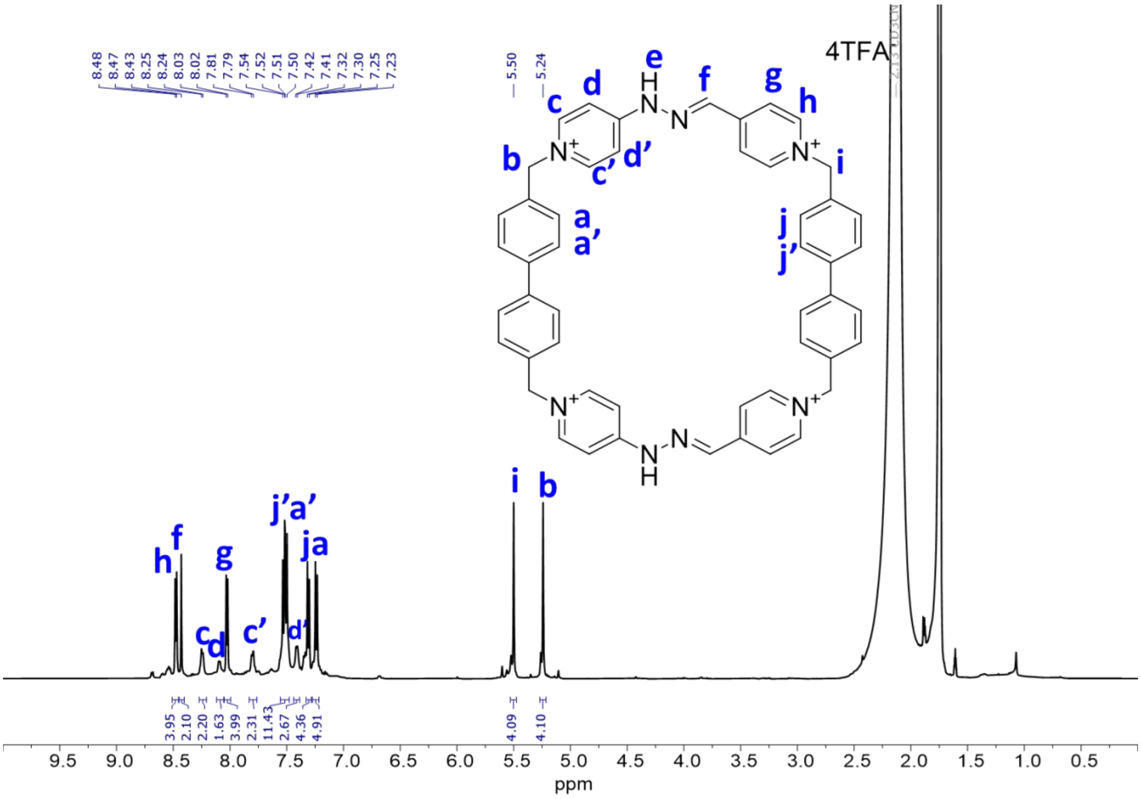

Figure S 113. ^{1}H NMR (500 MHz, CD_3CN) spectrum of $\mathbf{R_e}H_2{\cdot}4TFA$

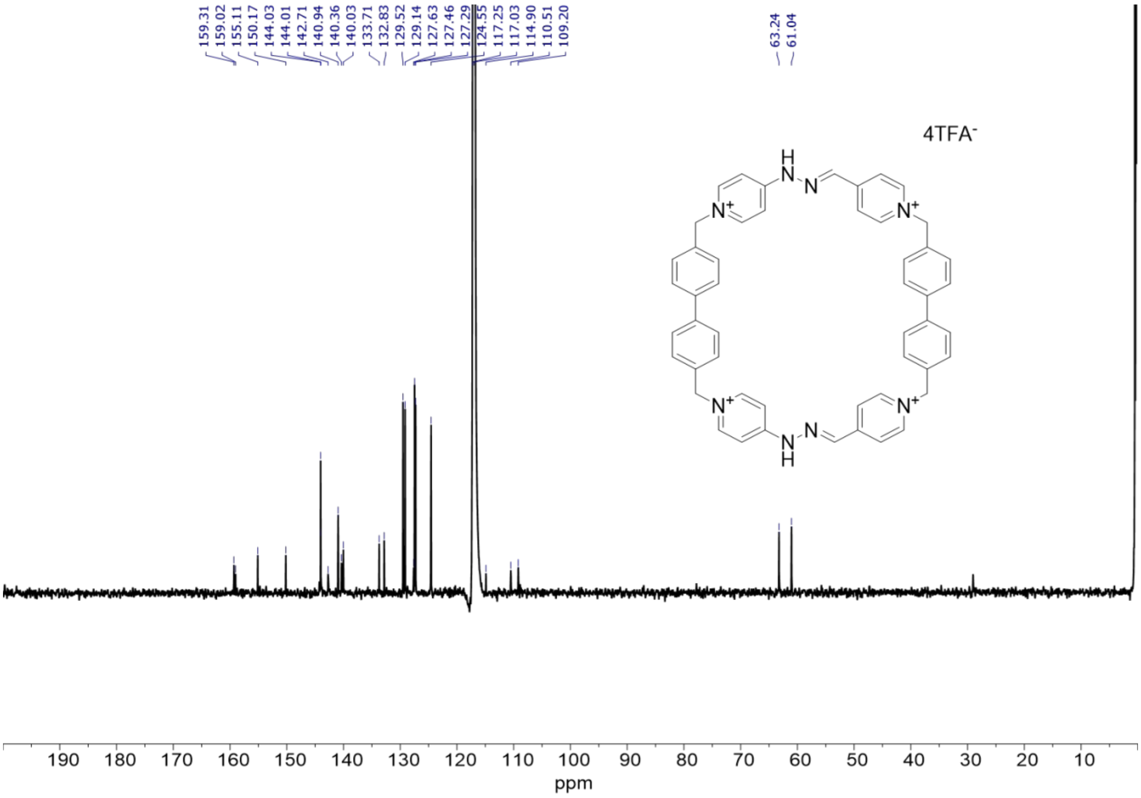

Figure S 114. $^{13}C\{^{1}H\}$ NMR (126 MHz, D_2O) spectrum of $\mathbf{R_e}H_2{\cdot}4TFA$

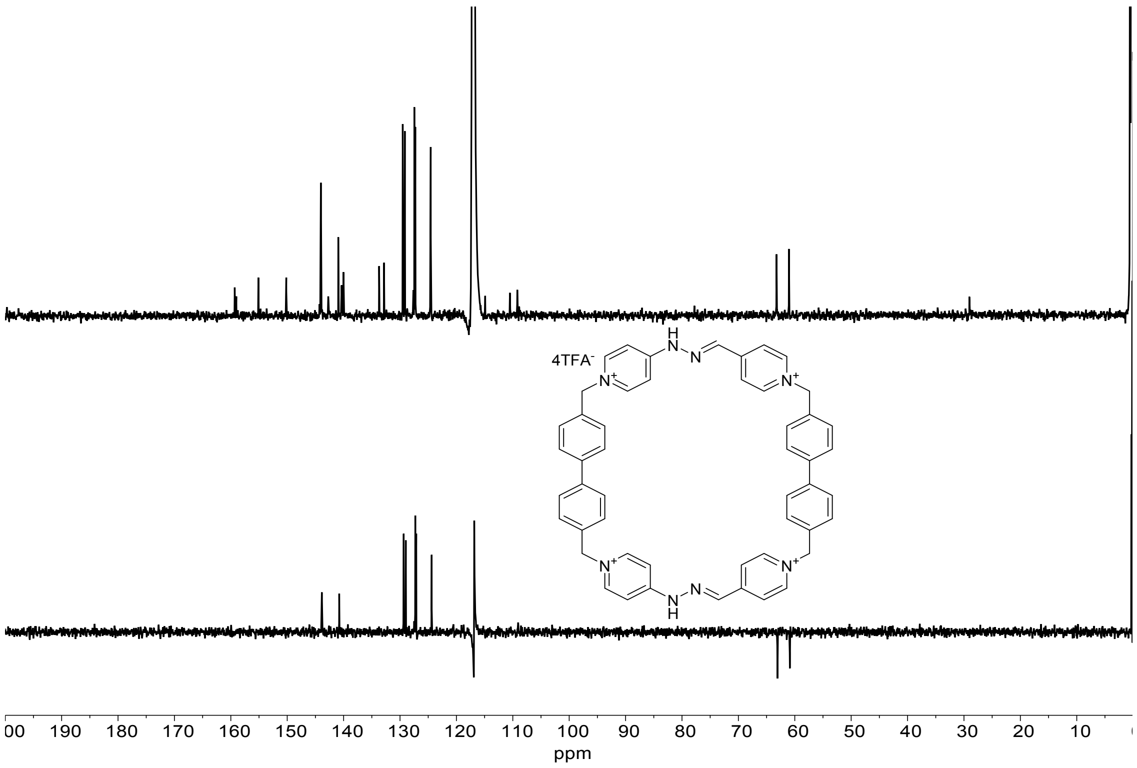

Figure S 115. $^{13}C\{^{1}H\}$ NMR (126 MHz, D_2O) spectrum (top) and DEPT-135 (126 MHz, D_2O) spectrum (bottom) of $\mathbf{R_e}H_2$·4TFA

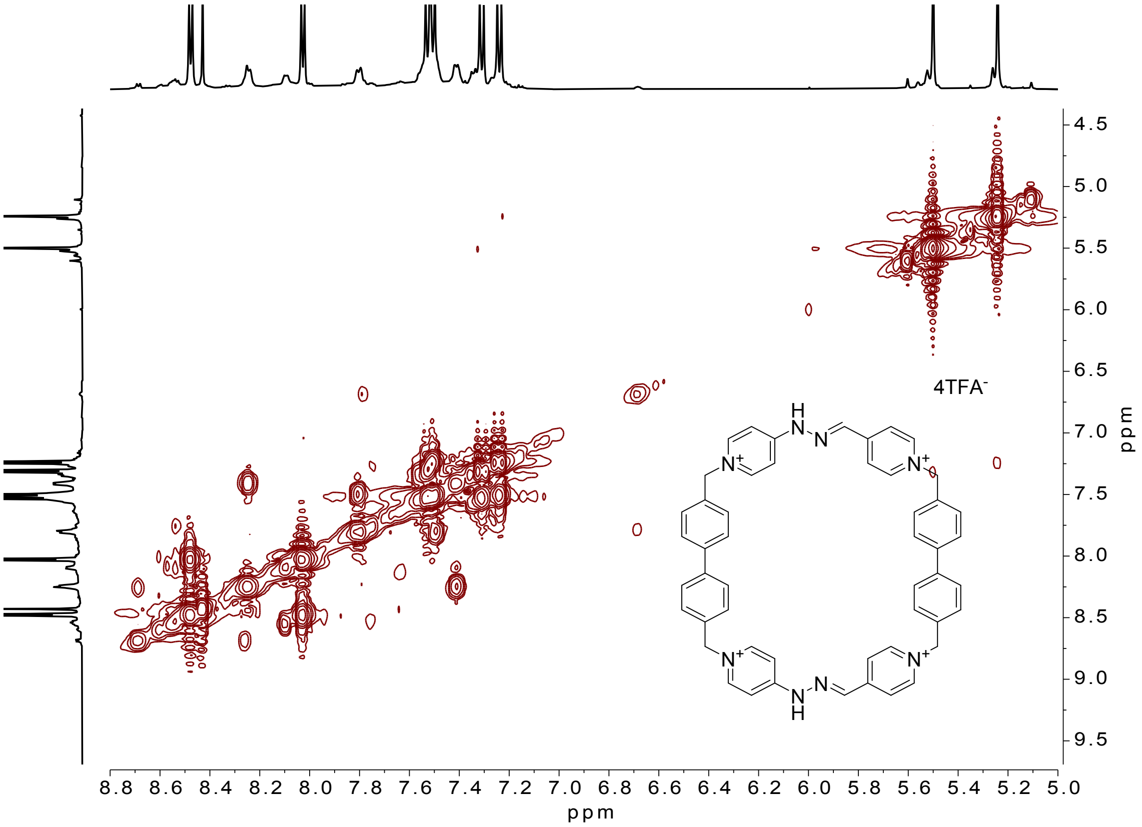

Figure S 116. ^{1}H-^{1}H COSY (500 MHz, CD_3CN) spectrum of $\mathbf{R_e}H_2$·4TFA

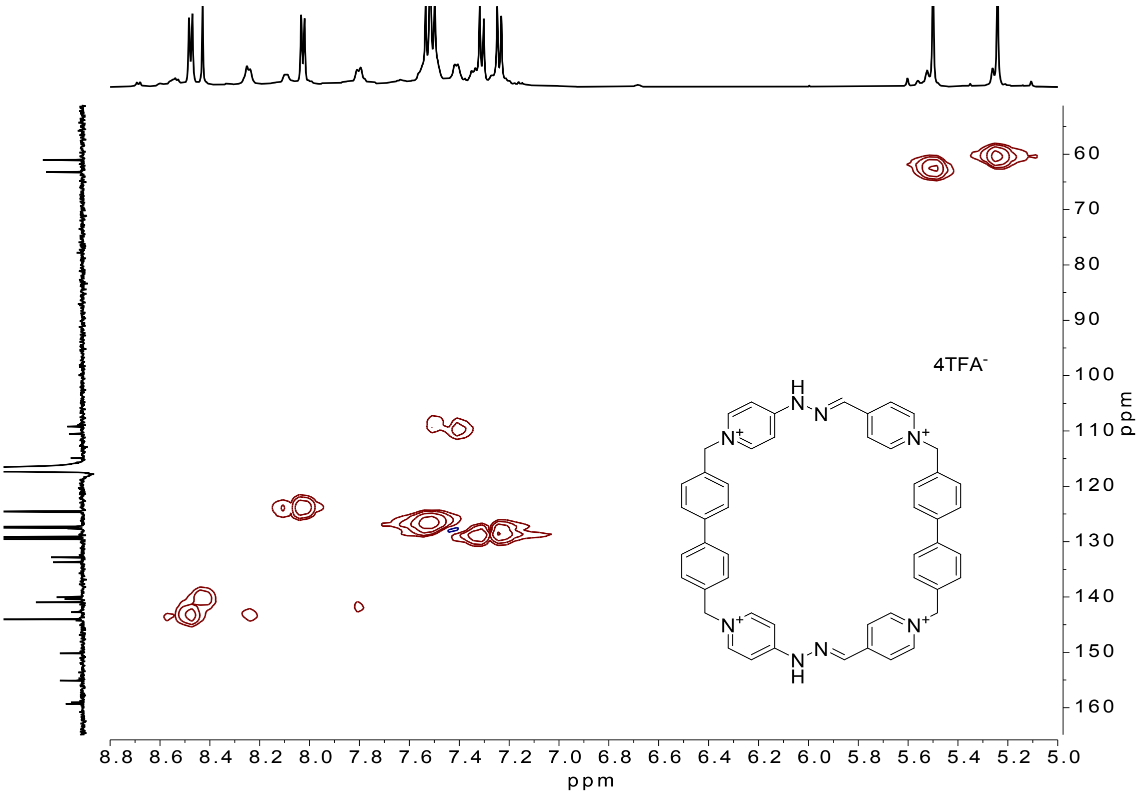

Figure S 117. ^{1}H-^{13}C HSQC (500 MHz, CD_3CN) spectrum of $\mathbf{R_e}H_2$·4TFA

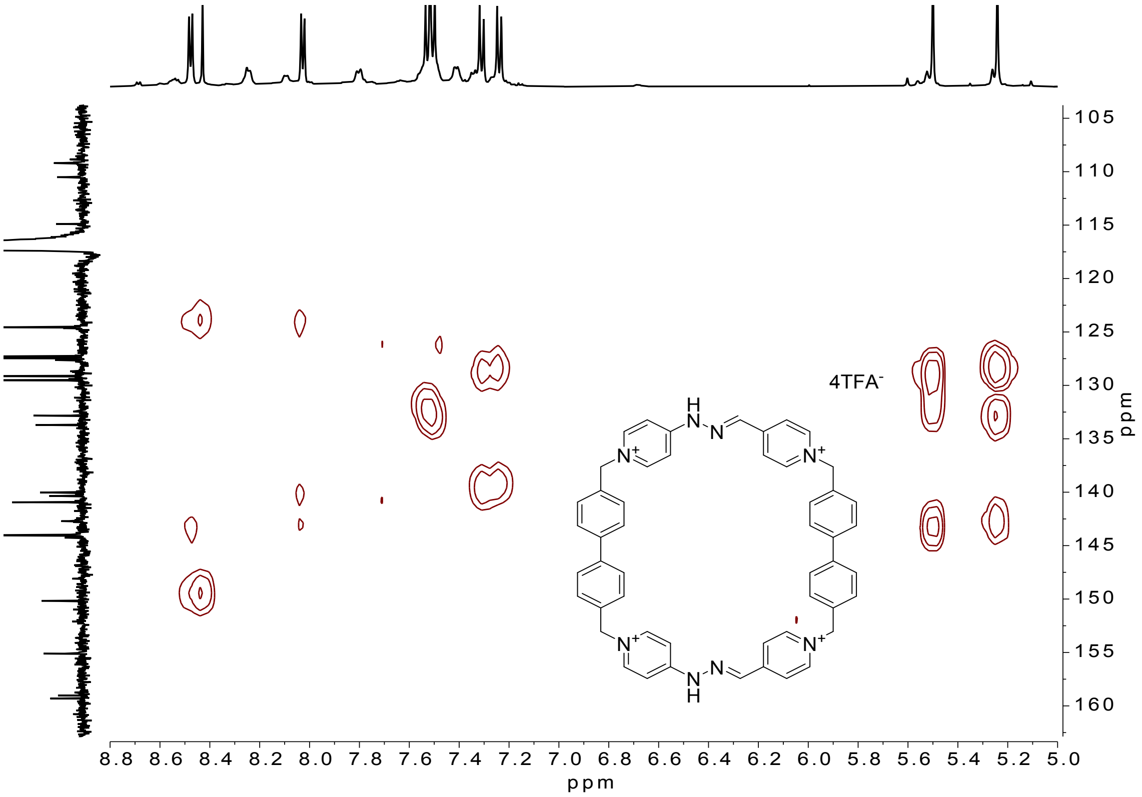

Figure S 118. ^{1}H-^{13}C HMBC (500 MHz/126 MHz, CD_3CN) spectrum of $\mathbf{R_e}H_2$·4TFA

1.3. Determination of the energy of the rotational barrier (ΔG≠)

The coalescence temperature (Tc) could be estimated for different protons on VT NMR experiments. This provides, in association with the maximum peak separation (Δ**ν** in Hz) at slow exchange between **c** – **c'** and **d** – **d'** for **R$_{b\text{-}e}$**H_2·4TFA, the energy of the rotational barrier using Equation (1).[2]

$$\Delta G^{\neq} = 4.57{\cdot}10^{-0}\, Tc\, (9.972\ +\ log\, Tc/\Delta\nu) \qquad (1)$$

1.3.1. ΔG≠ for R$_b$H$_2$·4TFA

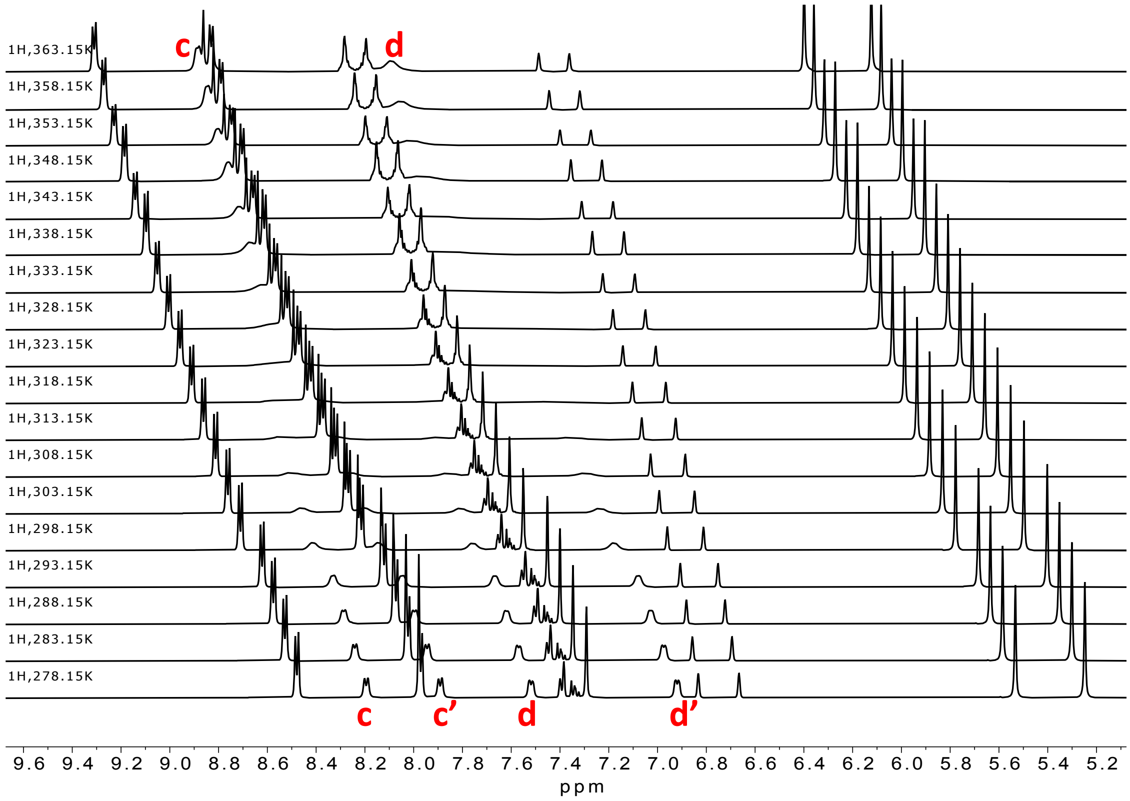

Figure S 119. VT ^{1}H NMR (400 MHz, D_2O) stacked spectra detail of **R$_b$**H_2·4TFA

[2] a)J. Sandstrom, Dynamic NMR Spectroscopy; Academic Press: New York, NY, USA, 1983. b) H. Kessler, Angew. Chem. Int. Ed. 1970, 9, 219-235.

Table S 1. Experimental data obtained for the calculation of ΔG≠ via the coalescence temperature of signals of **c** – **c'** and **d** – **d'** protons on the VT ^{1}H NMR of **R**$_b$$H_2$·4TFA

Signal	Δ*ν* (Hz)	Tc (K)	ΔG≠ (kcal mol^{-1})
**c – c'**	152	327.15	15.4
**d – d'**	300	338.15	15.5

1.3.2. **ΔG≠ for R$_c$H$_2$·4TFA**

The coalescence temperature (Tc) could not be estimated for different protons on VT NMR experiments.

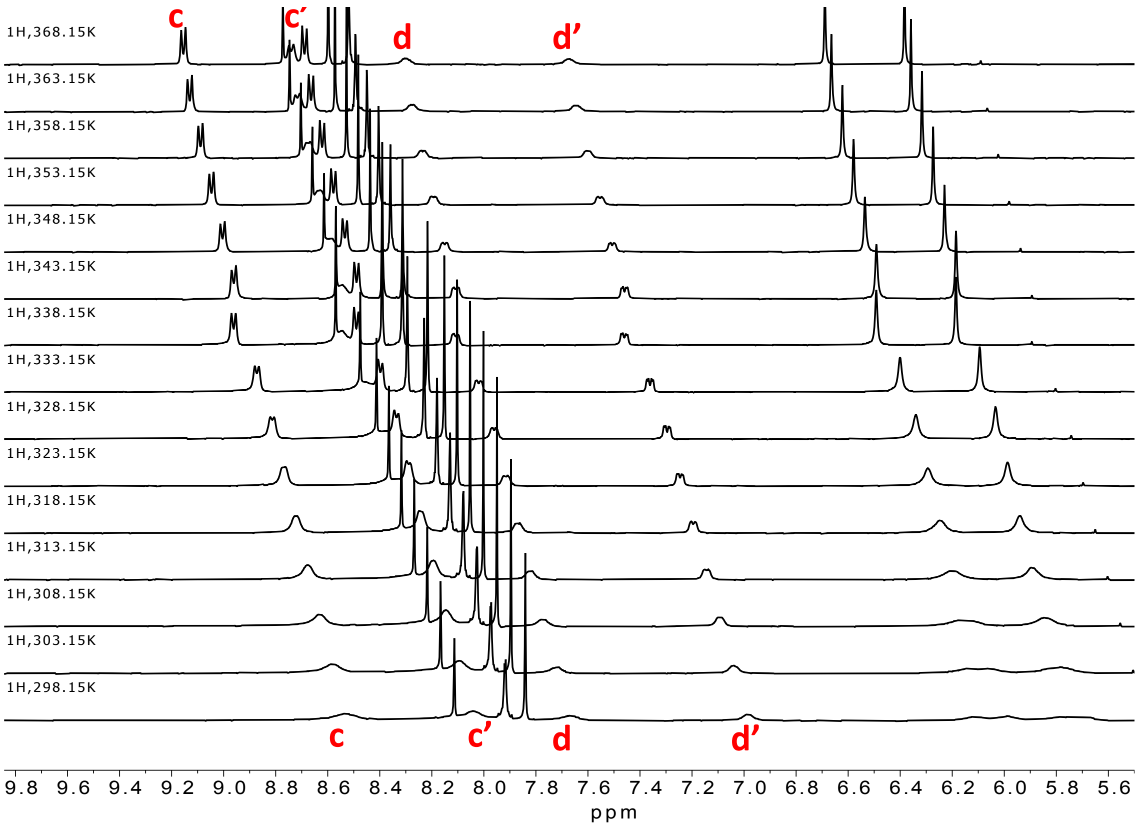

Figure S 120. VT ^{1}H NMR (400 MHz, D_2O) stacked spectra of **R**$_c$$H_2$·4TFA

1.3.3. **ΔG≠ for R_dH_2·4TFA**

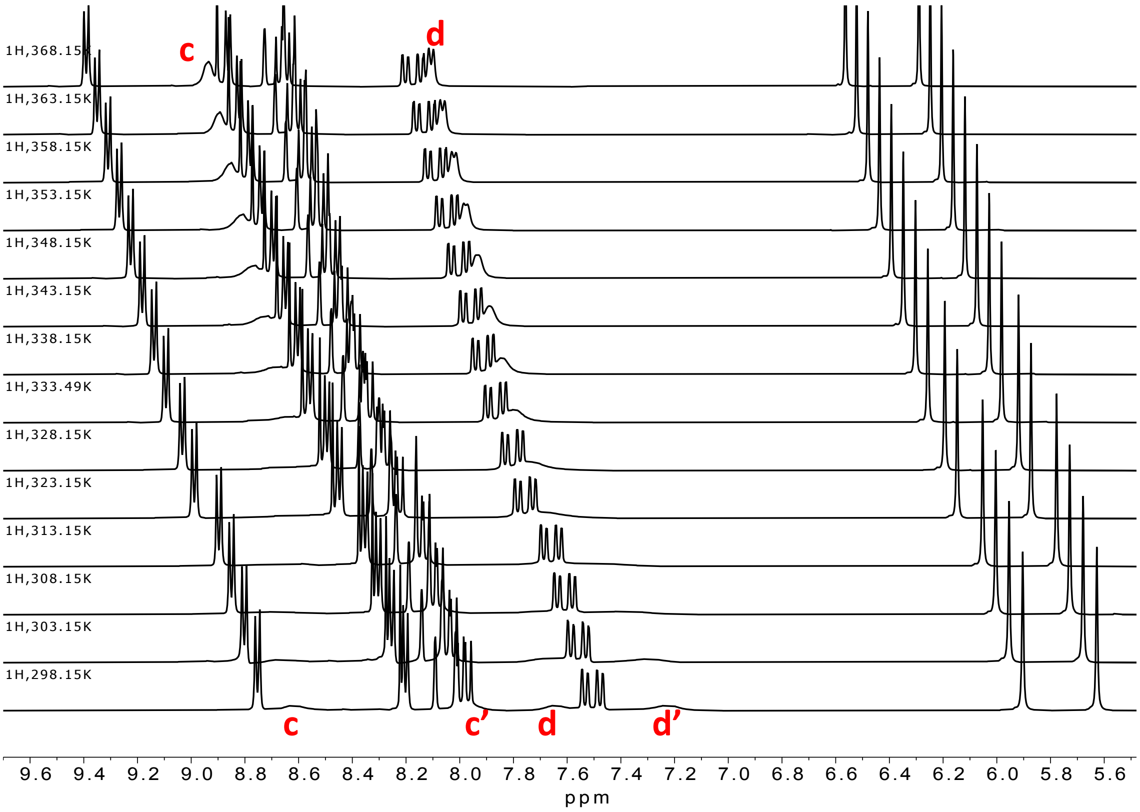

Figure S 121. VT ^{1}H NMR (400 MHz, D_2O) stacked spectra detail of $\mathbf{R_d}H_2$·4TFA

Table S 2. Experimental data obtained for the calculation of ΔG≠ via the coalescence temperature of signals of **c** – **c'** and **d** – **d'** protons on the VT ^{1}H NMR of $\mathbf{R_d}H_2$·4TFA

**Signal**	**Δ*ν* (Hz)**	**Tc (K)**	**ΔG≠ (kcal mol^{-1})**
**c – c'**	253	333.15	15.4
**d – d'**	170	328.15	15.4

1.3.4. **ΔG^≠ for R_eH_2·4TFA**

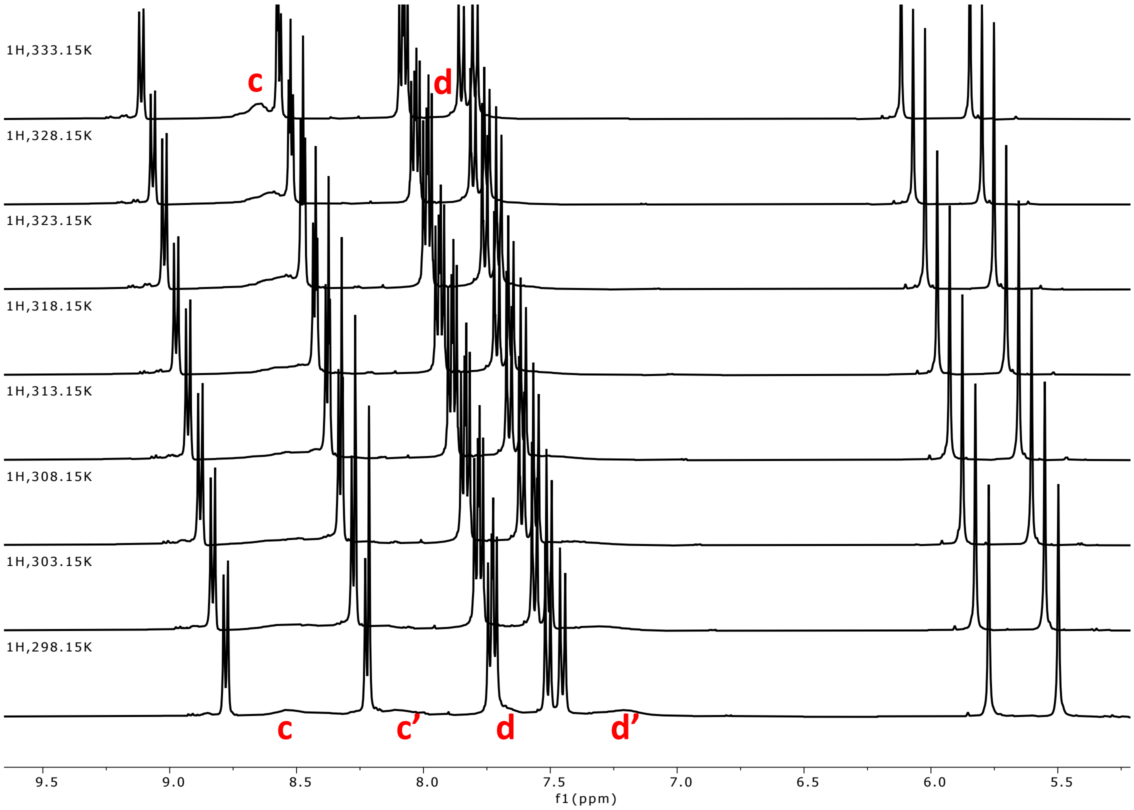

Figure S 122. VT ^{1}H NMR (400 MHz, D_2O) stacked spectra detail of $\mathbf{R_e}H_2$·4TFA

Table S 3. Experimental data obtained for the calculation of ΔG^≠ via the coalescence temperature of signals of **c** – **c'** and **d** – **d'** protons on the VT ^{1}H NMR of $\mathbf{R_e}H_2$·4TFA

Signal	Δν (Hz)	Tc (K)	ΔG^≠ (kcal mol^{-1})
**c – c'**	174	318.15	14.9
**d – d'**	200	323.15	15.0

1.4. UV-vis absorption spectra of $R_{b-e}H_2$·4TFA

1.4.1. UV-vis absorption spectrum of R_bH_2·4TFA

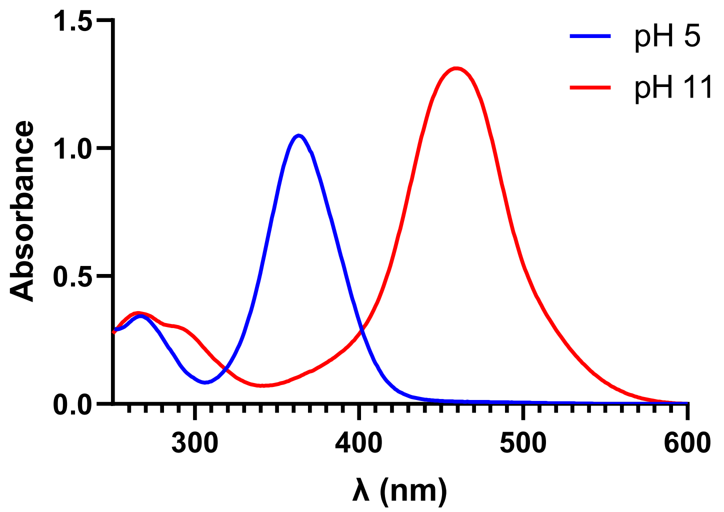

Figure S 123. UV-Vis absorption spectra (phosphate buffer 20 mM) of **R_b**H_2·4TFA 20 µM at pH = 5 (blue) and 11 (red)

1.4.2. UV-vis absorption spectrum of R_cH_2·4TFA

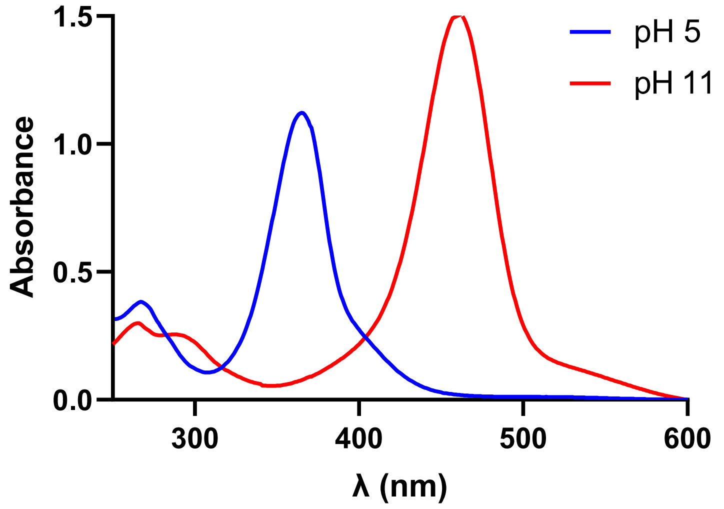

Figure S 124. UV-Vis absorption spectra (phosphate buffer 20 mM) of **R_c**H_2·4TFA 20 µM at pH = 5 (blue) and 11 (red)

1.4.3. **UV-vis absorption spectrum of R_dH_2·4TFA**

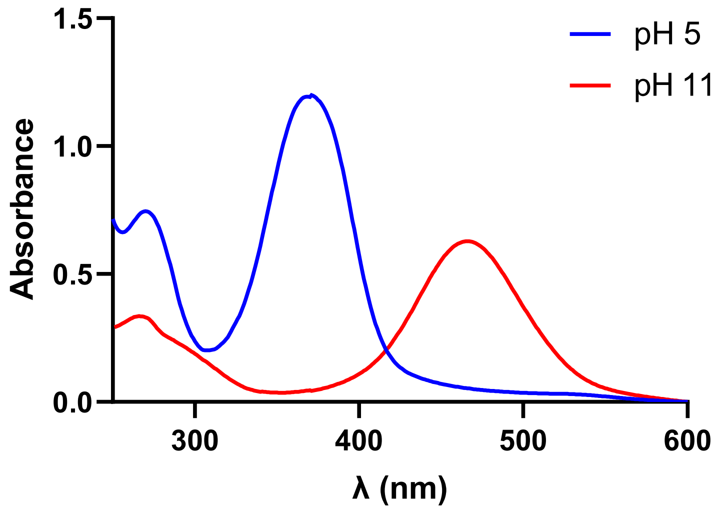

Figure S 125. UV-Vis absorption spectra (phosphate buffer 20 mM) of $\mathbf{R_d}H_2$·4TFA 20 μM at pH = 5 (blue) and 11 (red)

1.4.4. **UV-vis absorption spectrum of R_eH_2·4TFA**

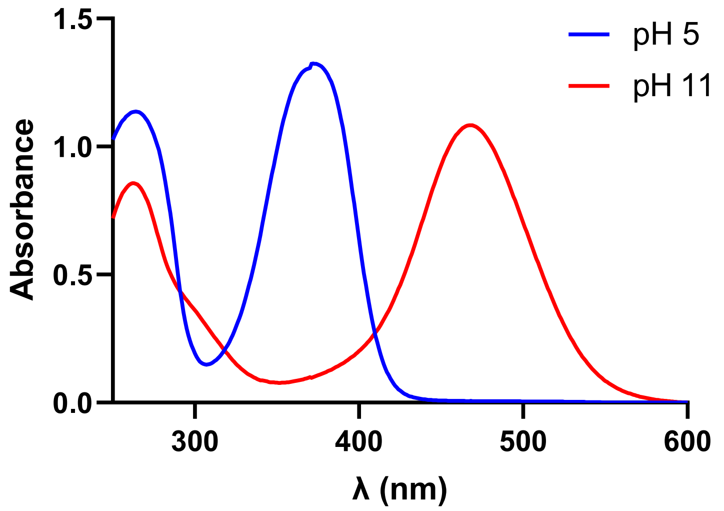

Figure S 126. UV-Vis absorption spectra (phosphate buffer 20 mM) of $\mathbf{R_e}H_2$·4TFA 20 μM at pH = 5 (blue) and 11 (red)

2. HOST-GUEST STUDIES

2.1. Synthesis and characterization data of **4**⊂$\mathbf{R_bH_2}$□4TFA

2.1.1. **4**⊂$\mathbf{R_bH_2}$□4TFA at pD 5

Equimolar 2 mM solutions of $\mathbf{R_b}H_2$□4TFA and the corresponding guest **4** were prepared in 20 mM phosphate buffer solution pD=5 at room temperature, and the corresponding NMR recorded immediately after.

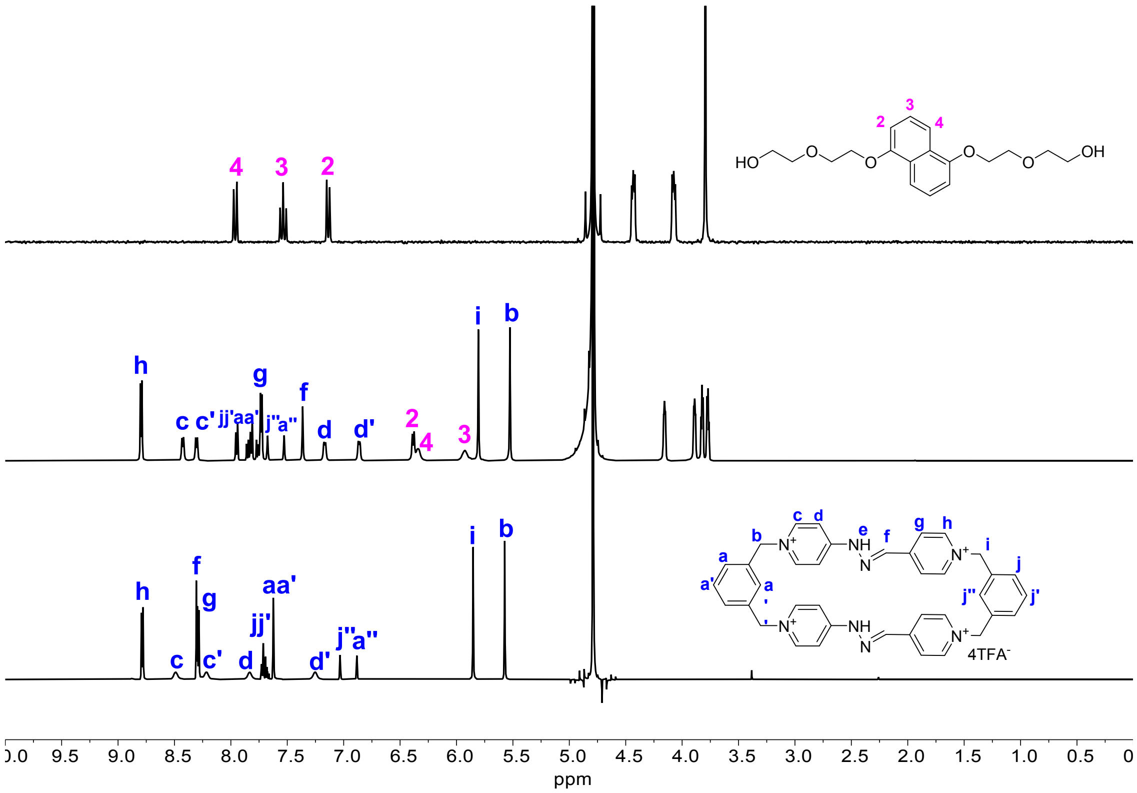

Figure S 127. ^{1}H NMR spectra comparison between the guest **4** (top), the 1:1 inclusion complex **4**⊂ $\mathbf{R_b}H_2$·4TFA at pD=5 (middle) and the host $\mathbf{R_b}H_2$·4TFA (bottom)

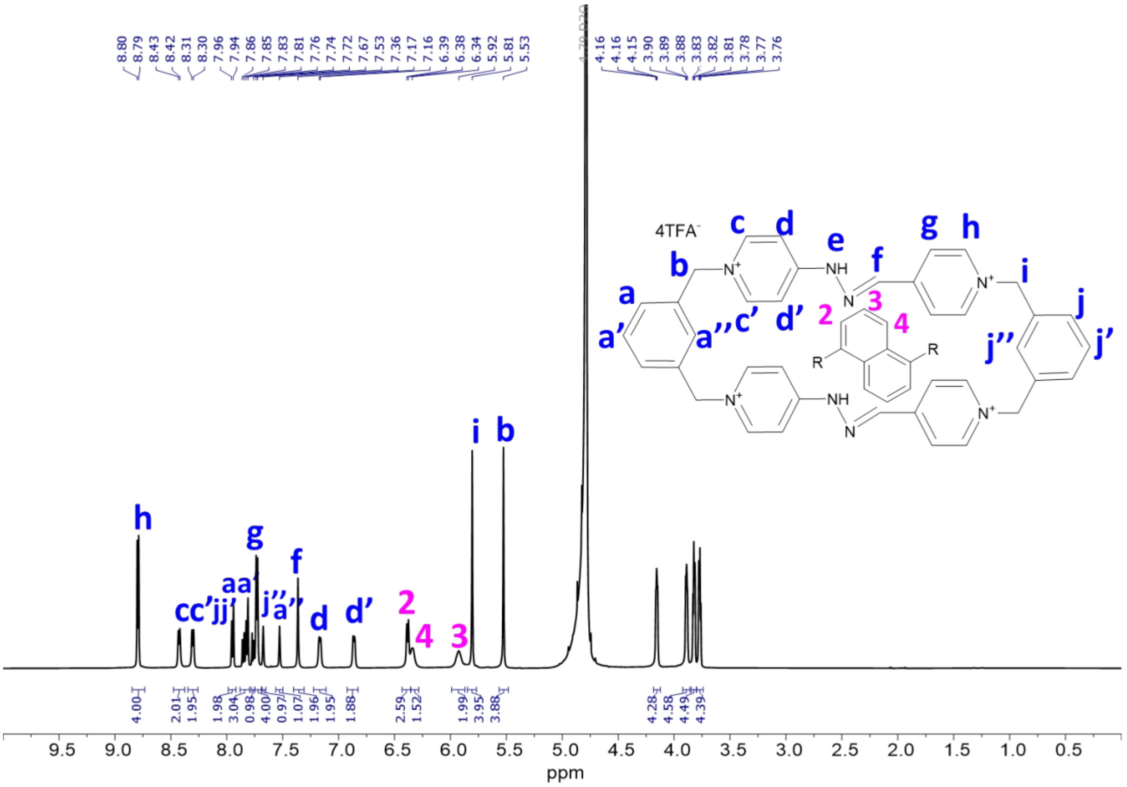

Figure S 128. ^{1}H NMR (500 MHz, D_2O) spectrum of the 1:1 inclusion complex **4**⊂$\mathbf{R_b}H_2$·4TFA at pD=5

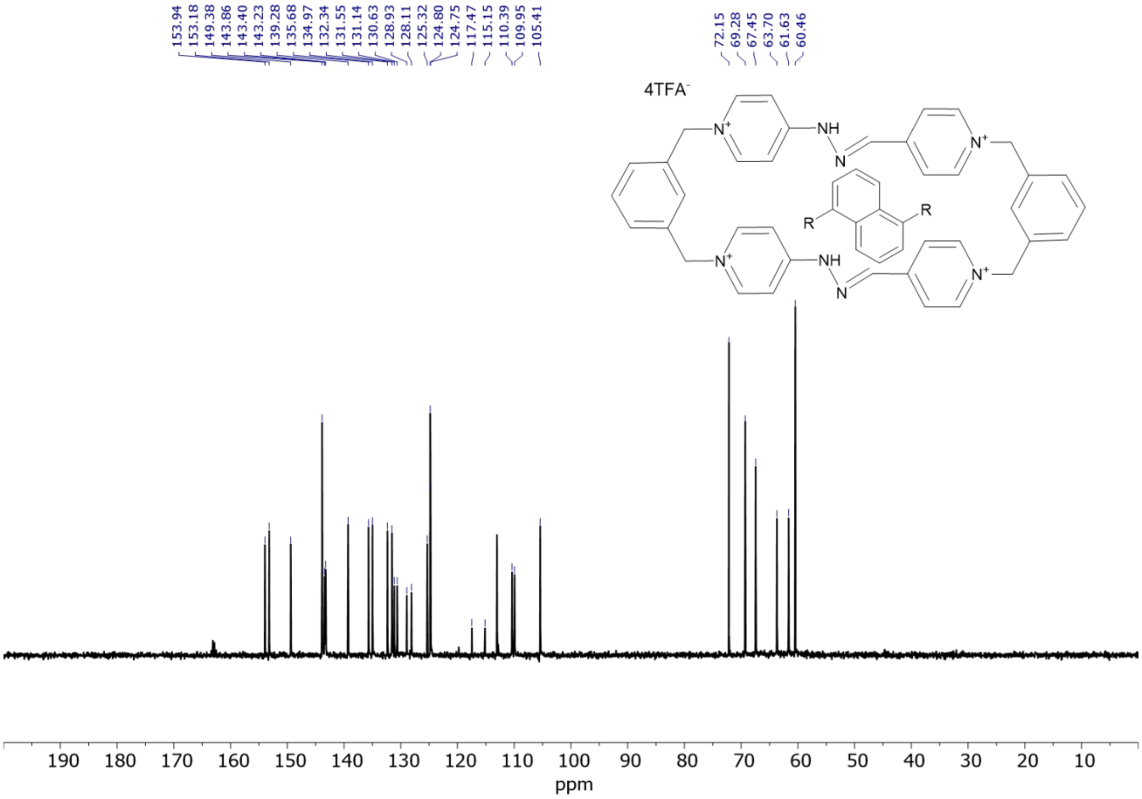

Figure S 129. $^{13}C\{^{1}H\}$ NMR (101 MHz, D_2O) spectrum of **4**⊂$\mathbf{R_b}H_2$·4TFA at pD=5

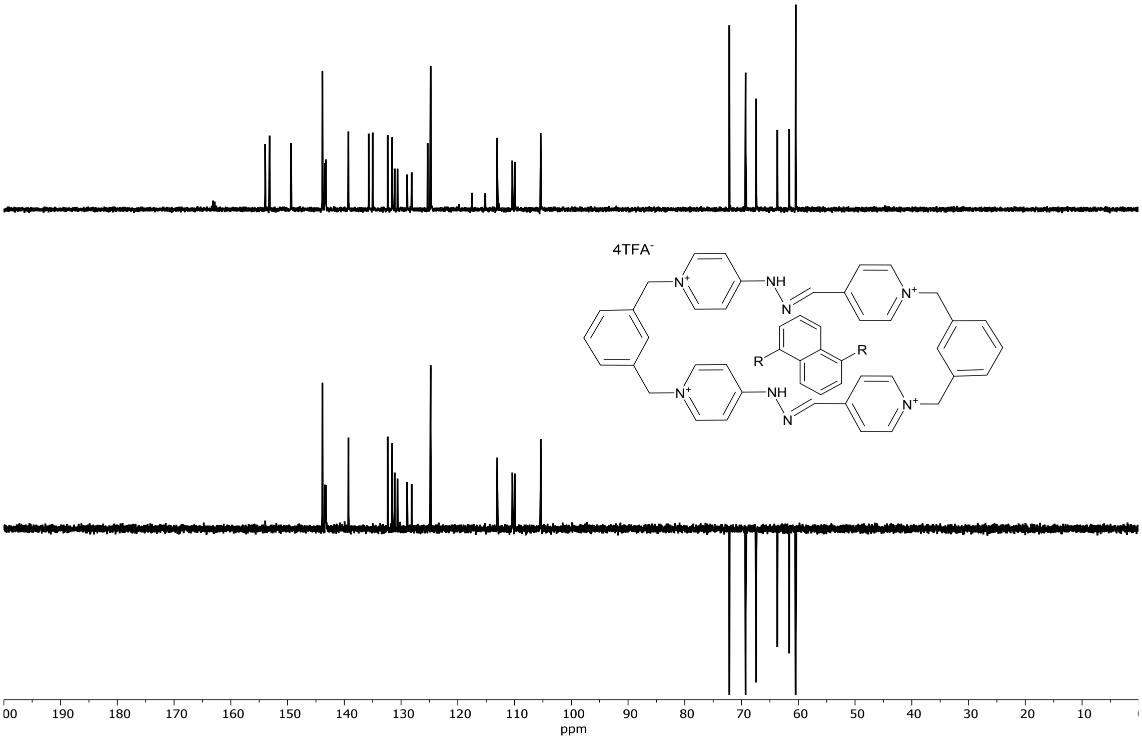

Figure S 130. $^{13}C\{^1H\}$ NMR (101 MHz, D_2O) spectrum (top) and DEPT-135 (101 MHz, D_2O) spectrum (bottom) of **4**⊂**R_b**H_2·4TFA at pD=5

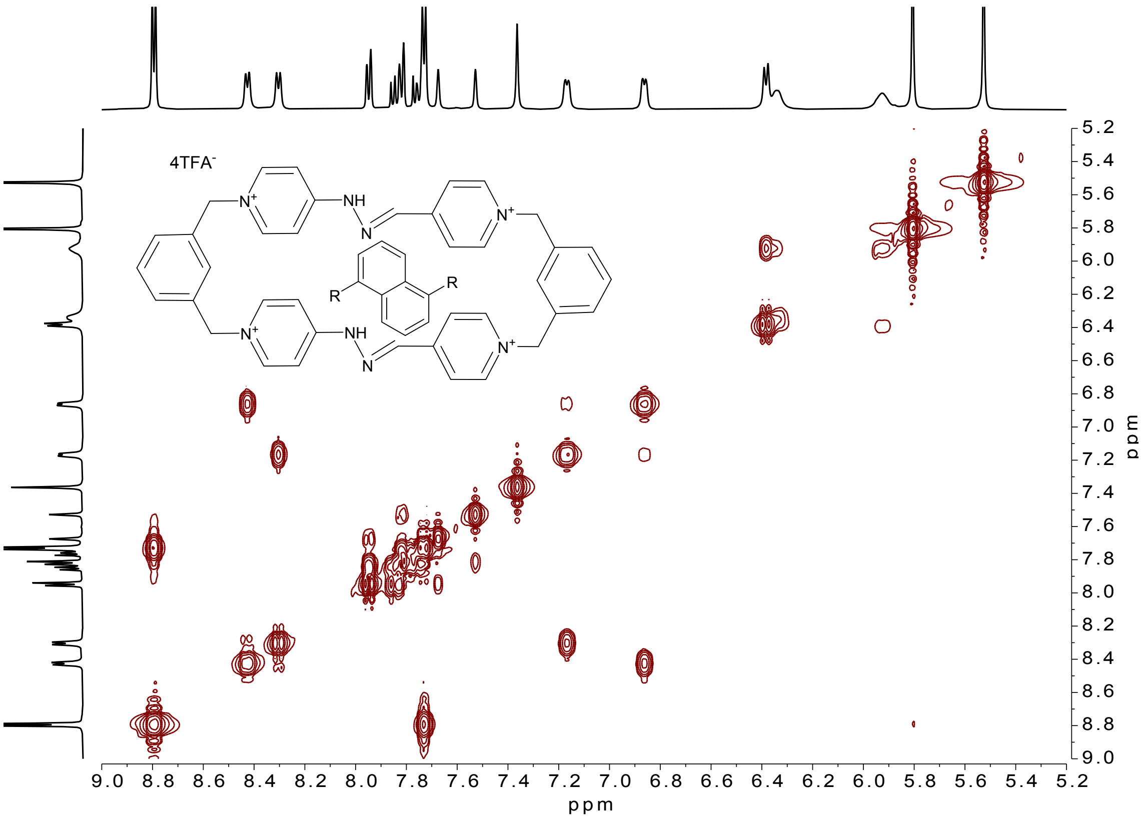

Figure S 131. ^1H-^1H COSY (500 MHz, D_2O) spectrum of **4**⊂**R_b**H_2·4TFA at pD=5

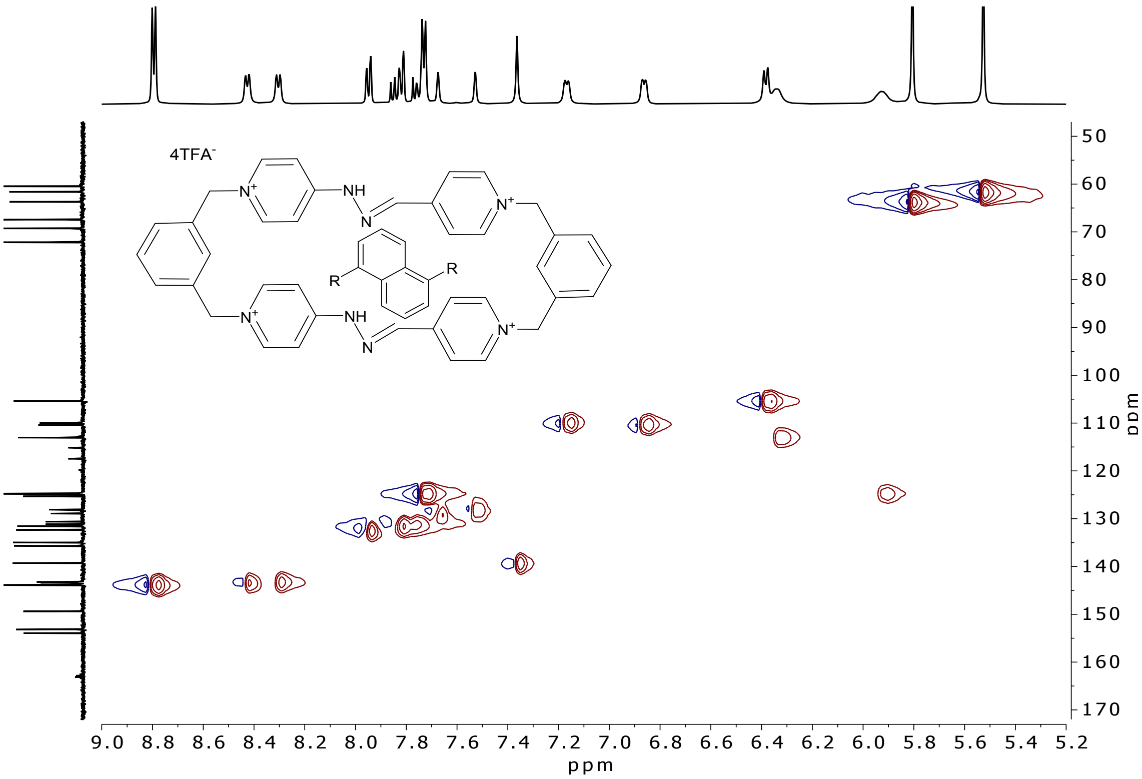

Figure S 132. ^{1}H-^{13}C HSQC (500 MHz/101 MHz, D_2O) spectrum of **4**⊂**R_b**H_2·4TFA at pD=5

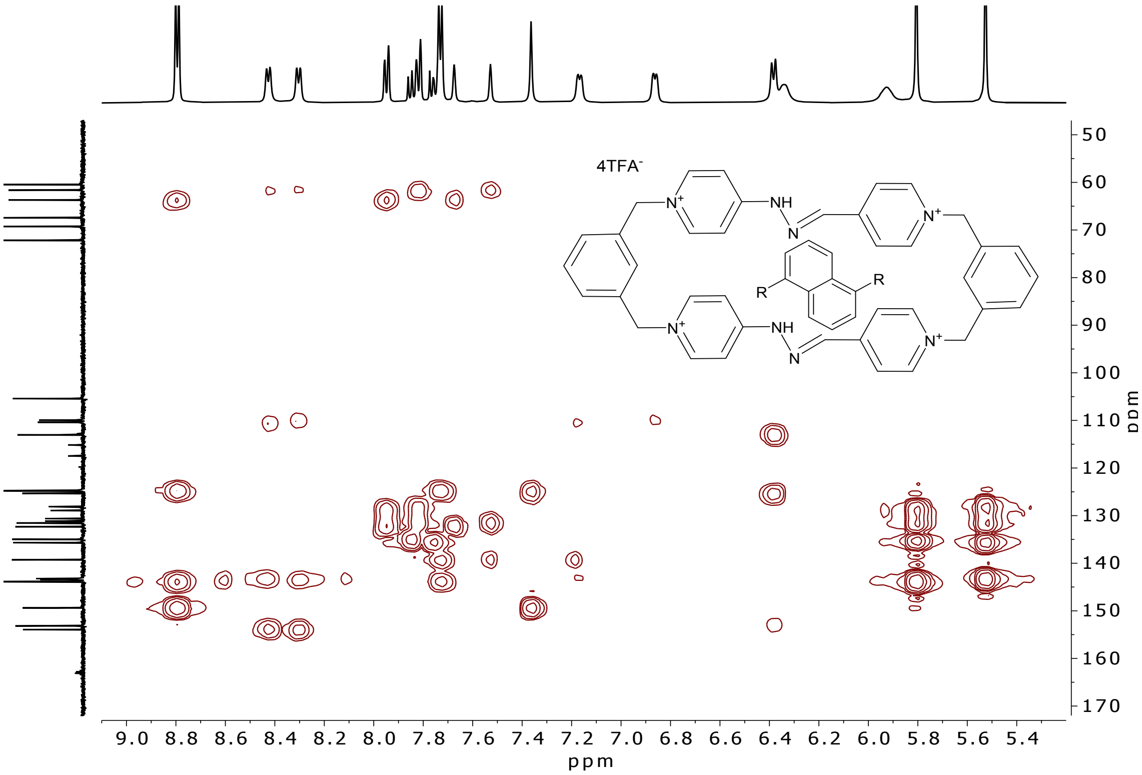

Figure S 133. ^{1}H-^{13}C HMBC (500 MHz/101 MHz, D_2O) spectrum of **4**⊂**R_b**H_2·4TFA at pD=5

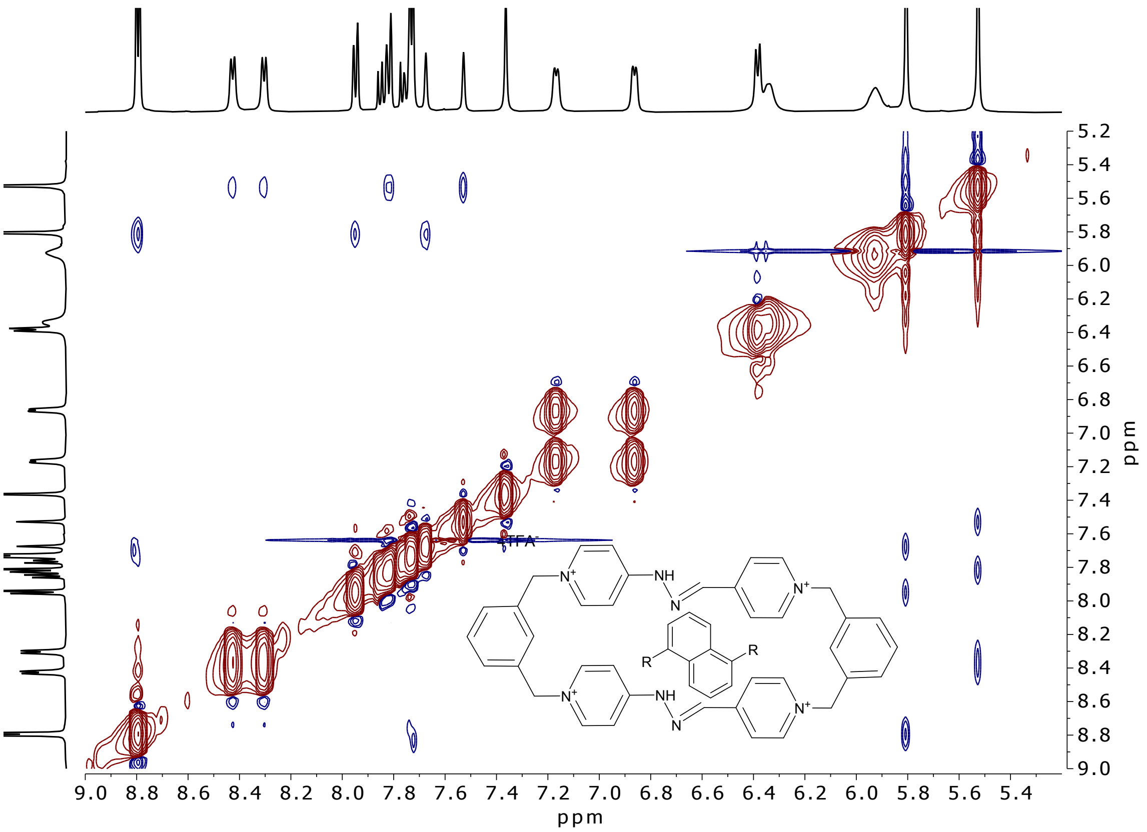

Figure S 134. ^{1}H-^{1}H NOESY (500 MHz, D_2O) spectrum of **4**$\subset$**R$_b$**H_2·4TFA at pD=5

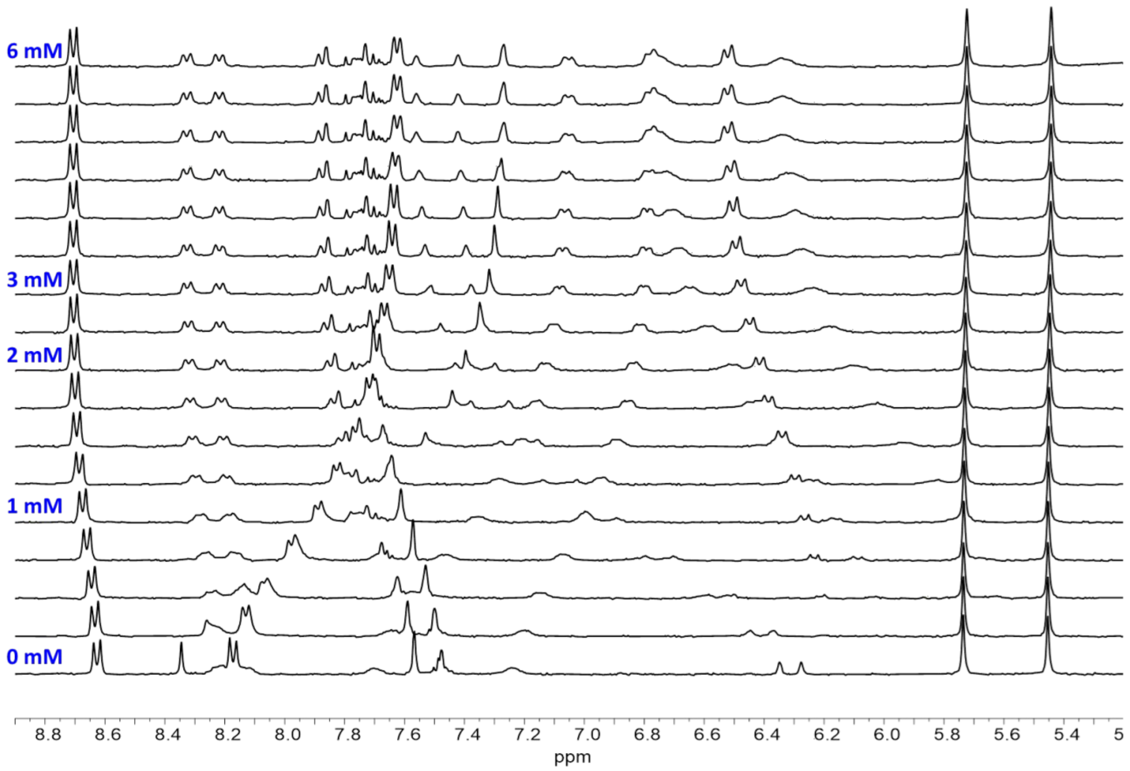

Figure S 135. ^{1}H NMR (300 MHz, D_2O) spectra of **R$_b$**H_2·4TFA (2 mM) upon titration with **4** (8 mM) at pD=5

The mechanism and adjustable parameters for the fitting task proposed for the fitting process equilibria, and introduced in the software Dynafit,[3] were the following:

$$\mathbf{R_b}\mathrm{H_2}^{4+} + \mathbf{4} \leftrightarrow \mathbf{4}\square\mathbf{R_b}\mathrm{H_2}^{4+}$$

[task]

task = fit ; simulate | design

data = equilibria ; progress | rates | equilibria | generic

[mechanism]

RB + DH <===> RBDH : Ka1 equilibria

[constants]

Ka1 = 0.029 ? ; µM-1

[concentrations]

RB = 2000; µM

[responses]

intensive

[data]

variable DH

plot titration

set Hf | resp RB = 8.3450 , RBDH = 7.2687 ?

set Hg | resp RB = 8.1617 , RBDH = 7.6150 ?

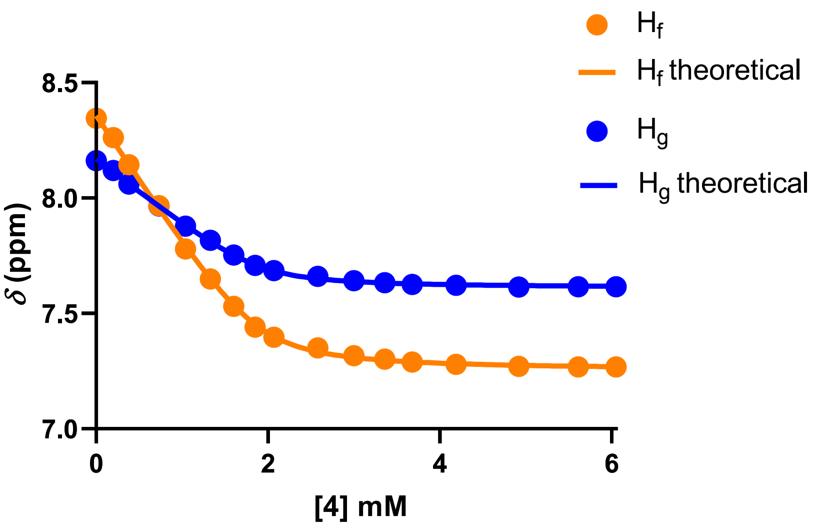

Figure S 136. Fitting of experimental data of the titration of $\mathbf{R_b}H_2$·4TFA (2 mM) with **4** (8 mM) at pD=5 (squares) for signals H_f (orange) and H_g (blue)

[3] P. Kuzmic, Program DYNAFIT for the Analysis of Enzyme Kinetic Data: Application to HIV Proteinase. *Anal. Biochem.* **1996**, *237*, 260-273.

Table S 4. Experimental data of the titration of $\mathbf{R_b}H_2$·4TFA (2 mM) with **4**(8 mM) at pD=5 for signals $\mathbf{H_f}$ and $\mathbf{H_g}$. The *p* value obtained from "run-of-sign" was 0.7[4]

[1,5-DHNc] (mM)	δ H_f (ppm)	Residual H_f	δ H_g (ppm)	Residual H_g
0.00	8.3450	-0.01277	8.1617	-0.00736
0.20	8.2623	0.01665	8.1198	0.00752
0.38	8.1447	-0.00091	8.0607	-0.00092
0.73	7.9666	0.011945	7.9659	0.00099
1.04	7.7797	-0.01246	7.8787	-0.00392
1.33	7.6490	-0.00185	7.8168	0.00575
1.60	7.5300	-0.00610	7.7536	0.00066
1.85	7.4412	-0.01108	7.7076	-0.00289
2.07	7.3966	-0.00274	7.6856	0.00192
2.58	7.3505	0.01717	7.6609	0.01065
3.00	7.3175	0.00850	7.6417	0.00377
3.36	7.3013	0.00436	7.6327	0.00088
3.68	7.2896	-0.00012	7.6258	-0.00236
4.19	7.2789	-0.00305	7.6217	-0.00253
4.92	7.2701	-0.00498	7.6149	-0.00585
5.61	7.2687	-0.00226	7.6150	-0.00366
6.05	7.2687	-0.00032	7.6150	-0.00268

[4] For the evaluation of goodness-of-fit, Dynafit software employs the analysis of the residuals through the runs-of-sign test. Thus, by analyzing the randomness of the residuals plot against the independent variable (concentration) and the *p* parameter (> 0.05), we can confirm the appropriateness of the fitted model. See, for instance: P. Kuzmic, T. Lorenz, J. Reinstein, Analysis of residuals from enzyme kinetic and protein folding experiments in the presence of correlated experimental noise. *Anal. Biochem.*, **2009**, *395*, 1-7.

2.1.2. 4⊂R_bH_2□4TFA at pD 11

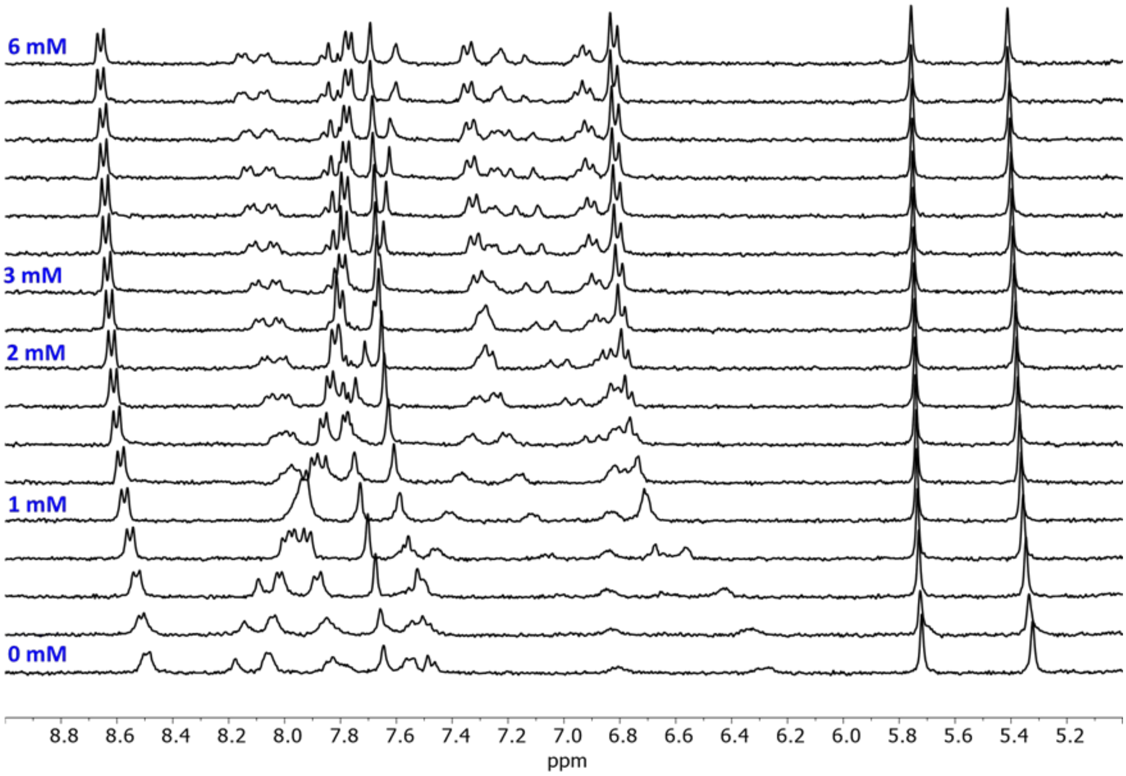

Figure S 137. ^{1}H NMR (300 MHz, D_2O) spectra of **R$_b$**H_2·4TFA (2 mM) upon titration with **4** (6 mM) at pD=11

The mechanism and adjustable parameters for the fitting task proposed for the fitting process equilibria, and introduced on the software Dynafit,[3] were the following:

$$\mathbf{R_b}H_2^{2+} + \mathbf{4} \leftrightarrow \mathbf{4}□\mathbf{R_b}H_2^{2+}$$

[task]

task = fit ; simulate | design

data = equilibria ; progress | rates | equilibria | generic

[mechanism]

RB + DH <===> RBDH : Ka1 equilibria

[constants]

Ka1 = 0.029 ? ; µM-1

[concentrations]

RB = 2000; µM

[responses]

intensive

[data]

variable DH

plot titration

set Hf | resp RB = 8.1750 , RBDH = 7.6030 ?

set Hg | resp RB = 8.0600 , RBDH = 7.7720 ?

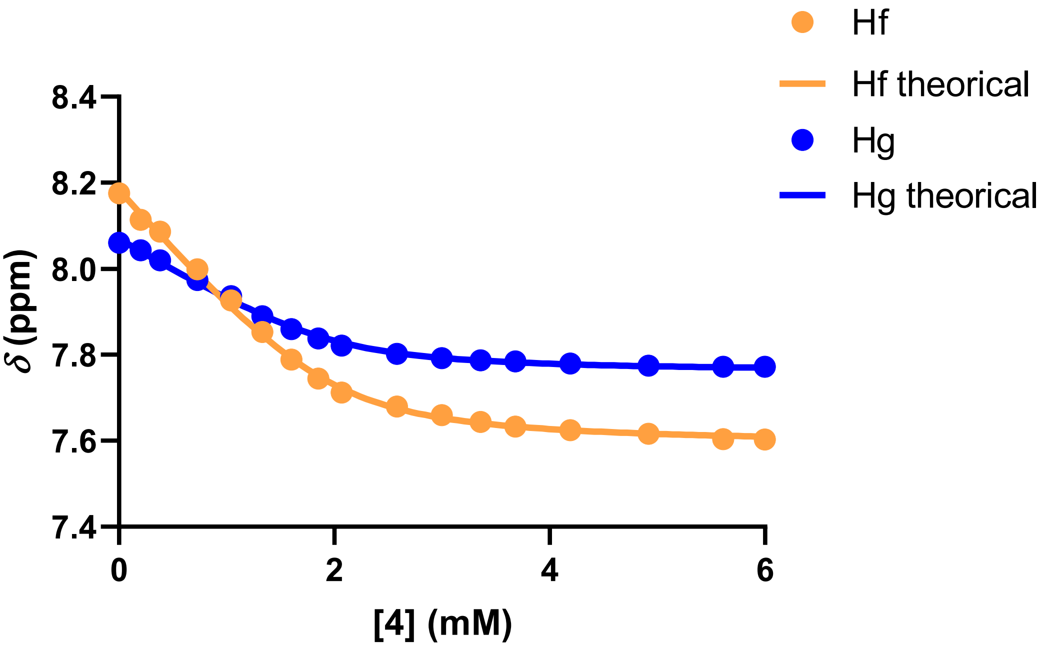

Figure S 138. Fitting of experimental data of the titration of **R$_b$**H$_2$·4TFA (2 mM) with **4** (6 mM) at pD=11 (squares) for signals H$_f$ (orange) and **H$_g$** (blue)

Table S 5. Experimental data of the titration of **R$_b$**H$_2$·4TFA (2 mM) with **4**(8 mM) at pD=5 for signals H$_f$ and **H$_g$**. The *p* value obtained from "run-of-sign" was 0.05[4]

[1,5-DHNc] (mM)	*δ* H$_f$ (ppm)	Residual H$_f$	*δ* H$_g$ (ppm)	Residual H$_g$
0,00	8,1750	-0,00796	8,0600	-0,00928
0,20	8,1140	-0,01402	8,0430	0,00240
0,38	8,0860	0,00663	8,0200	0,00482
0,73	7,9990	0,01135	7,9740	0,00671
1,04	7,9260	0,01487	7,9360	0,00867
1,33	7,8530	0,00732	7,8900	-0,00315
1,60	7,7890	-0,00325	7,8600	-0,00525
1,85	7,7450	-0,00585	7,8380	-0,00563
2,07	7,7120	-0,00939	7,8210	-0,00724
2,58	7,6800	0,00478	7,8020	-0,00213
3,00	7,6600	0,00673	7,7920	-0,000671
3,36	7,6440	0,002969	7,7870	0,00072
3,68	7,6330	-0,000243	7,7850	0,002787
4,19	7,6250	0,000485	7,7800	0,002345

4,92	7,6160	-0,000525	7,7750	0,001517
5,61	7,6040	-0,007619	7,7720	0,001079
6,00	7,6030	-0,006286	7,7720	0,002298

2.2. Verification of the non-formation of $4{\subset}\mathbf{R_c}H_2$□4TFA

Equimolar 2.5 mM solutions of $\mathbf{R_c}H_2$□4TFA and the corresponding guest **4** were prepared in 20 mM phosphate buffer solution pD=5 at room temperature, and the corresponding NMR recorded immediately after.

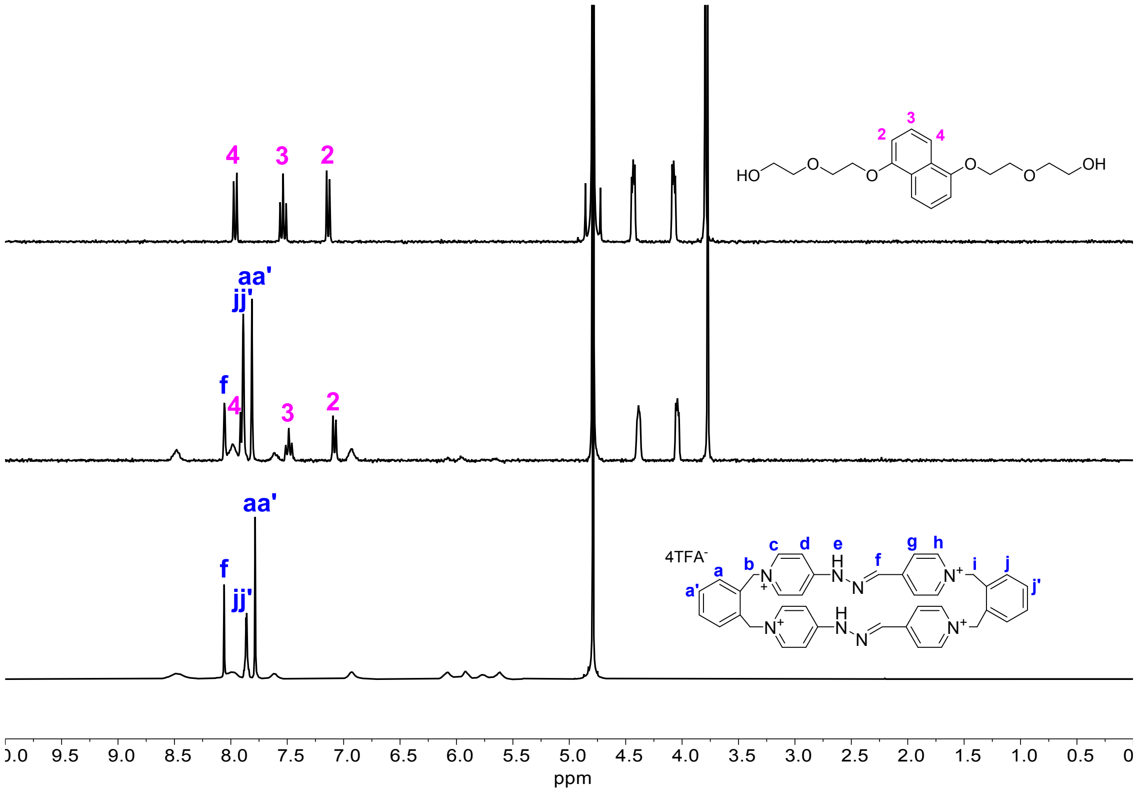

Figure S 139. ^{1}H NMR spectra comparison between the guest **4** (top), the 1:1 equimolar solution of **4** and $\mathbf{R_c}H_2$·4TFA at pD=5 (middle) and the host $\mathbf{R_c}H_2$·4TFA (bottom)

2.3. Synthesis and characterization data of $\mathbf{4_2{\subset}R_dH_2}$□4TFA

Equimolar 1.3 mM solutions of $\mathbf{R_d}H_2$□4TFA and the corresponding guest $\mathbf{4_2}$ were prepared in 20 mM phosphate buffer solution pD=5 at room temperature, and the corresponding NMR recorded immediately after.

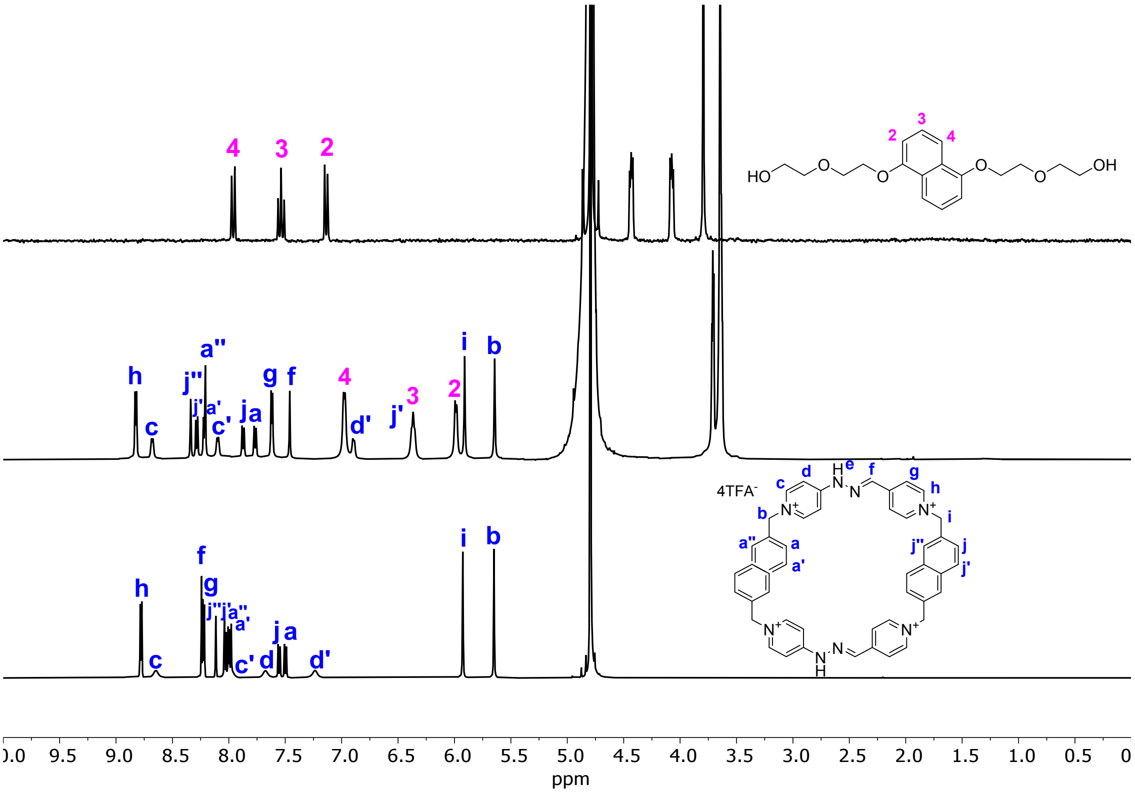

Figure S 140. ^{1}H NMR spectra comparison between the guest $\mathbf{4_2}$ (top), the 1:1 inclusion complex $\mathbf{4_2{\subset}R_d}H_2$□4TFA at pD=5 (middle) and the host $\mathbf{R_d}H_2$□4TFA (bottom)

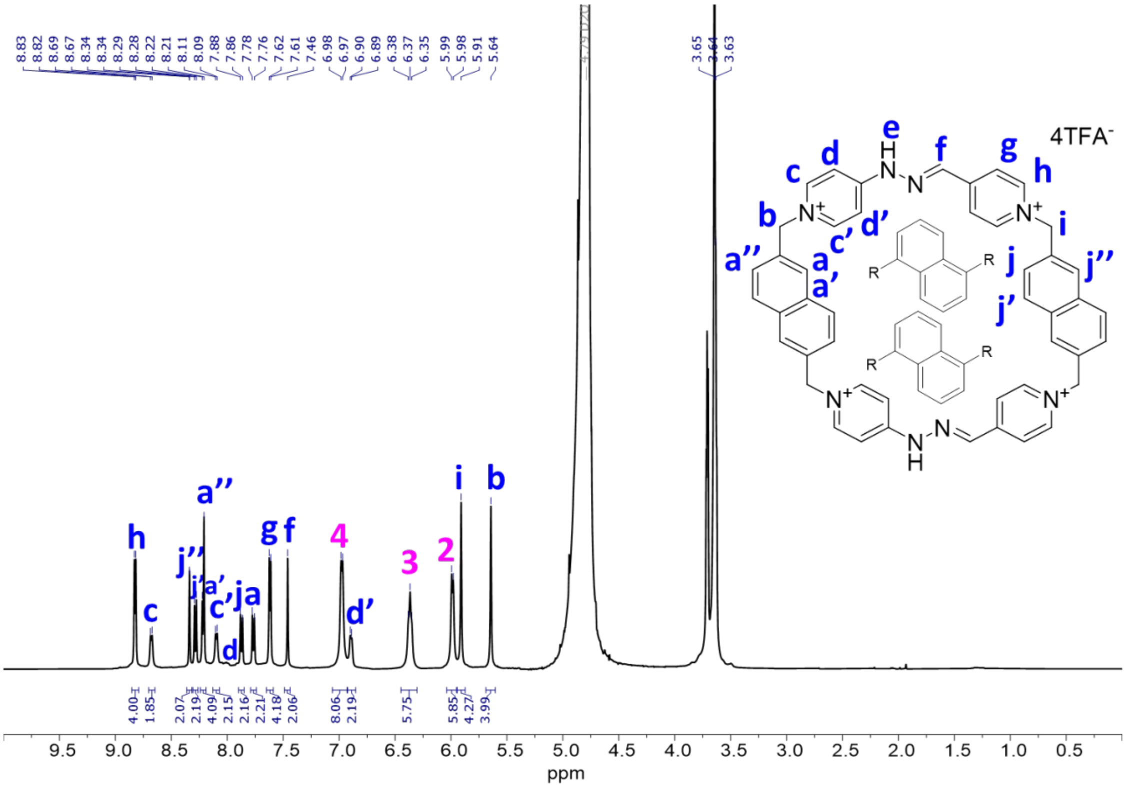

Figure S 141. ^{1}H NMR (500 MHz, D_2O) spectrum of $\mathbf{4_2}\subset\mathbf{R_d}H_2$□4TFA at pD=5

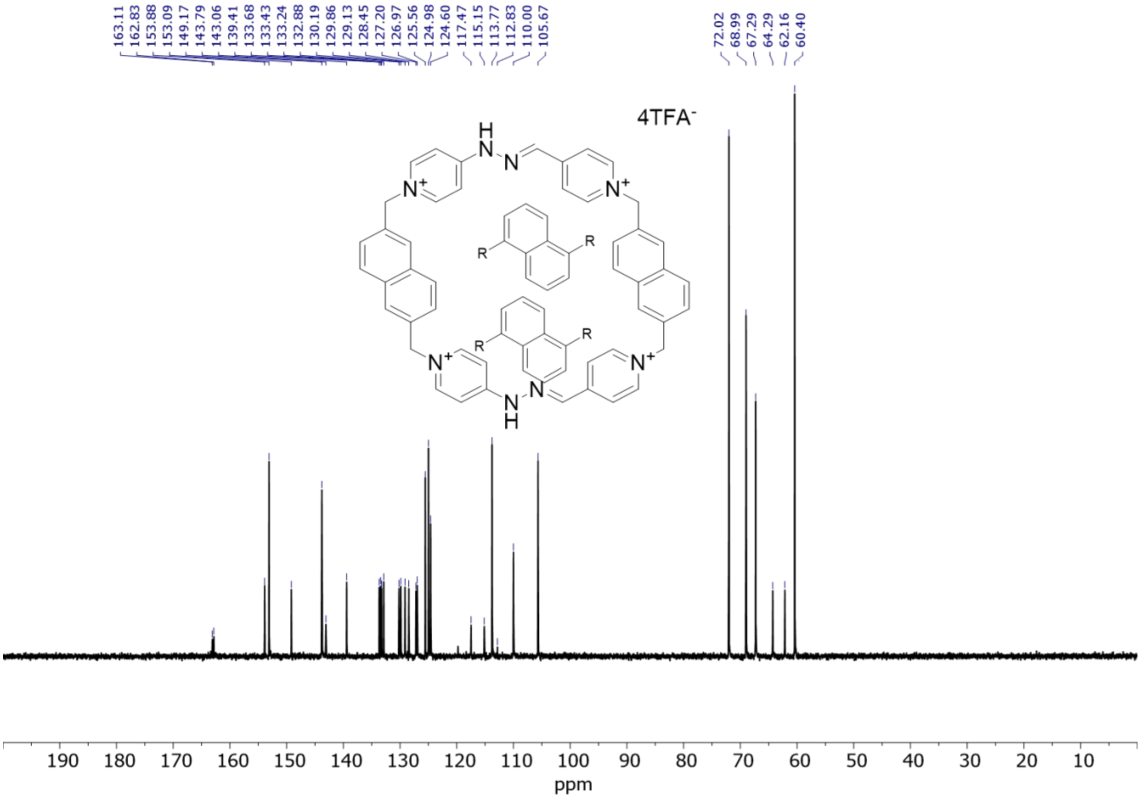

Figure S 142. $^{13}C\{^{1}H\}$ NMR (126 MHz, CD_3CN) spectrum of $\mathbf{4_2}\subset\mathbf{R_d}H_2$□4TFA at pD=5

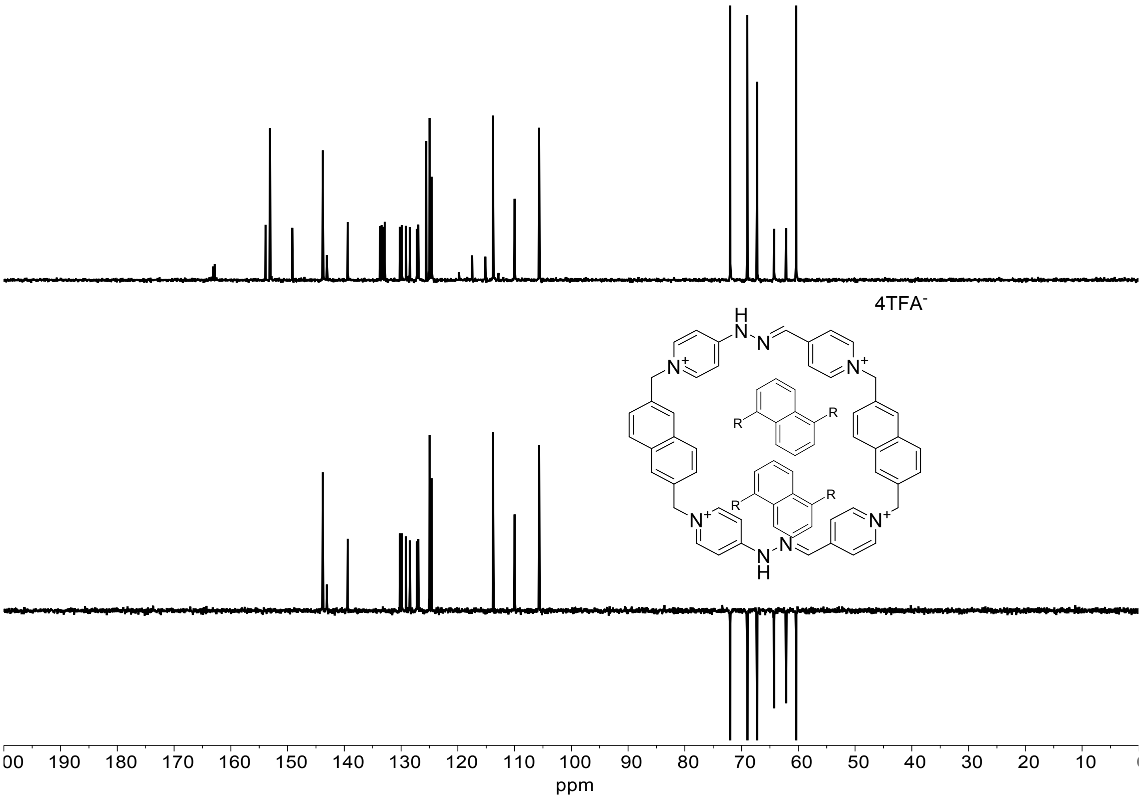

Figure S 143. $^{13}C\{^{1}H\}$ NMR (126 MHz, CD_3CN) spectrum (top) and DEPT-135 (126 MHz, CD_3CN) spectrum (bottom) of $\mathbf{4_2}\subset\mathbf{R_d}H_2$□4TFA at pD=5

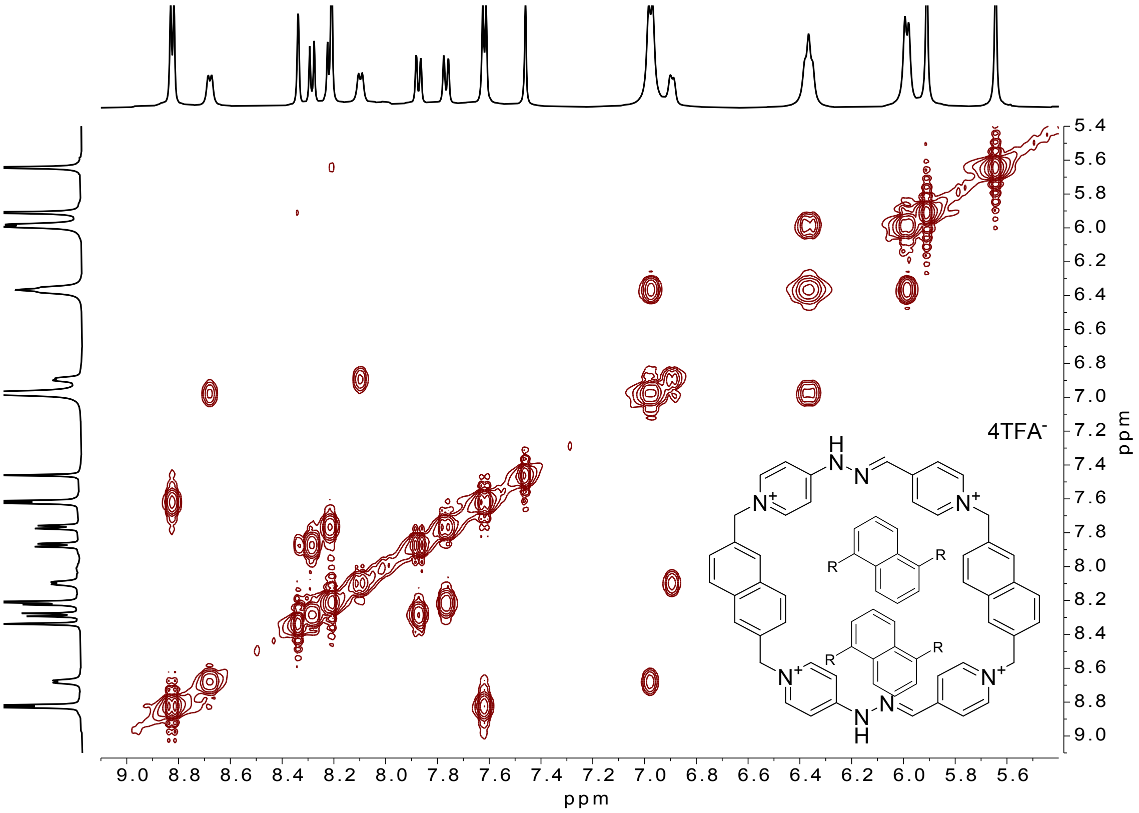

Figure S 144. ^{1}H-^{1}H COSY (500 MHz, D_2O) spectrum of $\mathbf{4_2}\subset\mathbf{R_d}H_2$□4TFA at pD=5

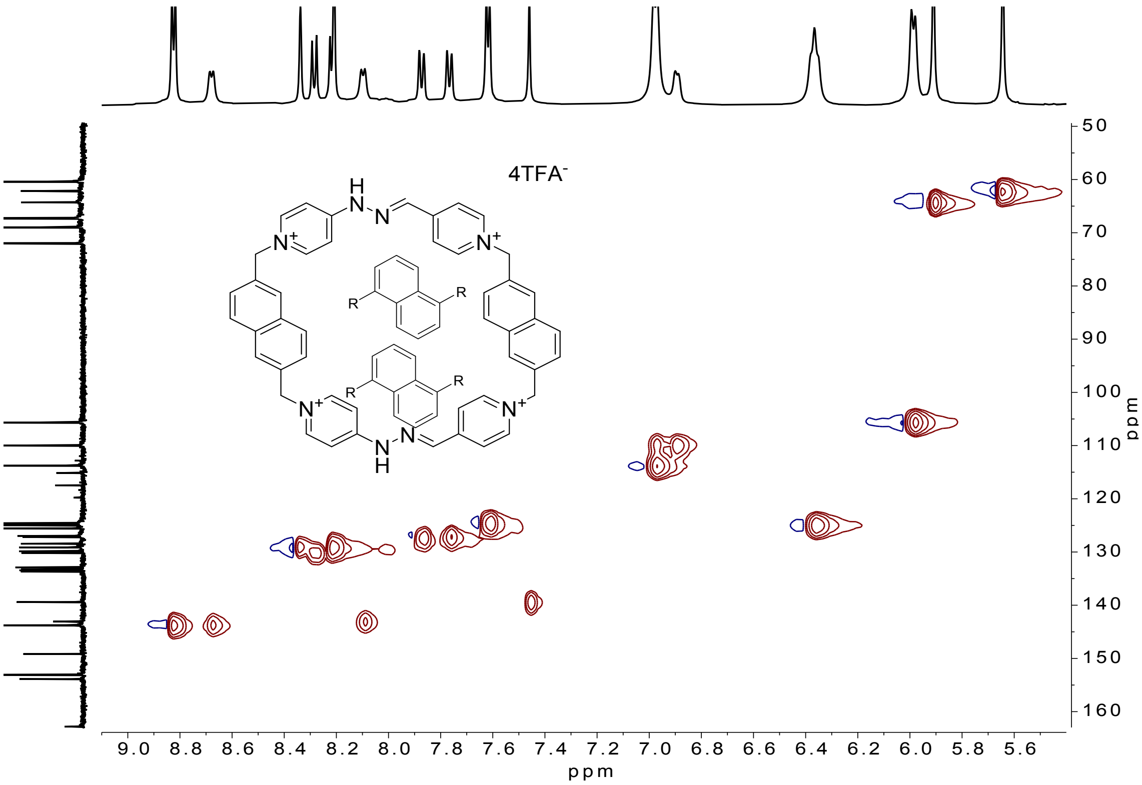

Figure S 145. ^{1}H-^{13}C HSQC (500 MHz/126 MHz, D_2O) spectrum of $\mathbf{4_2\subset R_d}H_2$□4TFA at pD=5

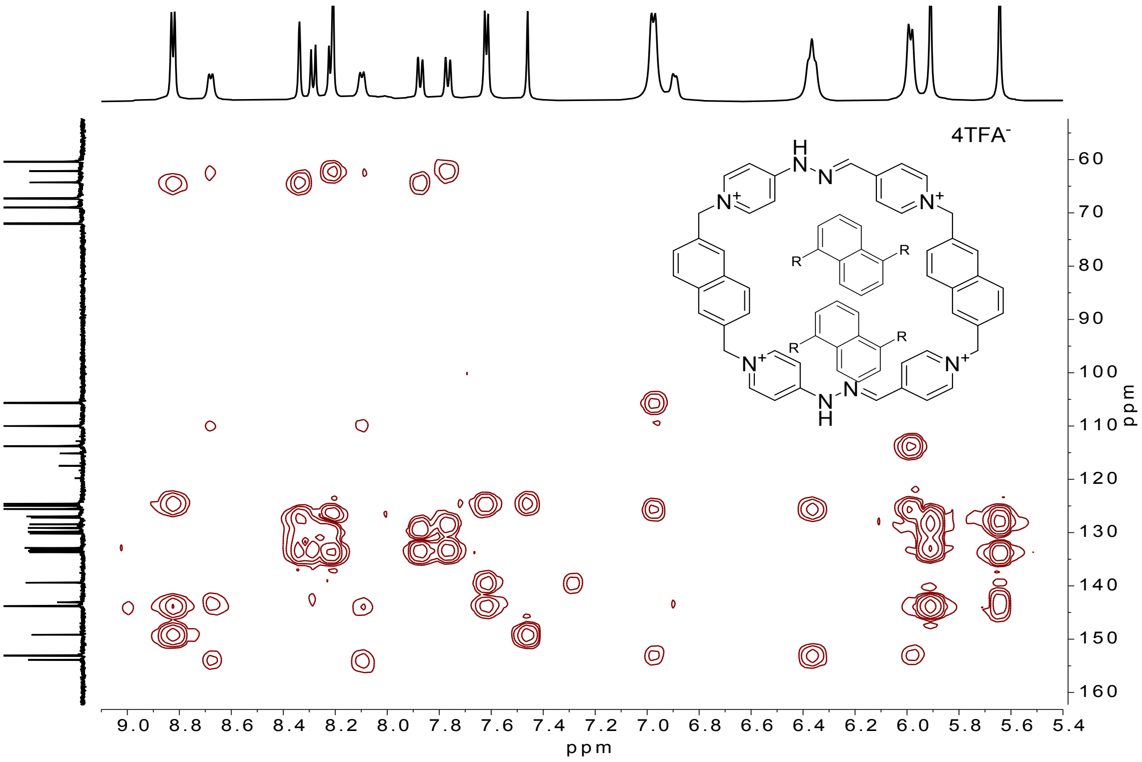

Figure S 146. ^{1}H-^{13}C HMBC (500 MHz/126 MHz, D_2O) spectrum of $\mathbf{4_2\subset R_d}H_2$□4TFA at pD=5

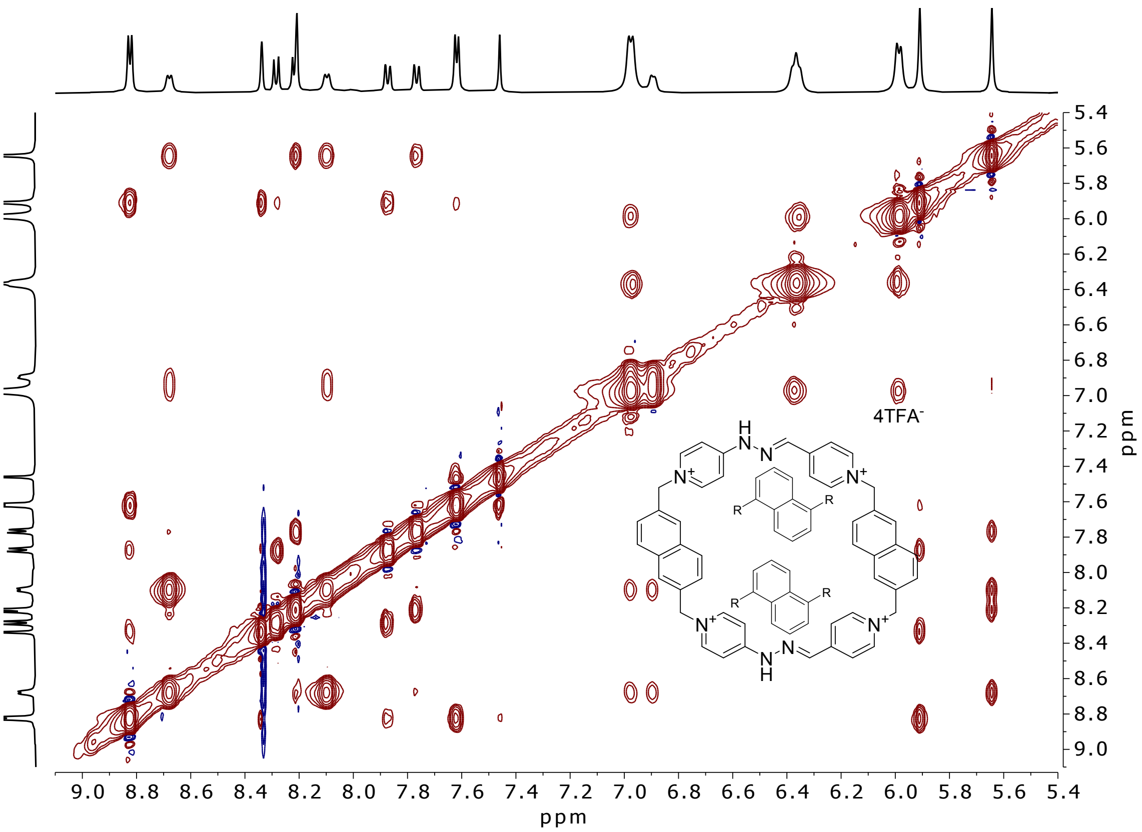

Figure S 147. ^{1}H-^{1}H NOESY (500 MHz, D_2O) spectrum of $\mathbf{4_2}\subset\mathbf{R_d}H_2$□4TFA at pD=5

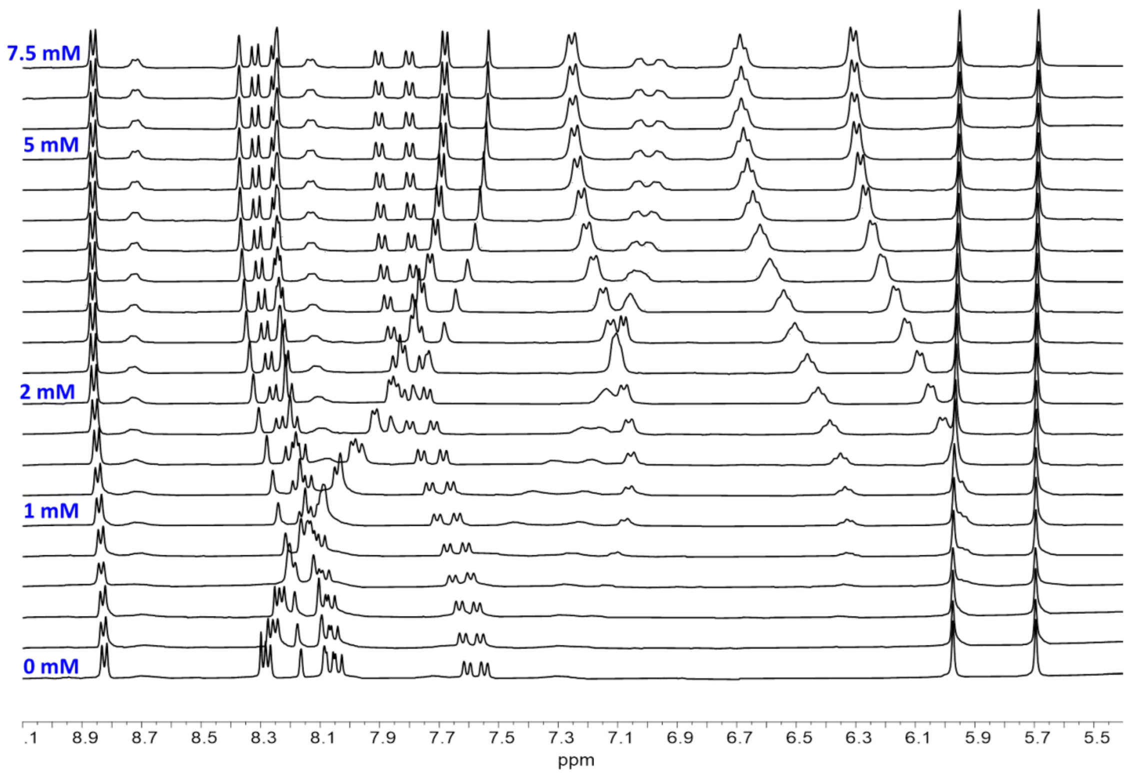

Figure S 148. ^{1}H NMR (400 MHz, D_2O) spectra of $\mathbf{R_d}H_2$□4TFA (1.3 mM) upon titration with **4** (15 mM) at pD=5

The mechanism and adjustable parameters for the fitting task proposed for the fitting process equilibria, and introduced on the software Dynafit,[3] were the following:

$$\mathbf{R_d}H_2^{4+} + \mathbf{4} \leftrightarrow \mathbf{4}\square\mathbf{R_d}H_2^{4+}$$

$$\mathbf{4}\square\mathbf{R_d}H_2^{4+} + \mathbf{4} \leftrightarrow \mathbf{4_2}\square\mathbf{R_d}H_2^{4+}$$

```
[task]
task = fit
data =  equilibria
[mechanism]
RB + DH <===>  RBDH: Ka1 equilibria
RBDH + DH <===>  RBDHDH : Ka2 equilibria
[constants]
Ka1 = 0.01?
Ka2 = 0.001?
[concentrations]
RB = 1300; µM
[responses]
intensive
[data]
variable DH
plot titration
set Hf   | resp RB = 8.2955   ,RBDHDH = 7.5343 ?
set Hg  | resp RB = 8.2749  , RBDHDH = 7.6813 ?
```

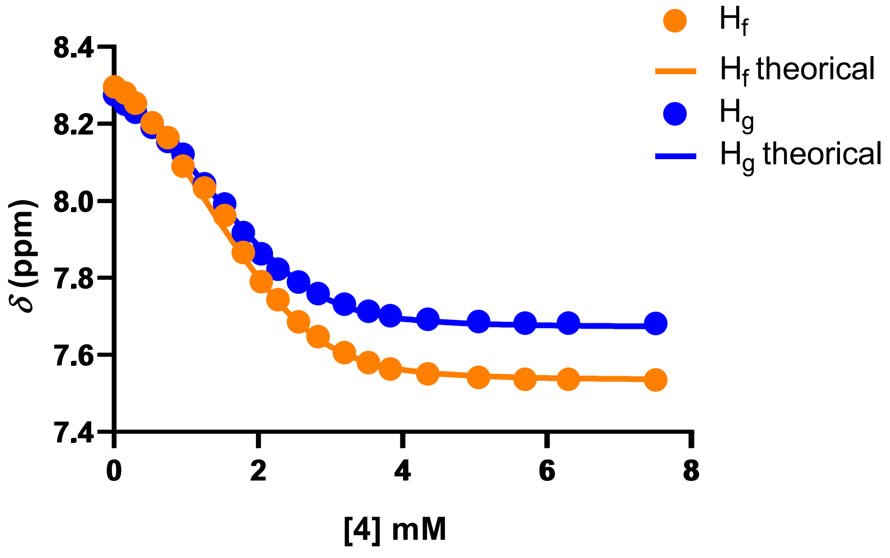

Figure S 149. Fitting of experimental data of the titration of $\mathbf{R_d}H_2\square$4TFA (1.3 mM) with **4** (15 mM) at pD=5 (squares) for signals $\mathbf{H_f}$ (orange) and $\mathbf{H_g}$ (blue)

Table S 6. Experimental data of the titration of $\mathbf{R_d}H_2$□4TFA (1.3 mM) with **4** (15 mM) at pD=5 for signals H_f and H_g. The *p* value obtained from "run-of-sign" was 0.05[4]

[1,5-DHNc] (mM)	δ H_f (ppm)	Residual H_f	δ H_g (ppm)	Residual H_g
0.0	8.2955	0.00000	8.2749	0.00000
0.1	8.2755	-0.00722	8.2508	-0.01398
0.3	8.2529	-0.00562	8.2292	-0.01642
0.5	8.2022	-0.00598	8.1905	-0.01526
0.7	8.1640	0.01180	8.1540	-0.00745
1.0	8.0897	-0.00511	8.1201	0.00409
1.2	8.0332	0.02333	8.0434	-0.00535
1.5	7.9614	0.03112	7.9909	0.00520
1.8	7.8655	0.00717	7.9167	-0.01211
2.0	7.7900	-0.00542	7.8625	-0.01648
2.3	7.7427	0.00036	7.8225	-0.01444
2.6	7.6852	-0.00096	7.7885	-0.00397
2.8	7.6467	0.00187	7.7588	-0.00096
3.2	7.6055	0.00083	7.7311	0.00314
3.5	7.5792	-0.00237	7.7129	0.00324
3.8	7.5627	-0.00528	7.7010	0.00209
4.3	7.5502	-0.00405	7.6925	0.00446
5.0	7.5416	-0.00386	7.6865	0.00542
5.7	7.5364	-0.00507	7.6823	0.00438
6.3	7.5361	-0.00321	7.6823	0.00610
7.5	7.5343	-0.00269	7.6813	0.00693

To corroborate that the fitting does not reflect a non-cooperative binding mode, the mechanism and adjustable parameters for the fitting task proposed for the fitting process equilibria, and introduced on the software Dynafit,[3] were the following:

$$\mathbf{R_d}H_2^{4+} + \mathbf{4} \leftrightarrow \mathbf{4}\square\mathbf{R_d}H_2^{4+}$$

$$\mathbf{4}\square\mathbf{R_d}H_2^{4+} + \mathbf{4} \leftrightarrow \mathbf{4_2}\square\mathbf{R_d}H_2^{4+}$$

```
[task]
task = fit  ;  simulate | design
data =  equilibria ;  progress | rates | equilibria | generic
[mechanism]
RB + DH <===>  RBDH: Ka1 equilibria
RBDH + DH <===>  RBDHDH : Ka2 equilibria
```

[constants]

Ka1 = 4 * Ka2

Ka2 = 0.001?

[concentrations]

RB = 1300; µM

[responses]

intensive

[data]

variable DH

plot titration

set Hf | resp RB = 8.2955 ,RBDHDH = 7.5343 ?

set Hg | resp RB = 8.2749 , RBDHDH = 7.6813 ?

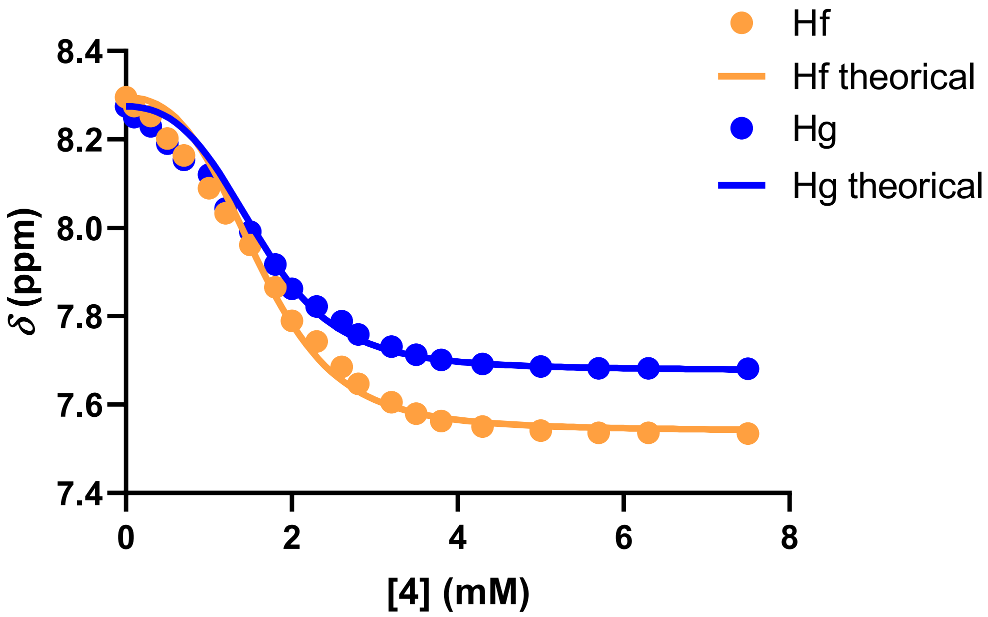

Figure S 150. Fitting of experimental data of the titration of $\mathbf{R_d}H_2$□4TFA (1.3 mM) with **4** (15 mM) at pD=5 (squares) for signals H_f (orange) and $\mathbf{H_g}$ (blue) to a non-cooperative binding system

Table S 7. Experimental data of the titration of $\mathbf{R_d}H_2$□4TFA (1.3 mM) with **4** (15 mM) at pD=5 for signals H_f and $\mathbf{H_g}$ to a non-cooperative binding system. The *p* value obtained from "run-of-sign" was 0.000071 (< 0.05)[4]

[1,5-DHNc] (mM)	δ H_f (ppm)	Residual H_f	δ H_g (ppm)	Residual H_g
0.0	8.2955	0.00000	8.2749	0.00000
0.1	8.2755	-0.01779	8.2508	-0.02234
0.3	8.2529	-0.03330	8.2292	-0.03834
0.5	8.2022	-0.05968	8.1905	-0.05780
0.7	8.1640	-0.05672	8.1540	-0.06172

1.0	8.0897	-0.07459	8.1201	-0.05096
1.2	8.0332	-0.02666	8.0434	-0.04501
1.5	7.9614	0.01130	7.9909	-0.01065
1.8	7.8655	0.01332	7.9167	-0.00736
2.0	7.7900	0.01612	7.8625	0.00041
2.3	7.7427	0.02709	7.8225	0.00652
2.6	7.6852	0.02291	7.7885	0.01472
2.8	7.6467	0.01868	7.7588	0.01214
3.2	7.6055	0.00788	7.7311	0.00850
3.5	7.5792	-0.00142	7.7129	0.00375
3.8	7.5627	-0.00769	7.7010	-0.00005
4.3	7.5502	-0.00923	7.6925	0.00012
5.0	7.5416	-0.01015	7.6865	0.00020
5.7	7.5364	-0.01158	7.6823	-0.00101
6.3	7.5361	-0.00972	7.6823	0.00069
7.5	7.5343	-0.00911	7.6813	0.00160

2.4. Synthesis and characterization data of $4_2 \subset R_eH_2$·4TFA

Equimolar 1.3 mM solutions of $\mathbf{R_eH_2}$·4TFA and the corresponding guest **4** were prepared in 20 mM phosphate buffer solution pD=5 at room temperature, and the corresponding NMR recorded immediately after.

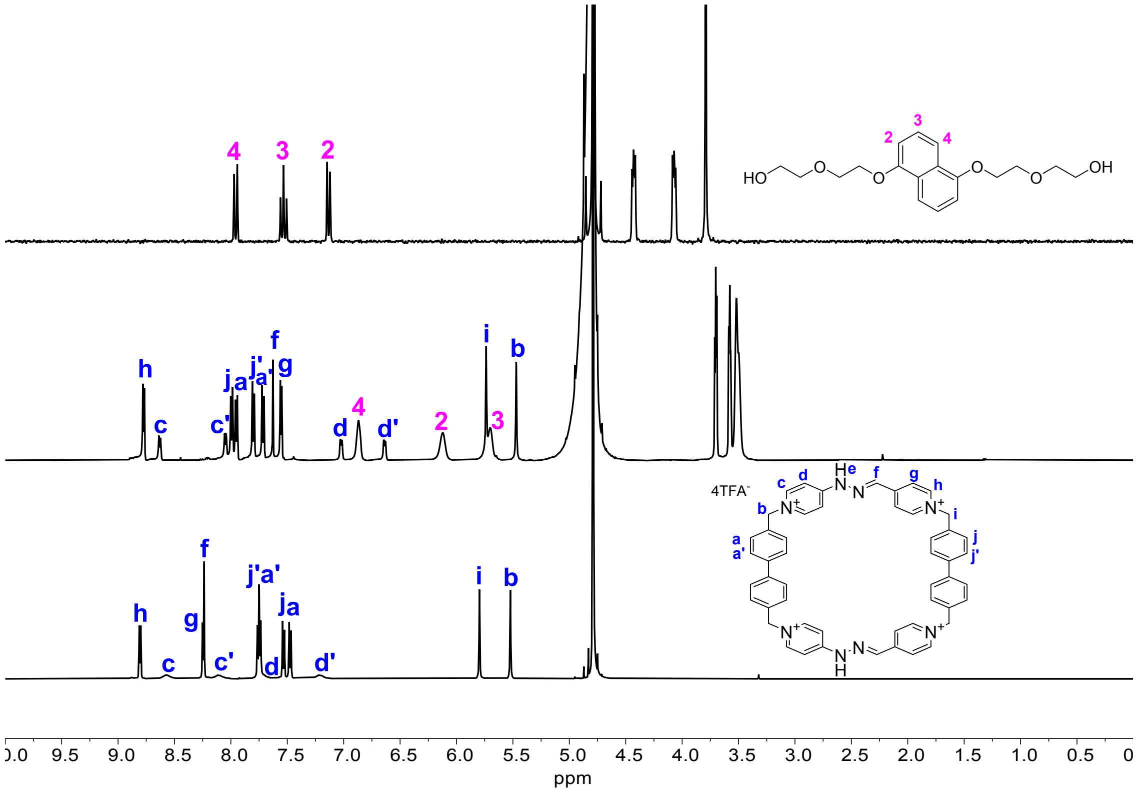

Figure S 151. ^{1}H NMR spectra comparison between the guest **4** (top), $\mathbf{4_2 \subset R_e}H_2$·4TFA at pD=5 (middle) and $\mathbf{R_e}H_2$·4TFA (bottom)

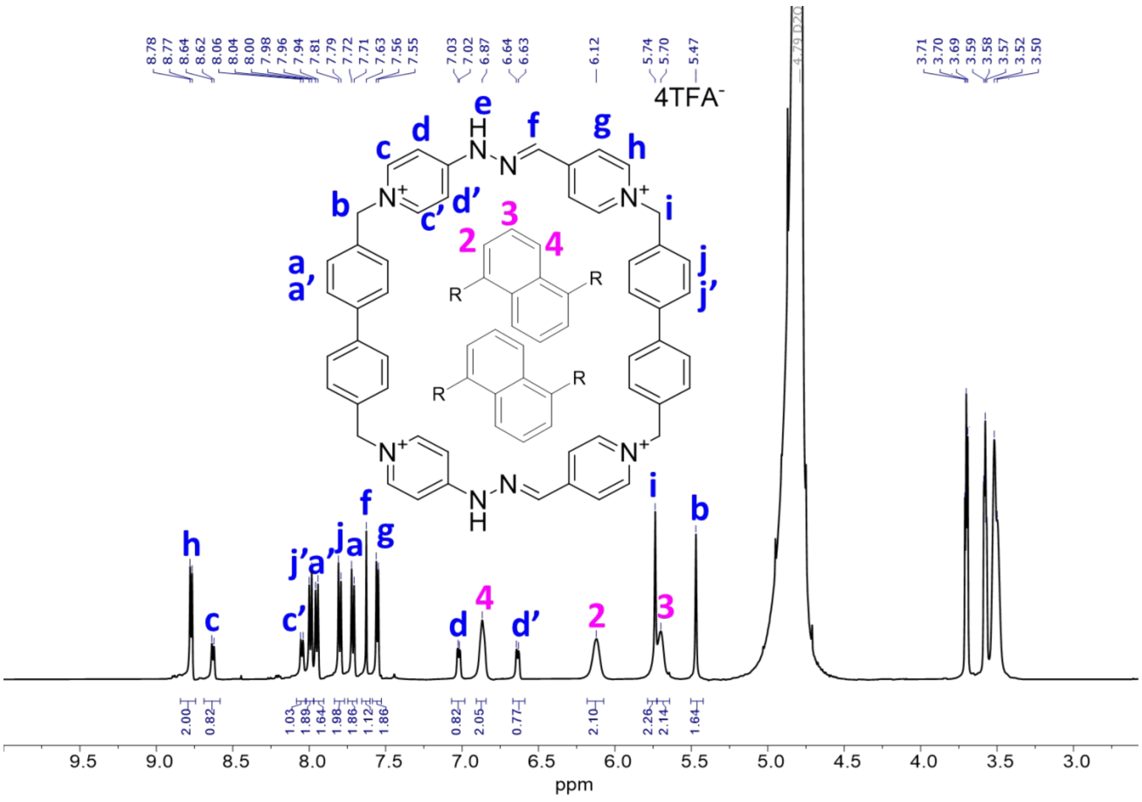

Figure S 152. ^{1}H NMR (500 MHz, D_2O) spectrum of $\mathbf{4_2}\subset\mathbf{R_e}H_2\cdot$4TFA at pD=5

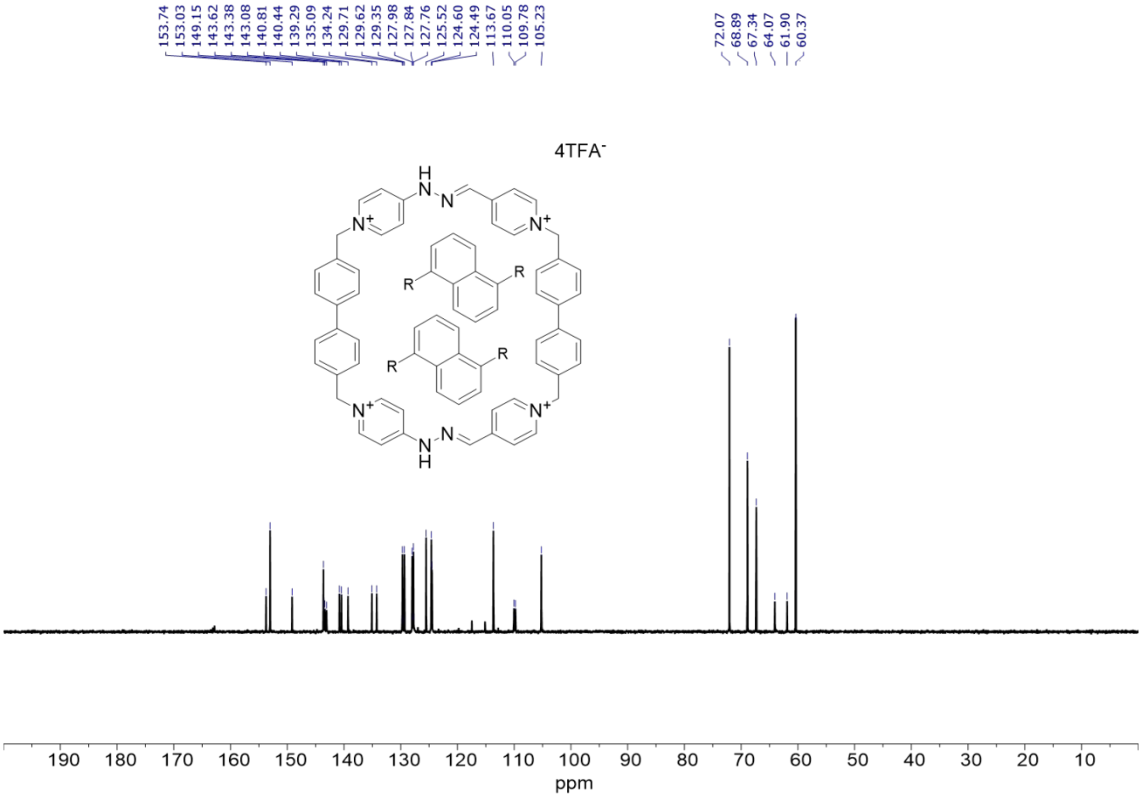

Figure S 153. $^{13}C\{^{1}H\}$ NMR (126 MHz, D_2O) spectrum of $\mathbf{4_2}\subset\mathbf{R_e}H_2\cdot$4TFA at pD=5

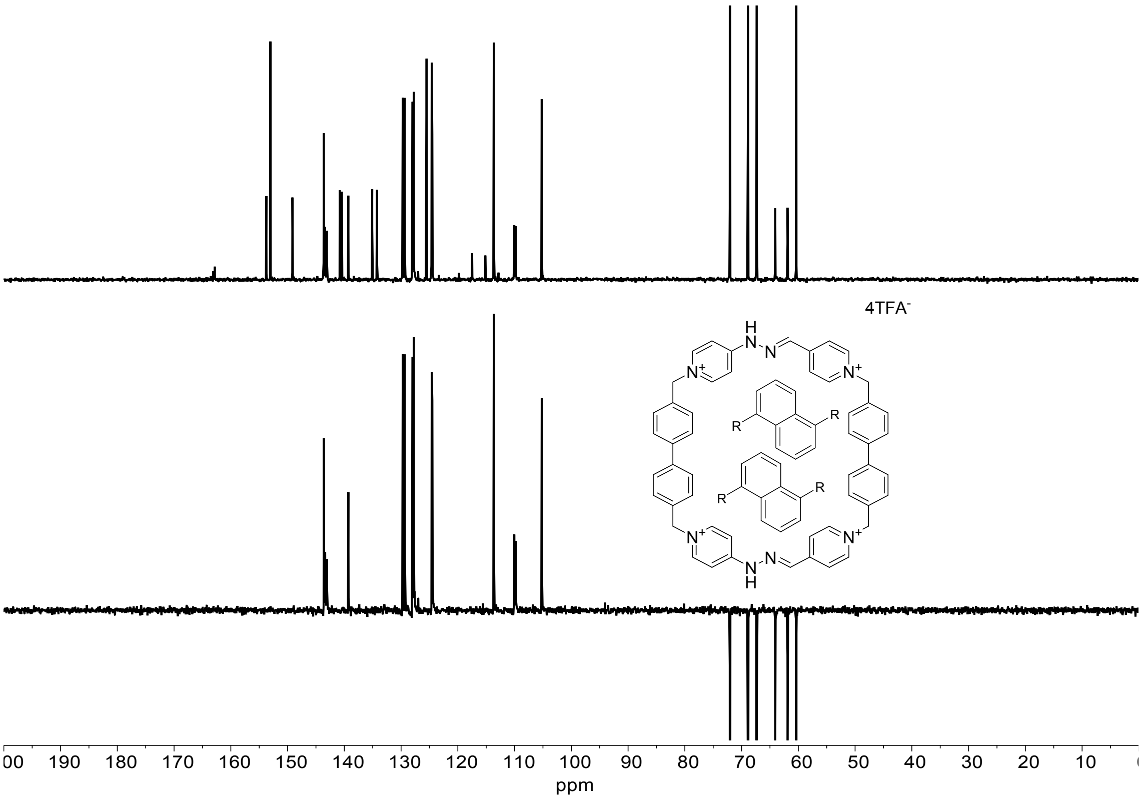

Figure S 154. $^{13}C\{^{1}H\}$ NMR (126 MHz, D_2O) spectrum (top) and DEPT-135 (126 MHz, D_2O) spectrum (bottom) of $\mathbf{4_2}\subset\mathbf{R_e}H_2\cdot$4TFA at pD=5

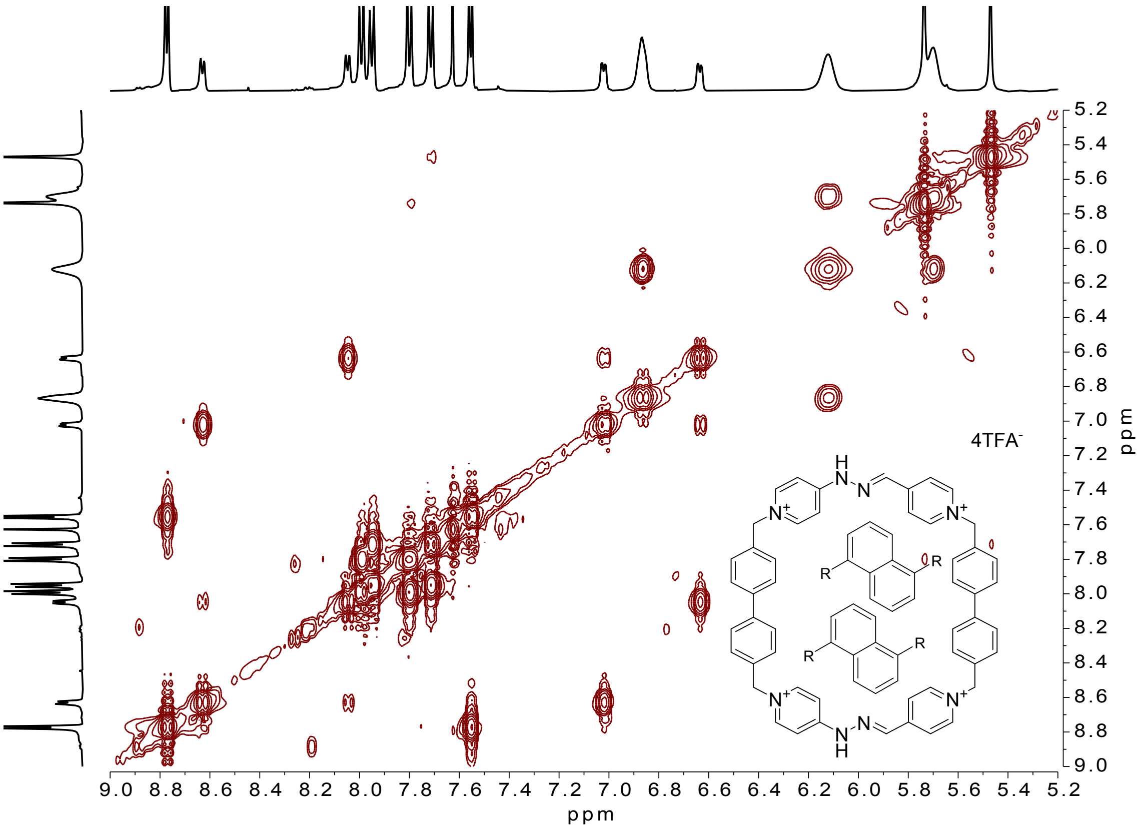

Figure S 155. ^{1}H-^{1}H COSY (500 MHz, D_2O) spectrum of $\mathbf{4_2}\subset\mathbf{R_e}H_2\cdot$4TFA at pD=5

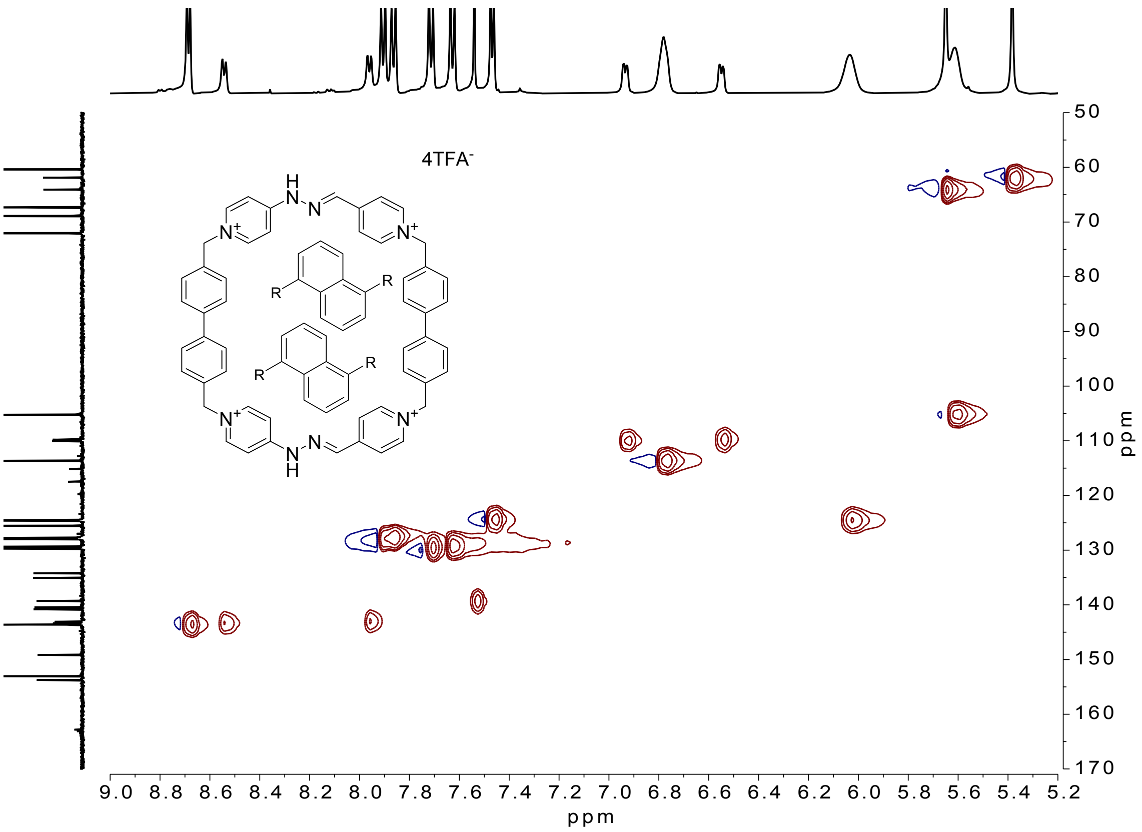

Figure S 156. ^{1}H-^{13}C HSQC (500 MHz, D_2O) spectrum of $\mathbf{4_2}\subset\mathbf{R_e}H_2\cdot 4TFA$ at pD=5

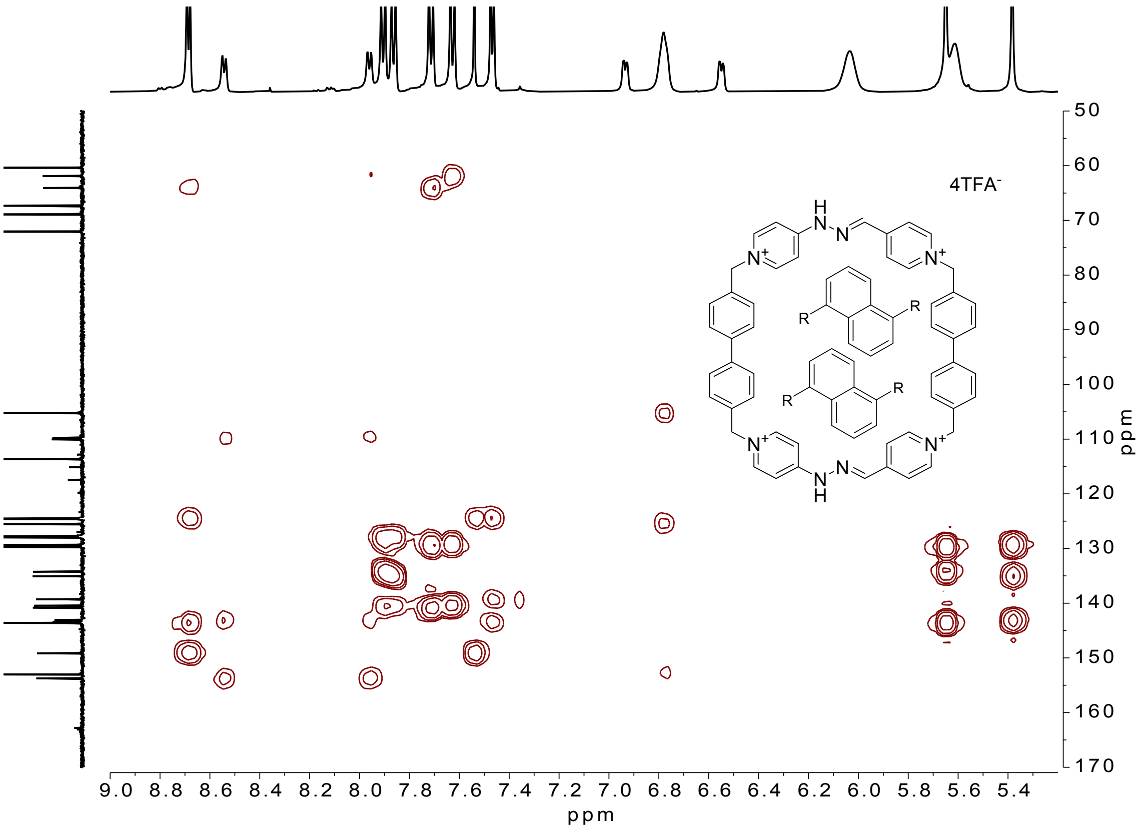

Figure S 157. ^{1}H-^{13}C HMBC (500 MHz, D_2O) spectrum of $\mathbf{4_2}\subset\mathbf{R_e}H_2\cdot 4TFA$ at pD=5

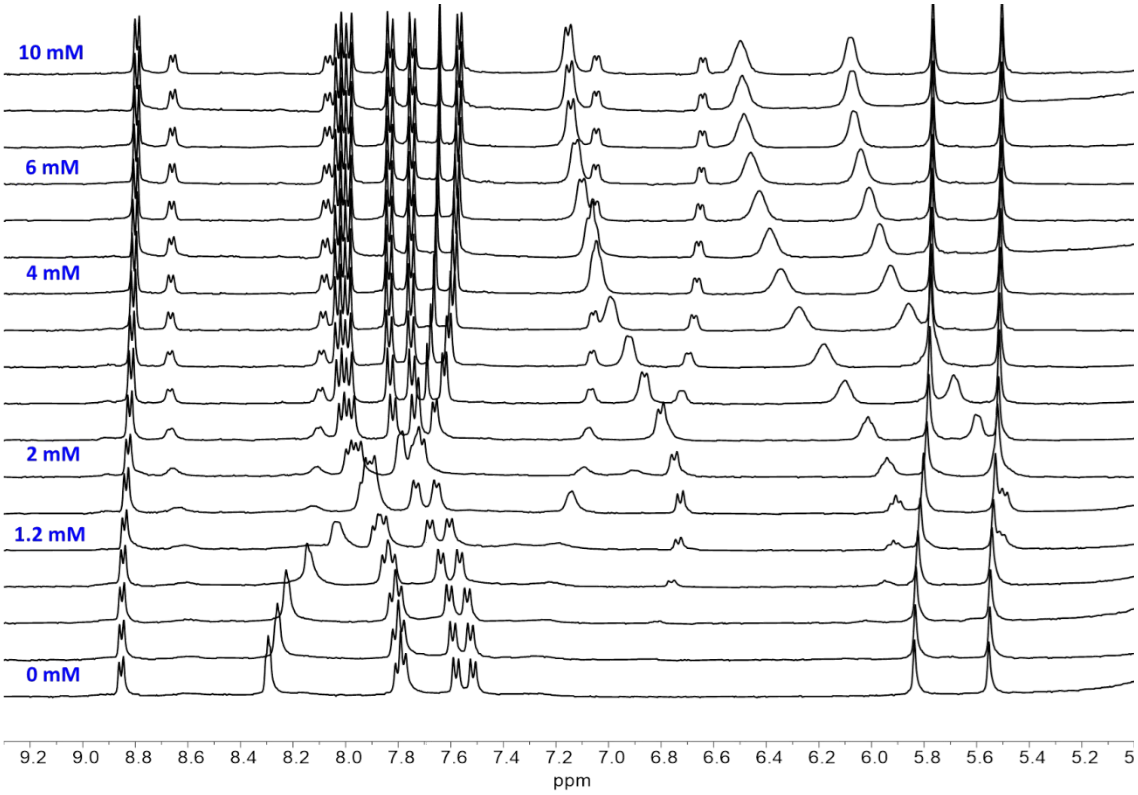

Figure S 158. ^{1}H NMR (400 MHz, D_2O) spectra of $\mathbf{R_e}H_2$·4TFA (1.3 mM) upon titration with **4** (15 mM) at pD=5

The mechanism and adjustable parameters for the fitting task proposed for the fitting process equilibria, and introduced on the software Dynafit,[3] were the following:

$$\mathbf{R_e}H_2^{4+} + \mathbf{4} \leftrightarrow \mathbf{4}\square\mathbf{R_e}H_2^{4+}$$

$$\mathbf{4}\square\mathbf{R_e}H_2^{4+} + \mathbf{4} \leftrightarrow \mathbf{4_2}\square\mathbf{R_e}H_2^{4+}$$

```
[task]
task = fit  ;  simulate | design
data =  equilibria ;  progress | rates | equilibria | generic
[mechanism]
RB + DH <===>  RBDH: Ka1 equilibria
RBDH + DH <===>  RBDHDH : Ka2 equilibria
[constants]
Ka1 = 0.01?
Ka2 = 0.001?
[concentrations]
RB = 1300; μM
[responses]
intensive
[data]
variable DH
```

plot titration

set Hf | resp RB = 8.2948 ,RBDHDH = 7.6422 ?

set Hg | resp RB = 8.2958 , RBDHDH = 7.5667 ?

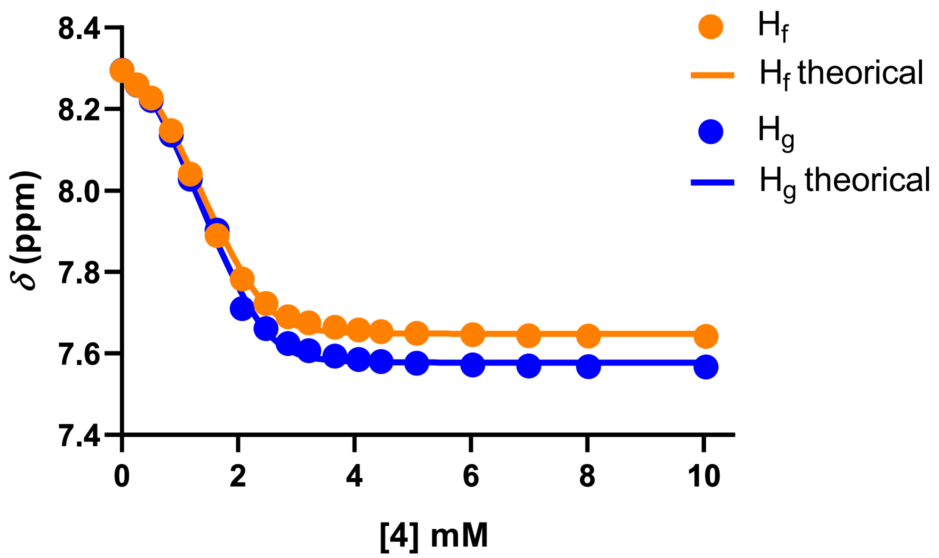

Figure S 159. Fitting of experimental data of the titration of $\mathbf{R_e}H_2$·4TFA (1.3 mM) with **4** (15 mM) at pD=5 (squares) for signals H_f (orange) and $\mathbf{H_g}$ (blue)

Table S 8. Experimental data of the titration of $\mathbf{R_e}H_2$·4TFA (1.3 mM) with **4** (15 mM) at pD=5 for signals H_f and $\mathbf{H_g}$. The *p* value obtained from "run-of-sign" was 0.093[4]

[1,5-DHNc] (mM)	δ H_f (ppm)	Residual H_f	δ $\mathbf{H_g}$ (ppm)	Residual $\mathbf{H_g}$
0.0	8.2948	0.015267	8.2958	0.00779
0.3	8.2597	-0.00384	8.2590	-0.01098
0.5	8.2267	0.00169	8.2211	-0.00548
0.9	8.1467	0.00155	8.1366	0.00001
1.2	8.0407	-0.00923	8.0281	-0.00120
1.6	7.8899	-0.01743	7.9033	0.03467
2.0	7.7832	-0.01035	7.7108	-0.02963
2.5	7.7232	0.00229	7.6615	0.00292
2.9	7.6905	0.00734	7.6243	0.00826
3.2	7.6758	0.00992	7.6073	0.01073
3.7	7.6644	0.00802	7.5933	0.00773
4.1	7.6577	0.00521	7.5853	0.00381
4.5	7.6534	0.00280	7.5801	0.00074

5.1	7.6499	0.00085	7.5760	-0.00161
6.0	7.6466	-0.00138	7.5720	-0.00441
7.0	7.6439	-0.00362	7.5692	-0.00669
8.0	7.6430	-0.00426	7.5676	-0.00800
10.0	7.6422	-0.00483	7.5667	-0.00864

To corroborate that the fitting does not reflect a non-cooperative binding mode, the mechanism and adjustable parameters for the fitting task proposed for the fitting process equilibria, and introduced on the software Dynafit,[3] were the following:

$$\mathbf{R_e}\mathrm{H}_2^{4+} + \mathbf{4} \leftrightarrow \mathbf{4}\square\mathbf{R_e}\mathrm{H}_2^{4+}$$

$$\mathbf{4}\square\mathbf{R_e}\mathrm{H}_2^{4+} + \mathbf{4} \leftrightarrow \mathbf{4_2}\square\mathbf{R_e}\mathrm{H}_2^{4+}$$

```
[task]
task = fit  ;  simulate | design
data =  equilibria ;  progress | rates | equilibria | generic
[mechanism]
RB + DH <===>  RBDH: Ka1 equilibria
RBDH + DH <===>  RBDHDH : Ka2 equilibria
[constants]
Ka1 = 4 * Ka2
Ka2 = 0.001?
[concentrations]
RB = 1300; µM
[responses]
intensive
[data]
variable DH
plot titration
set Hf   | resp RB = 8.2948   ,RBDHDH = 7.6422 ?
set Hg  | resp RB = 8.2958  , RBDHDH = 7.5667 ?
```

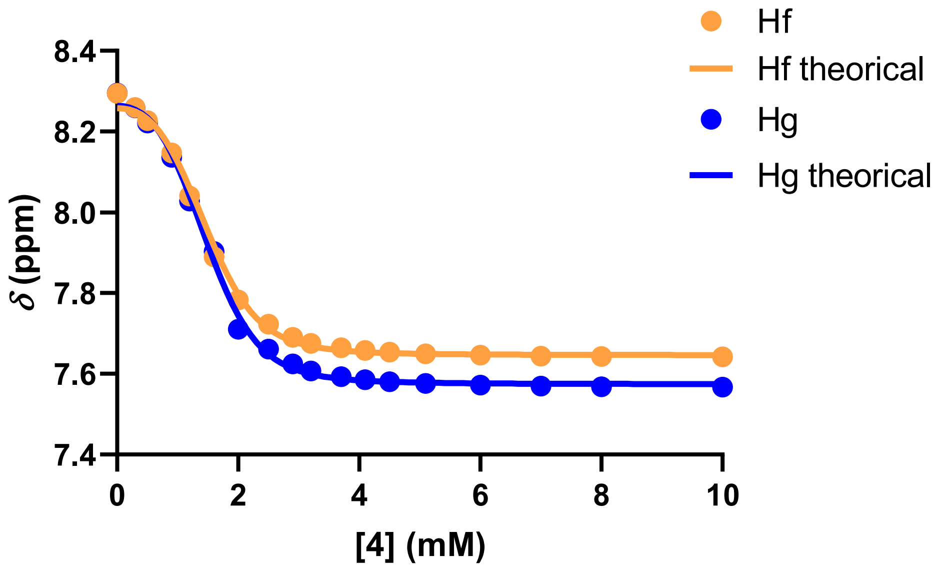

Figure S 160. Fitting of experimental data of the titration of $\mathbf{R_e}H_2$·4TFA (1.3 mM) with **4** (15 mM) at pD=5 (squares) for signals H_f (orange) and $\mathbf{H_g}$ (blue) to a non-cooperative binding system

Table S 9. Experimental data of the titration of $\mathbf{R_e}H_2$·4TFA (1.3 mM) with **4** (15 mM) at pD=5 for signals H_f and $\mathbf{H_g}$ to a non-cooperative binding system. The *p* value obtained from "run-of-sign" was 0.0023 (<0.05).[4]

[1,5-DHNc] (mM)	δ H_f (ppm)	Residual H_f	δ H_g (ppm)	Residual H_g
0.0	8.2948	0.03681	8.2958	0.03189
0.3	8.2597	0.00785	8.2590	0.00202
0.5	8.2267	-0.00377	8.2211	-0.01178
0.9	8.1467	-0.02052	8.1366	-0.02498
1.2	8.0407	-0.02839	8.0281	-0.02289
1.6	7.8899	-0.01402	7.9033	0.03848
2.0	7.7832	0.00132	7.7108	-0.01646
2.5	7.7232	0.00774	7.6615	0.00910
2.9	7.6905	0.00697	7.6243	0.00789
3.2	7.6758	0.00764	7.6073	0.00822
3.7	7.6644	0.00583	7.5933	0.00532
4.1	7.6577	0.00362	7.5853	0.00208
4.5	7.6534	0.00171	7.5801	-0.00042
5.1	7.6499	0.00033	7.5760	-0.00214
6.0	7.6466	-0.00142	7.5720	-0.00439
7.0	7.6439	-0.003415	7.5692	-0.00639

8.0	7.6430	-0.00392	7.5676	-0.00754
10.0	7.6422	-0.00434	7.5667	-0.00802

2.5. DOSY spectrum of 4□R$_b$H$_2$·4TFA /4$_2$□R$_{d\text{-}e}$H$_2$·4TFA

2.5.1. DOSY spectrum of 4□R$_b$H$_2$·4TFA

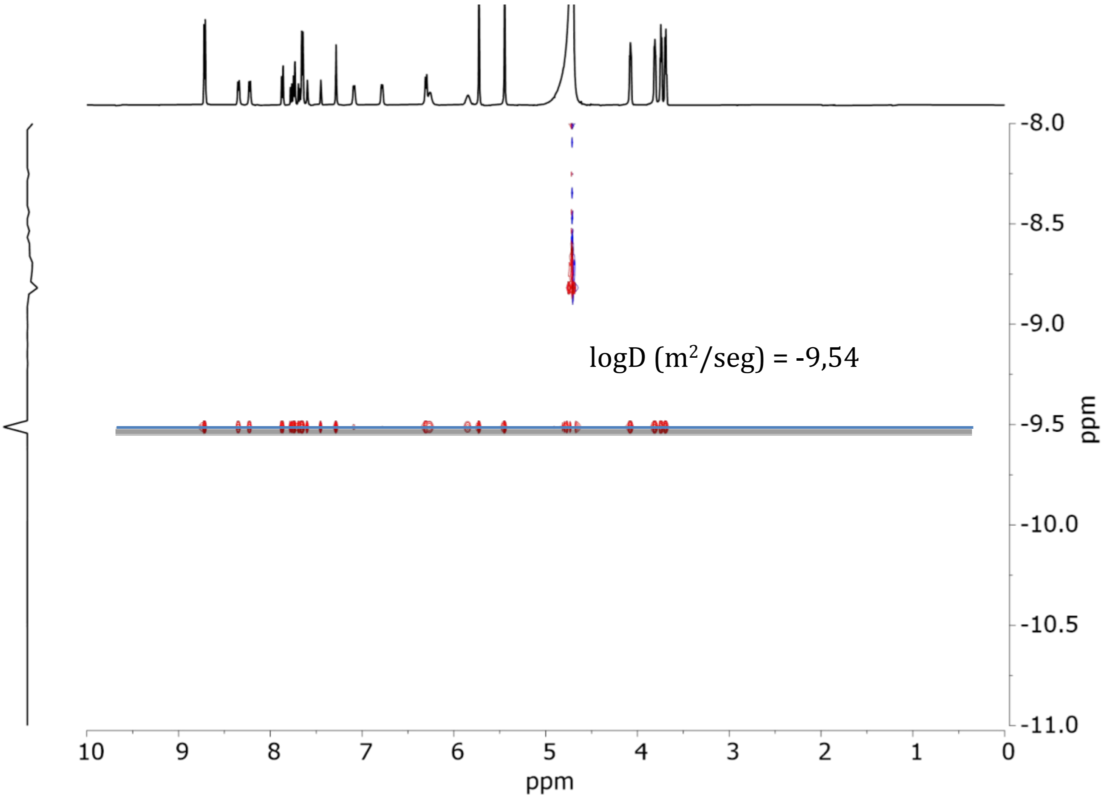

Figure S 161. DOSY (500 MHz, D_2O) spectrum of **4**⊂**R$_b$**H$_2$·4TFA at 2mM and pD=5

2.5.2. **DOSY spectrum of 4_2□R_dH_2·4TFA**

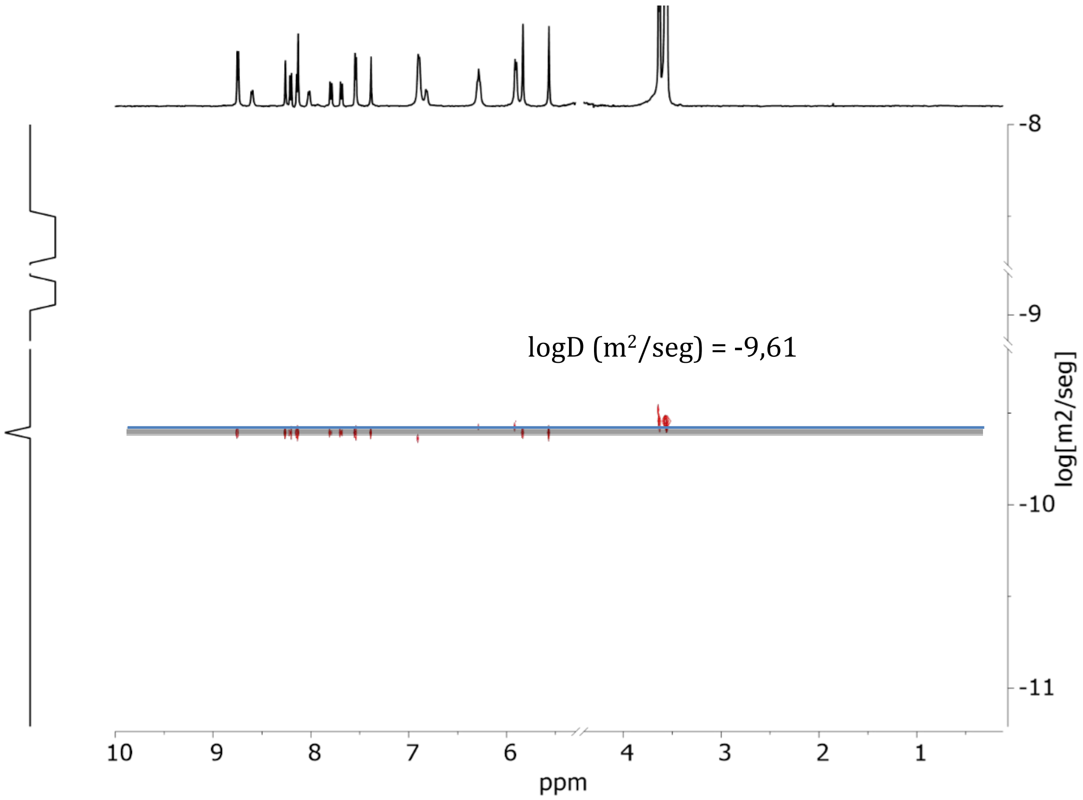

Figure S 162. DOSY (500 MHz, D_2O) spectrum of $\mathbf{4_2 \subset R_d}H_2$·4TFA at 1.3 mM and pD=5

2.5.3. **DOSY spectrum of 4_2□R_eH_2·4TFA**

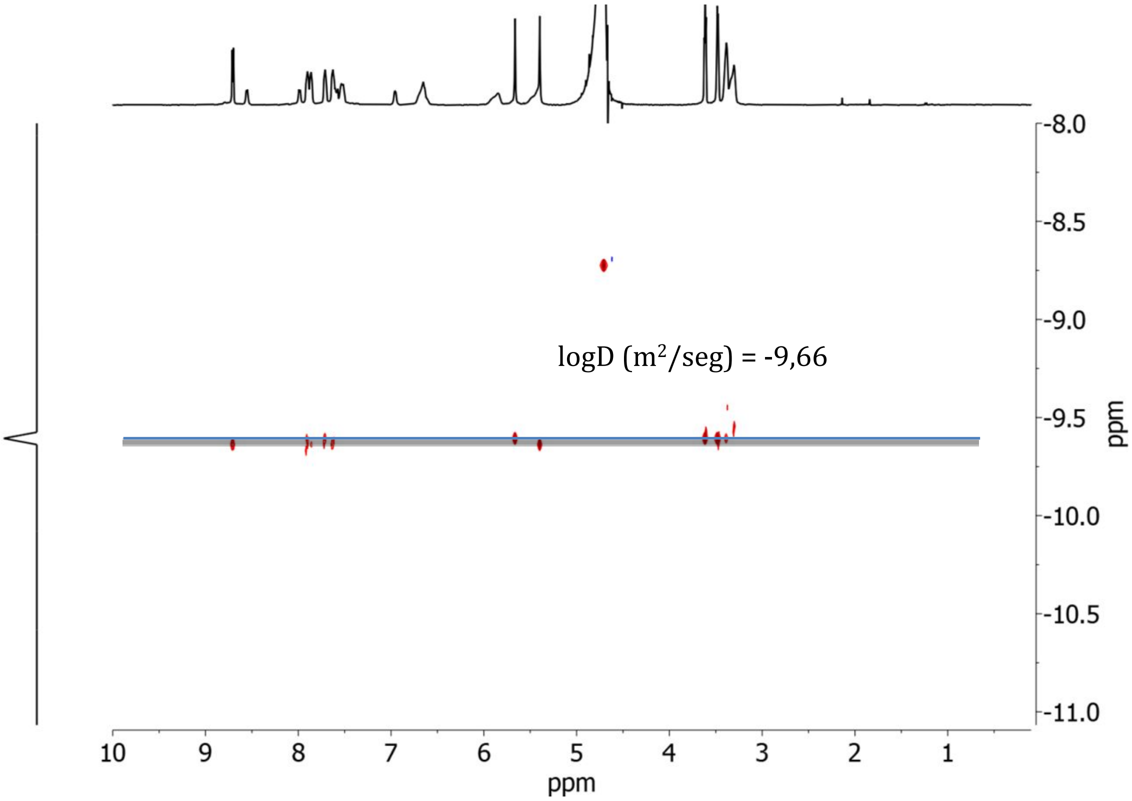

Figure S 163. DOSY (500 MHz, D_2O) spectrum of $\mathbf{4_2 \subset R_e}H_2$·4TFA at 1.3 mM and pD=5

3. COMPUTATIONAL DETAILS

All calculations reported herein were performed using the free-available program packages ORCA 6.0.0,[5] CREST 2.11.2,[6] CENSO 1.2.0.[7] and xTB 6.6.0.[8] Unless otherwise indicated, computations were carried out with default settings. Initial geometries for the different species discussed in the manuscript were generated by hand using the AVOGADRO software.[9] Cartesian coordinates for the geometries discussed herein have been included as xyz files in the supplementary file S1.zip.

As shown in **Figure S160**, the computational analysis of macrocycle $\mathbf{R_c}H_2^{4+}$ started with a conformational search carried out using the Conformer–Rotamer Ensemble Sampling Tool (CREST) with standard settings at the GFN2-xTB/ALPB(water) level,[8,10] producing as output a conformational ensemble of 43 unique structures within a 20 kcal/mol energy window.

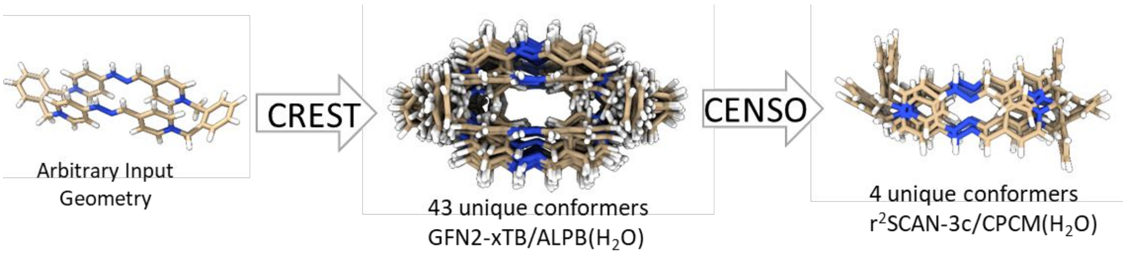

Figure S 164. Conformational ensemble generation (CREST),[6] and refinement (CENSO),[7] for macrocycle $\mathbf{R_c}H_2^{4+}$.

This initial ensemble was further refined at the DFT level employing the Command-line ENergetic Sorting software (CENSO),[7] using part_1 (screening) and part_2 (optimization) of the protocol as explained in the original publication.[7] In brief, the CENSO algorithm allows for the iterative refinement of conformational ensembles at increasingly more accurate levels of theory, and on the basis of free energies for the conformers estimated as G°(conformer) = [E_{gas}^{DFT}(conformer) + δ_{solv}(conformer)] + G_{mRRHO}°(conformer).

[5] Neese, F. Software update: The ORCA program system—Version 5.0. *WIREs Comput Mol Sci*. **2022**, 12, e1606.
[6] Pracht, P.; Bohle, F.; Grimme, S. Automated exploration of the low-energy chemical space with fast quantum chemical methods*, Phys. Chem. Chem. Phys.*, **2020**, *22*, 7169-7192.
[7] Grimme, S.; Bohle, F.; Hansen, A.; Pracht, P.; Spicher, S.; Stahn, M. Efficient Quantum Chemical Calculation of Structure Ensembles and Free Energies for Nonrigid Molecules. *J. Phys. Chem. A* **2021**, *125*, 4039-4054.
[8] Bannwarth, C.; Caldeweyher, E.; Ehlert, S.; Hansen, A.; Pracht, P.; Seibert, J.; Spicher, S.; Grimme, S. Extended tight-binding quantum chemistry methods. *WIREs Comput. Mol. Sci.* **2021**, 11, e1493
[9] Hanwell, M. D.; Curtis, D. E.; Lonie, D. C.; Vandermeersch, T.; Zurek, E.; Hutchison, G. R. Avogadro: An advanced semantic chemical editor, visualization, and analysis platform. *J. Cheminform.* **2012**, *4*, 17.
[10] Ehlert, S.; Stahn, M.; Spicher, S.; Grimme, S. Robust and Efficient Implicit Solvation Model for Fast Semiempirical Methods. *J. Chem. Theory Comput.* **2021,** *17*, 4250-4261.

Consequently, part_1 (screening) ranks the initial ensemble of semiempirically-optimized conformers on a 5 kcal/mol window on the basis of G°(conformer) computed as $E_{\mathrm{gas}}^{\mathrm{DFT}}$(conformer) + δ_{solv}(conformer) obtained by single points at the r^{2}SCAN-3c/CPCM(water) level,[11] and thermostatistical contributions $G_{\mathrm{mRRHO}}^{\circ}$ obtained by single point Hessian calculations at the GFN2-xTB/ALPB(water) level. On the other hand, Part_2 (optimization) ranks the subsequent ensemble from part_1 on a 3.0 kcal/mol free energy window at the same levels of theory discussed for part_1, but only after optimizing first the ensemble at the r^{2}SCAN-3c/CPCM(water) level.[12][13]

The CENSO protocol yielded a refined ensemble of 4 unique conformers for $\mathbf{R_c}\mathrm{H_2}^{4+}$ arbitrarily labeled as the atropisomers (u,u)-/(u,d)-/(d,u)-/(d,d)- $\mathbf{R_c}\mathrm{H_2}^{4+}$ discussed in the text. The nature of the obtained geometries as minima on the r^{2}SCAN-3c/CPCM(water) potential energy surface, was corroborated by frequency calculations, which allowed as well for the refined estimation of the free energies of the conformers at this level of theory (**Table S7**):

Table S 10. Free energies at the r^{2}SCAN-3c/CPCM(water) level of theory for the geometries discussed in the text for species (u,u)-/(u,d)-/(d,u)-/(d,d)-$\mathbf{R_c}\mathrm{H_2}^{4+}$, TS$_a$-TS$_d$, **4t**□$\mathbf{R_b}\mathrm{H_2}^{4+}$ and $\mathbf{4t_2}$□$\mathbf{R_d}\mathrm{H_2}^{4+}$. The magnitude of the negative frequencies for maxima TS$_a$-TS$_d$ also included.

Geometry ($\mathbf{R_c}\mathrm{H_2}^{4+}$)	Free Energy (hartrees)	Negative Frequencies (cm^{-1})
(u,u)-	-1906.047256	none
TS$_a$	-1906.016014	-65.12
(u,d)-	-1906.047736	none
TS$_b$	-1906.014746	-35.56
(d,d)-	-1906.047023	none
TS$_c$	-1906.014063	-46.32
(d,u)-	-1906.047648	none
TS$_d$	-1906.015778	-58.46
**4t**□$\mathbf{R_b}\mathrm{H_2}^{4+}$	-2520.69860281	none
$\mathbf{4t_2}$□$\mathbf{R_d}\mathrm{H_2}^{4+}$	-3442.47557283	none

[11] Spicher, S.; Grimme, S. Single-Point Hessian Calculations for Improved Vibrational Frequencies and Rigid-Rotor Harmonic-Oscillator Thermodynamics. *J. Chem. Theory Comput.* **2021,** *17*, 1701-1714.
[12] Grimme, S.; Hansen, A.; Ehlert, S.; Mewes, J. M. r2SCAN-3c: A “Swiss army knife” Composite Electronic-structure Method. *J. Chem. Phys.* **2021**, *154*, 064103.
[13] Miertus, S.; Scrocco, E.; Tomasi, J. Electrostatic Interaction of a Solute With a Continuum. A Direct Utilization of Ab Initio Molecular Potentials for the Prevision of Solvent Effects. *J. Chem. Phys.* **1981**, *55*, 117-129.

In order to obtain the different interconversion barriers reported in the manuscript, the Nudged Elastic Band algorithm was used as implemented in ORCA 6.6.0,[14] allowing for the obtention of minimum energy paths (MEPs), connecting the different atropisomers at the r^2SCAN-3c/CPCM(water) level discussed above. From the obtained MEPs, transition state guesses for the interconversions were obtained, and used as starting points for the optimization of the geometries by using an eigenvector following the procedure as implemented in ORCA 6.6.0. The resulting geometries for the transition states TS_a-TS_d were verified as maxima on the corresponding potential energy surfaces by frequency analysis (one negative frequency, **Table S7**). IRC calculations,[15] were used to corroborate the correspondence between the proposed TSs and the connected atropisomers.

In the case of the host-guest complexes **4t□R**$_{\mathbf{b}}H_2^{4+}$ and **4t**$_2$□**R**$_{\mathbf{d}}H_2^{4+}$, initial geometries were prepared by hand using the AVOGADRO software considering the co-conformational restrictions inferred in each case from the NMR data. The structures were then optimized at the r^2SCAN-3c/CPCM(water) level of theory, and subsequently corroborated as minima on the corresponding potential energy surface by frequency calculations (**Table S7**).

[14] Ásgeirsson, V.; Birgisson, B. O.; Bjornsson, R.; Becker, U.; Neese, F.; Riplinger, C.; Jónsson, H. Nudged Elastic Band Method for Molecular Reactions Using Energy-Weighted Springs Combined with Eigenvector Following. *J. Chem. Theory Comput.* **2021**, *17*, 4929-4945.

[15] Maeda, S.; Harabuchi, Y.; Ono, Y.; Taketsugu, T.; Morokuma, K. Intrinsic reaction coordinate: Calculation, bifurcation, and automated search. *Int. J. Quantum Chem.* **2015**, *115*, 258–269.
